# Site‐Selective B─H Activation via HAT Toward Xanthyl‐*closo*‐Carboranes as Bench‐Stable Precursors of Organosulfur Boron Clusters

**DOI:** 10.1002/anie.7686382

**Published:** 2026-04-22

**Authors:** Marco Rusconi, Eugenia Magi, Polyssena Renzi, Emanuele Azzi, Valeria Lagostina, Enrico Salvadori, Mario Chiesa, Birgit Hischa, Giovanni Ghigo, Annamaria Deagostino

**Affiliations:** ^1^ Department of Chemistry University of Torino Torino Italy; ^2^ Department of X‐ray Structure Analysis University of Regensburg Regensburg Germany

**Keywords:** B─H activation, boryl radicals, carboranes, HAT, nitrogen centered radicals, visible light, xanthates

## Abstract

In this work, a light mediated direct regioselective B─H functionalization to afford unexplored xanthyl‐*closo*‐carboranes is described by a nitrogen‐centered radical (NCR)‐mediated hydrogen atom transfer (HAT) process. To directly activate a B─H bond in an icosahedral carborane, which contains 10 B─H with very similar electronic properties, represents a challenging goal. The reaction is applied to several mono and disubstituted *o*‐ and *m*‐carboranes affording, in discrete to good yields, boryl xanthates which act as novel bench‐stable and versatile platforms for the functionalization of the carborane cage with sulfur‐containing functionalities: alkyl‐ and aryl sulfides, thiols, thioesters and sulfonyl chlorides are readily obtained. The reaction has also been scaled up and an analogue of the pesticide Chlorbenside has been synthesized. EPR studies, additionally to deuteration experiments, confirm the formation of an NCR as the promoter of a HAT process to the most electron‐rich boron vertex with the lowest B─H bond dissociation energy (BDE), to produce a B(9)‐centered radical. A deep computational study contributes to the mechanism proposal.

## Graphical abstract

A light mediated direct regioselective B─H functionalization, via hydrogen atom transfer (HAT), is described. Unexplored xanthyl‐*closo*‐carboranes as novel bench‐stable and versatile platforms to access sulfur‐containing boron clusters are reported for the first time. A robust combination of experimental and theoretical studies elucidates the radical reaction mechanism.

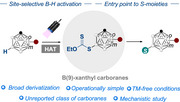

## 1 Introduction

Carboranes are icosahedral clusters [1], distinguished by unique electronic and structural properties. Their rigid three‐dimensional framework has positioned them as emerging motifs in modern medicinal chemistry, in line with a recent paradigm shift toward sp^3^‐enriched molecular topologies to effectively target biomolecules [2, 3]. Accordingly, suitably functionalized carboranes are employed as analogues of aromatic moieties in known drugs [4, 5, 6, 7], or, owing to their high boron content, as promising boron neutron capture therapy (BNCT) agents [8, 9, 10, 11, 12]. Beyond medicinal applications, carboranes serve as valuable building blocks in nanomaterials design, carborane‐based luminescent materials, and as robust ligands in coordination and organometallic chemistry [13, 14]. This versatility places the development of efficient synthetic strategies aimed at targeting specific vertices within carborane cages as a longstanding challenge in the field.

Conventional approaches to vertex‐specific decoration rely on the intrinsic orthogonal reactivity of boron and carbon sites [1, 15]. The mildly acidic C─H vertices readily undergo reactions with electrophiles following deprotonation, whereas the derivatization of the boron vertices can be achieved using a number of different strategies. The treatment of *o*‐carborane in liquid ammonia with alkali metals, followed by oxidation, affords 3‐amino‐*o*‐carboranes [16, 17, 18]. B‐halo and alkyl derivatives can be prepared under Friedel/Crafts type reaction conditions, requiring high temperatures, long reaction time and strong Lewis acids as the catalysts, not compatible with several functional groups [19, 20]. Therefore, the regioselective discrimination of a single B─H vertex among the 10 boron sites within a carborane cage, in mild conditions, remains highly challenging [21]. The closely related chemical environment and orbital hybridization allow only subtle differences in both B─H bond dissociation energies (BDEs) and electron densities, which decrease in the order B(9,12) > B(8,10) > B(4,5,7,11) > B(3,6) in *o*‐carboranes.

**FIGURE 1:**
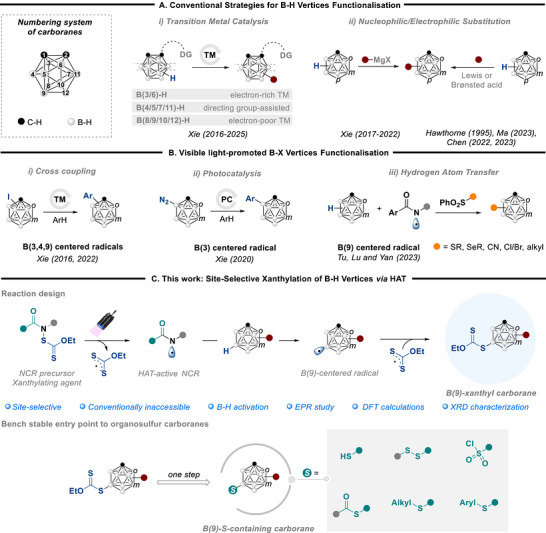
(A) Functionalization of B─H vertices in *closo*‐carboranes. (B) Photoinduced strategies for the generation of B‐centered carboranyl radicals. (C) Photoinduced B─H xanthylation of *closo*‐carboranes via HAT.

Nonetheless, recent strategies elegantly exploit the B─H site polarity matching in nucleophilic and electrophilic substitution reactions (Figure 1A). The more electron‐deficient B─H vertices readily undergo nucleophilic substitution with Grignard reagents, enabling B‐alkylation and arylation [22, 23]. In contrast, the most electron‐rich B(9)‐vertex reacts with electrophiles in the presence of activating systems such as trifluoromethanesulfonic acid (HOTf)/hexafluoroisopropanol (HFIP) [24, 25, 26, 27, 28, 29], consistently with a preference of *o*‐carboranes for soft electrophiles [30], allowing site‐selective B‐halogenation, hydroxylation, and amination to be accomplished. Transition metal catalysis also covers a prominent role, as demonstrated by predominant contributions from the group of Xie, that elaborated a general strategy for the B─H vertex‐selective functionalization of *o*‐carboranes [31, 32, 33]. These methods mostly employ iridium [34, 35, 36, 37, 38, 39, 40, 41] and palladium [42, 43, 44, 45, 46, 47], but also copper, nickel, and ruthenium, assisted by cage‐bonded directing groups [48, 49, 50].

Lately, the formation of B‐centered *closo*‐carboranyl radicals has emerged as a valuable alternative route to cage functionalization via orthogonal pathways to classical closed‐shell reactions (Figure 1B). Besides an early seminal example based on a Mn‐catalyzed oxidative process [51], light‐mediated methodologies have become pivotal to access B‐carboranyl radicals from either B‐iodo [52, 53], B‐diazonium carboranes [54], or by decarboxylation of carborane carboxylic acids [55, 56]. In this context, only one example from Yan and co‐workers described the direct site‐selective B─H activation of *closo*‐clusters, achieved through a nitrogen‐centered radical (NCR)‐mediated hydrogen atom transfer (HAT) strategy to generate B‐centered carboranyl radicals, without prior substrate pre‐functionalization [57]. The same group has recently reported the B─H functionalization of carboranes via the synergistic exploitation of ligand‐to‐metal charge transfer (LMCT) and HAT [58].

Since our group has been working for several years on the synthesis of new Gd‐ and B‐NCT agents based on appropriately functionalized carboranes [59, 60, 61, 62], we sought to expand the accessible chemical space in carborane chemistry by exploiting a NCR‐promoted HAT process to directly introduce new functionalities on unactivated *closo*‐carboranes. As extensively reported by Alexanian and co‐workers [63, 64, 65, 66, 67], sterically and electronically fine‐tuned amidyl radicals (N─H BDE >100 kcal/mol) [68] efficiently accomplish remote aliphatic C(sp^3^)─H and decarboxylative functionalization [69]. Supported by computational analysis, we envisaged that an analogous system could render a visible light‐enabled HAT process thermodynamically feasible at the vertex exhibiting the weakest BDE (B(9)─H BDE = 103.9 kcal/mol), thereby generating a B‐carboranyl radical that could be trapped with a functionality prone to be manipulated, offering a pathway to obtain differently substituted *closo*‐carboranes. In order to maximize functional differentiation, and in light of the limited representation of boryl xanthates in the literature [70, 71], we identified the xanthate (dithiocarbonate) group as an attractive target due to its rich chemistry [72, 73, 74, 75, 76, 77, 78, 79, 80]. Herein, we describe for the first time the synthesis and the synthetic applications of *B*‐xanthyl carboranes as a previously unreported class of carborane derivatives that serve as bench‐stable and versatile linchpin to *B*‐bonded *S*‐containing carborane frameworks. (Figure 1C).

## 2 Results and Discussion

To verify the feasibility of the envisaged HAT process enabled by in situ generated NCRs, xanthylamide **2a** (2 eq., N─H BDE = 111 kcal/mol) was subjected to purple light irradiation (390 nm, 40 W) in the presence of *m*‐carborane **1a** in a PhCF_3_ solution (0.5 M) at room temperature for 22 h. Under these conditions, only B(9)‐xanthyl‐*m*‐carborane **3a** was isolated in 49% yield (Table 1, Entry 1). The structure was confirmed by spectroscopic methods and single‐crystal x‐ray diffraction. Cognizant of the influence of steric and electronic effects on the BDEs [81], a series of xanthyl transfer agents (**2a**‐**e**) was subsequently evaluated (selected results are summarized in Table 1; see Table S1 for the complete optimization study). Among them, beside **2a**, only **2b** (N─H BDE = 111 kcal/mol) afforded the desired product **3a** in 41% yield, (Entry 2). Coherently with the requirement of sufficiently strong BDEs to accomplish a HAT process at the B(9)─H vertex of **1a**, the use of xanthyl transfer agents **2c‐e** led to suppression of product formation (Entry 17). Having identified **2a** as the optimal xanthylating agent for our system, the effect of the substrate concentration on the efficiency of the process was studied. While employing 2 eq. of **2a**, the dilution of the PhCF_3_ solutions to 0.3, 0.1, and 0.05 M only slightly affected the yield of **3a** (40%, 42%, 40%, respectively; Entries 3–5). On this basis, a concentration of 0.5 M was initially selected for further evaluation of **2a** stoichiometry. Reducing the amount of **2a** led to diminished yields (Entries 6 and 7), whereas increasing the loading to 3 and 4 eq. in PhCF_3_ (0.5 M for **1a**; Entries 8 and 9) caused formation of a heterogeneous slurry, resulting in compromised stirring and decreased irradiation efficiency, and ultimately in significantly lower yields (16% and 10%, respectively). To overcome this issue, we adjusted the concentration to 0.1 M while employing 4 eq. of **2a**, successfully isolating **3a** in 64% yield (Entry 10) as the unique B(9)‐regioisomer. While evaluating solvent effects, PhCl gave a lower yield (42%; Entry 11), whereas 1,2‐DCE and a 1,2‐DCE/PhCF_3_ (1:2) solution resulted in greatly reduced reactivity (Entries 12 and 13), likely due to undesired solvent‐mediated quenching of the NCR via HAT. Additionally, blue LEDs proved to be a less effective irradiation source for the photogeneration of the NCR from **2a** (Entry 14). Control experiments conducted in the absence of light and open‐air conditions translated into complete or partial suppression of product formation (Entries 15 and 16), suggesting a photoinduced operative mechanism with the involvement of radical intermediates.

**TABLE 1:** Selected results for the optimization.

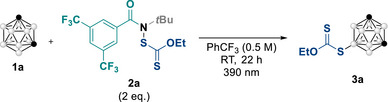		
Entry	Deviation	3a yield^b^
1	None	49
2	**2b** instead of **2a**	41
3	PhCF_3_ 0.3 M	40
4	PhCF_3_ 0.1 M	42
5	PhCF_3_ 0.05 M	40
6	**2a** 1.0 eq.	25
7	**2a** 1.5 eq.	37
8	**2a** 3.0 eq.	16
9	**2a** 4.0 eq.	10
**10**	**2a** 4.0 eq, PhCF_3_ 0.1 M	**64**
11	**2a** 4.0 eq, PhCl 0.1 M	42
12	**2a** 4.0 eq, 1,2‐DCE 0.1 M	28
13	**2a** 4.0 eq., 1,2‐DCE/ PhCF_3_ (1:2) 0.1 M	36
14	Blue LEDs^c^	20
15	In dark	0
16	Open air	22
17	**2c‐e** instead of **2a**	0
Structures of xanthyl transfer agents [29]		
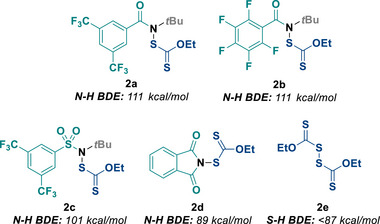		

^a^Reaction conditions: **1a** (0.15 mmol), **2a** (0.3 mmol, 2.0 eq.), freshly distilled and degassed PhCF_3_ (3 mL; 0.5 M), Kessil purple LEDs lamp (390 nm, 40 W), 22 h, RT.

^b^Isolated yield.

^c^Kessil LED A160WE Tuna Blue (40 W).

**SCHEME 1:**
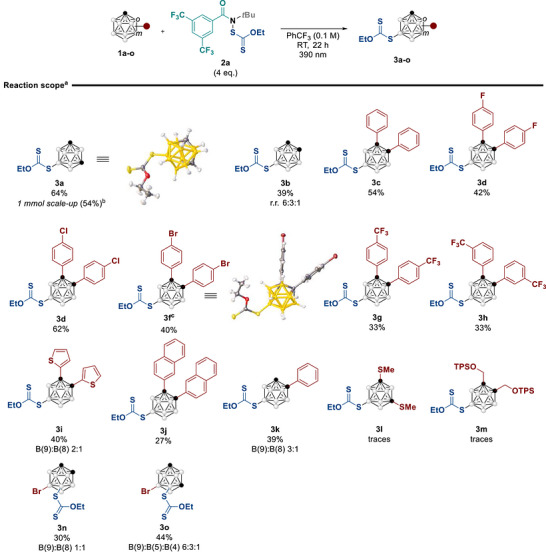
Substrate scope. ^a^Reaction Conditions: Unless otherwise noted, reactions were carried out in degassed PhCF_3_ on a 0.2 mmol scale using carborane **1a‐o** (1 eq.) and xanthylamide **2a** (4 eq.) under 390 nm irradiation (40 W) for 22 h. Yields and isomer ratio determined on the isolated products **3a‐o**. ^b^Isolated yield. ^c^Reaction run for 48 h.

Having established the optimized conditions for the synthesis of B‐xanthyl carboranes (Entry 10), we next turned our attention to the exploration of the scope and limitations of the reaction (Scheme 1).

To properly design and choose the substrates able to efficiently afford the carboranyl radical upon HAT promoted by NCRs, a computational study was carried out. C─H and B─H bond energies in some selected carboranes (see ESI, Tables S3.3.2a–i) were calculated, confirming that the weakest bonds are B(9,12)─H (103.5 kcal/mol) in *o*‐carborane, and B(9,10)─H (103.9 kcal/mol) in *m*‐carborane, as similarly reported in the literature [57]. Then, the study was extended to substituted carboranes demonstrating that aliphatic C─H and O─H bonds possess BDEs lower than 103.5/9 kcal/mol of B(9)─H, differently from aromatic C─H bonds (∼110 kcal/mol). In particular, the calculated C─H BDEs of differently substituted CH_3_ groups linked to carboranes fell in a range of 94–100 kcal/mol, the same for O─H bond which possess an estimated BDE of 101.6 kcal/mol. These results suggest that only the aryl‐substituted carboranes are suitable substrates for the activation by HAT of the B(9)─H bond. The protocol was firstly applied to both *m*‐ and *o*‐carboranes, **1a** and **1b**. Comparatively better yield and regioselectivity were observed for the *m*‐isomer delivering xanthate **3a** in 64% yield which was isolated as a single isomer, as confirmed by XRD analysis. Notably, this transformation could be successfully scaled up to 1 mmol in 54% yield. In contrast, the *o*‐isomer **1b** furnished an inseparable mixture of three regioisomers in 6:3:1 ratio, (**3b**, 39%). This outcome suggested that comparable site‐discrimination is not feasible in unbiased *o*‐carboranes, likely due to closely related B─H BDEs of the boron vertices. Difunctionalized *o*‐carboranes were subsequently investigated under optimized conditions. *C*,*C’*‐bis‐phenyl‐*o*‐carborane **1c** selectively afforded B(9) xanthyl derivative **3c** in 54% yield. Modifications of the aryl substituent were also tolerated. Best results were observed with 4‐ClPh derivative **1e**, affording **3e** in 62% yield, followed by 4‐BrPh and 4‐FPh derivatives, which furnished the corresponding B(9)‐xanthyl‐*o*‐carboranes **3d** and **3f** in 42% and 40% yield, respectively. In particular, compound **3f** has been characterized by XRD analysis, confirming functionalization at the B(9) position. This structural assignment enabled isomer identification of the other *o*‐carborane derivatives by comparison of their respective ^11^B NMR spectral patterns. 4‐CF_3_Ph (**1g**) and 3‐CF_3_Ph (**1h**) also reacted in complete regioselectivity toward the B(9)‐isomer (**3g** and **3h**), albeit in lower yields (33%). Conversely, lack of selectivity was observed in the case of *C*,*C*’‐bis‐(2‐thienyl)‐*o*‐carborane **1i**, yielding B(9) and B(8) isomers (**3i**) in 2:1 ratio, respectively. This behavior was attributed to the electron‐rich character of the thienyl ring, which may promote competing secondary HAT pathways. The corresponding B(9)‐xanthyl scaffold containing the 2‐naphthyl luminophore unit was obtained (**3j**) with complete site‐selectivity. As expected, loss of regiocontrol was observed in monosubstituted unsymmetric *o*‐carboranes, such as *C*‐phenyl‐*o*‐carborane **1k**, which afforded **3k** as an inseparable 3:1 mixture of regioisomers in 39% yield. To assess the compatibility of C(sp^3^)─H bonds under the reaction conditions, bis‐thioether (**1l**) was examined, which yielded only traces amount of xanthylated product (**3l**). Similar results were observed with the introduction of sterically hindered C(sp^3^)─H triphenylsilylether on the carborane cluster (**3m**), corroborating the unfavorable competition of C(sp^3^)─H bonds with B─H sites for the NCR during the HAT process. Finally, masking the adjacent boron vertex with a bromo group on both *o*‐ and *m*‐clusters (**1n** and **1o**) was tolerated, although with diminished regioselectivity, since products **3n** and **3o** were obtained as mixtures of xanthylated isomers. The regioisomer ratio was determined by the analysis of the signals pertinent to C─H cage bonds in the ^1^H‐NMR spectra of **3n** and **3o**, that were tentatively assigned to different regioisomers (B(9)/B(8) and B(9)/(B(5)/B(4), respectively) following the order of increasing calculated B─H BDEs, see ESI Tables S3.3.2a and S3.3.2 h.

**SCHEME 2:**
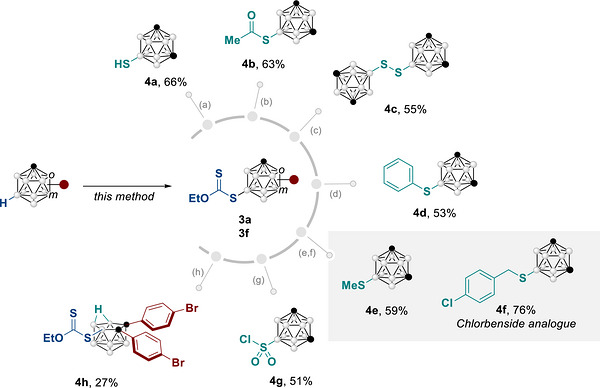
Synthetic derivatizations of B(9)‐xanthylcarboranes^i^. Reaction conditions: (a) EDA (4 eq.), EtOH, RT, 4 h; then H_2_SO_4_ (2 M). (b) EDA (4 eq.), EtOH, RT, 4 h; then Ac_2_O (4 eq.), Et_3_N (4 eq.), DCM, 0°C to RT, 6 h. (c) EDA (4 eq.), EtOH, RT, 4 h; then air, 48 h. (d) PhI (1.2 eq.), NiCl_2_DME (10 mol%), bpy (12 mol%), Zn (3 eq.), DMA, 110°C, 16 h. (e) EDA (4 eq.), EtOH, RT, 4 h; then MeI (10 eq.), RT, 16 h. (f) EDA (4 eq.), EtOH, RT, 4 h; then 1‐chloro‐4‐(iodomethyl)benzene (1 eq.), K_2_CO_3_ (1 eq.), DMF, RT, 16 h. (g) NCS (4 eq.), HCl (2 M), MeCN, 0°C to RT, 1 h. (h) CsF (8 eq.), EtOH, 75°C, 24 h. (i) Yields are calculated on the isolated products **4a‐h**

To demonstrate the value of B‐xanthyl carboranes as novel versatile platforms to introduce several sulfur‐containing functionalities directly at the boron vertex, synthetic manipulations of **3a** and **3f** were carried out (Scheme 2). Since the reactivity of B‐xanthates has never been reported, we firstly tested the classical conditions developed by Zard et al. [64, 65, 67] for C‐xanthates. Surprisingly, when visible light or radical initiators were used, no reaction was observed which makes B‐xanthates inert. This is probably due to the strength of B─S bond for which we calculated a value of 78.2 kcal/mol (to be compared with that calculated for the N─S bond in **2a** of 48.7 kcal/mol) and to the fact that in the first excited state of **3a** the B─S bond undergoes to a negligible elongation (from 1.89–1.91 Å) compared to that which occurs in the excited state of the conformers of **2a** (from 1.71/2–1.81 Å or more) suggesting that the photolysis of **3a** should be a difficult process. Quite the opposite, as demonstrated in Scheme 2, protocols used for alkyl(aryl)‐xanthates were successfully applied to *closo*‐carboranyl‐xanthates **3a** and **3f,** allowing several sulfur containing *closo*‐carboranes, as key intermediates for further derivatizations, to be obtained.

Treatment with ethylenediamine (EDA) followed by acid quench allowed **3a** to be converted to the thiol group in good yield (**4a**, 66%). Notably, this transformation provides a convenient and odorless access to B(9)‐mercapto carboranes, in contrast to known traditional methods, which are based on the use of AlCl_3_ and S_8_ or S_2_Cl_2_ at high temperatures, showing low tolerance with several functional groups [82, 83]. Additionally, the in situ‐generated thiolate readily underwent acylation to furnish the thioester moiety (**4b**, 63%), as well as open‐air oxidation to yield the disulfide (**4c**, 55%). Furthermore, with the employment of nickel catalysis, the xanthate moiety underwent efficient cross‐coupling with iodobenzene, affording the *S*‐aryl thioether (**4d**, 53%) in good yield. *S*‐aliphatic thioethers were also conveniently obtained by quenching with the appropriate alkyl iodides, providing compounds **4e** in 59% yield, and the carborane analogue of the pesticide Chlorbenside (**4f**, 76%). Chlorination of **3a** with *N*‐chlorosuccinimide (NCS), followed by acid treatment, enabled the synthesis of B(9)‐sulfonyl chloride (**4g**, 51%). Finally, CsF‐promoted deboronation of the *closo* cluster **3f** furnished the corresponding B(9)‐xanthyl‐*nido*‐carborane (**4h**) in a modest 27% yield, possibly due to degradation of the xanthyl group in basic conditions under reflux.

### 2.1 Mechanistic Studies

**FIGURE 2:**
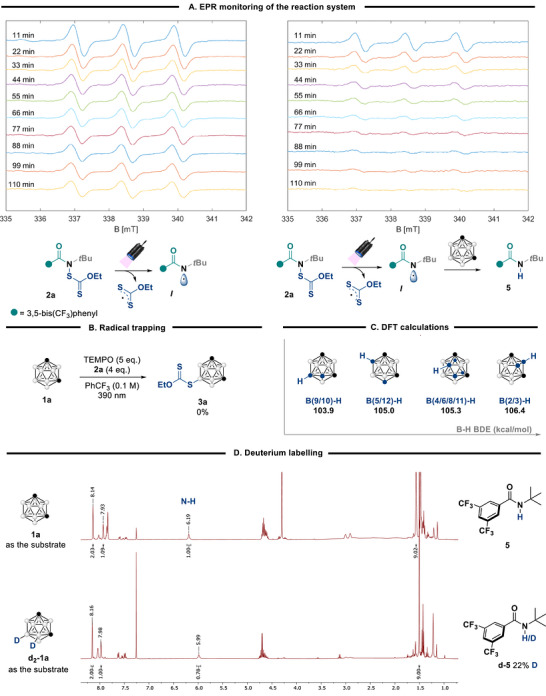
Mechanistic study. (A) EPR monitoring of NCR photogeneration from **2a** (left); quenching of the NCR signal by *m*‐carborane (right). (B) Radical trapping experiment using TEMPO. (C) Calculated B─H BDEs. (D) Deuterium labeling experiment: comparison of the integration area of the amide **5** N─H peak in ^1^H‐NMR when **1a** (top) and **d_2_‐1a** (bottom) are respectively used as the starting materials. The area decreases from 1 to 0.78 with **d_2_‐1a**.

To gain a comprehensive understanding of the observed reactivity, mechanistic studies, both experimentally and computationally were carried out. EPR spectroscopy was employed to investigate the reaction between xanthyl amide **2a** and *m*‐carborane **1a**. To this end, the intensity of the photo‐generated NCR in a solution of **2a** alone (Figure 2A, left), and in a solution of **2a** in the presence of *m*‐carborane **1a** at room temperature (Figure 2A, right), as a function of irradiation time was measured. In the absence of **1a**, a persistent EPR signal pertinent to the NCR **I**, generated by irradiation of **2a**, is observed. Whereas, the intensities of the EPR signal show a reduced concentration of *N*‐amidyl radical **I** at each time point and a faster decay in the presence of *m*‐carborane **1a** (Figure 2A, right). These observations are consistent with a photoinduced homolytic splitting of the N─S bond of xanthyl amide **2a** to yield *N*‐amidyl radical **I**, which subsequently promotes the hydrogen abstraction from *m*‐carborane. The latter eventually reacts with the xanthyl radical to form the final product **5**. Although no direct observation of either the xanthyl‐ or the carboranyl‐radicals was achieved under experimental conditions, EPR confirms the HAT process from NCR **I** to **1a**, promoted by light irradiation. Additionally, to probe the involvement of radical intermediates, a control experiment was conducted. Introducing the radical scavenger TEMPO to the reaction mixture resulted in the complete suppression of the desired reactivity (Figure 2B). However, no notable TEMPO‐derived adducts were recovered, reasonably due to the short lifetimes of the B‐centered carboranyl radicals.

Furthermore, to establish the involvement of an NCR‐mediated HAT process at the B(9)─H vertex, a deuteration labeling experiment was performed (Figure 2D). Deuterated d_2_‐*m*‐carborane (**d_2_‐1a**) was subjected to the optimized reaction conditions, the outcome was compared to that obtained with **1a**. ^1^H‐ and ^2^H‐NMR analysis of the crude reaction mixtures confirmed formation of amide **5**, consistently with a HAT‐enabled mechanism. In particular, the integration of the signal at ∼6 ppm, pertinent to the N─H of **5**, was evaluated, resulting in a reduced value of 0.78 when employing **d_2_‐1a** versus 1.00 in the case of **1a**, thus confirming the D incorporation in **5**, and supporting site‐selectivity at the B(9)‐vertex. Complementary DFT calculations supported these findings by mapping the B─H BDEs of the cage vertices, identifying the B(9/10) vertices as the most thermodynamically favored positions for the HAT event (Figure 2C).

**FIGURE 3:**
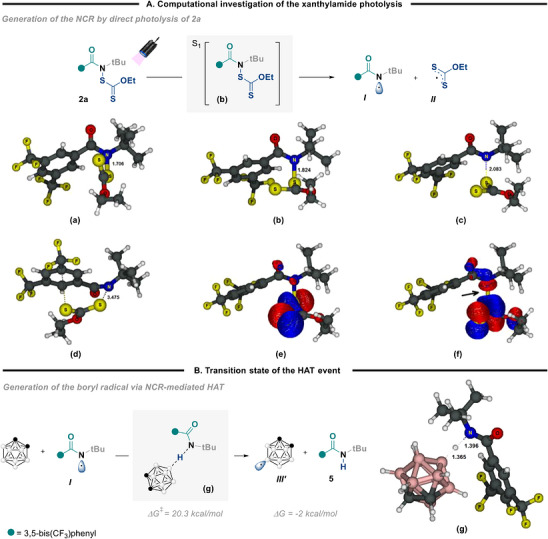
(A) The photolysis of the most stable conformer of **2a**. (a) the conformer **2a‐1**; (b) its first singlet excited state (S_1_)2a‐1*; (c) the transition structure for the N─S bond breaking from (S_1_)2a‐1*; (d) the radical pair formed by the NCR and the xanthyl radical; (e) the HOMO in (S_1_)2a‐1*; (f) the LUMO in (S_1_)2a‐1*. (B) The transition structure for the HAT at the B(9,12)–H site of *o*‐carborane by the NCR.

The photolysis of **2a** was also studied by computational method (see ESI). After identifying seven conformations of **2a**, (**2a‐1 – 2a‐7**), the structures of the first singlet excited state of the four most stable conformers were optimized. In all cases an elongation of the N─S bond was observed which is due to the nature of the electronic transition. This is mainly described as a HOMO–LUMO excitation. In the latter, the antibonding interaction (indicated by the arrow in Figure 3‐A(f)), is responsible for the elongation of the N─S bond and of its easy breaking. Those from three conformers (**2a‐1**, **2a‐2**, and **2a‐4**) lead to stable structures from which the relative transition structures for the dissociation to the radical pair formed by the NCR and the xanthyl radical were optimized. The activation energies are all around 1 kcal/mol. The one originating from the conformer **2a‐3** spontaneously breaks in the radical pair, which is thermodynamically more stable than the excited states. Moreover, its dissociation in the two free radicals is also thermodynamically favored with respect to the corresponding excited states. These results confirm that, after light absorption, the photolysis of **2a** is a feasible process. Finally, the HAT at the B(9,12)─H site of *o*‐carborane by the NCR was studied, finding the quite low enthalpy barrier of 6.4 and 20.2 kcal/mol in terms of free energy (ESI, Section S10) (Figure 3‐B and ESI, Section S3.3.4). This reaction, that generates the B(9)–centered radical *III’* and the *N‐t*‐butyl‐(3,5‐di‐trifluoromethyl)benzamide **5**, is slightly thermodynamically favored (*∆H* = −1.7 kcal/mol, *∆G* = −2.0 kcal/mol). The forthcoming radical coupling between ***III’*** and the xanthyl radical takes place without any energy barrier because extremely exoergic (*∆H* = −77.8 kcal/mol, *∆G* = −66.0 kcal/mol).

**SCHEME 3:**
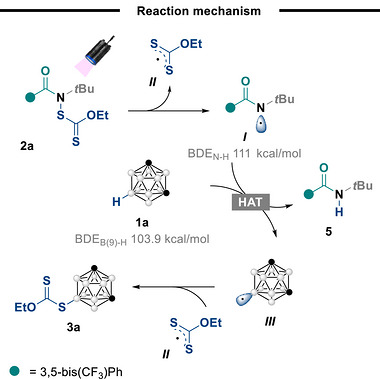
Mechanism of the photoinduced B(9)─H xanthylation reaction

Supported by the comprehensive mechanistic investigation, a reaction mechanism for the xanthylation at the B(9)─H vertex of **1a** is proposed (Scheme 3). Xanthylamide **2a** readily undergoes homolysis of the N─S bond upon irradiation, generating the NCR **I** (N─H BDE = 111 kcal/mol) and the xanthyl radical **II**. Subsequent HAT from **I** to the weakest B─H vertex of **1a** (B(9)─H BDE = 103.9 kcal) affords the corresponding B‐centered radical **III**, which rapidly traps by radical coupling the previously generated xanthyl radical **II** to yield B(9)‐xanthyl‐*m*‐carborane **3a** as the final product.

## 3 Conclusions

In summary, the synthesis of boryl xanthates by the regioselective activation of the B(9)─H bond in *closo*‐carboranes is here reported for the first time. Experimental and computational mechanistic studies demonstrate the feasibility of the photolysis of a properly designed xanthylamide **2a** by visible light to generate the NCR, and EPR studies on the irradiated xanthylamide solution, with and without carborane **1a**, corroborate the HAT‐mediated selective generation of a B(9)‐centered radical as the key intermediate of the process. The so‐formed boron centered radicals readily react with the xanthyl group affording several xanthylated substituted *o*‐ and‐ *m*‐*closo*‐carboranes. While NCR‐mediated HAT was previously demonstrated, the present work reveals an unprecedented radical trapping manifold enabling access to boryl xanthates, which unlock orthogonal sulfur‐based diversification pathways not achievable in prior studies. The preparation of different organosulfur derivatives is then accomplished, thus demonstrating the role of these xanthylated boron derivatives as bench‐stable versatile platforms for the selective B‐functionalization in carborane clusters. Thiol, thioether, disulfide, thioester, and halosulfonyl groups have been successfully introduced. The reaction has been scaled up to 1 mmol, and a carboranyl analogue of the pesticide Chlorbenside has been prepared, demonstrating the synthetic value of this protocol.

## Author Contributions

**Marco Rusconi**: investigation, writing – review and editing, data curation. **Eugenia Magi**: investigation. **Polyssena Renzi**: investigation, writing – review and editing, methodology. **Emanuele Azzi**: writing – review and editing, investigation. **Valeria Lagostina**: investigation, validation. **Enrico Salvadori**: conceptualization, investigation, data curation. **Mario Chiesa**: conceptualization, data curation, validation. **Birgit Hischa**: investigation, data curation. **Giovanni Ghigo**: conceptualization, investigation, writing – review and editing, data curation. **Annamaria Deagostino**: conceptualization, supervision, resources, funding acquisition, writing – original draft.

## Conflicts of Interest

The authors declare no conflicts of interest.

## Supporting information




**Supporting File 1**: anie72341‐sup‐0001‐SuppMat.pdf.

**Supporting File 2**: anie72341‐sup‐0002‐Data.zip.

## Data Availability

The data that support the findings of this study are available from the corresponding author upon reasonable request.

## References

[1] R. N. Grimes , Carboranes, (Academic Press, 2016): 1041.

[2] F. Lovering , J. Bikker , and C. Humblet , “Escape From Flatland: Increasing Saturation as an Approach to Improving Clinical Success,” Journal of Medicinal Chemistry 52 (2009): 6752–6756, 10.1021/jm901241e. 19827778

[3] F. Lovering , “Escape From Flatland 2: Complexity and Promiscuity,” Medicinal Chemistry Communications 4 (2013): 515–519, 10.1039/c2md20347b.

[4] M. Scholz and E. Hey‐Hawkins , “Carbaboranes as Pharmacophores: Properties, Synthesis, and Application Strategies,” Chemical Reviews 111 (2011): 7035–7062, 10.1021/cr200038x. 21780840

[5] F. Issa , M. Kassiou , and L. M. Rendina , “Boron in Drug Discovery: Carboranes as Unique Pharmacophores in Biologically Active Compounds,” Chemical Reviews 111 (2011): 5701–5722, 10.1021/cr2000866. 21718011

[6] A. Marfavi , P. Kavianpour , and L. M. Rendina , “Carboranes in Drug Discovery, Chemical Biology and Molecular Imaging,” Nature Reviews Chemistry 6 (2022): 486–504, 10.1038/s41570-022-00400-x. 37117309

[7] E. Hey‐Hawkins and C. Viñas Teixidor , Boron‐Based Compounds: Potential and Emerging Applications in Medicine (John Wiley & Sons Ltd, 2018), 470, 10.1002/9781119275602.

[8] A. H. Soloway , W. Tjarks , B. A. Barnum , et al., “The Chemistry of Neutron Capture Therapy,” Medicinal Chemistry Communications 98 (1998): 1515–1562, 10.1021/cr941195u. 11848941

[9] K. Hu , Z. M. Yang , L. L. Zhang , et al., Coordination Chemistry Reviews 405 (2020): 213139.

[10] M. F. Hawthorne and M. W. Lee , “A Critical Assessment of Boron Target Compounds for Boron Neutron Capture Therapy,” Journal of Neuro–Oncology 62 (2003): 33–45, 10.1007/BF02699932. 12749701

[11] P. J. Kueffer , C. A. Maitz , A. A. Khan , et al., “Boron Neutron Capture Therapy Demonstrated in Mice Bearing EMT6 Tumors Following Selective Delivery of Boron by Rationally Designed Liposomes,” Proceedings National Academy of Science USA 110 (2013): 6512–6517, 10.1073/pnas.1303437110. PMC3631690 23536304

[12] K. Shelly , D. A. Feakes , M. F. Hawthorne , P. G. Schmidt , T. A. Krisch , and W. F. Bauer , “Model Studies Directed Toward the Boron Neutron‐capture Therapy of Cancer: Boron Delivery to Murine Tumors With Liposomes,” Proceedings National Academy of Science USA 89 (1992): 9039–9043, 10.1073/pnas.89.19.9039. PMC50060 1409600

[13] S. P. Fisher , A. W. Tomich , S. O. Lovera , et al., “Nonclassical Applications of *closo*‐Carborane Anions: From Main Group Chemistry and Catalysis to Energy Storage,” Chemical Reviews 119 (2019): 8262–8290, 10.1021/acs.chemrev.8b00551. 30707011

[14] X. Yu , J. Xu , M. Wang , et al., “A Hot Exciton Luminogen Constructed by an *o*‐Carborane Scaffold,” Advanced Optical Materials 12 (2024): 2302218, 10.1002/adom.202302218.

[15] J. F. Valliant , K. J. Guenther , A. S. King , et al., “The Medicinal Chemistry of Carboranes,” Coordination Chemistry Reviews 232 (2002): 173–230, 10.1016/S0010-8545(02)00087-5.

[16] V. I. Bregadze , “Fifty Years of Carborane Chemistry: The History of Discovery and the First Results,” Russian Chemical Bulletin 63 (2014): 1021–1026, 10.1007/s11172-014-0543-5.

[17] K.‐X. Yan , R.‐X. Yan , Z.‐W. Wang , D. Tu , C.‐S. Lu , and H. Yan , “Boron Cluster Functionalization: From Vertex‐Specific Modifications to Boron Cage Extension,” Chemistry of European Journal 31 (2025): e01847, 10.1002/chem.202501847. 40702825

[18] L. I. Zakharkin , V. N. Kalinin , and V. V. Gedymin , “Synthesis and Some Reactions of 3‐amino‐*o*‐carboranes,” Journal of Organometallic Chemistry 16 (1969): 371–379, 10.1016/S0022-328X(00)89762-4.

[19] H. A. Mills , F. Alsarhan , T.‐C. Ong , M. Gembicky , A. L. Rheingold , and A. M. Spokoyny , “Icosahedral *m*‐Carboranes Containing Exopolyhedral B–Se and B–Te Bonds,” Inorganic Chemistry 60 (2021): 19165–19174, 10.1021/acs.inorgchem.1c02981. 34855370 PMC8761389

[20] W. Jiang , C. B. Knobler , C. E. Curtis , M. D. Mortimer , and M. F. Hawthorne , “Iodination Reactions of Icosahedral Para‐Carborane and the Synthesis of Carborane Derivatives With Boron‐Carbon Bonds,” Inorganic Chemistry 34 (1995): 3491–3498, 10.1021/ic00117a018.

[21] Z. Qiu and Z. Xie , “A Strategy for Selective Catalytic B–H Functionalization of *o*‐Carboranes,” Accounts of Chemical Research 54 (2021): 4065–4079, 10.1021/acs.accounts.1c00460. 34693715

[22] C. Tang , J. Zhang , and Z. Xie , “Direct Nucleophilic Substitution Reaction of Cage B−H Bonds by Grignard Reagents: A Route to Regioselective B4‐Alkylation of *o*‐Carboranes,” Angewandte Chemie International Edition 56 (2017): 8642–8646, 10.1002/anie.201702347. 28544370

[23] C. Tang , J. Zhang , J. Zhang , and Z. Xie , “Regioselective Nucleophilic Alkylation/Arylation of B–H Bonds in *o*‐Carboranes: An Alternative Method for Selective Cage Boron Functionalization,” Journal of the American Chemical Society 140 (2018): 16423–16427, 10.1021/jacs.8b10270. 30441890

[24] Y. Wang , Y. Gao , W. Guo , Q. Zhao , Y.‐N. Ma , and X. Chen , “Highly Selective Electrophilic B(9)‐amination of *o*‐carborane Driven by HOTf and HFIP,” Organic Chemistry Frontiers 9 (2022): 4975–4980, 10.1039/D2QO00732K.

[25] W. Guo , C. Guo , Y.‐N. Ma , and X. Chen , “Practical Synthesis of B(9)‐Halogenated Carboranes With N‐Haloamides in Hexafluoroisopropanol,” Inorganic Chemistry 61 (2022): 5326–5334, 10.1021/acs.inorgchem.2c00074. 35311288

[26] Y.‐G. Li and Y.‐N. Ma , “Selective B(9)–H Bond Oxidation of *o*/*m*‐Carboranes With M CPBA,” Inorganic Chemistry 64 (2025): 20997–21003, 10.1021/acs.inorgchem.5c03028. 41070550

[27] Y.‐N. Ma , H. Ren , Y. Wu , N. Li , F. Chen , and X. Chen , “B(9)‐OH‐*o*‐Carboranes: Synthesis, Mechanism, and Property Exploration,” Journal of the American Chemical Society 145 (2023): 7331–7342, 10.1021/jacs.2c13570. 36962083

[28] W. Lu , Y. Wu , Y.‐N. Ma , F. Chen , and X. Chen , “A Method for Highly Selective Halogenation of *o*‐Carboranes and *m*‐Carboranes,” Inorganic Chemistry 62 (2023): 885–892, 10.1021/acs.inorgchem.2c03694. 36584667

[29] Y.‐N. Ma , Y. Gao , Y. Ma , Y. Wang , H. Ren , and X. Chen , “Palladium‐Catalyzed Regioselective B(9)‐Amination of *o*‐Carboranes and *m*‐Carboranes in HFIP With Broad Nitrogen Sources,” Journal of the American Chemical Society 144 (2022): 8371–8378, 10.1021/jacs.2c03031. 35499359

[30] Y. Wang , Y.‐G. Li , F. Chen , Y.‐N. Ma , and X. Chen , “HSAB Theory Guiding Electrophilic Substitution Reactions of *o*‐carborane,” Organic Chemistry Frontiers 12 (2025): 76–84, 10.1039/D4QO01546K.

[31] J. Zhang and Z. Xie , “A Strategy for Regioselective B–H Functionalization of *o*‐carboranes via Base Metal Catalysis,” Organic Chemistry Frontiers 10 (2023): 3074–3079, 10.1039/D3QO00621B.

[32] J. Zhang and Z. Xie , “Directing‐Group‐Assisted Transition‐Metal‐Catalyzed Selective BH Functionalization of *o*‐Carboranes.”Synthesis 57 (2024): 495–521.

[33] Y. J. Quan and Z. W. Xie , “Controlled Functionalization of *o*‐carborane via Transition Metal Catalyzed B–H Activation,” Chemical Society Reviews 48 (2019): 3660–3673, 10.1039/C9CS00169G. 31090766

[34] Y. K. Au , J. Zhang , Y. Quan , and Z. Xie , “Ir‐Catalyzed Selective B(3)‐H Amination of *o*‐Carboranes With NH_3_ ,” Journal of the American Chemical Society 143 (2021): 4148–4153, 10.1021/jacs.1c00593. 33719434

[35] Y. Chen , Y. Quan , and Z. Xie , “Ir‐catalyzed Selective Dehydrogenative Cross‐coupling of Aryls With *o*‐carboranes via a Mixed Directing‐group Strategy,” Chemical Communications 56 (2020): 7001–7004, 10.1039/D0CC02531C. 32441711

[36] R. Cheng , J. Zhang , H. Zhang , Z. Qiu , and Z. Xie , “Ir‐catalyzed Enantioselective B−H Alkenylation for Asymmetric Synthesis of Chiral‐at‐cage *o*‑Carboranes,” Nature Communications 12 (2021): 7146, 10.1038/s41467-021-27441-y. PMC8654863 34880231

[37] L.‐B. Zhang and Z. Xie , “Iridium‐Catalyzed Selective B(4)–H Acylmethylation of *o*‐Carboranes With Sulfoxonium Ylides,” Organic Letters 24 (2022): 1318–1322, 10.1021/acs.orglett.1c04335. 35129366

[38] C.‐Y. Zhang , K. Cao , D. Liu , et al., “Iridium‐catalyzed Selective Amination of B(4)–H for the Synthesis of *o*‐carborane‐fused Indolines,” Dalton Transactions 52 (2023): 2933–2936, 10.1039/D3DT00316G. 36815456

[39] Y. K. Au , Q. Ma , J. Zhang , and Z. Xie , “Chemistry ‐ An Asian Journal 18 (2023): E202300611,” Chemistry ‐ An Asian Journal 18 (2023): e202300611. 37694997 10.1002/asia.202300611

[40] H.‐B. Yang , Y. Guo , K. Cao , et al., “Iridium‐catalyzed Selective Arylation of B(6)–H of 3‐aryl‐ *o*‐carboranes With Arylboronic Acid via Direct B–H Activation,” Chemical Communications 60 (2024): 1124–1127, 10.1039/D3CC05630A. 38193475

[41] K. Lee , J. Kim , D. Hwang , et al., “Iridium(III)‐catalyzed Remote B(9)−H Alkylation of *o*‐carboranes With Nitrile Template,” Nature Communications 16 (2025): 10628, 10.1038/s41467-025-65616-z. PMC12660857 41309547

[42] K. Cao , J. Wu , C.‐Y. Zhang , L.‐F. Ding , and J. Yang , “Palladium Catalyzed Selective Amination of B (7)‐H for Synthesis of *o*‐Carborane Fused Isoquinolinones,” Journal of Organometallic Chemistry 954 (2021): 122069.

[43] Y. Ge , J. Zhang , Z. Qiu , and Z. Xie , “Pd‐Catalyzed Sequential B(3)–I/B(4)–H Bond Activation for the Synthesis of 3,4‐benzo‐ *o*‐carboranes,” Dalton Transactions 50 (2021): 1766–1773, 10.1039/D0DT03740K. 33459736

[44] C.‐C. Teng , Y. Guo , K. Cao , et al., “Palladium Catalyzed Selective Arylation of B(4)‐H of *o*‐Carboranes With Potassium Aryltrifluoroborate,” Journal of Organometallic Chemistry 1008 (2024): 123051.

[45] H.‐T. Zhang , Y. Gao , Y.‐N. Ma , and X. Chen , “Pd(ii)‐Catalyzed B(9)‐Alkynylation of *o*/*m*‐Carboranes,” Organic Chemistry Frontiers 11 (2024): 6706–6711, 10.1039/D4QO01428F.

[46] J.‐R. Chang , H.‐J. Cao , Y.‐N. Ma , and X. Chen , “Palladium‐Catalyzed Cross‐Coupling Reactions of Carboranes With Alkenes via Selective B–H Bond Activation,” Organic Letters 27 (2025): 1858–1863, 10.1021/acs.orglett.5c00059. 39960023

[47] S. Zhu , Y. Liu , and Z. Xie , “Palladium‐Catalyzed Selective B(3)‐Esterification of *o*‐Carboranes With CO and Alcohols,” Organic Letters 27 (2025): 2429–2432, 10.1021/acs.orglett.5c00308. 40026134

[48] P. Zhou , Y. Chen , and Z. Xie , “Iron‐Catalyzed Selective B–H Activation for 4/5‐fold Methylation and Arylation of Carboranes,” ACS Catalysis 12 (2022): 8761–8767, 10.1021/acscatal.2c02120.

[49] J. Zhao and Z. Xie , “Nickel‐catalyzed Regioselective B(3,4,5,6)‐H Tetra‐alkylation of *o*‐Carboranes,” Science China Chemistry 66 (2023): 2836–2841, 10.1007/s11426-023-1750-0.

[50] H.‐J. Cao , J.‐X. Li , J.‐H. Yan , et al., “Post‐coordination of Ru(ii) Controlled Regioselective B(4)–H Acylmethylation of *o*‐carboranes With Sulfoxonium Ylides,” Chemical Science 16 (2025): 9406–9412, 10.1039/D5SC01576F. 40308949 PMC12038429

[51] H. A. Mills , J. L. Martin , A. L. Rheingold , and A. M. Spokoyny , “Oxidative Generation of Boron‐Centered Radicals in Carboranes,” Journal of the American Chemical Society 142 (2020): 4586–4591, 10.1021/jacs.0c00300. 32073842 PMC7276281

[52] S. Li , J. Zhang , and Z. Xie , “Visible‐Light‐Induced Palladium‐Catalyzed Cross‐Coupling of Iodocarboranes With (Hetero)Arenes,” Organic Letters 24 (2022): 7497–7501, 10.1021/acs.orglett.2c02648. 36201284

[53] S. Li and Z. Xie , “Visible‐Light‐Promoted Nickel‐Catalyzed Cross‐Coupling of Iodocarboranes With (Hetero)Arenes via Boron‐Centered Carboranyl Radicals,” Journal of the American Chemical Society 144 (2022): 7960–7965, 10.1021/jacs.2c02329. 35451827

[54] D. Zhao and Z. Xie , “Visible‐Light‐Promoted Photocatalytic B−C Coupling via a Boron‐Centered Carboranyl Radical: Facile Synthesis of B(3)‐Arylated *o*‐Carboranes,” Angewandte Chemie (International ed in English) 55 (2016): 3166–3170, 10.1002/anie.201511251. 26822125

[55] M. Chen , J. Xu , D. Zhao , et al., “Site‐Selective Functionalization of Carboranes at the Electron‐Rich Boron Vertex: Photocatalytic B−C Coupling via a Carboranyl Cage Radical,” Angewandte Chemie (International ed in English) 61 (2022): e202205672, 10.1002/anie.202205672. 35670361

[56] F. Paulus , C. Heusel , M. Jaspers , et al., “ *closo*‐Carboranyl Analogs of β‐Arylethylamines: Direct Synthesis From Alkenes via EnT‐Catalysis,” Angewandte Chemie International Edition 64 (2025): e202504793, 10.1002/anie.202504793. 40274532 PMC12207362

[57] H. Ren , P. Zhang , J. Xu , et al., “Direct B–H Functionalization of Icosahedral Carboranes via Hydrogen Atom Transfer,” Journal of the American Chemical Society 145 (2023): 7638–7647, 10.1021/jacs.3c01314. 36946888

[58] H. Ren , N. Zhou , W. Ma , D. Tu , C.‐S. Lu , and H. Yan , “Direct B–H Activation of Carborane Clusters via Synergistic LMCT and HAT Photocatalysis,” Journal of the American Chemical Society 148 (2026): 4680–4693, 10.1021/jacs.5c20945. 41570202

[59] A. Lanfranco , S. Rakhshan , D. Alberti , et al., “Combining BNCT With Carbonic Anhydrase Inhibition for Mesothelioma Treatment: Synthesis, In Vitro, In Vivo Studies of Ureidosulfamido Carboranes,” European Journal of Medicinal Chemistry 270 (2024): 116334, 10.1016/j.ejmech.2024.116334. 38552427

[60] J. Sforzi , A. Lanfranco , R. Stefania , et al., “A Novel pH Sensitive Theranostic PLGA Nanoparticle for Boron Neutron Capture Therapy in Mesothelioma Treatment,” Scientific Reports 13 (2023): 620, 10.1038/s41598-023-27625-0. 36635364 PMC9837127

[61] D. Alberti , A. Deagostino , A. Toppino , et al., “An Innovative Therapeutic Approach for Malignant Mesothelioma Treatment Based on the Use of Gd/Boron Multimodal Probes for MRI Guided BNCT,” Journal of Controlled Release 280 (2018): 31–38, 10.1016/j.jconrel.2018.04.043. 29730155

[62] S. Rakhshan , A. Lanfranco , D. Alberti , et al., “A Comparative Study of ^157^Gd and ^10^B Effect in a Carborane‐Based Theranostic Agent for Membrane‐Targeted Carbonic Anhydrase IX Inhibition and MRI‐Guided Neutron Capture Therapy in Mesothelioma Treatment,” ACS Central Science 11 (2025): 2215–2229, 10.1021/acscentsci.5c01632. 41341048 PMC12670288

[63] V. A. Schmidt , R. K. Quinn , A. T. Brusoe , and E. J. Alexanian , “Site‐Selective Aliphatic C–H Bromination Using N‐Bromoamides and Visible Light,” Journal of the American Chemical Society 136 (2014): 14389–14392, 10.1021/ja508469u. 25232995

[64] A. D. Handlin , F. A. Leibfarth , and E. J. Alexanian , “Divergent Concerted Proton–Electron Transfer (CPET) and Hydrogen Atom Transfer (HAT) Pathways of Amidyl Radical Reactivity Enable Chemoselective Functionalization,” Organic Letters 27 (2025): 12018–12023, 10.1021/acs.orglett.5c03705. 41100840

[65] C. Pratley , S. Fenner , and J. A. Murphy , “Nitrogen‐Centered Radicals in Functionalization of sp^2^ Systems: Generation, Reactivity, and Applications in Synthesis,” Chemical Reviews 122 (2022): 8181–8260, 10.1021/acs.chemrev.1c00831. 35285636

[66] M. M. Tierney , S. Crespi , D. Ravelli , and E. J. Alexanian , “Identifying Amidyl Radicals for Intermolecular C–H Functionalizations,” Journal of Organic Chemistry 84 (2019): 12983–12991, 10.1021/acs.joc.9b01774. 31441300 PMC6834340

[67] A. M. Carestia , D. Ravelli , and E. J. Alexanian , “Reagent‐dictated Site Selectivity in Intermolecular Aliphatic C–H Functionalizations Using Nitrogen‐centered Radicals,” Chemical Science 9 (2018): 5360–5365, 10.1039/C8SC01756E. 30009007 PMC6009468

[68] E. C. Gentry and R. R. Knowles , “Synthetic Applications of Proton‐Coupled Electron Transfer,” Accounts of Chemical Research 49 (2016): 1546–1556, 10.1021/acs.accounts.6b00272. 27472068 PMC5102158

[69] G. Kumar , S. Pradhan , and I. Chatterjee , “N‐Centered Radical Directed Remote C−H Bond Functionalization via Hydrogen Atom Transfer,” Chemistry ‐ An Asian Journal 15 (2020): 651–672, 10.1002/asia.201901744. 32011095

[70] A. Solovyev , S. H. Ueng , J. Monot , et al., “Estimated Rate Constants for Hydrogen Abstraction From N‐Heterocyclic Carbene−Borane Complexes by an Alkyl Radical,” Organic Letters 12 (2010): 2998–3001, 10.1021/ol101014q. 20536158

[71] A. L. Vallet , S. Telitel , J. Lalevée , and E. Lacôte , “Reactivity of B‐Xanthyl N‐Heterocyclic Carbene‐Boranes,” Helvetica Chimica Acta 102 (2019): e1900198.

[72] S. Sumino , T. Fukuyama , M. Sasano , et al., “Vicinal Difunctionalization of Alkenes by Four‐component Radical Cascade Reaction of Xanthogenates, Alkenes, CO, and Sulfonyl Oxime Ethers,” Beilstein Journal of Organic Chemistry 15 (2019): 1822–1828, 10.3762/bjoc.15.176. 31467602 PMC6693370

[73] B. Quiclet‐Sire and S. Z. Zard , “Xanthates and Vinyl Esters, a Remarkably Powerful Alliance,” Heterocycles 99 (2019): 742–765.

[74] S. Z. Zard , “The Xanthate Route to Ketones: When the Radical Is Better Than the Enolate,” Accounts of Chemical Research 51 (2018): 1722–1733, 10.1021/acs.accounts.8b00201. 29932322

[75] L. Forezi , R. K. F. Marra , F. D. da Silva , and V. F. Ferreira , “Synthetic Strategies for Obtaining Xanthenes,” Current Organic Synthesis 14 (2017): 929–951.

[76] S. Z. Zard , “The Xanthate Route to Organofluorine Derivatives. A Brief Account,” Organic & Biomolecular Chemistry 14 (2016): 6891–6912, 10.1039/C6OB01087C. 27327241

[77] L. Wang , W.‐Y. Zhou , S.‐C. Chen , M.‐Y. He , and Q. Chen , “An Efficient Copper‐Catalyzed One‐Pot Synthesis of Diaryl Thioethers by Coupling of Arylboronic Acids With Potassium Ethyl Xanthogenate Under Mild Conditions,” Synlett 2011 (2011): 3041–3045, 10.1055/s-0031-1289874.

[78] S. Z. Zard , “The Xanthate Route to Indolines, Indoles, and Their Aza Congeners,” Chemistry of European Journal 26 (2020): 12689–12705, 10.1002/chem.202001341. 32314826

[79] L. Tai , L. Chen , Y. Shi , and L.‐A. Chen , “Redox‐active Alkyl Xanthate Esters Enable Practical C–S Cross‐coupling by Nickel Catalysis,” Organic Chemistry Frontiers 10 (2023): 2505–2516, 10.1039/D3QO00136A.

[80] J. J. Monteith and S. A. L. Rousseaux , “Redox‐Active Thiocarbonyl Auxiliaries in Ni‐Catalyzed Cross‐Couplings of Aliphatic Alcohols,” Accounts of Chemical Research 56 (2023): 3581–3594, 10.1021/acs.accounts.3c00541. 38047525

[81] C. G. Na , D. Ravelli , and E. J. Alexanian , “Direct Decarboxylative Functionalization of Carboxylic Acids via O–H Hydrogen Atom Transfer,” Journal of the American Chemical Society 142 (2020): 44–49, 10.1021/jacs.9b10825. 31877036 PMC7147874

[82] L. I. Zakharkin and I. V. Pisareva , “A New Simple Method for the Production and Some Conversions of B[sbnd]S Bond Containing *o*‐ and *m*‐carboranyl,” Phosphorus Sulfur and Related Elements 20 (1984): 357–370, 10.1080/03086648408077645.

[83] D. Wang , Z. He , C. Liu , et al., “Synthesis of Carboranyl Alkyl Sulfide via Radical Thiol–Ene “Click” Reaction,” Organic Letters 27 (2025): 4000–4005, 10.1021/acs.orglett.5c00959. 40192119

