# Supplementary material for: Site‐Selective B─H Activation via HAT Toward Xanthyl‐closo‐Carboranes as Bench‐Stable Precursors of Organosulfur Boron Clusters
Source: Angew Chem Int Ed Engl. 2026 Apr 22;65(24):e7686382. doi: 10.1002/anie.7686382 (PMC13245599; doi:10.1002/anie.7686382)

Supporting Information and Experimental Procedures

Site-Selective B-H Activation via HAT towards Xanthyl-closo-Carboranes as Bench-Stable Precursors of Organosulfur Boron Clusters

Marco Rusconi,[a] Eugenia Magi,[a] Polyssena Renzi,[a] Emanuele Azzi,[a] Valeria Lagostina,[a] Enrico Salvadori,[a] Mario Chiesa,[a] Birgit Hischa,[b] Giovanni Ghigo,[a] Annamaria Deagostino[a*]

[a]Department of Chemistry, University of Torino, via P. Giuria 7, 10125, Torino, Italy

[b]Department of X-ray Structure Analysis, University of Regensburg, Universitäetsstr. 31D-93053 Regensburg, Germany

Table of contents

S1. Materials and methods 3

S2. Optimisation of reaction conditions 4

S3. Mechanistic study 6

S3.1. Deuterium Labelling 6

S3.2. EPR Spectroscopy 9

S3.3. Computational method with references 10

S3.3.1. Computational method with references 10

S4. Experimental procedures 22

S4.1. Synthesis of 2e 22

S4.2. Synthesis of 2d 22

S4.3. Synthesis of 2c 23

S4.4. Synthesis of 2b 24

S4.5. Synthesis of 2a 25

S4.6. General procedure for the synthesis of carboranes 1c-j 27

S4.7. Synthesis of carborane 1l 31

S4.8. Synthesis of carboranes 1n-o 31

S4.9. General procedure for the synthesis of xanthyl carboranes 3a-o 32

S4.10. Synthetic applications 37

S4.10.1. Synthesis of 4a 37

S4.10.2. Synthesis of 4b 37

S4.10.3. Synthesis of 4c 38

S4.10.4. Synthesis of 4d 38

S4.10.5. Synthesis of 4e 39

S4.10.6. Synthesis of 4f 39

S4.10.7. Synthesis of 4g 40

S4.10.8. Synthesis of 4h 40

S5. Crystal data 41

S6. References 47

S7. NMR Spectra 49

S1. Materials and methods

Flasks and all equipment employed for moisture-sensitive reactions and compounds were dried by electric heat gun under N_2. Analytical grade solvents were used as received. All commercially available reagents were used as received. Products were purified by preparative column chromatography on Sigma-Aldrich silica-gel for flash chromatography, 0.04 0.063 mm/230-400 mesh. Reactions were monitored by TLC using silica-gel on TLC-PET foils Sigma Aldrich, 2.25 μm, layer thickness 0.2 mm, medium pore diameter. Carboranes and their derivatives were visualised on TLC plates using a 5% $PdCl_2$ aqueous solution in HCl.
Photochemical reactions were carried out in a 4 ml scintillation flask. A Kessil Purple LED (390 nm) was used as the irradiation source. The irradiation source was located at 4 cm from the walls of the vessel.
NMR spectra were recorded employing a Bruker Avance Neo 400 and Jeol ECZR 600 MHz. ^{1}H NMR spectra were recorded in $CDCl_3$ at 400 MHz or 600 MHz. $^{13}C\{^{1}H\}$-NMR spectra were recorded in $CDCl_3$ 100 MHz or 150 MHz. $^{11}B\{^{1}H\}$-NMR spectra were recorded in $CDCl_3$ 128 MHz.. DEPT experiments were carried out with a DEPT-135 sequence. ^{1}H NMR coupling constants (J) were reported in Hertz (Hz) and multiplicities are indicated as follows s (singlet), d (doublet), t (triplet), q (quarte), m (multiplet), bs (broad singlet). Structural assignments were made with additional information from gCOSY and HSQC experiments.
HRMS spectra were obtained on a Orbitrap IQ-X high resolution mass spectrometer (Thermo Fisher Scientific, Rodano, Italy), equipped with a heated electrospray ionization source (HESI). Diluted samples (1:10 in acetonitrile) were injected into the flowing solvent (acetonitrile : water, 90:10 (v/v) acidified with 0.1 % (v/v) formic acid) at a flow rate of 300-400 μL/min and delivered directly to the ESI source by a syringe pump at constant flow (15-30 μL/min). The tuning parameters adopted for the ESI source were: ion spray voltage 3.4 kV and tube lens voltage 60%. The ion transfer tube and the vaporizer temperature were maintained at 275 °C and 290 °C, respectively. Sheat and auxiliary gases were set at 50 and 20 arbitrary unit, respectively. The mass accuracy of the recorded ions (vs. the calculated ones) was <5 mmu (milli-mass units). Analyses were run using full MS at 150-2000 m/z in the positive ion mode (resolution FWHM, at m/z 200: 120K). IR spectra were recorded on a Perkin Elmer BX FT-IR. Melting point analyses were carried out with a SMP3 Bibbi Stuart Scientific system.

S2. Optimisation of reaction conditions

Table S1: Screening of xanthyl group transfer agent

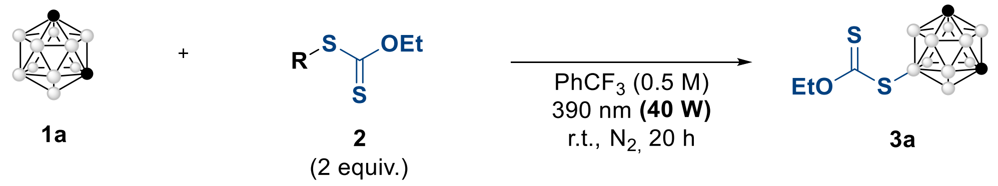

Entry	Xanthylating agent	Yield[a] (%)
1	2e	0
2	2d	0
3	2c	<5
4	2b	41
**5**	**2a**	**49**

1a (0.1 mmol, 1 equiv.); [a] Determined on the isolated product.

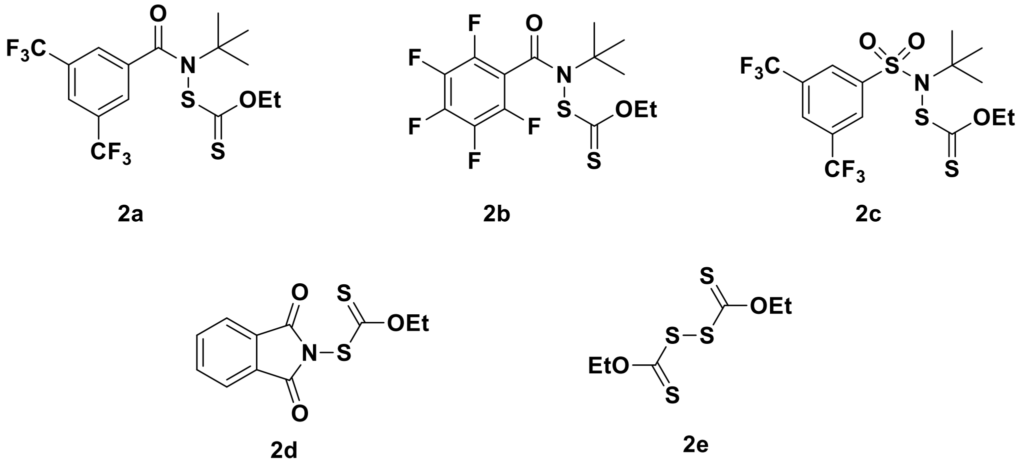

Table S2: Screening of concentration.

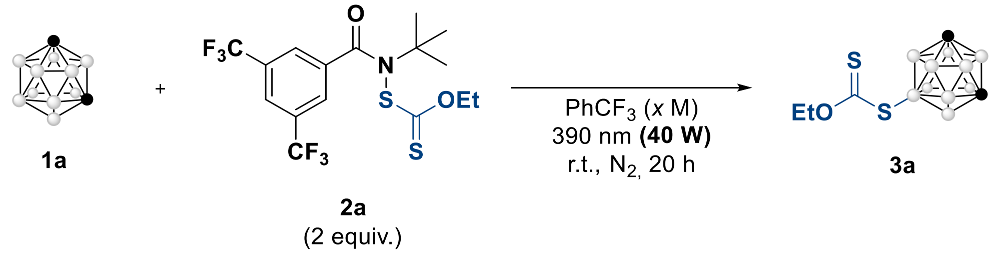

Entry	Concentration	Yield[a] (%)
**1**	**0.5**	**49**
2	0.3	40
3	0.1	42
4	0.05	40

1a (0.1 mmol, 1 equiv.); [a] Determined on the isolated product.

Table S3: Screening of light source

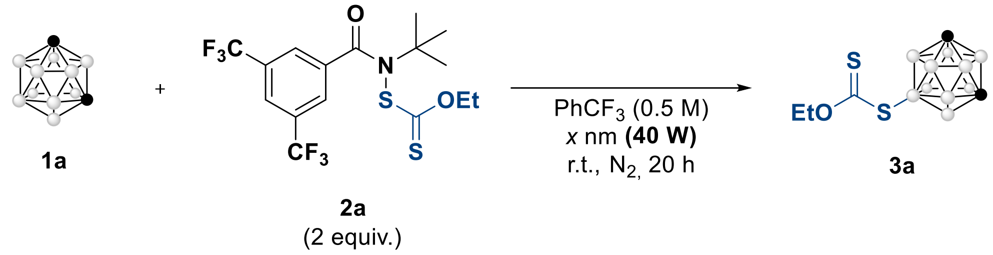

Entry	Wavelenght (nm)	Yield[a] (%)
1	390	49
2	440	20

1a (0.1 mmol, 1 equiv.); [a] Determined on the isolated product.

Table S4: Screening of xanthylamide **2a** equivalents

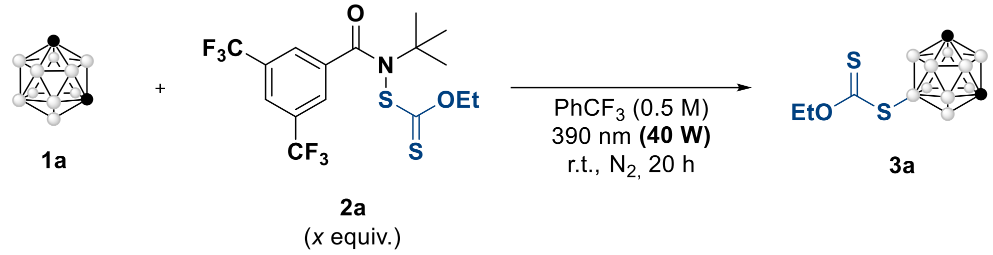

Entry	2a equiv.	Yield[a] (%)
1	1	25
2	1.2	28
3	1.5	37
**4**	**2**	**49**
5	4	10

1a (0.1 mmol, 1 equiv.); [a] Determined on the isolated product.

Table S5: Screening of combined effects of **2a** equivalents and concentration

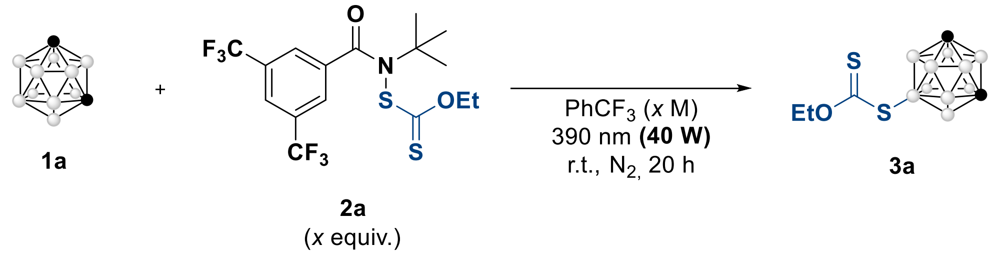

Entry	2a equiv.	Concentration	Yield[a] (%)
1	2	0.5	49
2	3	0.	57
3	4	0.5	10
**4**	**4**	**0.1**	**64**
5	5	0.1	64

1a (0.1 mmol, 1 equiv.); [a] Determined on the isolated product.

Table S6: Screening of solvent

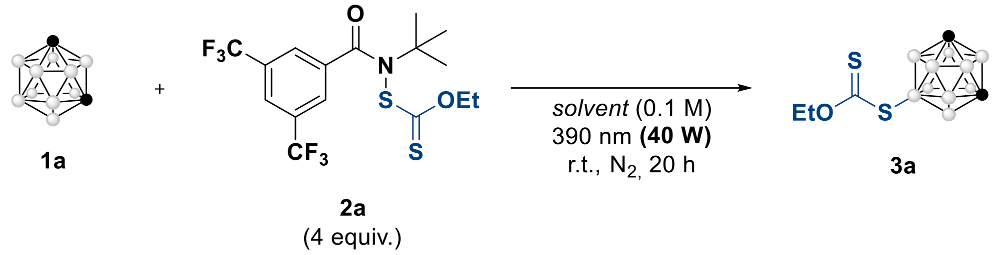

Entry	Solvent	Yield[a] (%)
**1**	**$PhCF_3$**	**64**
3	1,2-DCE	28
4	PhCl	42
5	THF	12
6	$PhCF_3$:1,2-DCE (2:1)	36

1a (0.1 mmol, 1 equiv.); [a] Determined on the isolated product.

Table S7: Control experiments

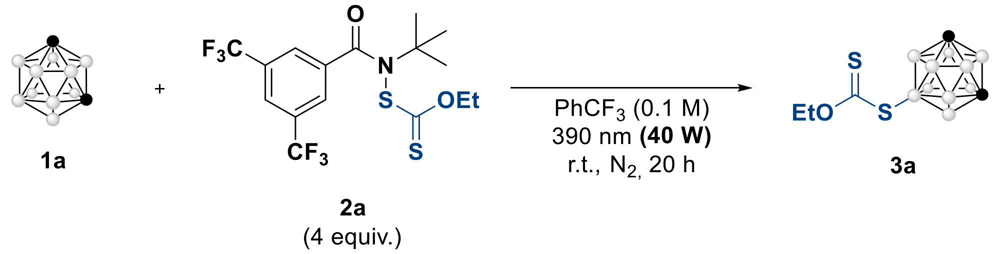

Entry	Control	Yield[a] (%)
1	Dark	0
2	Under air	22
3	TEMPO (4 equiv.)	0

1a (0.1 mmol, 1 equiv.); [a] Determined on the isolated product.

S3. Mechanistic study

S3.1. Deuterium Labelling

In order to demonstrate a HAT process from the *in situ*-generated nitrogen-centred radical and **1a** is operative in our reaction system, a deuterium labelling experiment with deuterated **d_2-1a** was conducted.

d_2-1a was synthesized following a reported procedure in the literature.[1] Spectroscopic data are coherent with those reported therein:

^{1}H-NMR (400 MHz, $CDCl_3$) δ: 2.91 (bs, 2H, carborane C-*H*), 3.43-1.45 (m, 10H, carborane B-*H*).

^{11}B{^{1}H}-NMR (128 MHz, $CDCl_3$) δ: -16.7, -13.3, -10.6, -6.6.

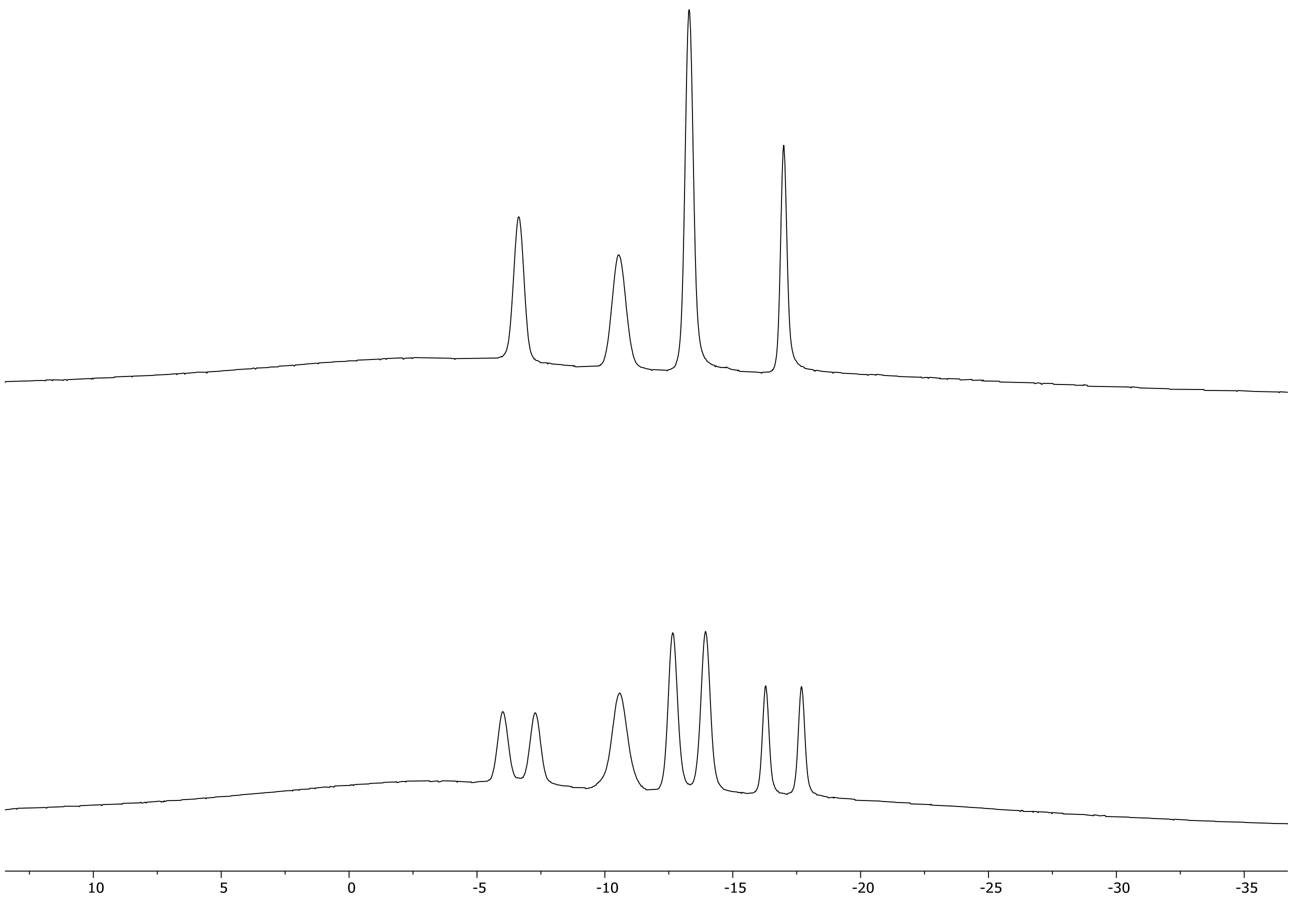

Figure S1. Comparison between $^{11}B\{^{1}H\}$-NMR and ^{11}B-NMR of **d_2-1a**.

From the reaction mixture of the standard xanthylation reaction between **1a** and **2a**, amide **5** can be isolated as a white solid. In the deuteration labelling experiment, **d_2-1a** was subjected to the optimised reaction conditions. After complete conversion of the starting material, the volatiles were removed *in vacuo* and the crude reaction was analysed by NMR spectroscopy, showing formation of deuterated amide **d-5**.

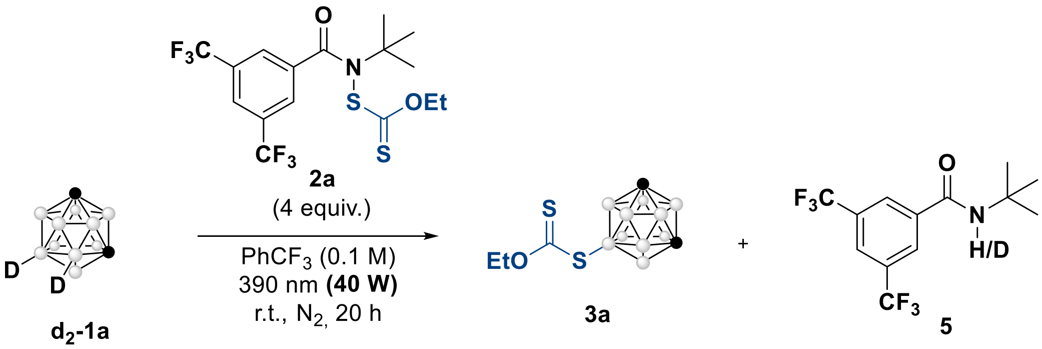

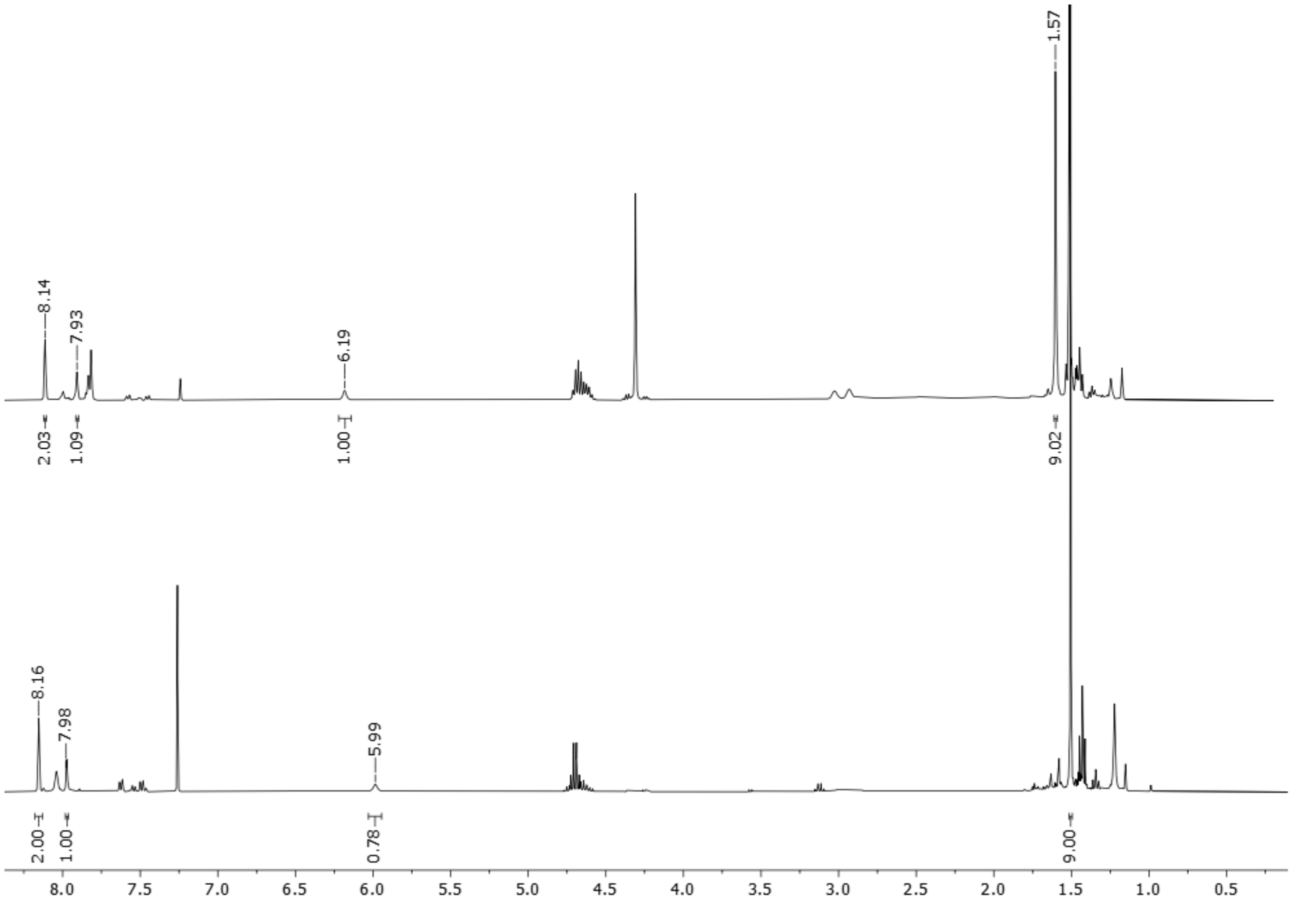

Figure S2. Comparison between ^{1}H-NMR of crude standard reaction with **1a** and ^{1}H-NMR of crude reaction with **d$_2$-1a**.

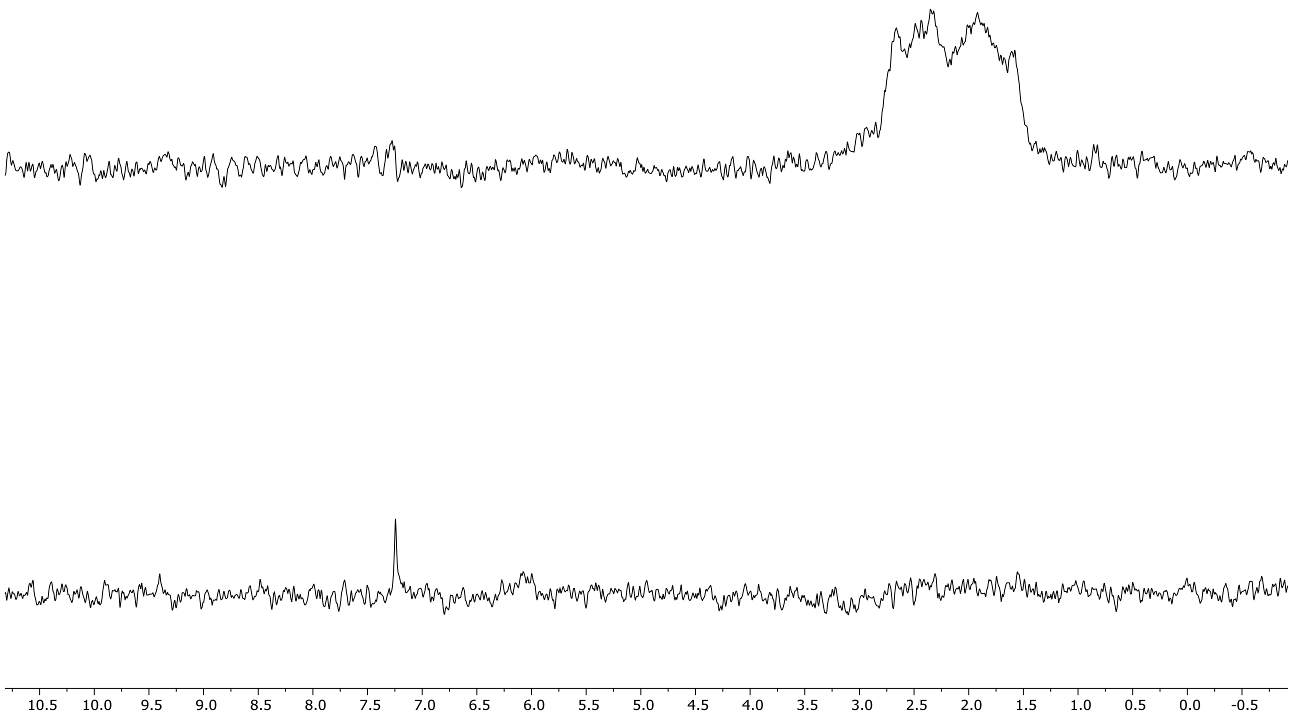

Figure S3. Comparison between ^{2}H-NMR of **d$_2$-1a** and ^{2}H-NMR of crude reaction with **d$_2$-1a**.

S3.2. EPR Spectroscopy

X-band continuous-wave (CW) EPR spectra (microwave frequency 9.5 GHz) were recorded on a Bruker EMX spectrometer equipped with an SHQ cylindrical cavity. The EPR samples, either 1 M xanthylamide **2a** alone or 1 M xanthylamide **2a** with 4 M *m*-carborane **1a** in (trifluoromethyl)benzene (trifluorotoluene), were thoroughly degassed using three freeze–pump–thaw cycles to remove dissolved oxygen. The solutions were then loaded into glass capillaries and irradiated *in situ* using a 415 nm LED (SOLIS-415C, Thorlabs).

For the measurements as a function of irradiation time (Figure S4, panels a and b), a modulation amplitude of 0.4 mT and a microwave power of 10 mW were used. To record the true linewidth (Figure S4, panel d), a modulation amplitude of 0.2 mT and a microwave power of 1 mW were employed. A modulation frequency of 100 kHz was used for all measurements.

The EPR spectrum of the N-amidyl radical was simulated using the EasySpin package,[2] based on the following spin Hamiltonian:

$$\mathcal{H} = \mu_B g_{iso} \hat{\boldsymbol{S}} \cdot \boldsymbol{B} + a_{iso} \hat{\boldsymbol{I}} \cdot \hat{\boldsymbol{S}}$$

where the first term describes the Zeeman interaction and the second term corresponds to the hyperfine interaction with the ^{14}N nucleus characteristic of nitrogen-based radical systems.

Figure S4 reports the primary EPR data. Panels a-c report the time-dependence of the EPR spectra as a function of the irradiation time. The data is presented both as stack plots of EPR spectra (panels a and b) and as time traces (panel c). The EPR spectrum of the *N*-amidyl radical in solution at room temperature can be simulated with a g_{iso} = 2.0049 ± 0.0001 and a ^{14}N isotropic hyperfine coupling a_{iso} = 41 ± 1 MHz (Figure XXXd). These values, which are comparable to the typical parameters for a nitroxide (aminoxyl) radical (g_{iso} ≈ 2.0056 and a_{iso} ≈ 43 MHz), suggest that the unpaired electron is delocalized over the amide bond.

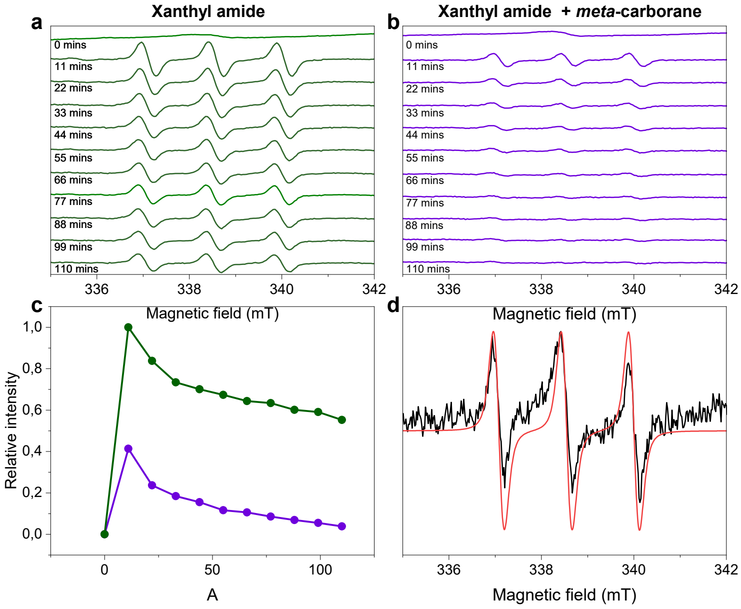

Figure S4: **a** – EPR spectra of the N-amidyl radical obtained from a 1 M solution of xanthylamide **2a** as a function of photoexcitation at 415 nm at room temperature. **b** – EPR spectra of the *N*-amidyl radical obtained

from a 1 M solution of xanthylamide **2a** in the presence of 4 M *m*-carborane **1a** under the same conditions. **c** – Decay traces of the *N*-amidyl radical derived from panels a and b, based on the peak-to-peak amplitude of the central line

S3.3. Computational method with references

S3.3.1. Computational method with references

The structures of the reactants, intermediates and transition states have been optimized by using the density functional method (DFT)[1] with the functional M06[2,3] and the basis sets def2-SVP extended with diffuse functions on non-hydrogen atoms (hereafter def2-SVP(D)).[4] The electronic energy values where refined by re-optimization with the basis set def2-TZVPD.[5] For the HATreaction, the energies from single-point calculations with the basis set def2-QZVPD[6] at the def2-TZVPD geometies, were used instead. The nature of the critical points was characterized by using vibrational analysis[7] which also the thermal corrections for the calulation of the Entalphy and the Free Energies.[7] The solvent effects (trifluorotoluene) were introduced in all calculations using the universal solvation model (SMD) by Truhalr *et al*.[8]

The rate constants of the reactions are calculated using canonical transition state theory. [9]

The vertical electronic transition energies and the first singlet excited state structuries have been calculated with the Time-Dependet DFT[10] with the def2-SVP(D) basis set. The calculations were performed by the quantum package Gaussian 16-A.03[11] The Karlsruhe's "def2" basis set was taken form ref 12. The figures were obtained using the graphical program Molden.[13]

[1] R.G. Parr, Density Functional Theory of Atoms and Molecules, in: Horizons Quantum Chem., Springer Netherlands, 1980: pp. 5–15. DOI:10.1007/978-94-009-9027-2_2.

[2] Y. Zhao, D.G. Truhlar, "The M06 suite of density functionals for main group thermochemistry, thermochemical kinetics, noncovalent interactions, excited states, and transition elements: Two new functionals and systematic testing of four M06-class functionals and 12 other function." *Theor. Chem. Acc.* 120 (2008) 215–241, DOI: 10.1007/s00214-007-0310-x.

[3] Y. Zhao, D.G. Truhlar "Density functionals with broad applicability in chemistry." *Acc. Chem. Res.* 41 (2008) 157–167, DOI: 10.1021/ar700111a.

[4] A. Schaefer, H. Horn, and R. Ahlrichs, "Fully optimized contracted Gaussian-basis sets for atoms Li to Kr." *J. Chem. Phys.*, 97 (1992) 2571–2577. DOI: 10.1063/1.463096.

[5] A. Schaefer, C. Huber, and R. Ahlrichs, "Fully optimized contracted Gaussian-basis sets of triple zeta valence quality for atoms Li to Kr." *J. Chem. Phys.*, 100 (1994) 5829–5835. DOI: 10.1063/1.467146 .

[6] (a) F. Weigend, F. Furche, R. Ahlrichs, "Gaussian basis sets of quadruple zeta valence quality for atoms H-Kr." *J. Chem. Phys.* 119, 12753–12762 (2003). DOI: 10.1063/1.1627293; (b) D. Rappoport, F. Furche "Property-optimized Gaussian basis sets for molecular response calculations." *J. Chem. Phys.* 133, 134105 (2010). DOI: 10.1063/1.3484283

[7] J. Foresman, A. Frisch, Exploring chemistry with electronic structure methods, 1996, Gaussian Inc, Pittsburgh, PA, 1996, http://gaussian.com/expchem3/(accessed June 4, 2021).

[8] A. V. Marenich, C. J. Cramer, and D. G. Truhlar, "Universal solvation model based on solute electron density and a continuum model of the solvent defined by the bulk dielectric constant and atomic surface tensions," *J. Phys. Chem. B*, 113 (2009) 6378–6396. DOI: 10.1021/jp810292n .

[9] D. G. Truhlar, B. C. Garrett, S. J. Klippenstein "Current Status of Transition-State Theory" *J. Phys. Chem.* 100 (1996) 12771

[10] F. Furche and R. Ahlrichs, "Adiabatic time-dependent density functional methods for excited state properties," *J. Chem. Phys.*, 117 (2002) 7433–7447. DOI: 10.1063/1.1508368

[11] D.J. Frisch, M. J.; Trucks, G. W.; Schlegel, H. B.; Scuseria, G. E.; Robb, M. A.; Cheeseman, J. R.; Scalmani, G.; Barone, V.; Petersson, G. A.; Nakatsuji, H.; Li, X.; Caricato, M.; Marenich, A. V.; Bloino, J.; Janesko, B. G.; Gomperts, R.; Mennucci, B.; Hratch, Gaussian 16, Revision A.03, (2016).

[12]. B. P. Pritchard, D. Altarawy, B. Didier, T. D. Gibson, T. L. Windus. “A New Basis Set Exchange: An Open, Up-to-date Resource for the Molecular Sciences Community” *J. Chem. Inf. Model.* 2019, 59(11), 4814-4820, doi:10.1021/acs.jcim.9b00725

[13] G. Schaftenaar, J.H. Noordik, Molden: A pre- and post-processing program for molecular and electronic structures, J. Comput. Aided. Mol. Des. 14 (2000) 123–134, DOI: 10.1023/A:1008193805436.

S3.3.2. The C–H and B–H: Tables with absolute and relative (in kcal mol^{-1}) energies & Pictures.

Table S3.3.2a. *ortho*-Carborane C–H and B–H bond energies.

***ortho*-Carborane**	E /au[a]	ΔE[a]	ZPE /au[a]	δH^{298K} /au[a]	E TZD/ au[b]	ΔE[c]	ΔH^{298K,c}
ortho-Carborane	-331.667594	0.00	0.17588	0.18435	-332.006310		
H Rad.	-0.497585	*1.52*	0.00000	0.00236	-0.499694		
C-de-H *ortho*-Carborane Rad.	-330.983807		0.16264	0.17118	-331.320473		
**C-H Bond energies**	0.186202	116.84	-0.01324	-0.010811	0.186143	116.81	110.0
B(3/6)-de-H *ortho*-Carborane Rad.	-330.991440		0.16511	0.17350	-331.327872		
***o*-CB $B_{3/6}$-H Bond energies**	0.178569	112.05	-0.01077	-0.00848	0.178744	112.16	106.8
B(4/5/7/11)-de-H *ortho*-Carborane Rad.	-330.994569		0.16520	0.17358	-331.330912		
***o*-CB $B_{4/5/7/11}$-H Bond energies**	0.175440	110.09	-0.01068	-0.00840	0.175704	110.26	105.0
B(8/10)-de-H *ortho*-Carborane Rad.	-330.996577		0.16538	0.17376	-331.332886		
***o*-CB $B_{8/10}$-H Bond energies**	0.173432	108.83	-0.01050	-0.00823	0.173731	109.02	103.9
B(9/12)-de-H *ortho*-Carborane Rad.	-330.997274		0.16541	0.17379	-331.333468		
***o*-CB $B_{9/12}$-H Bond energies**	0.172735	108.39	-0.01047	-0.00820	0.173149	108.65	103.5

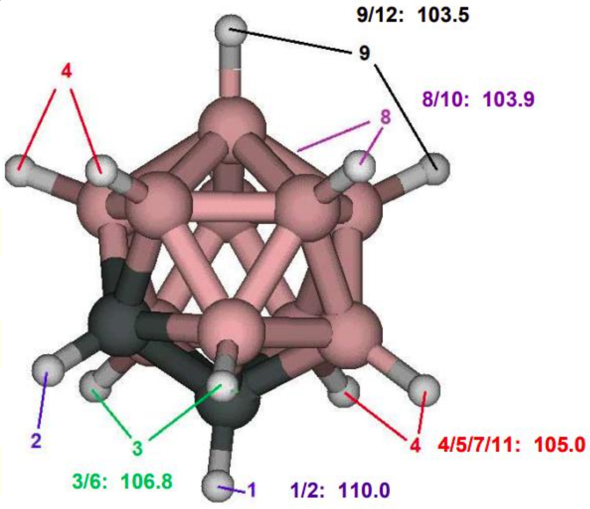

[a] M06/def2-SVP(D); [b] M06/def2-TZVPD; [c] M06/def2-TZVPD enegies combined with thermal corrections M06/def2-SVP(D).

Table S3.3.2b. 1-Methyl-*ortho*-Carborane C–H and B–H bond energies.

1-Methyl-*ortho*-Carborane	E /au[a]	ΔE[a]	ZPE /au[a]	δH[298K] /au[a]	E TZD/ au[b]	ΔE[c]	ΔH[298K,c]
1-Methyl-*ortho*-Carborane	-370.924801		0.20290	0.213130	-371.306699		
C-de-H *ortho*-Carborane Rad.	-370.241163		0.18958	0.199942	-370.620936		
**C-H Bond energy**	0.186053	116.75	-0.01331	-0.010828	0.186069	116.76	110.0
Rad. De-H on Me	-370.254445		0.18779	0.198450	-370.635162		
**C-H (CH_3) Bond energy**	0.172771	108.42	-0.01510	-0.012320	0.171843	107.83	100.1
B(9)-de-H *ortho*-Carborane Rad.	-370.254560		0.19221	0.202497	-370.633954		
**B(9)-H Bond energy**	0.172656	108.34	-0.01068	-0.008273	0.173051	108.59	103.4
B(12)-de-H *ortho*-Carborane Rad.	-370.254306		0.19254	0.202648	-370.633705		
**B(12)-H Bond energy**	0.172909	108.50	-0.01036	-0.008122	0.173300	108.75	103.7

[a] M06/def2-SVP(D); [b] M06/def2-TZVPD; [c] M06/def2-TZVPD enegies combined with thermal corrections M06/def2-SVP(D).

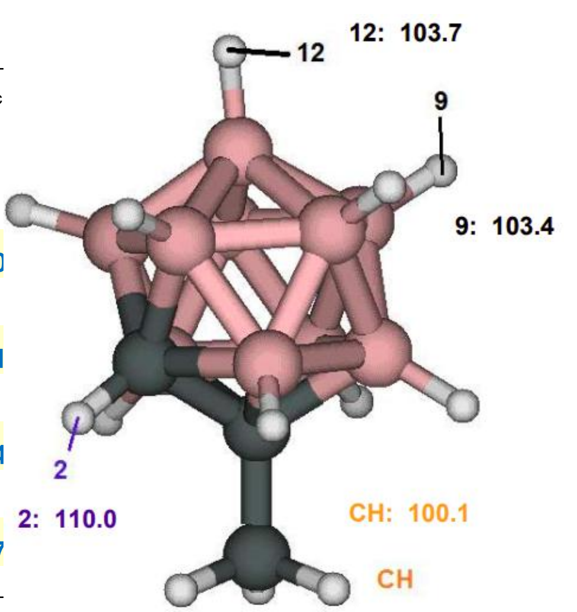

Table S3.3.2c. 1-Methoxyl-*ortho*-Carborane C–H and B–H bond energies.

1-Methoxyl-*ortho*-Carborane	E /au[a]	ΔE[a]	ZPE /au[a]	δH[298K] /au[a]	E TZD/ au[b]	ΔE[c]	ΔH[298K,c]
1-Methoxyl-*ortho*-Carborane	-446.038521		0.20760	0.218737	-446.501299		
Rad. De-H on Me	-445.368378		0.19310	0.204511	-445.830379		
**C-H (CH_3) Bond energy**	0.172558	108.28	-0.01450	-0.011866	0.171226	107.45	100.0
B(9)-de-H *ortho*-Carborane Rad.	-445.368560		0.19705	0.208137	-445.828922		
**B(9)-H Bond energy**	0.172376	108.17	-0.01055	-0.008240	0.172684	108.36	103.2

[a] M06/def2-SVP(D); [b] M06/def2-TZVPD; [c] M06/def2-TZVPD enegies combined with thermal corrections M06/def2-SVP(D).

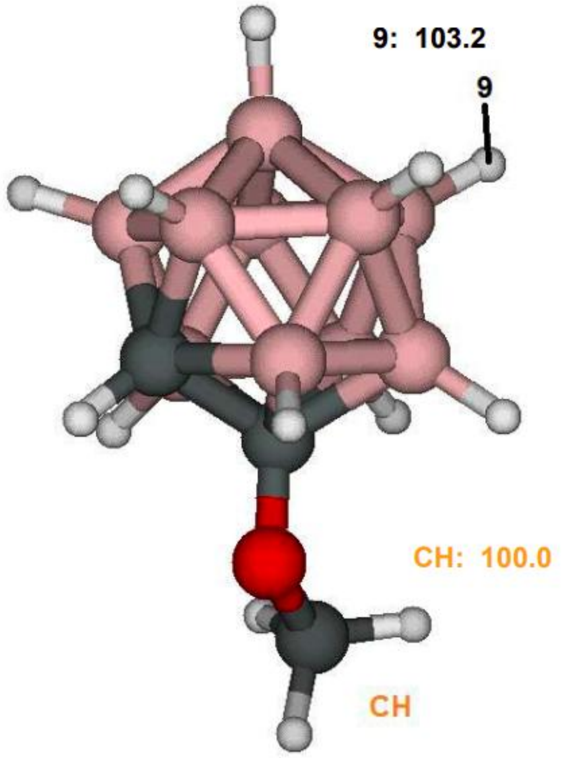

Table S3.3.2d. 1-Hydroxyl-*ortho*-Carborane C–H and B–H bond energies.

**1-Hydroxy-*ortho*-Carborane**	E /au[a]	ΔE[a]	ZPE /au[a]	δH[298K] /au[a]	E TZD/ au[b]	ΔE[c]	ΔH[298K,c]
1-Hydroxy-*ortho*-Carborane	-406.803759		0.17950	0.189331	-407.221257		
Rad. De-H on Me	-406.128507		0.16561	0.175330	-406.547996		
**O-H (OH) Bond energy**	0.177666	**111.49**	-0.01388	-0.011641	0.173567	**108.91**	**101.6**
B(9)-de-H *ortho*-Carborane Rad.	-406.133727		0.16905	0.178792	-406.548815		
**B(9)-H Bond energy**	0.172446	**108.21**	-0.01044	-0.008179	0.172748	**108.40**	**103.3**

[a] M06/def2-SVP(D); [b] M06/def2-TZVPD; [c] M06/def2-TZVPD enegies combined with thermal corrections M06/def2-SVP(D).

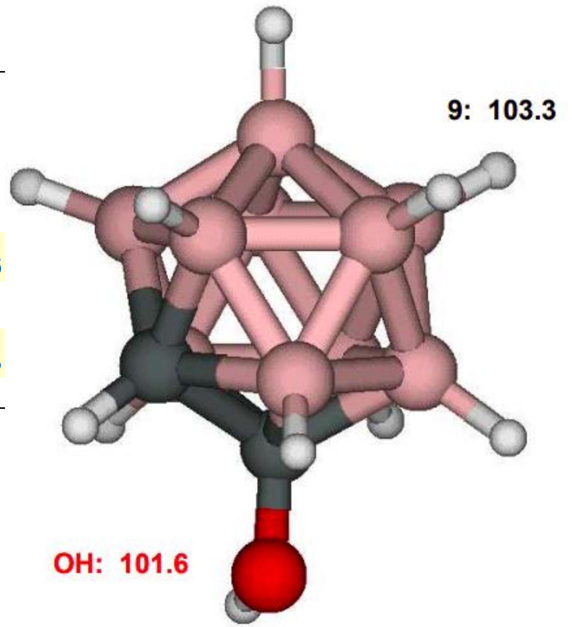

Table S3.3.2e. 1-Trimethylsilyl-*ortho*-Carborane C–H and B–H bond energies.

**1-Me_3Si-*ortho*-Carborane**	E /au[a]	ΔE[a]	ZPE /au[a]	δH[298K] /au[a]	E TZD/ au[b]	ΔE[c]	ΔH[298K,c]
1-Me_3Si-*ortho*-Carborane	-740.062267		0.27491	0.291414	-740.637504		
Rad. De-H on CH_3	-739.393881		0.26155	0.278098	-739.968422		
**C-H (CH_3) Bond energy**	0.170800	**107.18**	-0.01336	-0.010956	0.169387	**106.29**	**99.4**

[a] M06/def2-SVP(D); [b] M06/def2-TZVPD; [c] M06/def2-TZVPD enegies combined with thermal corrections M06/def2-SVP(D).

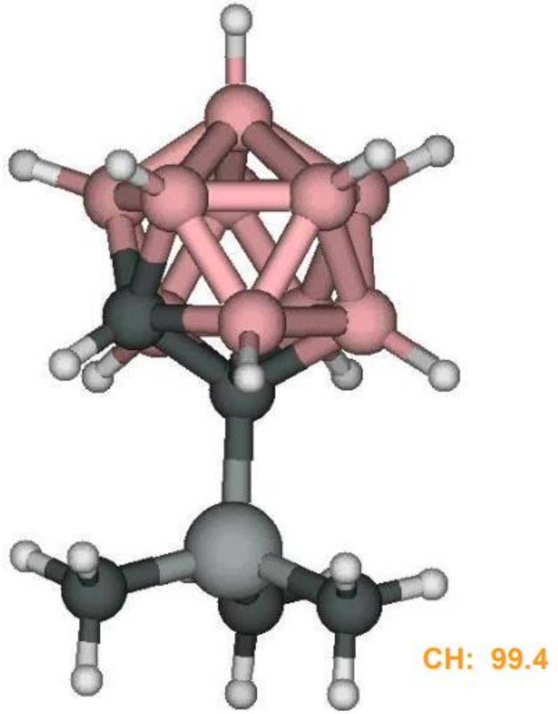

Table S3.3.2f. 1-Methoxycarbonylmethyl-*ortho*-Carborane C–H and B–H bond energies.

**1-MeOCOOCH2-*ortho*-Carborane**	E /au[a]	ΔE[a]	ZPE /au[a]	δH^{298K} /au[a]	E TZD/ au[b]	ΔE[c]	$\Delta H^{298K,c}$
1-MeOCOOCH2-*ortho*-Carborane	-673.671778		0.25189	0.267184	-674.380566		
Rad. De-H on CH2	-673.010223		0.23701	0.252737	-673.718873		
**C-H (CH_2) Bond energy**	0.163970	**102.89**	-0.01487	-0.012087	0.162000	**101.66**	**94.[illegible]**
B(9)-de-H *ortho*-Carborane Rad.	-673.001538		0.24145	0.256644	-673.707859		
**B(9)-H Bond energy**	0.172655	**108.34**	-0.01043	-0.008180	0.173013	**108.57**	**103.[illegible]**

[a] M06/def2-SVP(D); [b] M06/def2-TZVPD; [c] M06/def2-TZVPD enegies combined with thermal corrections M06/def2-SVP(D).

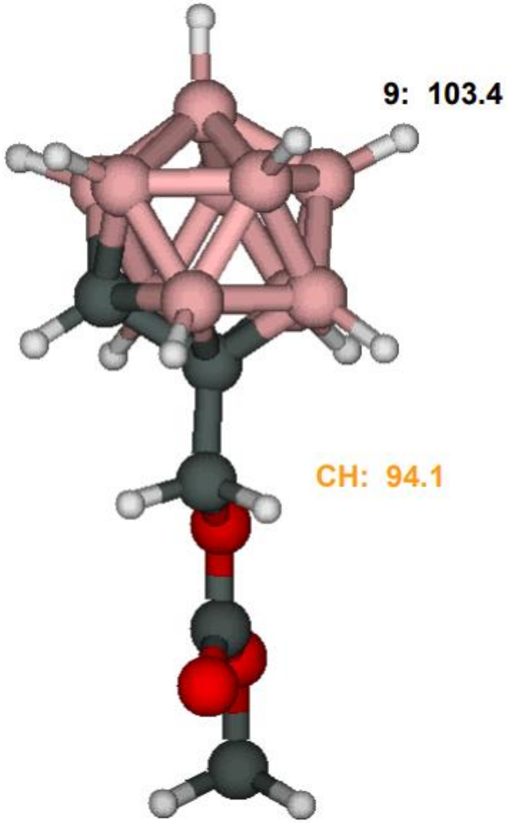

Table S3.3.2g. 1-Bromomethyl-*ortho*-Carborane C–H and B–H bond energies.

**1-BrCH2-*ortho*-Carborane**	E /au[a]	ΔE[a]	ZPE /au[a]	δH^{298K} /au[a]	E TZD/ au[b]	ΔE[c]	$\Delta H^{298K,c}$
1-BrCH2-*ortho*-Carborane	-2944.073739		0.19388	0.205163	-2944.768731		
Rad. De-H on CH2Br	-2943.413173		0.17985	0.191389	-2944.107361		
**C-H (CH_3) Bond energy**	0.162981	**102.27**	-0.01402	-0.011414	0.161676	**101.45**	**94.3**

[a] M06/def2-SVP(D); [b] M06/def2-TZVPD; [c] M06/def2-TZVPD enegies combined with thermal corrections M06/def2-SVP(D).

Table S3.3.2h. *meta*-Carborane C–H and B–H bond energies.

***meta*-Carborane**	E /au[a]	ΔE[a]	ZPE /au[a]	δH[298K] /au[a]	E TZD/ au[b]	ΔE[c]	ΔH[298K,c]
meta-Carborane	-331.691541	-15.03	0.17621	0.184635	-332.029708		
B(4/6/8/11)-de-H *meta*-Carborane Rad.	-331.018010		0.16553	0.173875	-331.353869		
***m*-CB $B_{4/6/8/11}$-H Bond energies**	0.175945	110.41	-0.01068	-0.008400	0.176145	110.53	105.3
B(5/12)-de-H *meta*-Carborane Rad.	-331.018631		0.16558	0.173922	-331.354410		
***m*-CB $B_{5/12}$-H Bond energies**	0.175325	110.02	-0.01062	-0.008353	0.175604	110.19	105.0
B(9/10)-de-H *meta*-Carborane Rad.	-331.020386		0.16566	0.173996	-331.356152		
***m*-CB $B_{9/10}$-H Bond energies**	0.173570	108.92	-0.01055	-0.008279	0.173862	109.10	103.9
B(2/3)-de-H *meta*-Carborane Rad.	-331.015997		0.165397	0.173745	-331.351962		
***m*-CB $B_{2/3}$-H Bond energies**	0.177959	108.92	-0.010818	-0.008530	0.178052	111.73	106.4

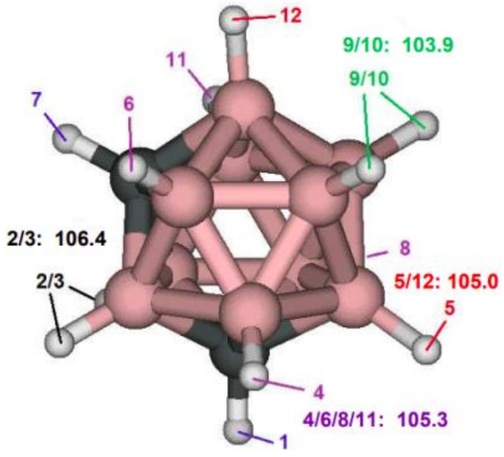

[a] M06/def2-SVP(D); [b] M06/def2-TZVPD; [c] M06/def2-TZVPD enegies combined with thermal corrections M06/def2-SVP(D).

Table S3.3.2i. 1-Methyl-*meta*-Carborane C–H and B–H bond energies.

**1-Methyl-*meta*-Carborane**	E /au[a]	ΔE[a]	ZPE /au[a]	δH[298K] /au[a]	E TZD/ au[b]	ΔE[c]	ΔH[298K,c]
1-Methyl-*meta*-Carborane	-370.946601		0.20324	0.213470	-371.328097		
Rad. De-H on Me	-370.277176		0.18839	0.198901	-370.657267		
**C-H (CH_3) Bond energy**	0.171840	107.83	-0.01484	-0.012209	0.171136	107.39	99.7
B(9/10)-de-H *meta*-Carborane Rad.	-370.275726		0.19280	0.202948	-370.654786		
***m*-CB $B_{9/10}$-H Bond energies**	0.173289	108.74	-0.01043	-0.008162	0.173617	108.95	103.8

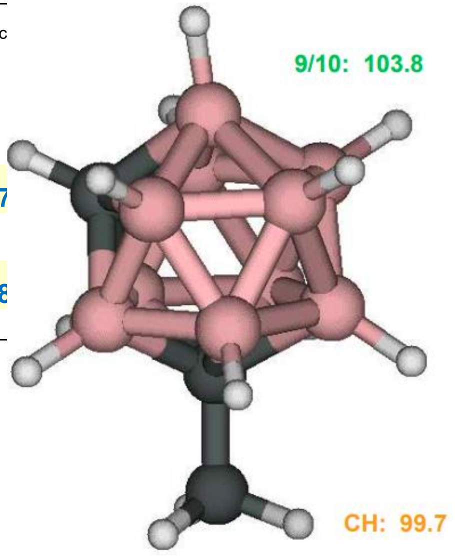

[a] M06/def2-SVP(D); [b] M06/def2-TZVPD; [c] M06/def2-TZVPD enegies combined with thermal corrections M06/def2-SVP(D).

S3.3.3. 2a and its Photolysis: Tables with absolute and relative (in kcal mol^{-1}) energies & Pictures.

Table S3.3.3a. The conformers of 2a.

2a conformes[a]		E /au[b]	ΔE^{b}	ZPE /au[b]	δG^{298K} /au[b]	E /au[b]	ΔE^{c}	$\Delta E^{0K,d}$	$\Delta G^{298K,d}$	N-S[e]
anti,syn,gauche-out	**2a-1**	-2218.625972	**1.68**	0.315346	0.253493	-2220.444047	**1.33**	**0.43**	**-1.28**	**1.706**
anti,syn,gauche-inner	**2a-2**	-2218.626331	**1.45**	0.315824	0.255415	-2220.444530	**1.03**	**0.43**	**-0.37**	**1.707**
anti,anti,gauche-out	**2a-3**	-2218.628650	**0.00**	0.316776	0.257646	-2220.446164	**0.00**	**0.00**	**0.00**	**1.724**
anti,anti,gauche-in	**2a-4**	-2218.628197	**0.28**	0.316916	0.258078	-2220.445801	**0.23**	**0.32**	**0.50**	**1.723**
syn,anti,gauche-out	**2a-5**	-2218.621374	**4.57**	0.316042	0.255159	-2220.439479	**4.19**	**3.73**	**2.63**	**1.728**
syn,syn,gauche-inner	**2a-6**	-2218.619123	**5.98**	0.315781	0.254563	-2220.437230	**5.61**	**4.98**	**3.67**	**1.716**
syn,syn,gauche-out	**2a-7**	-2218.619115	**5.98**	0.315890	0.255142	-2220.437227	**5.61**	**5.05**	**4.04**	**1.715**

[a] the structure names refere to the dihedral defined by: O=C–N–S, N–S–C=S, C–O–C–Me vs F; [b] M06/def2-SVP(D); [c] M06/def2-TZVPD; [d] M06/def2-TZVPD enegies combined with thermal corrections M06/def2-SVP(D); [e] N–S distances in Å.

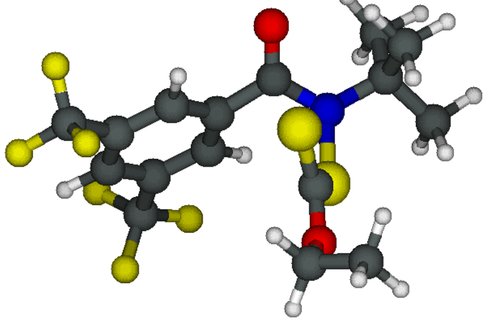
2a-1

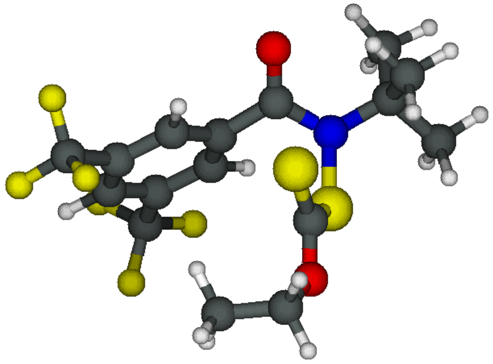
2a-2

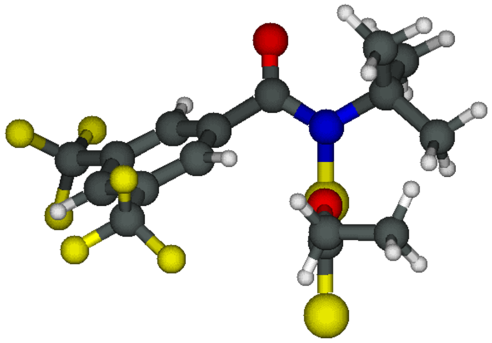
2a-3

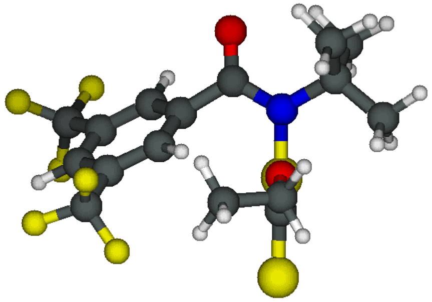
2a-4

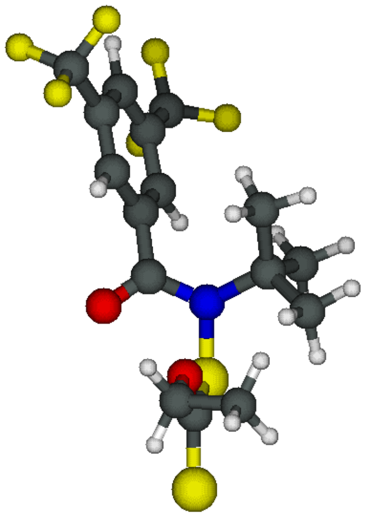

2a-5

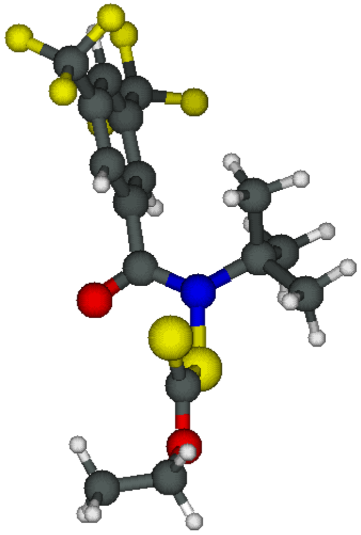

2a-6

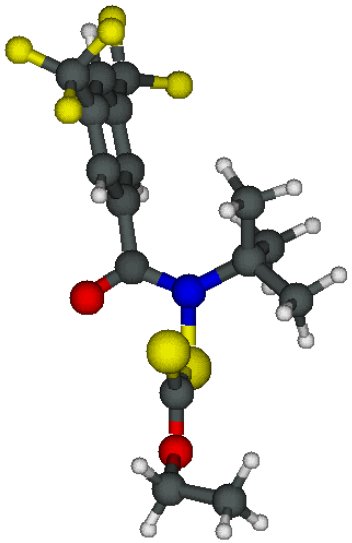

2a-7

Table S3.3.3b. The first excited state and photolysis of 2a.

		E /au[a]	ΔE[b]	ZPE /au[a]	δG^{298K} /au[a]	ΔE[c]	$\Delta E^{0K,c}$	$\Delta G^{298K,c}$	Δn	N-S[d]
**S_1 2a-1***	**2a-1***	-2218.533088	***58.29***	0.313512	0.251965	**0.00**	**0.00**	**0.00**	**0**	**1.824**
**S_1 TS_{N-S} from S_1 2a-1***	**TS-1***	-2218.531369	**59.36**	0.312646	0.250881	**1.08**	**0.54**	**0.40**	**0**	**2.083**
**Radical Couple**	**RC**	-2218.555607	***44.15***	0.312597	0.250405	**-14.13**	**-16.75**	**-18.67**	**0**	**3.475**
Rad. 3,5-(CF3)2C6H3-CO-N*(tert-Bu)		-1230.161153		0.233456	0.182030					
Rad. CH3CH2O-CS2*		-988.383621		0.077596	0.044552					
**Radical Fragments**		-2218.544774	***50.95***	0.311052	0.226582	**-7.33**	**-10.93**	**-24.93**	**1**	
**S_1 2a-2***	**2a-2***	-2218.534168	***57.83***	0.313858	0.253448	**0.00**	**0.00**	**0.00**		**1.810**
**S_1 TS_{N-S} from S_1 2a-2***	**TS-2***	-2218.531671	**59.40**	0.312917	0.253012	**1.57**	**0.98**	**1.29**		**2.108**
**S_1 2a-3***	**2a-3***	*-2218.530237*	***61.76***	0.000000		**(e)**				***2.254***
**S_1 2a-4***	**2a-4***	-2218.528020	***62.86***	0.313605	0.254505	**0.00**	**0.00**	**0.00**		**1.874**
**S_1 TS_{N-S} from S_1 2a-4***	**TS-4***	-2218.527593	**63.13**	0.312867	0.253726	**0.27**	**-0.20**	**-0.22**		**2.048**

[a] M06/def2-SVP(D) absolute energies; [b] relative energies calculated with respect to the corresponding ground state isomer; [c] relative energies calculated with respect to the corresponding excited state isomer ; [d] N–S distances in Å; [e] not fully optimized due to TD convergence problems..

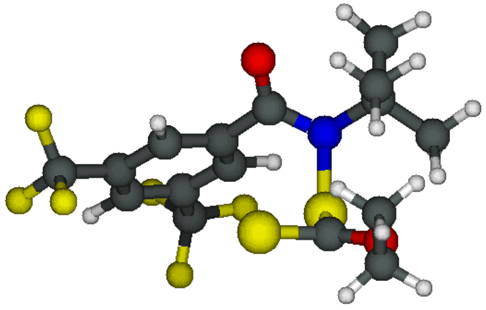

2a-1*

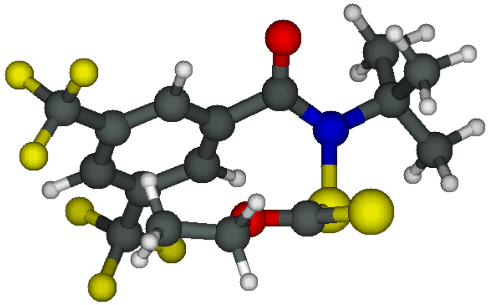

2a-2*

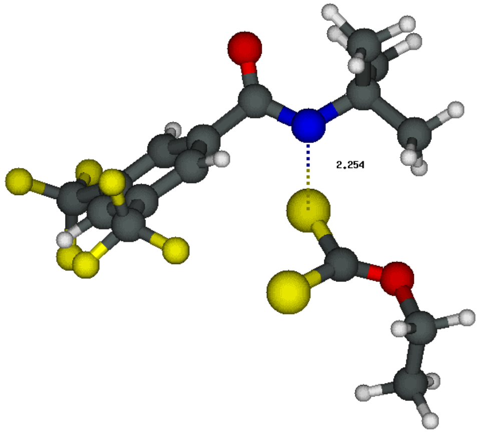

2a-3*

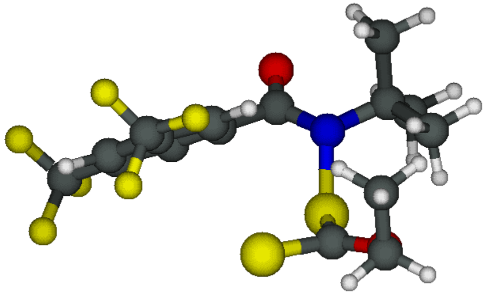

2a-4*

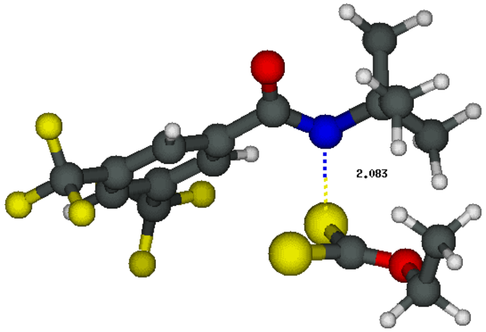

TS-1*

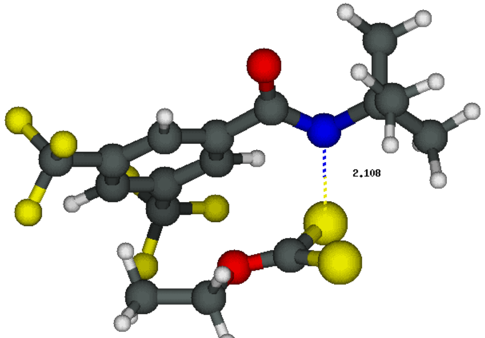

TS-2*

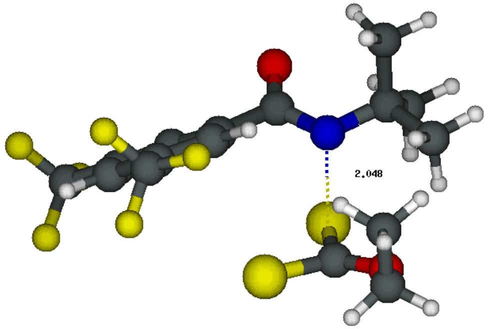

TS-4*

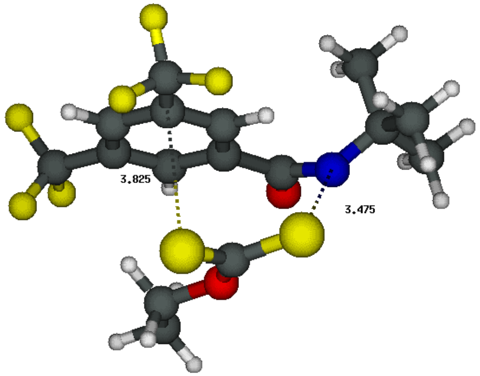

RC

S3.3.4. The HAT from *ortho*-Carborane to the *N*-Centred Radical from 2a: Tables with absolute and relative (in kcal mol^{-1}) energies & Pictures.

Table S3.3.4. The first excited state and photolysis of 2a.

	E /au[a]	ΔE[a]	δH^{298K} /au[a]	δG^{298K} /au[a]	E TZD/ au[b]	ΔE[b]	E QZD/ au[c]	ΔE[c]	ΔH^{298K,c}	ΔG^{298K,c}
ortho-Carborane	-331.667594		0.184355	0.145245	-332.006310		-332.044630			
3,5-$(CF_3)_2C_6H_3$-CO-N*-tBu Rad.	-1230.161153		0.254444	0.182030	-1231.511976		-1231.589991			
***ortho*-Carborane + *N*-CR**	-1561.828748	**0.00**	0.438799	0.327275	-1563.518286	**0.00**	-1563.634621	**0.00**	**0.00**	**0.00**
**TS_{HAT} on B(9/12)H**	-1561.820571	**5.13**	0.436332	0.346882	-1563.506721	**7.26**	-1563.621950	**7.95**	**6.40**	**20.25**
B(9/12)-de-H *ortho*-Carborane Rad.	-330.997274		0.173791	0.134176	-331.333468		-331.371378			
3,5-$(CF_3)_2C_6H_3$-CO-NH-tBu	-1230.839736		0.268473	0.196078	-1232.189662		-1232.269346			
**Prods**	-1561.837010	**-5.18**	0.442264	0.330254	-1563.523130	**-3.04**	-1563.640724	**-3.83**	**-1.66**	**-1.96**

[a] M06/def2-SVP(D); [b] M06/def2-TZVPD; [c] Single-Point M06/def2-QZVPD//def2-TZVPD energies combined with thermal corrections M06/def2-SVP(D).

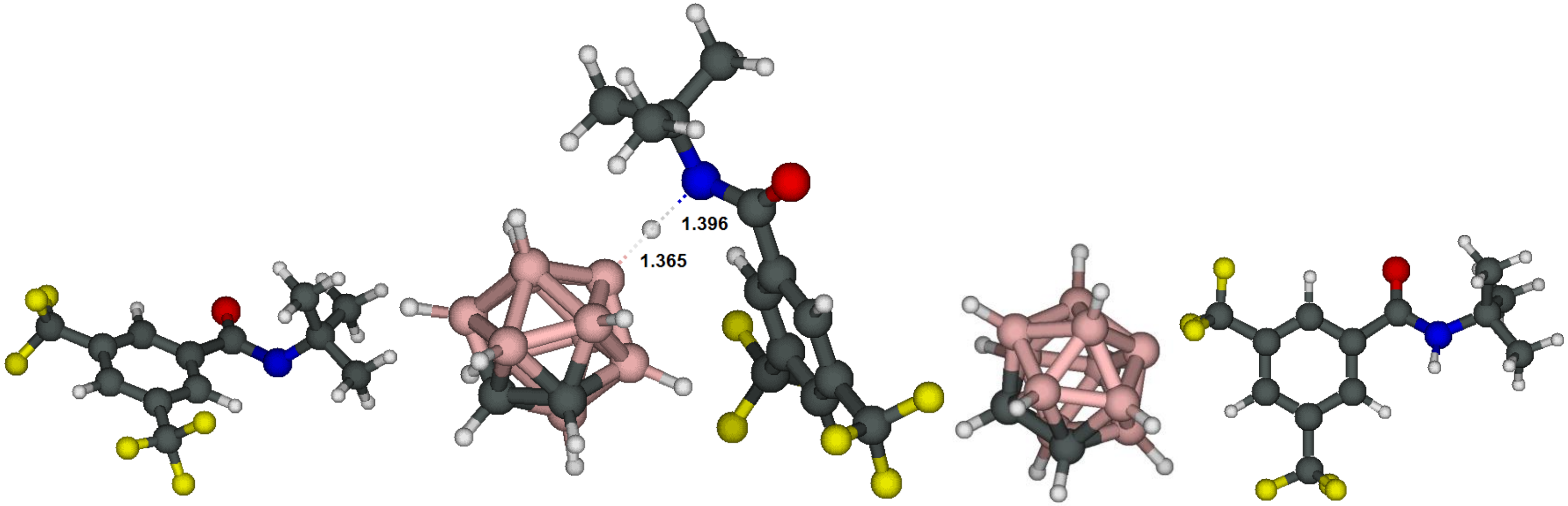

NCR **TS_{HAT}** **B(9)-de-H *o*-CB** **3,5-$(CF_3)_2C_6H_3$-CO-NH**

S4. Experimental procedures

S4.1. Synthesis of 2e

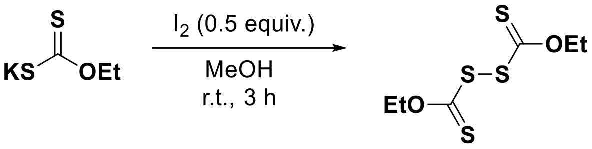

2e

Bis-ethoxythiocarbonyldisulfane (2e)

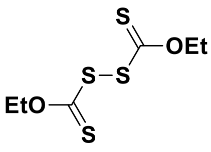

2e

Following a modified procedure of the reported literature:[3] in a 100 ml round bottom flask, iodine (1.764 g, 14 mmol, 0.5 equiv.) is dissolved in distilled water (30 ml) and potassium ethyl xanthogenate (4.480 g, 28 mmol, 1 equiv.) was added portion-wise. The reaction mixture is then concentrated *in vacuo* and the product is precipitated with water. The solid is washed with cold distilled water and aq. sat. $Na_2S_2O_3$. The product is obtained as a yellow solid (0.822 mg, 72%) and is used without further purification. Spectral data are coherent with those reported in the literature.[3]

^{1}H-NMR (400 MHz, $CDCl_3$) δ: 1.43 (t, *J* = 7.12 Hz, 6H, O-CH_2-CH_3), 4.70 (q, *J* = 7.12 Hz, 4H, O-CH_2-CH_3).

S4.2. Synthesis of 2d

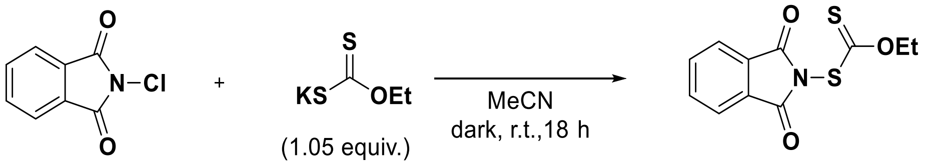

2d

N-ethylxanthyl phthalimide (2c)

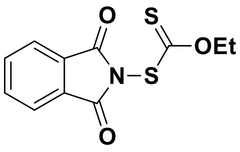

2d

Following a modified procedure of the reported literature:[4] in a 250 ml round bottom flask, covered with aluminium foil to minimise exposition to ambient light, potassium ethyl xanthogenate (1.60 g, 10 mmol 1.05 equiv.) is suspended in MeCN (60 ml). *N*-chloro phtalimide (1.816 g, 10 mmol, 1 equiv.) is added as a solution in MeCN (20 ml) over 20 min *via* an addition funnel. The reaction is left to stir at room temperature for 18 h, after which it is concentrated *in vacuo*. The reaction crude is taken in DCM/H_2O. The aqueous phase is extracted with DCM (3 x 50 ml) and the combined organic phases are washed with brine, dried over Na_2SO_4, and concentrated *in vacuo* The crude product is then purified by flash column chromatography (PE/EtOAc 90/10) to afford **2c** as a white solid (2.052 g, 76%). Spectral data are coherent with those reported in the literature.[4]

^{1}H-NMR (400 MHz, $CDCl_3$) δ: 1.34 (t, *J* = 7.12 Hz, 3H, O-CH_2-CH_3), 4.65 (q, *J* = 7.12 Hz, 4H, O-CH_2-CH_3), 7.85 (dd, *J* = 3.12, 5.52 Hz, 2H, Ar-*H*), 8.00 (dd, *J* = 3.12, 5.52 Hz, 2H, Ar-*H*).

S4.3. Synthesis of 2c

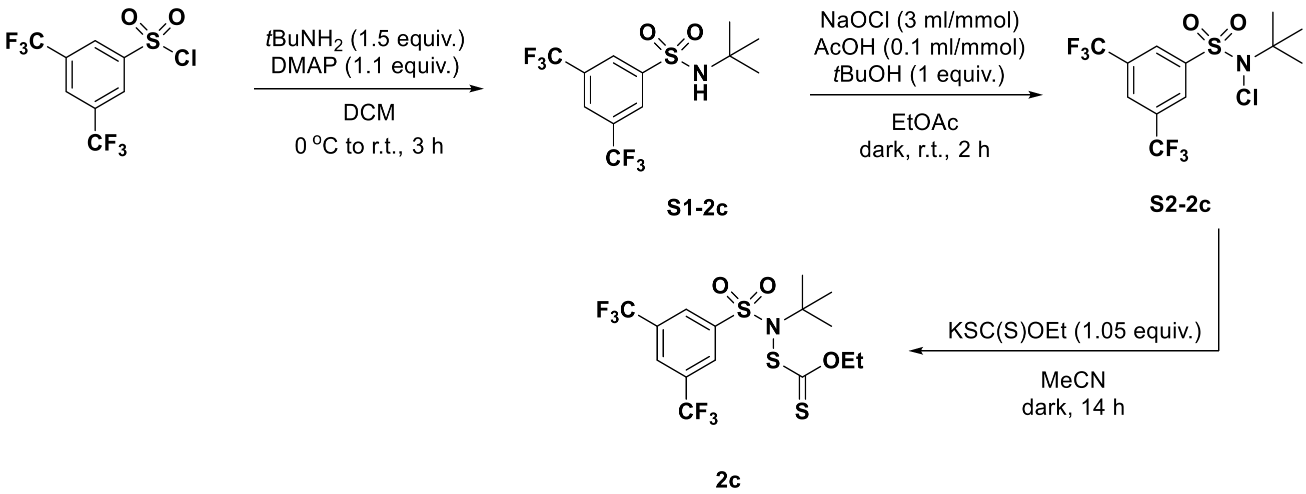

Following a modified procedure of the reported literature.[5]

***N*-(*tert*-butyl)-3,5-bis(trifluoromethyl)benzenesulfonamide (S1-2c)**

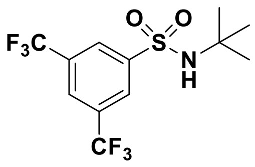

S1-2c

In a two-necked 250 ml round bottom flask with teflon-coated magnetic stir bar, 3,5-bis(trifluoromethyl)benzenesulfonyl chloride (6.24 g, 20 mmol, 1 equiv.) is dissolved in DCM (100 ml). The solution is cooled to 0 °C with an ice bath, followed by the addition of DMAP (1.72 g, 22 mmol, 1.1 equiv.) and *t*BuNH$_2$ (3.15 ml, 30 mmol, 1.5 equiv.). The mixture is warmed to room temperature and stirred until complete conversion of the starting material. The reaction mixture is diluted with DCM and the organic layer is washed with 1 M HCl, sat. aq. NaHCO$_3$ and brine, dried over Na$_2$SO$_4$, and concentrated *in vacuo* to afford the sulfonamide **S1-2c** as a white solid. The product was used for the next step without further purification.

***N*-(*tert*-butyl)-*N*-chloro-3,5-bis(trifluoromethyl)benzenesulfonamide (S2-2c)**

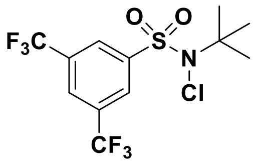

S2-2c

In a 250 ml flask with teflon-coated magnetic stir bar, covered with aluminum foil to minimise exposition to ambient light, sulfonamide **S1-2c** is dissolved in EtOAc (30 ml). In ambient atmosphere, NaOCl (6-14 % available chlorine, 60 ml, 3 ml/mmol), glacial AcOH (2 ml, 0.1 ml/mmol) and *t*BuOH (1.9 ml, 20 mmol, 1 equiv.) are added. The biphasic reaction mixture is vigorously stirred at room temperature for 2 h, after which it is quenched with sat. aq. NaHCO$_3$. The aqueous phase is extracted with EtOAc, and the combined organic phases are washed with brine, dried over Na$_2$SO$_4$, and concentrated *in vacuo* to afford the *N*-chlorosulfonamide **S2-2c** as a yellow oil. The product was used for the next step without further purification.

***N*-(tert-butyl)-*N*-((ethoxycarbonothioyl)thio)-3,5-bis(trifluoromethyl)benzenesulfonamide (2c)**

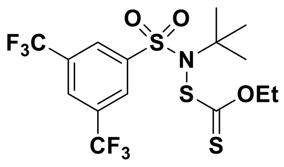

2c

In a three-necked 500 ml round bottom flask with teflon-coated magnetic stir bar, covered with aluminum foil to minimise exposition to ambient light, potassium ethyl xanthogenate (772.8 mg, 4.83 mmol, 1.05 equiv.) is suspended in MeCN (40 ml). To this suspension, a solution of *N*-chlorosulfonamide **S2-2c** in MeCN (15 ml) is added dropwise over 30 min *via* syringe pump. The mixture is stirred at room temperature overnight, after which it is concentrated *in vacuo*. The residue is

partitioned in DCM/H_2O (1:1, 250 ml total volume) and the layers are separated. The organic phase is washed with brine, dried with Na_2SO_4, and concentrated *in vacuo*. The crude product is then purified by silica gel chromatography (PE/Et_2O 99/1 to 97/3) to afford **2c** as a pale yellow solid (621 mg, 29%). Spectral data are coherent with those reported in the literature.[5]

^{1}H-NMR (400 MHz, $CDCl_3$) δ: 1.33 (t, *J* = 7.01 Hz, 3H, O-CH_2CH_3), 1.57 (s, 9H, N-C-(CH_3)$_3$), 4.26-4.34 (m, 1H, O-CH_2-CH_3), 4.61-4.69 (m, 1H, O-CH_2-CH_3), 8.06 (s, 1H, Ar-*H*), 8.36 (s, 2H, Ar-*H*).

S4.4. Synthesis of 2b

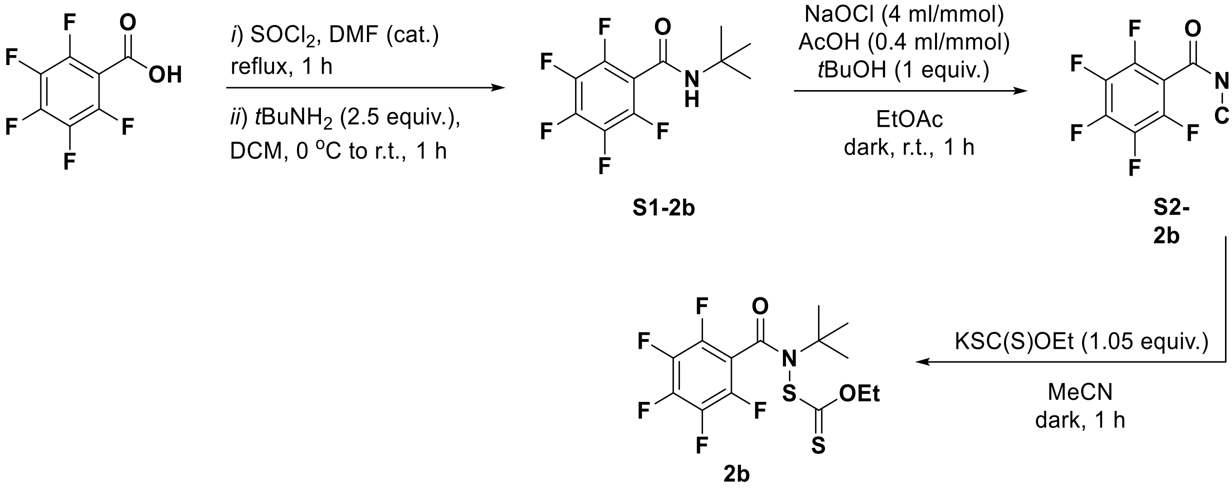

Compound **2b** was synthesized following a modified procedure of the reported literature[5]

N-(tert-butyl)-2,3,4,5,6-pentafluorobenzamide (S1-2b)

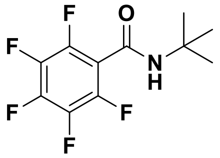

S1-2b

In a 100 ml flask, 2,3,4,5,6-pentafluorobenzoic acid (5.109 g, 24.1 mmol, 1 equiv.) is suspended in thionyl chloride (30 ml) and catalytic DMF is added (ca. 1%). The flask is then equipped with a condenser and the mixture is heated to 80 °C. After 1 h, the reaction is cooled to room temperature and the volatiles are carefully removed *in vacuo*. The resulting crude is then resuspended in DCM and cooled to 0 °C, followed by the addition of *t*BuNH_2 (12.6 ml, 120.5 mmol, 5 equiv.) dropwise. The reaction mixture is stirred while allowed to warm to room temperature until complete conversion of the starting material. The mixture is diluted with DCM, and the organic phases are washed with sat. aq. $NaHCO_3$, 1 M HCl and brine, dried over Na_2SO_4 and concentrated *in vacuo*. The residue is triturated with cold petroleum ether to afford amide **S1-2b** as a white solid. The product was used for the next step without further purification. Spectral data are coherent with those reported in the literature.[5]

^{1}H-NMR (400 MHz, $CDCl_3$) δ: 1.46 (s, 9H, N-C-(CH_3)$_3$), 5.64 (bs, 1H, N*H*).

N-(tert-butyl)-*N*-chloro-2,3,4,5,6-pentafluorobenzamide (S2-2b)

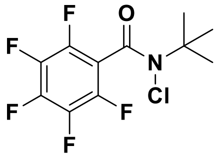

S2-2b

In a 250 ml flask with teflon-coated magnetic stir bar, covered with aluminum foil to minimise exposition to ambient light, amide **S1-2b** (2.136 g, 8 mmol, 1 eq.) is dissolved in EtOAc (30 ml). In ambient atmosphere, NaOCl (6-14 % available chlorine, 32 ml, 4 ml/mmol), glacial AcOH (2.72 ml, 0.34 ml/mmol) and *t*BuOH (0.756 ml, 8 mmol, 1 equiv.) are added. The biphasic reaction mixture is vigorously stirred at room temperature for 1

h, after which it is quenched with sat. aq. $NaHCO_3$. The aqueous phase is extracted with EtOAc, and the combined organic phases are washed with brine, dried over Na_2SO_4, and concentrated *in vacuo* to afford the *N*-chloronamide **S2-2b** as a pale yellow oil (2.253 g, 93%). The product was used for the next step without further purification.

N-(tert-butyl)-N-chloro-2,3,4,5,6-pentafluorobenzamide (2b)

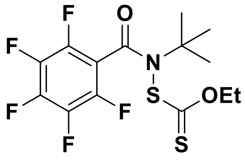

2b

In a three-necked 500 ml flask with teflon-coated magnetic stir bar, covered with aluminum foil to minimize exposition to ambient light, potassium ethyl xanthogenate (1.264 g, 7.9 mmol, 1.05 equiv.) is suspended in MeCN (175 ml). To this suspension, a solution of *N*-chloroamide **S2-2b** (2.2 g, 7.8 mmol, 1 eq.) in MeCN (20 ml) is added dropwise over 60 min *via* syringe pump. The mixture is stirred at room temperature for 1 h, after which it is concentrated *in vacuo*. The residue is partitioned in DCM/H_2O (1:1, 250 ml total volume) and the layers are separated. The organic phase is washed with brine, dried with Na_2SO_4, and concentrated *in vacuo*. The crude product is then purified by silica gel chromatography (PE/EtOAc 99/1 to 95/5) to afford **2b** as a pale yellow solid (1.211 g, 40%). Spectral data are coherent with those reported in the literature.[5]

^{1}H-NMR (400 MHz, $CDCl_3$) δ: 1.49 (t, J = 7.2 Hz, 3H, O-CH_2-CH_3), 4.58 (dq, J = 7.2, 10.7 Hz, 1H, O-CH_2-CH_3), 4.75 (dq, J = 7.2, 10.7 Hz, 1H, O-CH_2-CH_3).

S4.5. Synthesis of 2a

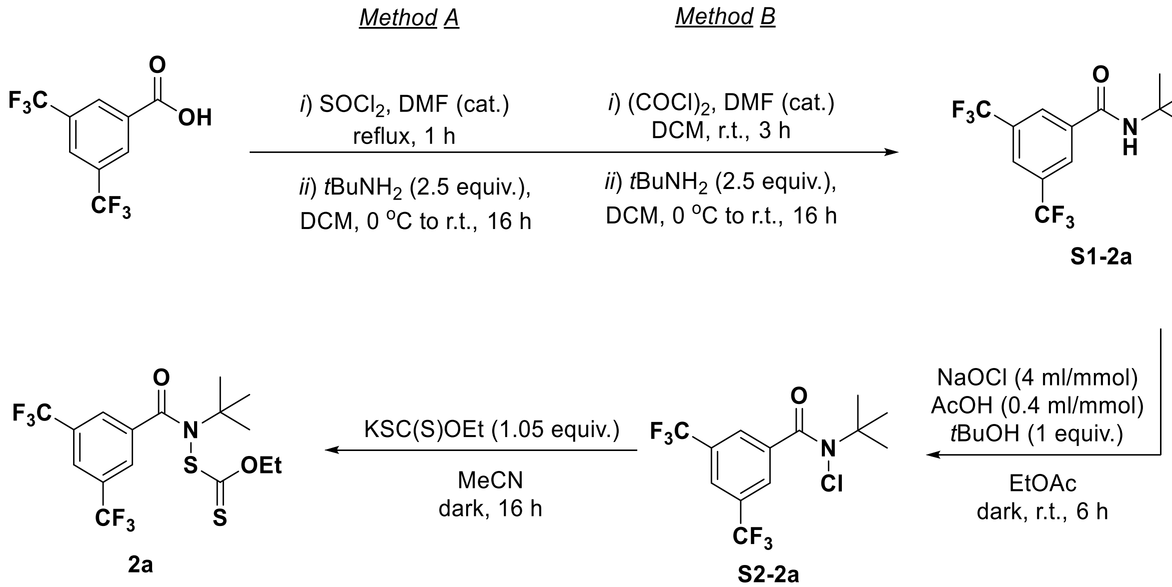

Compound **2a** was obtained following a modified procedure of the reported literature.[5]

N-(tert-butyl)-3,5-bis(trifluoromethyl)benzamide (S1-2a)

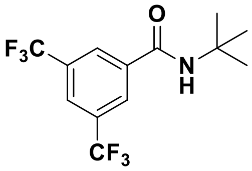

S1-2a

Method A: In a 100 ml flask, 3,5-bis(trifluoromethyl)benzoic acid (15.738 g, 61 mmol, 1 equiv.) is suspended in thionyl chloride (30 ml) and catalytic DMF is added (ca. 1%). The flask is then equipped with a condenser and the mixture is heated to 80˚C. After 1 h, the reaction is cooled to room temperature and the volatiles are carefully removed *in vacuo*. The resulting crude is then resuspended in DCM (200 ml) and cooled to 0 °C, followed by the addition of $tBuNH_2$ (13 ml, 125 mmol, 2 equiv.) dropwise. The reaction mixture is stirred

while allowed to warm to room temperature until complete conversion of the starting material. The mixture is diluted in DCM, and the organic layers are washed with sat. aq. $NaHCO_3$, 1 M HCl and brine, dried over Na_2SO_4 and concentrated *in vacuo*. The residue is triturated with cold petroleum ether to afford amide **S1-2a** as a white solid. The product was used for the next step without further purification.

Method B: In a 100 ml flask, 3,5-bis(trifluoromethyl)benzoic acid (2.581 mg, 10 mmol, 1 equiv.) is dissolved in DCM (40 ml). Catalytic DMF (ca. 1%) is added, followed by the dropwise addition of oxalyl chloride (1.69 ml, 20 mmol, 2 equiv.). The reaction mixture is stirred at room temperature for 3 h, after which the volatiles are carefully removed *in vacuo*. The resulting crude is then resuspended in DCM and cooled to 0 °C, followed by the addition of *t*BuNH$_2$ (2 ml, 20 mmol, 2 equiv.) dropwise. The reaction mixture is stirred while allowed to warm to room temperature until complete conversion of the starting material. The mixture is diluted in DCM, and the organic layers are washed with sat. aq. $NaHCO_3$, 1 M HCl and brine, dried over Na_2SO_4 and concentrated *in vacuo*. The residue is triturated with cold petroleum ether to afford amide **S1-2a** as a white solid. The product was used for the next step without further purification. Spectral data are coherent with those reported in the literature:[5]

^{1}H-NMR (400 MHz, $CDCl_3$) δ: 1.51 (s, 9H, N-C-(C*H*$_3$)$_3$), 5.96 (bs, 1H, N-*H*), 7.98 (s, 1H, Ar-*H*), 8.16 (s, 2H, Ar-*H*).

***N*-(tert-butyl)-*N*-chloro-3,5-bis(trifluoromethyl)benzamide (S2-2a)**

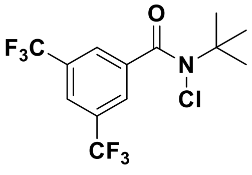

S2-2a

In a 250 ml flask with teflon-coated magnetic stir bar, covered with aluminum foil to minimise exposition to ambient light, amide **S1-2a** (3.4866 g, 11.1 mmol, 1 equiv.) is dissolved in EtOAc (20 ml). In ambient atmosphere, NaOCl (6-14 % available chlorine, 60.5 ml, 5.4 ml/mmol), glacial AcOH (6.05 ml, 0.54 ml/mmol) and *t*BuOH (1 ml, 11.1 mmol, 1 equiv.) are added. The biphasic reaction mixture is vigorously stirred at room temperature for 6 h, after which it is quenched with sat. aq. $NaHCO_3$. The aqueous phase is extracted with EtOAc, and the combined organic phases are washed with brine, dried over Na_2SO_4, and concentrated *in vacuo*. The crude product is then purified by silica gel chromatography (PE/DCM 95/5) to afford **S2-2a** as a colorless oil (3.778 g, 95%). Spectral data are coherent with those reported in the literature.[5]

^{1}H-NMR (400 MHz, $CDCl_3$) δ: 1.60 (s, 9H, N-C-(C*H*$_3$)$_3$), 7.95 (s, 1H, Ar-*H*), 8.06 (s, 2H, Ar-*H*).

***N*-(tert-butyl)-*N*-chloro-3,5-bis(trifluoromethyl)benzamide (2a)**

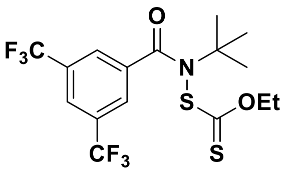

2a

In a three-necked 500 ml flask with teflon-coated magnetic stir bar, covered with aluminum foil to minimize exposition to ambient light, potassium ethyl xanthogenate (663 g, 4.1 mmol, 1 equiv.) is suspended in MeCN (120 ml). To this suspension, a solution of *N*-chloroamide **S2-2a** (1.369 g, 3.9 mmol, 1 equiv.) in MeCN (15 ml) is added dropwise over 70 min *via* syringe pump. The mixture is stirred at room temperature for 16 h, after which it is concentrated *in vacuo*. The residue is partitioned in DCM/H_2O (1:1, 250 ml total volume) and the layers are separated. The organic phase is washed with brine, dried with Na_2SO_4, and concentrated *in vacuo*. The crude product is then purified by silica gel chromatography (PE/Et_2O 100/0 to 97/3) to afford **2a** as a yellow solid (549 mg, 32%). Spectral data are coherent with those reported in the literature.[5]

^{1}**H-NMR** (400 MHz, $CDCl_3$) δ: 1.50 (t, *J* = 6.97 Hz, 3H, O-CH_2-C*H*$_3$), 1.59 (s, 9H, N-C-(C*H*$_3$)$_3$), 4.60-4.73 (m, 2H, O-C*H*$_2$-CH_3), 7.86 (s, 2H, Ar-*H*), 7.88 (s, 1H, Ar-*H*).

S4.6. General procedure for the synthesis of carboranes 1c-j

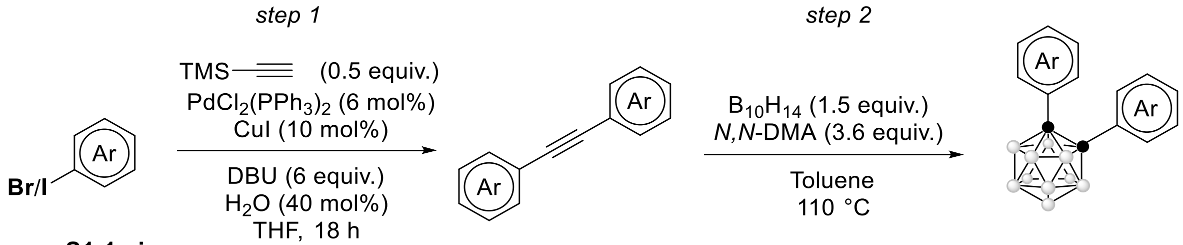

S1-1c-j **S2-1c-j**

Ar = C_6H_5, **1c**
Ar = 4-F-C_6H_4, **1d**
Ar = 4-Cl-C_6H_4, **1e**
Ar = 4-Br-C_6H_4, **1f**
Ar = 4-CF_3-C_6H_4, **1g**
Ar = 3-CF_3-C_6H_4, **1h**
Ar = 2-thiophen, **1i**
Ar = 2-naphtyl, **1j**

Step 1: Following a modified procedure of the reported literature,[6] a 50 ml Schlenk tube with teflon-coated magnetic stir bar fitted with a rubber septum and flame dried under vacuum with a heat gun is charged with $PdCl_2(PPh_3)_2$ (6 mol%), CuI (10 mol%) and (if solid) starting material halide (1 equiv,). The reaction vessel is evacuated again under vacuum and purged with N_2. While stirring, dry toluene (0.2 M wrt to halide) sparged with dry N_2 is added by syringe. N_2-sparged DBU (6 equiv.) is then added, followed by (if liquid) starting material halide (1 equiv.), trimethylsilylacetylene (0.5 equiv.) and distilled water (40 mol%). The rubber septum is then parafilmed and the reaction is stirred at a high rate of speed under a N_2 atmosphere at set temperature (specified for each substrate). After 18 h, the reaction mixture is partitioned in diethyl ether and distilled water (in a 1:1 ratio). The organic layer is washed with 10% HCl and brine, dried over Na_2SO_4 and the solvent is removed *in vacuo*. The crude product is then purified by silica gel chromatography using the specified combination of solvents.

1,2-Bis(4-fluorophenyl)ethyne (S2-1d)

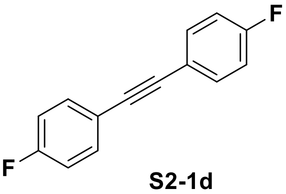

S2-1d

Following the described procedure, 4-fluoroiodobenzene (0.46 ml, 4 mmol, 1 equiv.) was reacted at 60 °C to afford **S2-1d** (470 mg, 55%) as a yellow solid after flash silica gel chromatography (PE). Spectral data are coherent with those reported in literature.[6]

^{1}**H-NMR** (400 MHz, $CDCl_3$) δ: 7.02-7.07 (m, 4H, Ar-*H*), 7.48-7.51 (m, 4H, Ar-*H*).

1,2-Bis(4-chlorophenyl)ethyne (S2-1e)

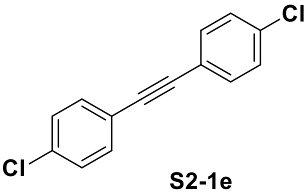

S2-1e

Following the described procedure, 4-chloroiodobenzene (952 mg, 4mmol, 1 equiv.) was reacted at room temperature to afford **S2-1e** (98 mg, 20%) as a yellow solid after flash silica gel chromatography (PE). Spectral data are coherent with those reported in literature.[6]

^{1}**H-NMR** (400 MHz, $CDCl_3$) δ: 7.33 (d, 4H, *J* = 8.50 Hz, Ar-*H*), 7.45 (d, 4H, *J* = 8.64 Hz, Ar-*H*).

1,2-Bis(4-bromophenyl)ethyne (S2-1f)

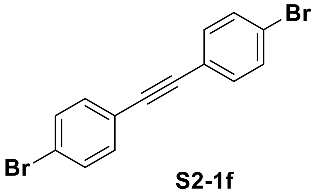

S2-1f

Following the described procedure, 4-bromoiodobenzene (1.1316 g, 4 mmol, 1 equiv.) was reacted at room temperature to afford **S2-1f** (225 mg, 34%) as a white solid after flash silica gel chromatography (PE). Spectral data are coherent with those reported in literature.[6]

^{1}H-NMR (400 MHz, $CDCl_3$) δ: 7.00-7.02 (m, 4H, Ar-*H*), 7.08-7.12 (m, 4H, Ar-*H*).

1,2-Bis(4-(trifluoromethyl)phenyl)ethyne (S2-1g)

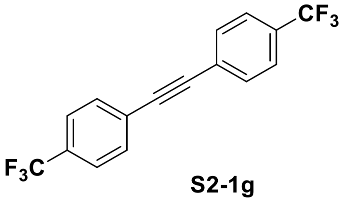

S2-1g

Following the described procedure, 4-iodobenzotrifluoride (0.440 ml, 3 mmol, 1 equiv.) was reacted at room temperature to afford **S2-1g** (366 mg, 77%) as a yellow solid after flash silica gel chromatography (PE). Spectral data are coherent with those reported in literature.[6]

^{1}H-NMR (400 MHz, $CDCl_3$) δ: 7.62-7.66 (m, 8H, Ar-*H*).

1,2-Bis(3-(trifluoromethyl)phenyl)ethyne (S2-1h)

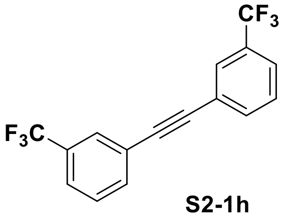

S2-1h

Following the described procedure, 3-iodobenzotrifluoride (0.553 ml, 4 mmol, 1 equiv.) was reacted at room temperature to afford **S2-1h** (509 mg, 81%) as a yellow solid after flash silica gel chromatography (PE/EtOAc 95:5). Spectral data are coherent with those reported in literature.[7]

^{1}H-NMR (400 MHz, $CDCl_3$) δ: 7.48-7.52 (m, 2H, Ar-*H*), 7.61-7.63 (m, 2H, Ar-*H*), 7.70-7.72 (m, 2H, Ar-*H*), 7.79-7.81 (m, 2H, Ar-*H*).

1,2-Di(thiophen-2-yl)ethyne (S2-1i)

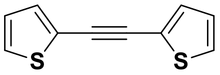

S2-1i

Following the described procedure, 2-bromothiophene (0.4 ml, 4 mmol, 1 equiv.) was reacted at 60 °C to afford **S2-1i** (307 mg, 80%) as a white solid after flash silica gel chromatography (PE). Spectral data are coherent with those reported in literature.[6]

^{1}H-NMR (400 MHz, $CDCl_3$) δ: 7.01 (dd, *J* = 3.69, 5.12 Hz, 2H, Ar-*H*), 7.28 (dd, *J* = 1.28, 3.76 Hz, 2H, Ar-*H*), 7.31 (dd, *J* = 1.12, 5.12 Hz, 2H, Ar-*H*).

1,2-bis(2-naphtyl)ethyne (S2-1j)

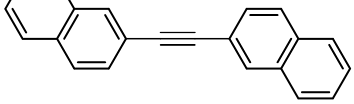

S2-1j

Following the described procedure, **S1-1j** (414 mg, 2 mmol, 1 equiv.) was reacted at 60 °C to afford **S2-1j** (83 mg, 15%) as a yellow solid after flash silica gel chromatography (PE). Spectral data are coherent with those reported in literature.[8]

^{1}H-NMR (400 MHz, $CDCl_3$) δ: 7.47-7.54 (m, 4H; Ar-*H*), 7.62 (dd, 2H, *J* = 8.5, 1.8 Hz; Ar-*H*), 7.81-7.87 (m, 6H; Ar-*H*), 8.11 (s, 2H; Ar-*H*)

Step 2: A heavy-wall Pyrex tube with teflon-coated magnetic stir bar fitted with a rubber septum and flame dried under vacuum with a heat gun is charged with alkyne (1 equiv.) and decaborane (1.5 equiv.). The reaction vessel is evacuated again under vacuum and purged with N_2. Then, dry toluene (0.2 M) and *N,N*-

dimethylaniline (3.6 equiv.) are added via syringe and the rubber septum is rapidly replaced by a screwing cap with a teflon seal. The reaction mixture is stirred at 110 °C (***Caution:*** *development of gaseous* H_2) until complete consumption of the starting material for 1-16 h. After cooling to room temperature, the volatiles are removed *in vacuo*. The crude product is then purified by silica gel chromatography using the specified combination of solvents.

1,2-Diphenyl-*o*-carborane (1c)

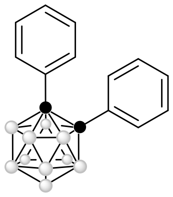

1c

Following the described procedure, 1,2-diphenylethyne (356 mg, 2 mmol, 1 equiv.) was reacted to afford **1c** (357 mg, 60%) as a white solid after flash silica gel chromatography (PE). Spectral data are coherent with those reported in literature.[9]

^{1}H-NMR (400 MHz, $CDCl_3$) δ: 1.75-3.41 (m, 10H, carborane B-*H*), 7.11-7.16 (m, 4H, Ar-*H*), 7.20-7.25 (m, 2H, Ar-*H*), 7.41-7.44 (n, 4H, Ar-*H*).

1,2-Bis(4-fluorophenyl)-*o*-carborane (1d)

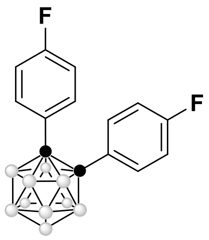

1d

Following the described procedure, 1,2-bis(4-fluorophenyl)ethyne **S2-1d** (150 mg, 0.7 mmol, 1 equiv.) was reacted to afford **1d** (207 mg, 89%) as a white solid after flash silica gel chromatography (PE). Spectral data are coherent with those reported in literature.[9]

^{1}H-NMR (400 MHz, $CDCl_3$) δ: 1.67-3.21 (m, 10H, carborane B-*H*), 6.82-6.87 (m, 4H, Ar-*H*), 7.39-7.43 (m, 4H, Ar-*H*).

1,2-Bis(4-chlorophenyl)-*o*-carborane (1e)

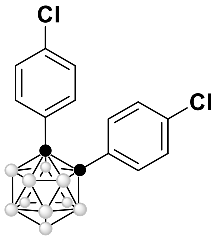

1e

Following the described procedure, 1,2-bis(4-chlorophenyl)ethyne **S2-1e** (98 mg, 0.397 mmol, 1 equiv.) was reacted to afford **1e** (80 mg, 55%) as a white solid after flash silica gel chromatography (PE). Spectral data are coherent with those reported in literature.[9]

^{1}H-NMR (400 MHz, $CDCl_3$) δ: 1.67-3.67 (m, 10H, carborane B-*H*), 7.13-7.17 (m, 4H, Ar-*H*), 7.34-7.37 (m, 4H, Ar-*H*).

1,2-Bis(4-bromophenyl)-*o*-carborane (1f)

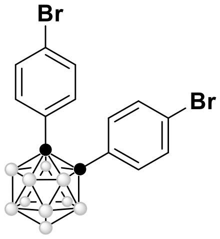

1f

Following the described procedure, 1,2-bis(4-bromophenyl)ethyne **S2-1f** (225 mg, 0.67 mmol, 1 equiv.) was reacted to afford **1f** (268 mg, 88%) as a white solid after flash silica gel chromatography (PE). Spectral data are coherent with those reported in literature.[10]

^{1}H-NMR (400 MHz, $CDCl_3$) δ: 1.61-3.54 (m, 10H, carborane B-*H*), 7.26-7.32 (m, 8H, Ar-*H*).

1,2-Bis((4-trifluoromethyl)phenyl)-*o*-carborane (1g)

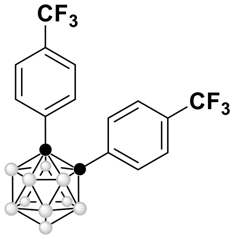

1g

Following the described procedure, 1,2-bis(4-(trifluoromethyl)phenyl)ethyne **S2-1g** (314 mg, 1 mmol, 1 equiv.) was reacted to afford **1g** (398 mg, 92%) as a white solid after flash silica gel chromatography (PE). Spectral data are coherent with those reported in literature.[9]

^{1}H-NMR (600 MHz, $CDCl_3$) δ: 1.91-3.89 (m, 10H, carborane B-*H*), 7.44 (d, J = 8.34 Hz, 4H, Ar-*H*), 7.57 (d, J = 8.28 Hz, 4H, Ar-*H*).

1,2-Bis((3-trifluoromethyl)phenyl)-*o*-carborane (1h)

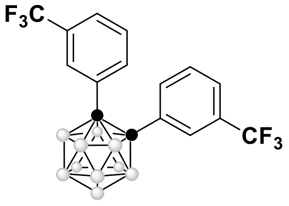

1h

Following the described procedure, 1,2-bis(3-(trifluoromethyl)phenyl)ethyne **S2-1h** (314 mg, 1 mmol, 1 equiv.) was reacted to afford **1h** (398 mg, 92%) as a white solid after flash silica gel chromatography (PE).

^{1}H-NMR (400 MHz, $CDCl_3$) δ: 1.63-3.50 (m, 10H, carborane B-*H*), 7.33 (t, *J* = 7.88 Hz, 2H, Ar-*H*), 7.52 (d, *J* = 7.84 Hz, 2H, Ar-*H*), 7.85 (s, 2H, Ar-*H*), 7.64 (d, *J* = 8.04 Hz, 2H, Ar-*H*).

^{13}C{^{1}H}-NMR (100 MHz, $CDCl_3$) δ: 83.6 (C_q), 121.9 (C_q), 124.6 (C_q), 127.2-127.4 (m, CH), 129.4 (CH), 131.3 (q, *J* = 32.9 Hz, C_q), 131.4 (C_q), 134.0 (CH).

^{11}B{^{1}H}-NMR (128 MHz, $CDCl_3$) δ: -11.3, -9.8, -9.0, -1.7.

^{19}F{^{1}H}-NMR (376 MHz, $CDCl_3$) δ: -63.2.

HRMS (ESI) m/z: $[M + H]^+$ Calcd for $C_{16}H_{19}B_{10}F_6$ 435.2321

IR (thin film, cm-1) ν: 2923, 2595, 1488, 1438, 1326, 1126, 747, 693.

R_f: 0.10 (PE/Et_2O 99/1).

1,2-(2,2'-Dithiophenyl)-*o*-carborane (1i)

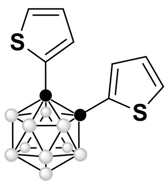

1i

Following the described procedure, 1,2-di(thiophen-2-yl)ethyne **S2-1i** (190.3 mg, 1 mmol, 1 equiv.) was reacted to afford **1i** (198 mg, 60%) as a yellow solid after flash silica gel chromatography (PE). Spectral data are coherent with those reported in literature.[11]

^{1}H-NMR (400 MHz, $CDCl_3$) δ: 1.59-3.33 (m, 10H, carborane B-*H*),6.81 (dd, *J* = 3.77, 5.19 Hz, 2H, Ar-*H*), 7.16-7.19 (m, 4H, Ar-*H*).

1,2-(2,2'-Napthyl)-*o*-carborane (1j)

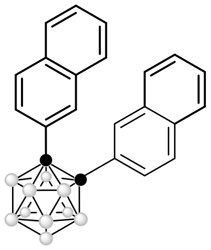

1j

Following the described procedure, 1,2-bis(2-naphtyl)ethyne **S2-1j** (210 ml, 0.5 mmol, 1 equiv.) was to afford **1j** (29 mg, 21%) as a bright yellow solid after flash silica gel chromatography (PE). Spectral data are coherent with those reported in literature.[12]

^{1}H-NMR (400 MHz, $CDCl_3$) δ: 1.90-3.80 (m, 10H, carborane B-*H*), 7.36-7.46 (m, 4H; Ar-*H*), 7.54 (sb, 4H; Ar-*H*), 7.64 (dd, 2H, *J*= 9.5, 4.6 Hz; Ar-*H*), 7.70 (dd, 2H, *J*= 9.6, 6.0 Hz; Ar-*H*), 8.01 (s, 2H; Ar-*H*).

1-Phenyl-*o*-carborane (1k)

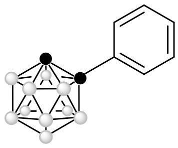

1k

Following a modified version of the described procedure, phenylacetylene (0.17 ml, 1.56 mmol, 2.6 equiv.) was reacted with decaborane (73 mg, 0.6 mmol, 1 equiv.) and bmimCl (50 mg, 0.3 mmol, 0.48 equiv.) to afford **1k** (29 mg, 21%) as a white solid after flash silica gel chromatography (PE). Spectral data are coherent with those reported in literature.[9]

^{1}H-NMR (400 MHz, $CDCl_3$) δ: 1.60-3.23 (m, 10H, carborane B-*H*), 3.97 (bs, 1H, carborane-C*H*), 7.31-7.36 (m, 2H, Ar-*H*), 7.38-7.42 (m, 1H, Ar-*H*), 7.49 (d, *J* = 7.25 Hz, 2H)

S4.7. Synthesis of carborane 1l

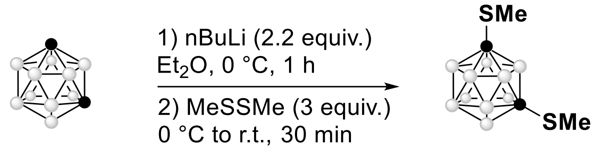

1l

1,7-Bis(methylsulfanyl)-*m*-carborane (1l)

Following a modified procedure of the reported literature:[13] in a 25 ml round bottom flask, m-carborane (115 mg, 0.8 mmol, 1 equiv.) is dissolved in diethyl ether (4 ml). The solution is cooled to 0 °C in an ice bath and nBuLi (1.6 M in Hexane, 0.067 ml, 2.1 equiv.) is added dropwise. After stirring at this temperature for 1 h, dimethyl disulfide (0.15 ml, 1.68 mmol, 2.1 equiv.) is added dropwise and the suspension is left to warm to room temperature in 30 min. The reaction mixture is quenched with water, extracted with DCM three times, washed with brine, dried over Na_2SO_4 and concentrated *in vacuo*. The crude product is then purified by silica gel chromatography (PE) to afford **1l** (46 mg, 24%) as a white solid. Spectral data are coherent with those reported in the literature.[13]

^{1}H-NMR (400 MHz, $CDCl_3$) δ: 1.56-3.30 (m, 10H, carborane B-*H*), 2.28 (s, 6H, CH_3).

S4.8. Synthesis of carboranes 1n-o

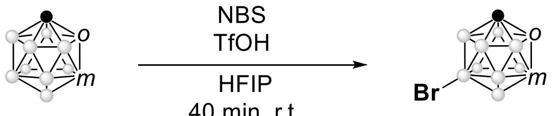

1n-o

9-Bromo-*o*-carborane (1n)

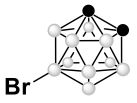

MR351

Following a modified procedure of the reported literature:[14] in a 100 ml round bottom flask, *o*-carborane (720 mg, 5 mmol, 1 equiv.) and NBS (979 mg, 5.5 mmol, 1.5 equiv.) are dissolved in HFIP (50 ml). Trifluoromethanesulfonic acid (0.22 ml, 50 mol%) is carefully added dropwise and the reaction system is stirred at room temperature for 40 min. After completion of the reaction, the volatiles are removed *in vacuo* and the crude is redissolved in a small amount of DCM (ca. 3 ml). Distilled water is added and the precipitate is collected by filtration on a sintered glass filter, washed with distilled water and cold diethyl ether. The product is obtained as a white solid (960 mg, 86%) and used without further purifications. Spectral data are coherent with those reported in the literature.[14]

^{1}H-NMR (400 MHz, $CDCl_3$) δ: 1.58-3.23 (m, 10H, carborane B-*H*), 3.60 (bs, 2H, carborane-C*H*).

9-Bromo-*m*-carborane (1o)

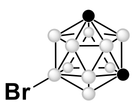

MR633

Following a modified procedure of the reported literature:[14] in a 50 ml round bottom flask, *m*-carborane (288 mg, 1 mmol, 1 equiv.) and NBS (359 mg, 2.02 mmol, 1.01 equiv.) are dissolved in HFIP (20 ml). Trifluoromethanesulfonic acid (0.01 ml, 10 mol%) is carefully added dropwise and the reaction system is stirred at room temperature for 40 min. After completion of the reaction, the volatiles are removed *in vacuo* and the crude is redissolved in a small amount of DCM (ca. 3 ml). Distilled water is added and the precipitate is collected by filtration on a sintered glass filter, washed with distilled water and cold diethyl ether. The product is obtained as a white solid (161 mg, 73%) and used without further purifications. Spectral data are coherent with those reported in the literature.[14]

^{1}H-NMR (400 MHz, $CDCl_3$) δ: 1.69-3.61 (m, 10H, carborane B-*H*), 2.99 (bs, 2H, carborane-C*H*).

S4.9. General procedure for the synthesis of xanthyl carboranes 3a-o

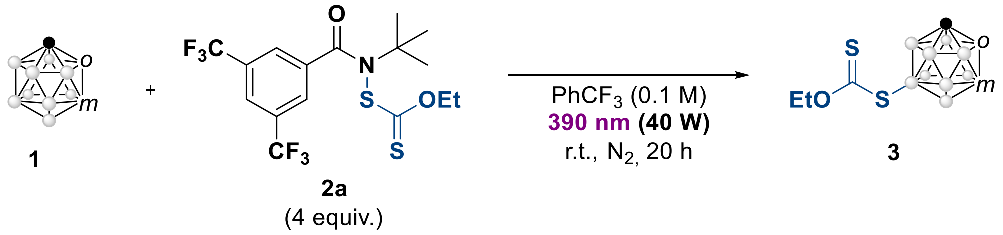

A 4 ml scintillation vial with a teflon-coated magnetic stir bar is charged with carborane **1a-o** and xanthylamide **2a** (4 equiv.). The vial is sealed with a rubber septum, evacuated under vacuum and purged with N_2 three times. Previously distilled and degassed $PhCF_3$ (0.1 M) is added *via* syringe. The reaction mixture is left to stir under the irradiation of a Kessil® LED lamp (390 nm, 40 W) for 20 h, after which it is concentrated *in vacuo*. The crude product is then purified by silica gel chromatography using the specified combination of solvents.

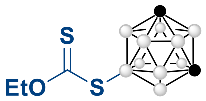

3a

Following the described procedure, carborane **1a (**21.6 mg, 0.15 mmol, 1 equiv.) was reacted with *N*-xanthylamide **2a** (260 mg, 0.60 mmol, 4 equiv.) to afford **3a** (25 mg, 64 %) as a pale yellow solid after silica gel chromatography (PE/Et_2O 99/1).

^{1}H-NMR (400 MHz, $CDCl_3$) δ: 1.45 (t, *J* = 7.13 Hz, 3H, CH_3-CH_2), 1.78-3.40 (m, 9H, carborane B-*H*), 3.00 (bs, 2H, carborane-C*H*), 4.63 (q, *J* = 7.13 Hz, 2H, O-CH_2-CH_3).

^{13}C{^{1}H}-NMR (100 MHz, $CDCl_3$) δ: 13.6 (CH_3), 54.4 (CH), 70.6 (CH_2), 212.0 (C_q).

^{11}B{^{1}H}-NMR (128 MHz, $CDCl_3$) δ: -18.5, -17.3, -13.7, -13.1, -10.0, -5.9, -3.2.

HRMS (ESI) m/z: $[M + H]^+$ Calcd for $C_5H_{17}B_{10}OS_2$ 267.1651. Found 267.1646

IR (thin film, cm-1) ν: 3042, 2607, 1228, 1111, 1043.

m.p.: 49.1 – 50.0 °C.

R_f: 0.10 (PE/Et_2O 99/1).

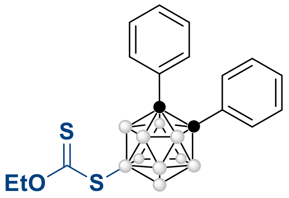

3c

Following the described procedure, carborane **1c** (59 mg, 0.2 mmol, 1 equiv.) was reacted with *N*-xanthylamide **2a** (346 mg, 0.80 mmol, 4 equiv.) to afford **1c** (45 mg, 54%) as a yellow solid after silica gel chromatography (PE/Et_2O 99/1).

^{1}H-NMR (400 MHz, $CDCl_3$) δ: 1.48 (t, *J* = 7.13 Hz, 3H, CH_3-CH_2), 2.00-3.22 (m, 9H, carborane B-*H*), 4.67 (q, *J* = 7.12 Hz, 2H, O-CH_2-CH_3), 7.14-7.18 (m, 4H, Ar-*H*), 7.24-7.28 (m, 4H, Ar-*H* + residual $CHCl_3$), 7.41-7.44 (m, 4H, Ar-*H*).

^{13}C{^{1}H}-NMR (100 MHz, $CDCl_3$) δ: 13.7 (CH_3), 70.5 (CH_2), 84.5 (C_q), 128.6 (CH), 130.0 (C_q), 130.7 (CH), 130.8 (CH), 212.4 (C_q).

^{11}B{^{1}H}-NMR (128 MHz, $CDCl_3$) δ: -11.0, -9.4, -3.1, -1.7, 4.2.

HRMS (ESI) m/z: $[M + H]^+$ Calcd for $C_{17}H_{25}B_{10}OS_2$ 419.2277. Found: 419.2272

IR (thin film, cm-1) ν: 3062, 2595, 1414, 1219, 1109, 520.

m.p.: 149.3 – 151.1 °C.

R_f: 0.12 (PE/Et_2O 99/1).

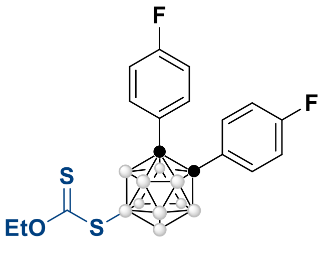

3d

Following the described procedure, carborane **1d** (67 mg, 0.2 mmol, 1 equiv.) was reacted with *N*-xanthylamide **2a** (346 mg, 0.8 mmol, 4 equiv.) to afford **3d** (30 mg, 33%) as a yellow solid after silica gel chromatography (PE/Et_2O 99/1).

^{1}H-NMR (400 MHz, $CDCl_3$) δ: 1.35 (t, *J* = 7.13 Hz, 3H, CH_3-CH_2), 1.86-3.38 (m, 9H, carborane B-*H*), 4.53 (q, *J* = 7.12 Hz, 2H, O-CH_2-CH_3), 6.78-6.83 (m, 4H, Ar-*H*), 7.30-7.35 (m, 4H, Ar-*H*).

^{13}C{^{1}H}-NMR (100 MHz, $CDCl_3$) δ: 13.5 (CH_3), 70.3 (CH_2), 81.4 (C_q), 83.3 (C_q), 115.8 (d, *J* = 10.3 Hz, CH), 116.0 (d, *J* = 9.6 Hz, CH), 125.6 (C_q), 126.1 (C_q), 132.7-132.9 (m, CH), 162.8 (C_q), 165.3 (C_q), 212.1 (C_q)

^{11}B{^{1}H}-NMR (128 MHz, $CDCl_3$) δ: -11.8, -9.9, -1.7, 4.4.

^{19}F{^{1}H}-NMR (376 MHz, $CDCl_3$) δ: 108.8, -108.7.

HRMS (ESI) m/z: $[M + H]^+$ Calcd for $C_{17}H_{23}B_{10}F_2OS_2$ 455.2089. Found: 455.2086.

IR (thin film, cm-1) ν: 2932, 2597, 1601, 1508, 1232, 1165, 1041, 840, 555.

m.p.: 153.8 – 154.0 °C.

R_f: 0.10 (PE/Et_2O 99/1).

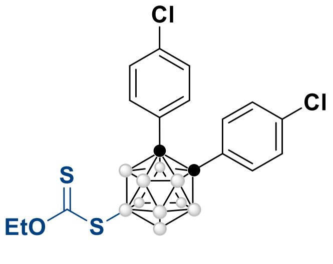

3e

Following the described procedure, carborane **1e** (64 mg, 0.17 mmol, 1 equiv.) was reacted with *N*-xanthylamide **2a** (346 mg, 0.8 mmol, 4 equiv.) to afford **1e** (51 mg, 62%) as a yellow solid after silica gel chromatography (PE/Et_2O 99/1).

^{1}H-NMR (400 MHz, $CDCl_3$) δ: 1.41 (t, *J* = 7.11 Hz, 3H, CH_3-CH_2), 1.91-3.34 (m, 9H, carborane B-*H*), 4.60 (q, *J* = 7.11 Hz, 2H, O-CH_2-CH_3), 7.15-7.18 (m, 4H, Ar-*H*), 7.31-7.35 (m, 4H, Ar-*H*).

^{13}C{^{1}H}-NMR (100 MHz, $CDCl_3$) δ: 13.6 (CH_3), 70.4 (CH_2), 81.1 (C_q), 83.1 (C_q), 128.1 (C_q), 128.5 (C_q), 131.9 (CH), 132.0 (CH), 137.6 (C_q), 137.6 (C_q), 212.0 (C_q).

^{11}B{^{1}H}-NMR (128 MHz, $CDCl_3$) δ: -11.9, -9.7, -1.6, 4.5.

HRMS (ESI) m/z: $[M + H]^+$ Calcd for $C_{17}H_{23}B_{10}Cl_2OS_2$ 487.1498. Found: 487.1491.

IR (thin film, cm-1) ν: 2923, 2599, 1492, 1278, 1229, 1043, 829, 428.

m.p.: 149.8 – 151.6 °C.

R_f: 0.10 (PE/Et_2O 99/1).

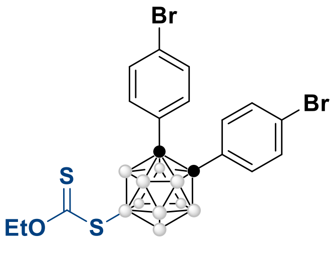

3f

Following the described procedure, carborane **1f** (90.8 mg, 0.2 mmol, 1 equiv.) was reacted with *N*-xanthylamide **2a** (346 mg, 0.8 mmol, 4 equiv.) to afford **3f** (46 mg, 40 %) as a yellow solid after silica gel chromatography (PE/Et_2O 99/1).

^{1}H-NMR (400 MHz, $CDCl_3$) δ: 1.41 (t, *J* = 7.13 Hz, 3H, CH_3-CH_2), 2.01-3.66 (m, 9H, carborane B-*H*), 4.60 (q, *J* = 7.13 Hz, 2H, O-CH_2-CH_3), 7.24-7.28 (m, 4H, Ar-*H* + residual $CHCl_3$), 7.32-7.35 (m, 4H, Ar-*H*).

^{13}C{^{1}H}-NMR (100 MHz, $CDCl_3$) δ: 13.6 (CH_3), 70.4 (CH_2), 81.1 (C_q), 83.0 (C_q), 126.0 (C_q), 126.0 (C_q), 128.7 (C_q), 129.0 (C_q), 132.0 (CH), 132.1 (CH), 132.1 (CH), 132.2 (CH), 212.0 (C_q).

^{11}B{^{1}H}-NMR (128 MHz, $CDCl_3$) δ: -12.0, -9.9, -1.6, 4.5.

HRMS (ESI) m/z: $[M + H]^+$ Calcd for $C_{17}H_{23}B_{10}Br_2OS_2$ 548.0487. Found: 548.0484.

IR (thin film, cm-1) ν: 2928, 2595, 1494, 1220, 1031, 833.

m.p.: 142.1 – 143.9 °C.
R_f: 0.10 (PE/Et_2O 99/1).

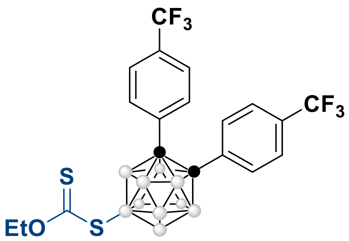

3g

Following the described procedure, carborane **1g** (87 mg, 0.2 mmol, 1 equiv.) was reacted with *N*-xanthylamide **2a** (4 equiv., 0.8 mmol, 346 mg) to afford **3g** (36 mg, 33%) as a yellow solid after silica gel chromatography (PE/Et_2O 99/1).
^{1}H-NMR (600 MHz, $CDCl_3$) δ: 1.42 (t, *J* = 7.02 Hz, 3H, CH_3-C*H*$_2$), 1.96-3.58 (m, 9H, carborane B-*H*), 4.60 (q, *J* = 7.20 Hz, 2H, O-C*H*$_2$-CH_3), 7.45-7.48 (m, 4H, Ar-*H*), 7.54-7.57 (m, 4H, Ar-*H*).
^{13}C{^{1}H}-NMR (150 MHz, $CDCl_3$) δ: 13.5 (CH_3), 70.4 (CH_2), 80.3 (C_q), 82.2 (C_q), 123.2 (q, *J* = 180.3 Hz, C_q), 125.9 (q, *J* =2.7 Hz, CH), 131.0 (CH), 131 (CH), 133.0 (C_q), 133.3 (C_q), 211.6 (C_q).
^{11}B{^{1}H}-NMR (192 MHz, $CDCl_3$) δ: -12.7, -10.5, -2.13, 3.92.
^{19}F{^{1}H}-NMR (564 MHz, $CDCl_3$) δ: 63.2.
HRMS (ESI) m/z: $[M + H]^+$ Calcd for $C_{19}H_{23}B_{10}F_6OS_2$ 555.2025. Found: 555.2018
IR (thin film, cm-1) v: 2925, 2602, 1617, 1409, 1322, 1229, 1170, 1062, 904, 840.
m.p.: 115.8 – 116.9 °C.
R_f: 0.10 (PE/Et_2O 99/1).

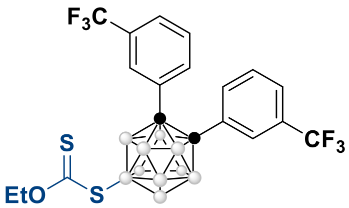

3h

Following the described procedure, carborane **1h** (87 mg, 0.2 mmol, 1 equiv.) was reacted with *N*-xanthylamide **2a** (4 equiv., 0.8 mmol, 346 mg) to afford **3h** (21 mg, 20%) as a yellow solid after silica gel chromatography (PE/Et_2O 99/1).
^{1}H-NMR (600 MHz, $CDCl_3$) δ: 1.43 (t, *J* = 7.14 Hz, 3H, CH_3-C*H*$_2$), 2.18-3.30 (m, 9H, carborane B-*H*), 4.61 (q, *J* = 7.14 Hz, 2H, O-C*H*$_2$-CH_3), 7.34-7.38 (m, 2H, Ar-*H*), 7.54-7.57 (m, 4H, Ar-*H*), 7.61-7.65 (m, 2H, Ar-*H*).
^{13}C{^{1}H}-NMR (150 MHz, $CDCl_3$) δ: 13.5 (CH_3), 70.4 (CH_2), 80.3 (C_q), 82.2 (C_q), 123.3 (q, *J* = 182.2 Hz, C_q), 127.2 (q, *J* = 2.7 Hz, CH), 127.4 (q, *J* = 2.0 Hz, CH), 127.8 (m, CH), 129.7 (CH), 129.8 (CH), 130.6 (q, *J* = 33.4 Hz, C_q), 131.6 (q, *J* = 34.2 Hz, C_q), 134.0 (CH), 134.1 (CH), 211.7 (C_q)
^{11}B{^{1}H}-NMR (192 MHz, $CDCl_3$) δ: -10.5, -9.43, -2.39, -1.05.
^{19}F{^{1}H}-NMR (564 MHz, $CDCl_3$) δ: -63.1
HRMS (ESI) m/z: $[M + H]^+$ Calcd for $C_{19}H_{23}B_{10}F_6OS_2$ 555.2025. Found: 555.2019
IR (thin film, cm-1) v: 2923, 2852, 2600, 2359, 1438, 1329, 1171, 1109, 1039, 694.
m.p.: 109.6 – 110.6 °C.
R_f: 0.10 (PE/Et_2O 99/1).

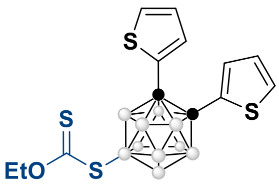

3i
B(9):B(8) = 2:1

Following the described procedure, carborane **1i** (62 mg, 0.2 mmol, 1 equiv.) was reacted with *N*-xanthylamide **2a** (4 equiv., 0.8 mmol, 346 mg) to afford **3i** as a mixture of regioisomers (25 mg, 40%, B(9):B(8) = 2:1) as a waxy yellow solid after silica gel chromatography (PE/Et_2O 99/1).
^{1}H-NMR (400 MHz, $CDCl_3$) δ: 1.42 (t, *J* = 7.12 Hz, 3H, CH_3-C*H*$_2$, major isomer), 1.50 (t, *J* = 7.12 Hz, 3H, CH_3-C*H*$_2$, minor isomer), 1.92-3.55 (m, 14H, carborane B-*H*, major isomer + minor isomer), 4.60 (q, *J* = 7.12 Hz, 2H, O-C*H*$_2$-CH_3, major isomer),

4.68 (q, J = 7.12 Hz, 2H, O-CH_2-CH_3, minor isomer), 6.81-6.85 (m, 3H, Ar-*H,* major isomer + minor isomer), 7.16-7.18 (m, 2H, Ar-*H*, major isomer + minor isomer), 7.19-7.23 (m, 5H, Ar-*H*, major isomer + minor isomer).

^{13}C{^{1}H}-NMR (100 MHz, $CDCl_3$) δ: 13.5 (CH_3, major isomer), 13.8 (CH_3, minor isomer), 70.4 (CH_2, major isomer), 70.6 (CH_2, minor isomer), 78.0 (C_q), 79.9 (C_q), 80.3 (C_q), 127.4 (CH), 127.4 (CH), 130.1 (CH), 130.1 (CH), 133.0 (CH), 133.1 (CH), 133.2 (CH), 133.7 (C_q), 134.1 (C_q), 212.2 (C_q).

^{11}B{^{1}H}-NMR (128 MHz, $CDCl_3$) δ: -10.2, -9.1, -3.5, -2.2, -4.0.

HRMS (ESI) m/z: $[M + H]^+$ Calcd for $C_{25}H_{29}B_{10}OS_4$ 583.2032. Found: 583.2024.

IR (thin film, cm-1) ν: 3067, 2924, 2599, 2159, 1438, 1372, 1186, 1061, 820, 694.

R_f: 0.10 (PE/Et_2O 99/1).

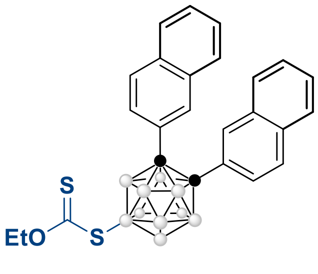

3j

Following the described procedure, carborane **1j** (28 mg, 0.1 mmol, 1 equiv.) was reacted with *N*-xanthylamide **2a** (173 mg, 0.40 mmol, 4 equiv.) to afford **1j** (14 mg, 27%) as a yellow solid after silica gel chromatography (PE/Et_2O 90/10 to 80/20).

^{1}H-NMR (400 MHz, $CDCl_3$) δ: 1.37 (t, J = 7.1 Hz, 3H, CH_3-CH_2), 2.46-3.88 (m, 9H, carborane B-*H*), 4.33 (q, J = 7.1 Hz, 2H, O-CH_2-CH_3), 7.41-7.44 (m, 5H, Ar-*H*), 7.56 (s, 4H, Ar-*H*), 7.63-7.65 (m, 2H, Ar-*H*), 7.70-7.72 (m, 2H, Ar-*H*), 8.03 (s, 1H, Ar-*H*).

^{13}C{^{1}H}-NMR (100 MHz, $CDCl_3$) δ: 14.5 (CH_3), 63.5 (CH_2), 85.4 (C_q), 127.0 (CH), 127.1 (CH), 127.5 (CH), 127.6 (C_q), 128.0 (CH), 128.4 (CH), 128.8 (CH), 131.7 (CH), 132.4 (C_q), 133.7 (C_q).

^{11}B{^{1}H}-NMR (128 MHz, $CDCl_3$) δ: -11.1, -.9.7, -3.1, -1.7.

HRMS (ESI) m/z: $[M + H]^+$ Calcd for $C_{25}H_{29}B_{10}OS_2$ 519.2512. Found: 519.2501.

IR (thin film, cm-1) ν: 3057, 2923, 2594, 1125, 1072, 1017, 724.

R_f: 0.30 (PE/Et_2O 90/10).

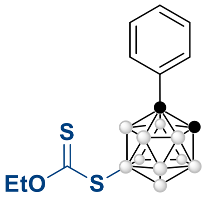

3k
B(9):B(8) = 3:1

Following the described procedure, carborane **1k** (44 mg, 0.2 mmol, 1 equiv) was reacted with *N*-xanthylamide **2a** (4 equiv., 0.8 mmol, 346 mg) to afford 3k as a mixture of regioisomers (26 mg, 39%, B(9):B(8)= 3:1) as a yellow solid after silica gel chromatography (PE/Et_2O 99/1).

^{1}H-NMR (400 MHz, $CDCl_3$) δ: 1.44 (t, J = 7.12 Hz, 3H, CH_3-CH_2, major isomer), 1.47-1.51 (m, 3H, CH_3-CH_2, minor isomer), 1.92-3.51 (m, 12H, carborane B-*H*, major isomer + minor isomer), 3.98 (bs, 1H, carborane-C*H,* minor isomer), 3.99 (bs, 1H, carborane-C*H,* major isomer), 4.61 (q, J = 7.24 Hz, 2H, O-CH_2-CH_3, major isomer), 4.68-4.73 (m, 2H, O-CH_2-CH_3,minor isomer), 7.33-7.38 (m, 3H, Ar-*H*, major isomer + minor isomer), 7.41-7.43 (m, 1H, Ar-*H*, major isomer + major isomer), 7.46-7.50 (m, 3H, Ar-*H*, major isomer + major isomer).

^{13}C{^{1}H}-NMR (100 MHz, $CDCl_3$) δ: 13.6 (CH_3, major isomer), 13.8 (CH_3, mionr isomer), 54.7 (carborane-CH, major isomer), 56.3 (carborane-CH, minor isomer), 70.4 (CH_2, major isomer), 71.1 (CH_2, minor isomer), 12.6 (CH), 127.7 (CH), 129.9 (CH), 130.4 (CH), 132.7 (C_q), 212.2 (C_q).

^{11}B{^{1}H}-NMR (128 MHz, $CDCl_3$) δ: -13.6, -12.0, -11.0, -8.7, -3.8, -1.3, 1.7, 4.0, 6.3, 7.2.

HRMS (ESI) m/z: $[M + H]^+$ Calcd for $C_{11}H_{21}B_{10}OS_2$ 342.1886. Found: 342.1881.

IR (thin film, cm-1) ν: 2922, 2600, 1230, 1111, 1040, 689.

m.p.: 178.2 – 181.1 °C.

R_f: 0.10 (PE/Et_2O 99/1).

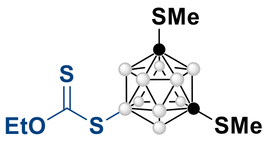

3l

Following the described procedure, carborane **1l** (35.4 mg, 0.15 mmol, 1 equiv.) was reacted with *N*-xanthylamide **2a** (260 mg, 0.60 mmol, 4 equiv.) to afford **3l** (2.5 mg, 4%) as a waxy yellow solid after silica gel chromatography (PE/Et_2O 99/1).

^{1}H-NMR (400 MHz, $CDCl_3$) δ: 1.23 (t, *J* = 7.12 Hz, 3H, CH_3-CH_2 + residual grease), 1.71-2.73 (m, 9H, carborane B-*H*), 2.23 (6s, 6H, S-CH_3), 4.17 (q, *J* = 7.12 Hz, 2H, O-CH_2-CH_3).

^{13}C{^{1}H}-NMR (100 MHz, $CDCl_3$) δ: 19.6 (CH_3), 29.9 (CH_3), 63.7 (CH_2). *Quaternary carbons were not detected.*

^{11}B{^{1}H}-NMR (128 MHz, $CDCl_3$) δ: -14.1, 12.8, -10.9, -10.3, -6.0, -3.6.

HRMS (ESI) m/z: $[M + H]^+$ Calcd for $C_7H_{21}B_{10}OS_4$ 359.1406. Found: 359.1392.

R_f: 0.12 (PE/Et_2O 99/1).

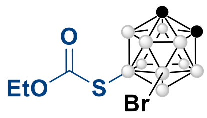

3n
B(10):B(8) = 1:1

Following the described procedure, carborane **1n (**44.4 mg, 0.2 mmol, 1 equiv.) was reacted with *N*-xanthylamide **2a** (346 mg, 0.80 mmol, 4 equiv.) to afford **3n** as a mixture of regioisomers (20 mg, 30%, B(10):B(8)= 1:1) as a waxy yellow solid after silica gel chromatography (PE/DCM 90/10).

^{1}H-NMR (400 MHz, $CDCl_3$) δ: 1.43-1.4 (m, 6H CH_3-CH_2), 2.01-3.15 (m, 16H, carborane B-*H*), 3.70 (bs, 2H, carborane-C*H*), 3.74 (bs, carborane-C*H*), 4.59-4.69 (m, 4H, O-CH_2-CH_3).

^{13}C{^{1}H}-NMR (100 MHz, $CDCl_3$) δ: 13.6 (CH_3), 13.6 (CH_3), 29.8 (CH), 30.4 (CH), 70.6 (CH_2), 70.7 (CH_2). *Quaternary carbons not detected.*

^{11}B{^{1}H}-NMR (128 MHz, $CDCl_3$) δ: -17.6, -16.3, -15.1, -14.3, -9.9, -9.1, -3.3, -2.1, -0.5, 2.4, 5.7.

HRMS (ESI) m/z: $[M + H]^+$ Calcd for $C_5H_{16}B_{10}BrOS_2$ 345,0767. Found: 345.0695.

IR (thin film, cm-1) ν: 3055, 2619, 1231, 1132, 1020.

R_f: 0.10 (PE/Et_2O 99/1).

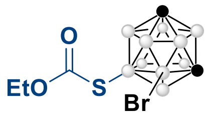

3o
B(9):B(5):B(4) = 6:3:1

Following the described procedure, carborane **1o (**44.4 mg, 0.2 mmol, 1 equiv.) was reacted with *N*-xanthylamide **2a** (346 mg, 0.80 mmol, 4 equiv.) to afford **3o** as a mixture of regioisomers (30 mg, 44%, B(9):B(5):B(4)= 6:3:1) as a waxy yellow solid after silica gel chromatography (PE/DCM 90/10).

^{1}H-NMR (400 MHz, $CDCl_3$) δ: 1.42-1.49 (m, CH_3-CH_2, major isomer + minor isomers), 1.77-3.41 (m, 11H, carborane B-*H*, major isomer + minor isomer), 3.05 (bs, 2H, carborane-C*H*, major isomer), 3.50 (bs, carborane-C*H*, minor isomer), 3.64 (bs, carborane-C*H*, minor isomer), 3.71 (bs, carborane-C*H*, minor isomer), 4.57-4.68 (m, O-CH_2-CH_3, major isomer + minor isomer).

^{13}C{^{1}H}-NMR (100 MHz, $CDCl_3$) δ: 13.5 (CH_3), 13.6 (CH_3), 13.7 (CH_3), 29.3 (CH), 29.4 (CH), 70.8 (CH_2), 71.0 (CH_2), 71.1 (CH_2), 208.9 (C_q), 209.3 (C_q), 215.7 (C_q)

^{11}B{^{1}H}-NMR (128 MHz, $CDCl_3$) δ: -20.8, -18.4, -17.8, -17.3, -14.7, -13.2, -9.5, -8.5, -6.5, -6.0, -0.43.

HRMS (ESI) m/z: $[M + H]^+$ Calcd for $C_5H_{16}B_{10}BrOS_2$ 345,0767. Found: 345.0691.

IR (thin film, cm-1) ν: 3049, 2608, 1230, 1143, 1039.

R_f: 0.10 (PE/Et_2O 99/1).

S4.10. Synthetic applications

S4.10.1. Synthesis of 4a

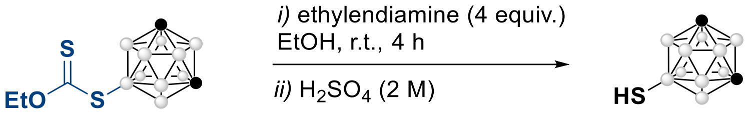

4a

9-mercapto-*m*-carborane (4a)

In a 25 ml round bottom flask, xanthyl carborane **3a** (0.13 mmol, 34 mg, 1 equiv.) is dissolved in absolute ethanol (5 ml). Previously distilled ethylendiamine (0.52 mmol, 34 ul, 4 equiv.) is added dropwise at room temperature. The reaction mixture is stirred at room temperature until complete conversion of the starting material. After 4 h, the volatiles are removed *in vacuo* and the crude is redissolved in DCM (10 ml), and acidified with 2 M H_2SO_4 (10 ml). The organic layer is washed with brine, dried over Na_2SO_4 and the solvent is removed *in vacuo*. The crude product is then purified by silica gel chromatography (EP/DCM 90/10) to afford **4a** as a white solid (13 mg, 57%). Spectral data are coherent with those reported in the literature.[15]

^{1}H-NMR (400 MHz, $CDCl_3$) δ: 0.47 (bs, 1H, carborane-S*H*), 1.72-2.28 (m, 9H, carborane B-*H*), 2.98 (bs, 2H, carborane-C*H*).

S4.10.2. Synthesis of 4b

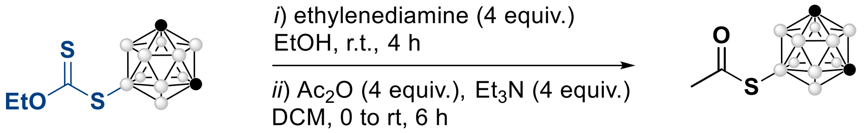

4b

***m*-Carborane-9-thiol acetate (4b)**

In a 10 ml round bottom flask, xanthyl carborane **3a** (0.2 mmol, 53 mg, 1 equiv.) is dissolved in absolute ethanol (2 ml). Previously distilled ethylendiamine (0.8 mmol, 53 ul, 4 equiv.) is added dropwise at room temperature. After 4 h, the volatiles are removed *in vacuo*. The crude material is dissolved in dry DCM (3 ml) and the mixture is cooled to 0 °C in an ice bath. Acetic anhydride (0.8 mmol, 76 ul, 4 equiv.) and triethylamine (0.8 mmol, 35 ul, 4 equiv.) are added dropwise. The reaction mixture is allowed to warm to room temperature for 6 h. After completion of the starting material, the reaction mixture is quenched with distilled water and extracted with DCM. The organic layer is dried over Na_2SO_4 and the volatiles are removed *in vacuo*. The crude product is then purified by silica gel chromatography (EP/Et_2O 98/2) to afford **4b** as a white solid (27 mg, 63%).

^{1}H-NMR (400 MHz, $CDCl_3$) δ: 1.68-3.43 (m, 9H, carborane-B*H*), 2.37 (s, 3H, CH_3), 2.98 (bs, 2H, carborane-C*H*).

^{13}C{^{1}H}-NMR (100 MHz, $CDCl_3$) δ: 33.2 (CH_3), 54.5 (CH), 194.3 (C_q).

^{11}B{^{1}H}-NMR (128 MHz, $CDCl_3$) δ: -18.5, -17.3, -13.9, -13.0, -10.1, -6.0, -4.1.

HRMS (ESI) m/z: [M + H]+ Calcd for $C_4H_{15}B_{10}OS$ 221.1774. Found 221.1769.

IR (thin film, cm-1) ν: 2930, 2902, 2598, 1652, 928, 711.

m.p.: 78.1 – 79.6 °C

R$_f$: 0.15 (PE/Et_2O 98/2).

S4.10.3. Synthesis of 4c

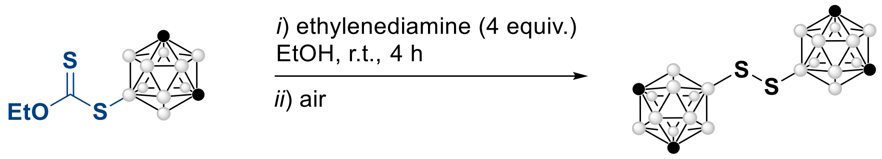

4c

1,2-di(1,7-dicarba-closo-dodecaboran-9-yl)disulfane (4c)

In a 8 ml scintillation vial, xanthyl carborane **3a** (0.026 mmol, 7 mg, 1 equiv.) is dissolved in absolute ethanol (1 ml). Previously distilled ethylendiamine (0.1 mmol, 6 ul, 4 equiv.) is added dropwise at room temperature. After 4 h, the reaction mixture is quenched with NH_4Cl sat. and extracted with diethyl ether. The organic layer is dried over Na_2SO_4 and the volatiles are removed *in vacuo*. The resulting white solid is left under air for 2 days. The crude product is then purified by silica gel chromatography (EP/$CHCl_3$ 80/20) to afford **4c** as a white solid (5 mg, 55%). Spectral data are coherent with those reported in the literature.[16]

^{1}H-NMR (400 MHz, $CDCl_3$) δ: 1.74-3.33 (m, 18H, carborane-B*H*), 3.04 (bs, 4H, carborane-C*H*).

S4.10.4. Synthesis of 4d

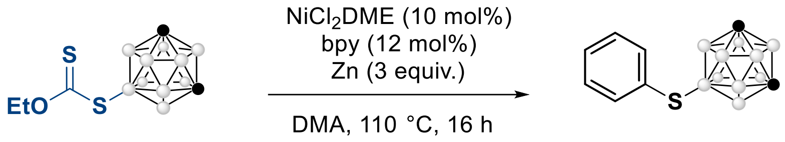

4d

9-phenylthio-*m*-carborane (4d)

Following a modified version of a procedure reported in the literature:[17] In an oven-dried Schlenk tube, xantyl carborane **3a** (0.2 mmol, 53 mg, 1 equiv.), $NiCl_2DME$ (0.02 mol, 4 mg, 10 mol%), bpy (0.024 mmol, 4 mg, 12 mol%) and zinc turnings (0.6 mmol, 40 mg, 3 equiv., previously activated with an acid wash) are added. The Schlenk tube is sealed with a rubber septum, evacuated and backfilled with N_2 three times DMA (3 ml) is then added and the reaction mixture is stirred at 110 °C for 16 h. After consumption of the starting material, the reaction mixture is cooled to room temperature, quenched with H_2O and extracted with ethyl acetate. The organic layer is dried over Na_2SO_4 and the volatiles are removed *in vacuo*. The crude product is then purified by silica gel chromatography (EP/Et_2O 98/2) to afford **4d** as a yellow solid (27 mg, 53%). Spectral data are coherent with those reported in the literature.[18]

^{1}H-NMR (400 MHz, $CDCl_3$) δ: 2.90 (bs, 1H, carborane-C*H*), 2.95 (bs, 1H, carborane-C*H*), 1.62-2.78 (m, 9H, carborane-B*H*), 7.26 (m, 4H, Ar-*H*), 7.51-7.52 (m, 1H, Ar-*H*).

S4.10.5. Synthesis of 4e

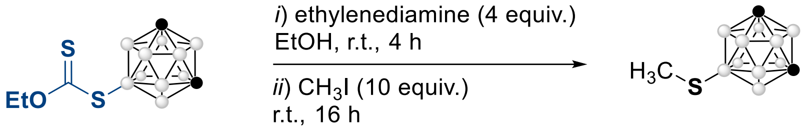

4e

9-Methylthio-*m*-carborane (4e)

In a 8 ml scintillation vial, xanthyl carborane **4a** (0.08 mmol, 21 mg, 1 equiv.) is dissolved in absolute ethanol (1.5 ml). Previously distilled ethylendiamine (0.32 mmol, 21 ul, 4 equiv.) is added dropwise at room temperature. After 4 h, iodomethane (0.8 mmol, 49 ul, 10 equiv.) is added. The reaction mixture is stirred at room temperature overnight. After consumption of the starting material, the reaction mixture is quenched with NH_4Cl sat. and extracted with diethyl ether. The organic layer is dried over Na_2SO_4 and the volatiles are removed *in vacuo*. The crude product is then purified by silica gel chromatography (EP/Et_2O 98/2 to 96/4) to afford **4e** as a white solid (9 mg, 59%). Spectral data are coherent with those reported in the literature.[13]

^{1}H-NMR (400 MHz, $CDCl_3$) δ: 1.82-3.35 (m, 9H, carborane B-*H*), 2.09-2.12 (m, 3H, S-CH_3), 2.95 (bs, 2H, carborane-C*H*).

S4.10.6. Synthesis of 4f

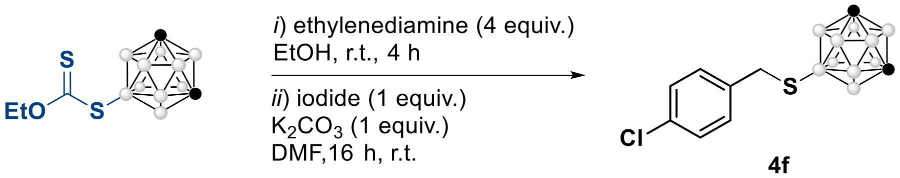

4f

9-(4-Chlorobenzyl)-*m*-carborane (4f)

1-Chloro-4-(iodomethyl)benzene was prepared following a reported procedure in the literature.[19]

In a round bottom flask, xanthyl carborane **4a** (0.3 mmol, 79 mg, 1 equiv.) is dissolved in absolute ethanol (3 ml). Previously distilled ethylendiamine (1.2 mmol, 80 ul, 4 equiv.) is added dropwise at room temperature. After 4 h, the volatiles are removed *in vacuo*. The resulting crude is redissolved in dry DMF (3 ml). K2CO3 (0.3 mmol, 41 mg, 1 equiv.) and 1-chloro-4-(iodomethyl)benzene (0.3 mmol, 58 mg, 1 equiv.) are added in one portion. The reaction mixture is stirred at room temperature overnight. After reaction completion, the volatiles are removed *in vacuo*. The crude product is then purified by silica gel chromatography (EP/Et_2O 98/2) to afford **4f** as a white solid (68 mg, 76%).

^{1}H-NMR (400 MHz, $CDCl_3$) δ: 1.79 -3.33 (m, 9H, carborane B-*H*), 2.95 (bs, 2H, carborane C-*H*), 3.76 (m, 2H, CH_2), 7.23-7.26 (m, 2H, Ar-*H*), 7.30-7.32 (m, 2H, Ar-*H*).

^{13}C{^{1}H}-NMR (100 MHz, $CDCl_3$) δ: 36.4 (CH_2), 54.2 (CH), 128.5 (CH), 130.2 (CH), 132.5 (C_q), 138.6 (C_q).

^{11}B{^{1}H}-NMR (128 MHz, $CDCl_3$) δ: -20.3, -17.5, -13.9, -13.1, -9.9, -6.5, -0.1.

HRMS (ESI) m/z: $[M + H]^+$ Calcd for $C_9H_{18}B_{10}ClS$ 303.1748. Found: 303.1743

IR (thin film, cm-1) ν: 2931, 2600, 1488, 1220, 1036, 799, 501.

m.p.: 106.0 – 107.7 °C

R$_f$: 0.4 (PE/Et_2O 90/10).

S4.10.7. Synthesis of 4g

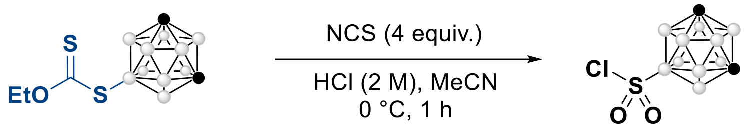

4g

9-chlorosulfonyl-*m*-carborane (4g)

In a 10 ml round bottom flask, NCS (0.84 mmol, 112.2 mg, 4 equiv.) is dissolved in a solution of HCl (2 M, 15 ul) in MeCN (1 ml) at 10 °C. The solution is cooled to 0 °C and xanthyl carborane **3a** (0.21 mmol, 57 mg, 1 equiv.) is added as a solution in MeCN (1.5 ml). The reaction mixture is stirred at 0 °C until conversion of the starting material. After 1 h, the reaction is quenched with H_2O and extracted with diethyl ether. The organic layer is dried over Na_2SO_4 and the volatiles are removed *in vacuo.* The crude product is then purified by silica gel chromatography (EP/EtOAc 85/15 to 70/30) to afford **4g** as a white solid (15 mg, 31%).

^{1}H-NMR (400 MHz, $CDCl_3$) δ: 1.72-3.67 (m, 9H, carborane B-*H*), 3.20 (bs, 2H, carborane-C*H*).

^{13}C{^{1}H}-NMR (100 MHz, $CDCl_3$) δ: 55.05 (CH).

^{11}B{^{1}H}-NMR (128 MHz, $CDCl_3$) δ: -16.6, -13.0, -9.9, -6.1, 0.1.

HRMS (ESI) m/z: $[M + H]^+$ Calcd for $C_2H_{12}B_{10}ClO_2S$ 245.1177. Found: 245.1168.

IR (thin film, cm-1) v: 2600, 1439, 1211, 1201, 1185, 589, 405.

m.p.: 68.1 – 68.8 °C (decomposition)

R_f: 0.20 (PE/EtOAc 80/20).

S4.10.8. Synthesis of 4h

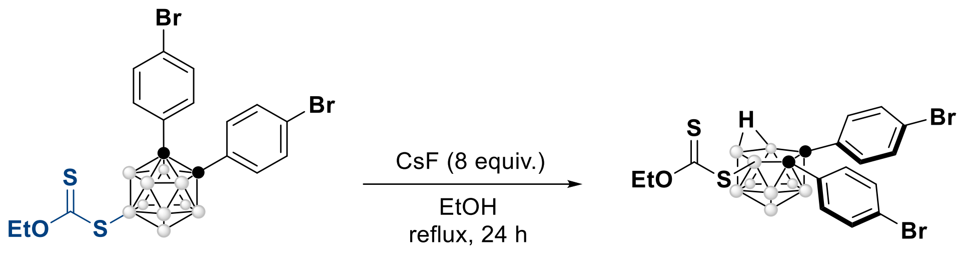

4h

1,2-bis(4-bromophenyl)-9-ethoxy(thiocarbonyl)thio-*nido*-carborane (4h)

In a 8 ml scintillation vial, xanthyl carborane **3f** (0.07 mmol, 18.5 mg, 1 equiv.) and CsF (0.56 mmol, 85 mg, 8 equiv.) are dissolved in absolute ethanol (2 ml). The solution is heated to reflux for 24 h. After completion, the reaction mixture is allowed to cool to room temperature and the volatiles are removed *in vacuo*. The crude product is then purified by silica gel chromatography (DCM/MeOH 100/0 to 90/10) to afford **4h** as a white solid (17 mg, 47%).

^{1}H-NMR (400 MHz, $CDCl_3$) δ: -1.33(sb, 1H, B-*H*-B), 1.47 (t, *J* = 7.2 Hz, 3H, CH_2-CH_3), 1.25-3.51 (m, 9H, carborane-B*H*), 4.60 (q, *J* = 7.2 Hz, 2H, O-CH_2-CH_3), 6.96 (dd, *J* = 15.5, 8.5 Hz, 4H, Ar-*H*), 7.07-7.11 (m, 4H, Ar-*H*).

^{13}C{^{1}H}-NMR (100 MHz, $CDCl_3$) δ: 13.7 (CH_3), 72.3 (CH_2), 120.6 (C_q), 120.7 (C_q), 130.5 (CH), 133.1 (CH), 33.2 (CH), 138.7 (C_q), 138.9 (C_q).

^{11}B{^{1}H}-NMR (128 MHz, $CDCl_3$) δ: -35.3, -32.0, -14.1, -11.8, -8.9.

HRMS (m/z) ESI: $[M - H]^-$ Calcd $C_{17}H_{22}OB_9Br_2S_2$ 567.0275. Found: 564.0345

IR (thin film, cm-1) v: 2921, 2526, 1612, 1486, 1113, 1035, 813.

$\mathbf{R_f}$: 0.50 (DCM/MeOH 90/10)

S5. Crystal data

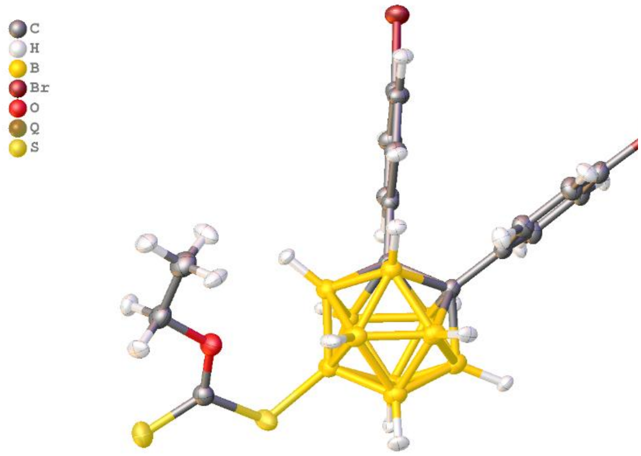

Experimental. Single clear colourless prism-shaped crystals of **3f** were used as supplied. A suitable crystal with dimensions 0.13 × 0.08 × 0.04 mm was selected and mounted on a MITIGEN holder with inert oil on a XtaLAB Synergy R, DW system, HyPix-Arc 150 diffractometer. The crystal was kept at a steady T = 123.00(10) K during data collection. The structure was solved with the ShelXT 2018/2 (Sheldrick, 2018) solution program using dual methods and by using Olex2 1.5-alpha (Dolomanov et al., 2009) as the graphical interface. The model was refined with olex2.refine 1.5-alpha (Bourhis et al., 2015) using full matrix least squares minimisation on $|F|^2$.

Crystal Data. $C_{17}H_{22}B_{10}Br_2OS_2$, M_r = 574.545, monoclinic, $P2_1/n$ (No. 14), a = 11.5368(1) Å, b = 14.1909(2) Å, c = 14.8720(1) Å, β = 96.797(1)°, $\alpha = \gamma$ = 90°, V = 2417.70(4) Å^3, T = 123.00(10) K, Z = 4, Z' = 1, μ(Cu K_α) = 5.926, 52434 reflections measured, 4981 unique (R_{int} = 0.0239) which were used in all calculations. The final wR_2 was 0.0325 (all data) and R_1 was 0.0158 (I≥2σ(I)).

**Compound**	**3f**
Formula	$C_{17}H_{22}B_{10}Br_2OS_2$
$D_{calc.}$/ g cm^{-3}	1.578
μ/mm^{-1}	5.926
Formula Weight	574.545
Colour	clear colourless
Shape	prism-shaped
Size/mm	0.13×0.08×0.04
T/K	123.00(10)
Crystal System	monoclinic
Space Group	$P2_1/n$
a/Å	11.5368(1)
b/Å	14.1909(2)
c/Å	14.8720(1)
α/°	90
β/°	96.797(1)
γ/°	90
V/Å^3	2417.70(4)
Z	4
Z'	1
Wavelength/Å	1.54184
Radiation type	Cu K_α
Θ_{min}/°	4.32
Θ_{max}/°	76.05
Index range h	-14 ≥ h ≥ 14
Index range k	-17 ≥ k ≥ 17
Index range l	-16 ≥ l ≥ 18
Measured Refl's.	52434
Indep't Refl's	4981
Refl's I≥2σ(I)	4696
R_{int}	0.0239
Parameters	487
Restraints	0
Largest Peak/eÅ^3	0.5938
Deepest Hole/eÅ^3	-0.4212
GooF	1.0695
R_1 (I≥2σ(I) / all)	0.0158 / 0.0176
wR_2 (I≥2σ(I) / all)	0.0318 / 0.0325

Structure Quality Indicators

**Reflections:**	d min (CuKα) 2Θ=152.1° 0.79	I/σ(I) 95.7	Rint m=10.86 2.39%	Full 135.4° 99% to 152.1° 100
**Refinement:**	Shift 0.000	Max Peak 0.6	Min Peak -0.4	GooF 1.069

A clear colourless prism-shaped crystal with dimensions 0.13 × 0.08 × 0.04 mm was mounted on a MITIGEN holder with inert oil. Data were collected using a XtaLAB Synergy R, DW system, HyPix-Arc 150 diffractometer equipped with an Oxford Cryosystems Cryostream 700 low-temperature device operating at T = 123.00(10) K.

Data were measured using ω scans with Cu K_α radiation. The diffraction pattern was indexed and the total number of runs and images was based on the strategy calculation from the program CrysAlis^{Pro} system (CCD 44.122a 64-bit (release 14-09-2025)). The maximum resolution achieved was Θ = 76.05° (0.79 Å).

The unit cell was refined using CrysAlis^{Pro} on 15818 reflections, 30% of the observed reflections.

Data reduction, scaling and absorption corrections were performed using CrysAlis^{Pro}. The final completeness is 100.00 % out to 76.05° in Θ. A gaussian absorption correction was performed using CrysAlis^{Pro} 1.171.44.129a (Rigaku Oxford Diffraction, 2025) Numerical absorption correction based on gaussian integration over a multifaceted crystal model Empirical absorption correction using spherical harmonics, implemented in SCALE3 ABSPACK scaling algorithm. The absorption coefficient μ of this material is 5.926 mm^{-1} at this wavelength (λ = 1.54184Å) and the minimum and maximum transmissions are 0.700 and 1.000.

The structure was solved in the space group $P2_1/n$ (# 14) by ShelXT 2018/2 (Sheldrick, 2018) using dual methods. It was refined by full matrix least squares minimisation on $|\boldsymbol{F}|^2$ using version of olex2.refine 1.5-alpha (Bourhis et al., 2015). All non-hydrogen atoms were refined anisotropically.

Hydrogen atom positions were calculated geometrically and refined using the riding model.

_olex2_refine_details: Refinement using NoSpherA2, an implementation ofNOn-SPHERical Atom-form-factors in Olex2.Please cite:F. Kleemiss et al. Chem. Sci. DOI 10.1039/D0SC05526C - 2021NoSpherA2 implementation of HAR makes use oftailor-made aspherical atomic form factors calculatedon-the-fly from a Hirshfeld-partitioned electron density (ED) - not fromspherical-atom form factors.The ED is calculated from a gaussian basis set single determinant SCFwavefunction - either Hartree-Fock or DFT using selected funtionals- for a fragment of the crystal.This fragment can be embedded in an electrostatic crystal field by employing cluster chargesor modelled using implicit solvation models, depending on the software used.The following options were used: SOFTWARE: ORCA 5.0 PARTITIONING: NoSpherA2 INT ACCURACY: Normal METHOD: r2SCAN BASIS SET: def2-TZVP CHARGE: 0 MULTIPLICITY: 1 DATE: 2025-11-20_12-01-16

_exptl_absorpt_process_details: CrysAlis^{Pro} 1.171.44.129a (Rigaku Oxford Diffraction, 2025)Numerical absorption correction based on gaussian integration overa multifaceted crystal modelEmpirical absorption correction using spherical harmonics,implemented in SCALE3 ABSPACK scaling algorithm.

There is a single formula unit in the asymmetric unit, which is represented by the reported sum formula. In other words: Z is 4 and Z' is 1. The moiety formula is C17 H22 B10 Br2 O S2.

Data Plots: Diffraction Data

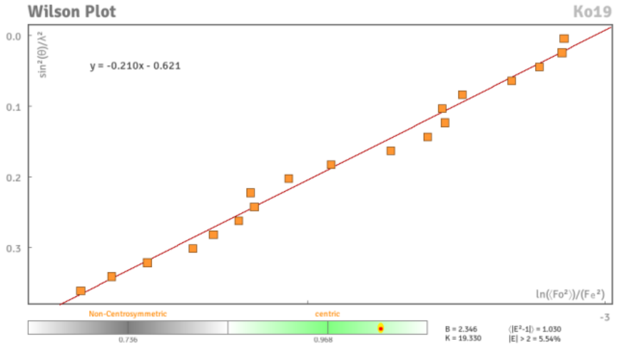

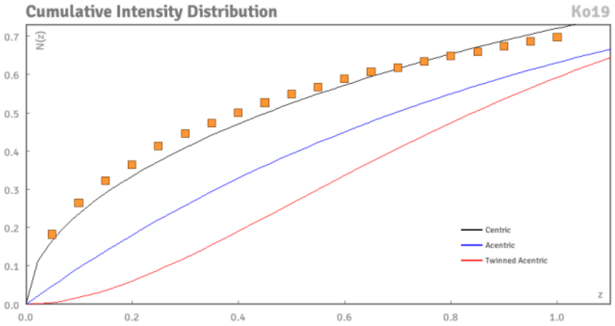

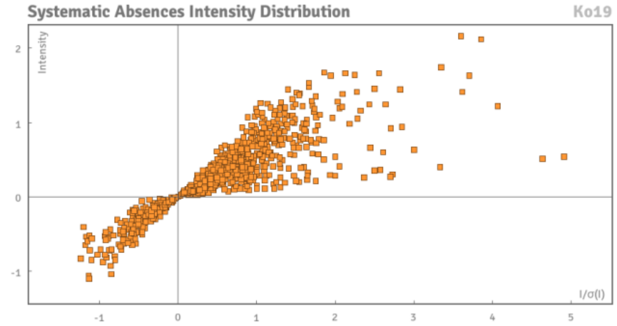

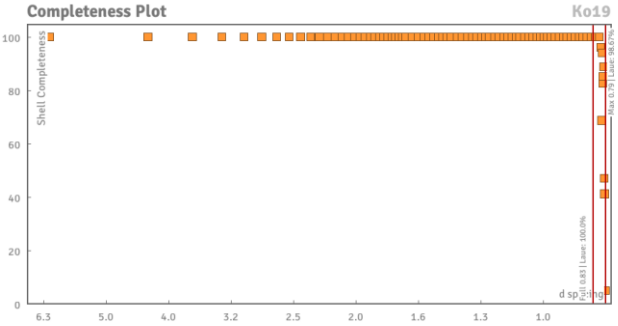

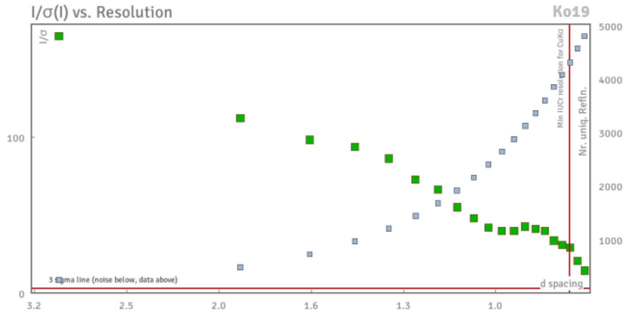

Data Plots: Refinement and Data

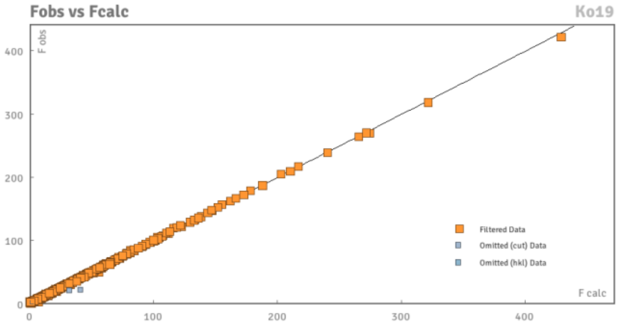

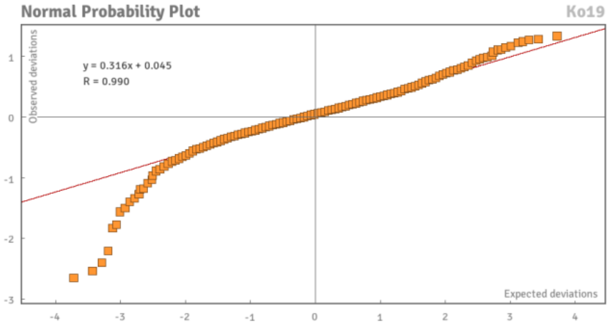

Reflection Statistics

Total reflections (after filtering)	54088	Unique reflections	4981
Completeness	0.987	Mean I/σ	59.21
hkl_{max} collected	(14, 17, 18)	hkl_{min} collected	(-14, -17, -16)
hkl_{max} used	(14, 17, 18)	hkl_{min} used	(-14, 0, 0)
Lim d_{max} collected	100.0	Lim d_{min} collected	0.77

d_{max} used	14.77	d_{min} used	0.79
Friedel pairs	5533	Friedel pairs merged	1
Inconsistent equivalents	10	R_{int}	0.0239
R_{sigma}	0.0104	Intensity transformed	0
Omitted reflections	0	Omitted by user (OMIT hkl)	2
Multiplicity	(3544, 4470, 2559, 1872, 535, 416, 300, 310, 192, 165, 133, 135, 101, 92, 77, 60, 50, 34, 38, 32, 22, 32, 22, 19, 11, 11)	Maximum multiplicity	51
Removed systematic absences	1656	Filtered off (Shel/OMIT)	0

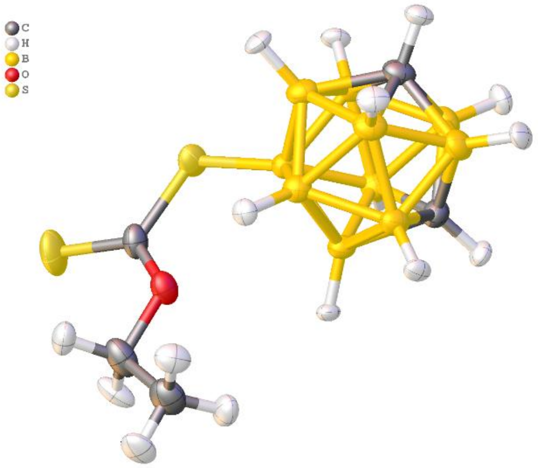

Experimental. Single clear colourless prism-shaped crystals of **3a** were used as supplied. A suitable crystal with dimensions 0.25 × 0.20 × 0.08 mm was selected and mounted on a MITIGEN holder with inert oil on a XtaLAB Synergy R, DW system, HyPix-Arc 150 diffractometer. The crystal was kept at a steady T = 123.00(10) K during data collection. The structure was solved with the ShelXT 2018/2 (Sheldrick, 2018) solution program using dual methods and by using Olex2 1.5-alpha (Dolomanov et al., 2009) as the graphical interface. The model was refined with olex2.refine 1.5-alpha (Bourhis et al., 2015) using full matrix least squares minimisation on $|\boldsymbol{F}|^2$.

Crystal Data. $C_5H_{16}B_{10}OS_2$, M_r = 264.557, monoclinic, $P2_1/n$ (No. 14), a = 6.58942(4) Å, b = 13.34256(10) Å, c = 16.09094(11) Å, β = 91.1508(6)°, $\alpha = \gamma$ = 90°, V = 1414.425(17) Å^3, T = 123.00(10) K, Z = 4, Z' = 1, μ(Cu K$_\alpha$) = 3.144, 30619 reflections measured, 2883 unique (R_{int} = 0.0200) which were used in all calculations. The final wR_2 was 0.0420 (all data) and R_1 was 0.0189 (I≥2σ(I)).

**Compound**	**3a**
Formula	$C_5H_{16}B_{10}OS_2$
$D_{calc.}$/ g cm^{-3}	1.242
μ/mm^{-1}	3.144
Formula Weight	264.557
Colour	clear colourless
Shape	prism-shaped
Size/mm	0.25×0.20×0.08
T/K	123.00(10)
Crystal System	monoclinic
Space Group	$P2_1/n$
a/Å	6.58942(4)
b/Å	13.34256(10)
c/Å	16.09094(11)
α/°	90
β/°	91.1508(6)
γ/°	90
V/Å^3	1414.425(17)
Z	4
Z'	1
Wavelength/Å	1.54184
Radiation type	Cu K$_\alpha$
Θ_{min}/°	4.30
Θ_{max}/°	75.55
Index range h	-5 ≥ h ≥ 8
Index range k	-16 ≥ k ≥ 16
Index range l	-19 ≥ l ≥ 20
Measured Refl's.	30619
Indep't Refl's	2883
Refl's I≥2σ(I)	2777
R_{int}	0.0200
Parameters	307
Restraints	0
Largest Peak/eÅ^3	0.4527
Deepest Hole/eÅ^3	-0.3499
GooF	1.0890
R_1 (I≥2σ(I) / all)	0.0189 / 0.0198
wR_2 (I≥2σ(I) / all)	0.0417 / 0.0420

Structure Quality Indicators

**Reflections:**	d min (CuKα) 2Θ=151.1° 0.80	I/σ(I) 112.0	Rint m=10.97 2.00%	Full 135.4° 98% to 151.1° 100
**Refinement:**	Shift -0.001	Max Peak 0.5	Min Peak -0.3	GooF 1.089

A clear colourless prism-shaped crystal with dimensions 0.25 × 0.20 × 0.08 mm was mounted on a MITIGEN holder with inert oil. Data were collected using a XtaLAB Synergy R, DW system, HyPix-Arc 150 diffractometer equipped with an Oxford Cryosystems Cryostream 700 low-temperature device operating at T = 123.00(10) K.

Data were measured using ω scans with Cu K_{α} radiation. The diffraction pattern was indexed and the total number of runs and images was based on the strategy calculation from the program CrysAlis^{Pro} system (CCD 44.122a 64-bit (release 14-09-2025)). The maximum resolution achieved was Θ = 75.55° (0.80 Å).

The unit cell was refined using CrysAlis^{Pro} on 15833 reflections, 52% of the observed reflections.

Data reduction, scaling and absorption corrections were performed using CrysAlis^{Pro}. The final completeness is 100.00 % out to 75.55° in Θ. A gaussian absorption correction was performed using CrysAlis^{Pro} 1.171.44.129a (Rigaku Oxford Diffraction, 2025) Numerical absorption correction based on gaussian integration over a multifaceted crystal model Empirical absorption correction using spherical harmonics, implemented in SCALE3 ABSPACK scaling algorithm. The absorption coefficient μ of this material is 3.144 mm^{-1} at this wavelength (λ = 1.54184Å) and the minimum and maximum transmissions are 0.448 and 1.000.

The structure was solved in the space group $P2_1/n$ (# 14) by ShelXT 2018/2 (Sheldrick, 2018) using dual methods. It was refined by full matrix least squares minimisation on $|\boldsymbol{F}|^2$ using version of olex2.refine 1.5-alpha (Bourhis et al., 2015). All non-hydrogen atoms were refined anisotropically.

Hydrogen atom positions were calculated geometrically and refined using the riding model.

_olex2_refine_details: Refinement using NoSpherA2, an implementation ofNOn-SPHERical Atom-form-factors in Olex2.Please cite:F. Kleemiss et al. Chem. Sci. DOI 10.1039/D0SC05526C - 2021NoSpherA2 implementation of HAR makes use oftailor-made aspherical atomic form factors calculatedon-the-fly from a Hirshfeld-partitioned electron density (ED) - not fromspherical-atom form factors.The ED is calculated from a gaussian basis set single determinant SCFwavefunction - either Hartree-Fock or DFT using selected funtionals- for a fragment of the crystal.This fragment can be embedded in an electrostatic crystal field by employing cluster chargesor modelled using implicit solvation models, depending on the software used.The following options were used: SOFTWARE: ORCA 5.0 PARTITIONING: NoSpherA2 INT ACCURACY: Normal METHOD: r2SCAN BASIS SET: def2-TZVP CHARGE: 0 MULTIPLICITY: 1 DATE: 2025-11-20_09-04-12

_exptl_absorpt_process_details: CrysAlis^{Pro} 1.171.44.129a (Rigaku Oxford Diffraction, 2025)Numerical absorption correction based on gaussian integration overa multifaceted crystal modelEmpirical absorption correction using spherical harmonics,implemented in SCALE3 ABSPACK scaling algorithm.

There is a single formula unit in the asymmetric unit, which is represented by the reported sum formula. In other words: Z is 4 and Z' is 1. The moiety formula is C5 H16 B10 O S2.

Data Plots: Diffraction Data

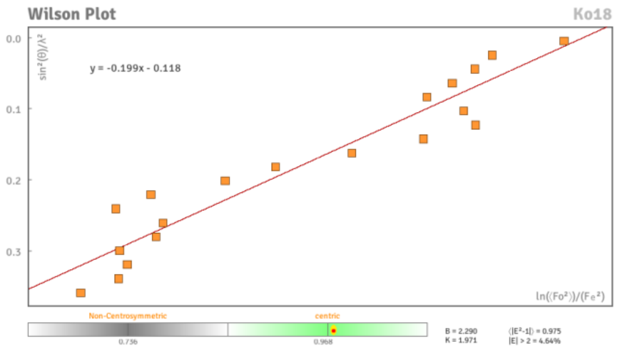

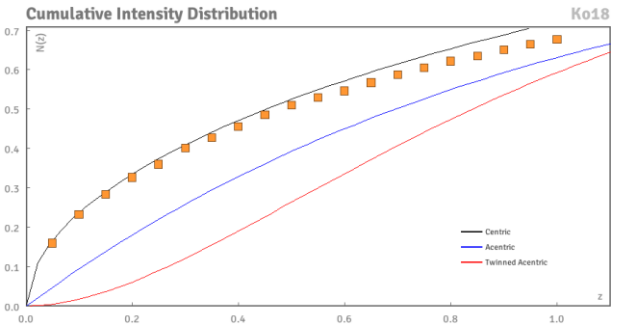

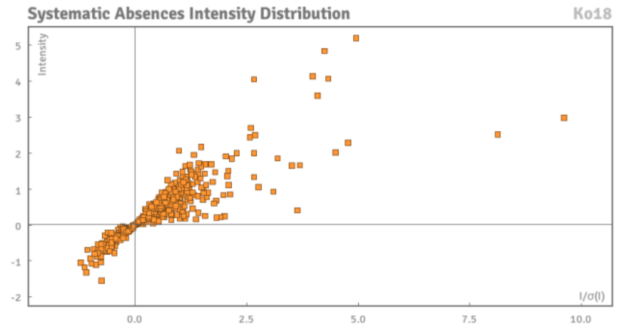

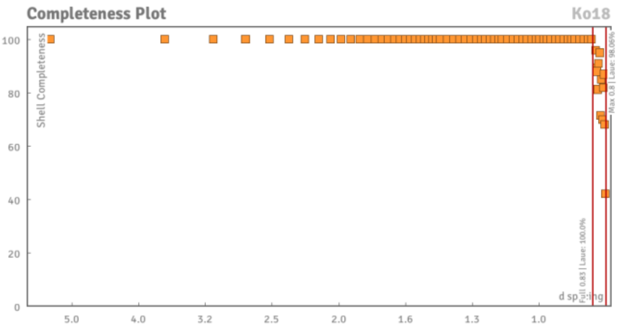

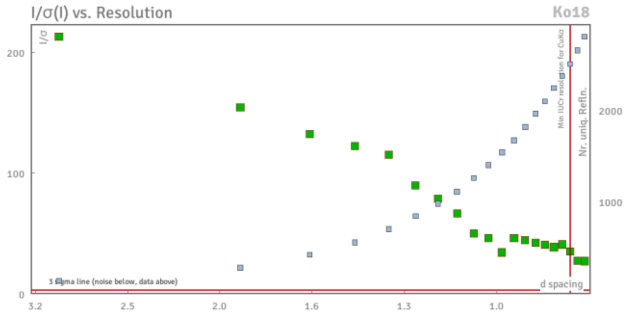

Data Plots: Refinement and Data

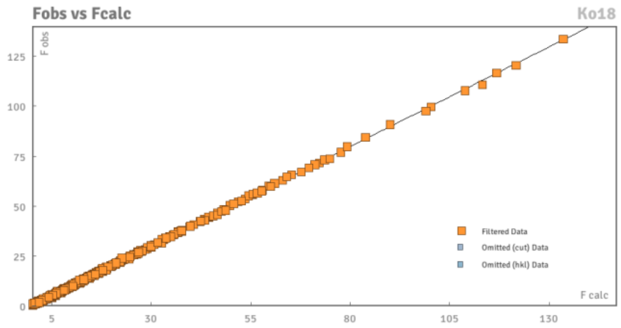

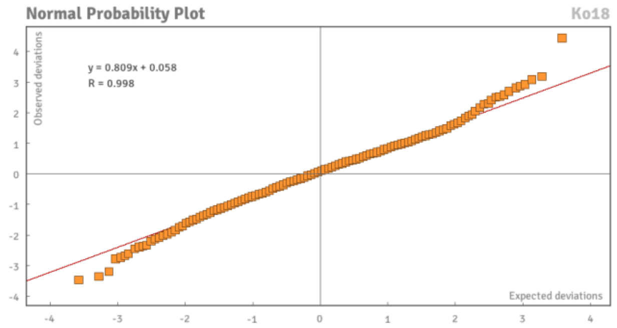

Reflection Statistics

Total reflections (after filtering)	31621	Unique reflections	2883
Completeness	0.981	Mean I/σ	72.8
hkl_{max} collected	(8, 16, 20)	hkl_{min} collected	(-5, -16, -19)
hkl_{max} used	(8, 16, 20)	hkl_{min} used	(-7, 0, 0)
Lim d_{max} collected	100.0	Lim d_{min} collected	0.77

d_{max} used	16.09	d_{min} used	0.8
Friedel pairs	3179	Friedel pairs merged	1
Inconsistent equivalents	9	R_{int}	0.02
R_{sigma}	0.0089	Intensity transformed	0
Omitted reflections	0	Omitted by user (OMIT hkl)	0
Multiplicity	(1666, 2198, 1939, 1366, 503, 241, 195, 134, 87, 75, 72, 57, 53, 40, 32, 25, 27, 26, 14, 18, 15, 15, 11, 10, 1, 1)	Maximum multiplicity	54
Removed systematic absences	1002	Filtered off (Shel/OMIT)	0

Citations

CrysAlis^{Pro} (Rigaku Oxford Diffraction), Rigaku Oxford Diffraction, Poland (?).

L.J. Bourhis and O.V. Dolomanov and R.J. Gildea and J.A.K. Howard and H. Puschmann, The Anatomy of a Comprehensive Constrained, Restrained, Refinement Program for the Modern Computing Environment - Olex2 Disected, *Acta Cryst. A*, (2015), **A71**, 59-71.

O.V. Dolomanov and L.J. Bourhis and R.J. Gildea and J.A.K. Howard and H. Puschmann, Olex2: A complete structure solution, refinement and analysis program, *J. Appl. Cryst.*, (2009), **42**, 339-341.

Sheldrick, G.M., ShelXT-Integrated space-group and crystal-structure determination, *Acta Cryst.*, (2015), **A71**, 3-8.

S6. References

[1] R. M. Dziedzic, J. L. Martin, J. C. Axtell, L. M. A. Saleh, T.-C. Ong, Y.-F. Yang, M. S. Messina, A. L. Rheingold, K. N. Houk, A. M. Spokoyny, *J. Am. Chem. Soc.* **2017**, *139*, 7729-7732.

[2] S. Stoll, A. Schweiger, *J. Magn. Reson.* **2006**, *178*, 42-55.

[3] K. Hakobyan, C. S. P. McErlean, M. Müllner, *Macromol.* **2020**, *53*, 10357-10365.

[4] S. Wang, L. Yang, F. Liang, Y. Zhong, X. Liu, Q. Wang, D. Zhu, *Chem. Sci.* **2023**, *14*, 9197-9206.

[5] C. G. Na, D. Ravelli, E. J. Alexanian, *J. Am. Chem. Soc.* **2020**, *142*, 44-49.

[6] M. J. Mio, L. C. Kopel, J. B. Braun, T. L. Gadzikwa, K. L. Hull, R. G. Brisbois, C. J. Markworth, P. A. Grieco, *Org. Lett.* **2002**, *4*, 3199-3202.

[7] M. Huai, L. Chen, W. Dong, W. Wang, Z. Qin, K. Dai, Y. Li, X. Zhang, C. Tao, *Org. Biomol. Chem.* **2024**, *22*, 5385-5392.

[8] A. Suárez-Lustres, N. Martínez-Yáñez, Á. Velasco-Rubio, J. A. Varela, C. Saá, *Org. Lett.* **2023**, *25*, 794-799.

[9] C. Tang, Z. Xie, *Angew. Chem. Int. Ed.* **2015**, *54*, 7662-7665.

[10] X. Yang, Y. Zhang, B. Zhang, S. Zhang, X. Liu, G. Li, D. Chu, Y. Zhao, G. He, *J. Mater. Chem. C* **2020**, *8*, 16326-16332.

[11] C. Tang, J. Zhang, Z. Xie, *Angew. Chem. Int. Ed.* **2017**, *56*, 8642-8646.

[12] K.-R. Wee, Y.-J. Cho, J. K. Song, S. O. Kang, *Angew. Chem. Int. Ed.* **2013**, *52*, 9682-9685.

[13] C. N. Kona, R. Oku, S. Nakamura, M. Miura, K. Hirano, Y. Nishii, *Chem* **2024**, *10*, 402-413.

[14] W. Lu, Y. Wu, Y.-N. Ma, F. Chen, X. Chen, *Inorg. Chem.* **2023**, *62*, 885-892.

[15] K. Z. Kabytaev, T. A. Everett, A. V. Safronov, Y. V. Sevryugina, S. S. Jalisatgi, M. F. Hawthorne, *Eur. J. Org. Chem.* **2013**, *2013*, 2488-2491.

[16] M. Kellert, D. J. Worm, P. Hoppenz, M. B. Sárosi, P. Lönnecke, B. Riedl, J. Koebberling, A. G. Beck-Sickinger, E. Hey-Hawkins, *Dalton Trans.* **2019**, *48*, 10834-10844.

[17] L. Tai, L. Chen, Y. Shi, L.-A. Chen, *Org. Chem. Front.* **2023**, *10*, 2505-2516.

[18] H. A. Mills, J. L. Martin, A. L. Rheingold, A. M. Spokoyny, *J. Am. Chem. Soc.* **2020**, *142*, 4586-4591.

[19] A. Artaryan, A. Mardyukov, K. Kulbitski, I. Avigdori, G. A. Nisnevich, P. R. Schreiner, M. Gandelman, *J. Org. Chem.* **2017**, *82*, 7093-7100.

S7. NMR Spectra

^{1}H-NMR spectra were recorded using an NMR Bruker AdvanceNeo 400 MHz and/or NMR Jeol ECZR 600 MHz in $CDCl_3$. ^{13}C-NMR spectra were measured with complete proton decoupling (notation ^{13}C{^{1}H}-NMR). DEPT experiments were carried out with a DEPT-135 sequence. ^{11}B-NMR were measured with complete proton decoupling (notation ^{11}B{^{1}H}-NMR). ^{19}F-NMR were measured with complete proton decoupling (notation ^{19}F{^{1}H}-NMR).

For compounds already reported in the literature, ^{1}H-NMR spectra were reported.

For each new compound, ^{1}H-NMR, ^{13}C{^{1}H}-NMR, DEPT-135, 2D – COSY and, when applicable, ^{11}B{^{1}H}-NMR and ^{19}F{^{1}H}-NMR spectra were reported.

Data were reported as follows: chemical shifts in ppm from Me_4Si as an internal standard, multiplicity, coupling constants (Hz), integration and assignment. Chemical shifts were reported in ppm from the residual solvent peak. Spectra are referenced to the residual solvent peak.

Annotations in spectra:

- Residual solvent peaks intrinsic to the deuterated solvent used are not notated in ^{1}H-NMR ($CHCl_3$ at 7.26 ppm, moisture at 1.56 ppm).
- Residual grease was found to be inseparable from most xanthyl carboranes due to their high lipophilic nature. The peaks are notated in ^{1}H-NMR (0.88, 1.26 ppm), ^{13}C-NMR and DEPT-135 spectra (29.8 ppm).
- In the case of a mixture of isomers:
  When a mixture enriched on one major isomer was found:
  § annotation indicates a peak of the major isomer
  # annotation indicates a peak of the minor isomer(s)
  When peaks could not be clearly assigned to a specific major isomer, no annotations were given.

^{1}H-NMR (400 MHz, $CDCl_3$)

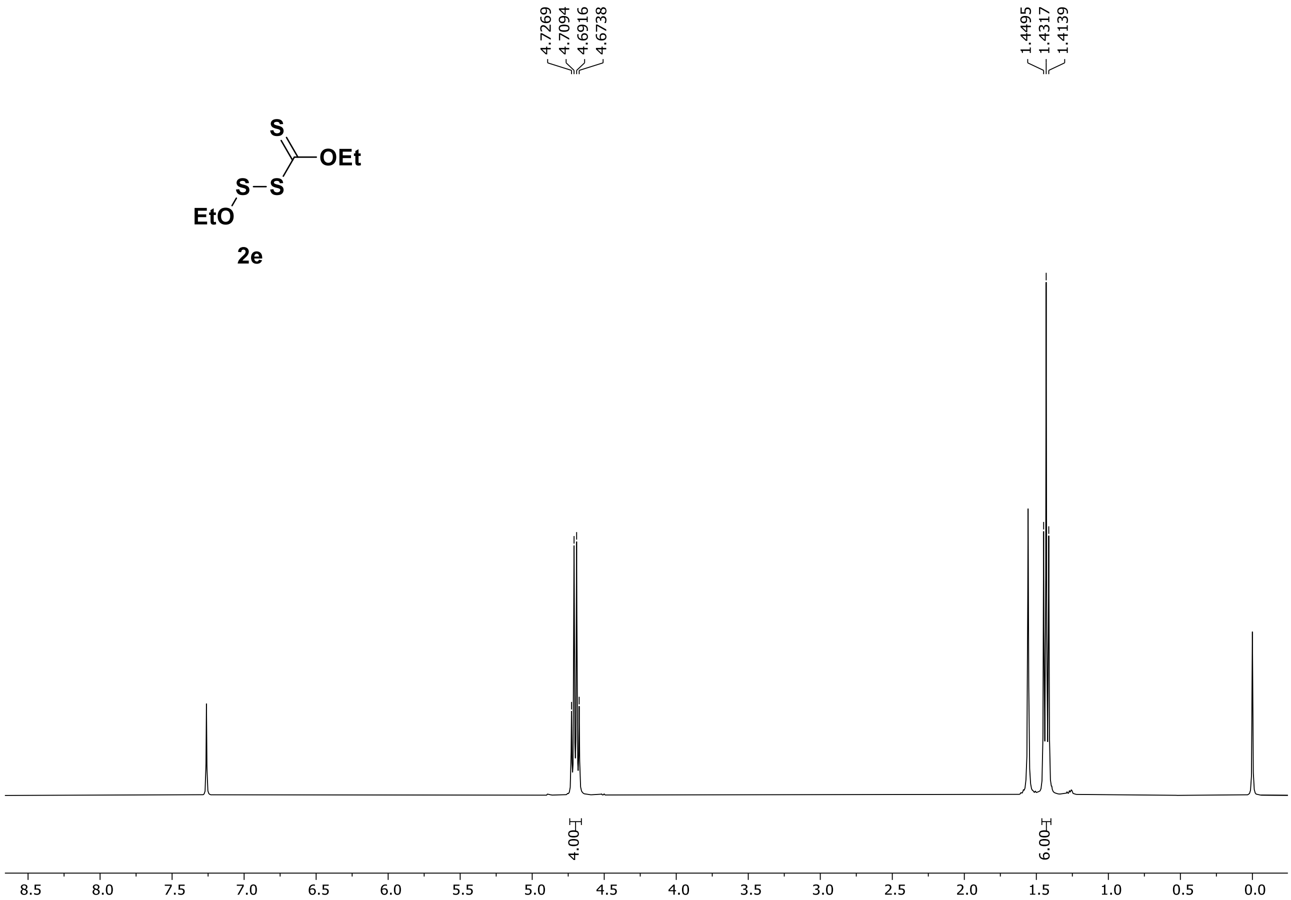

^{1}H-NMR (400 MHz, $CDCl_3$)

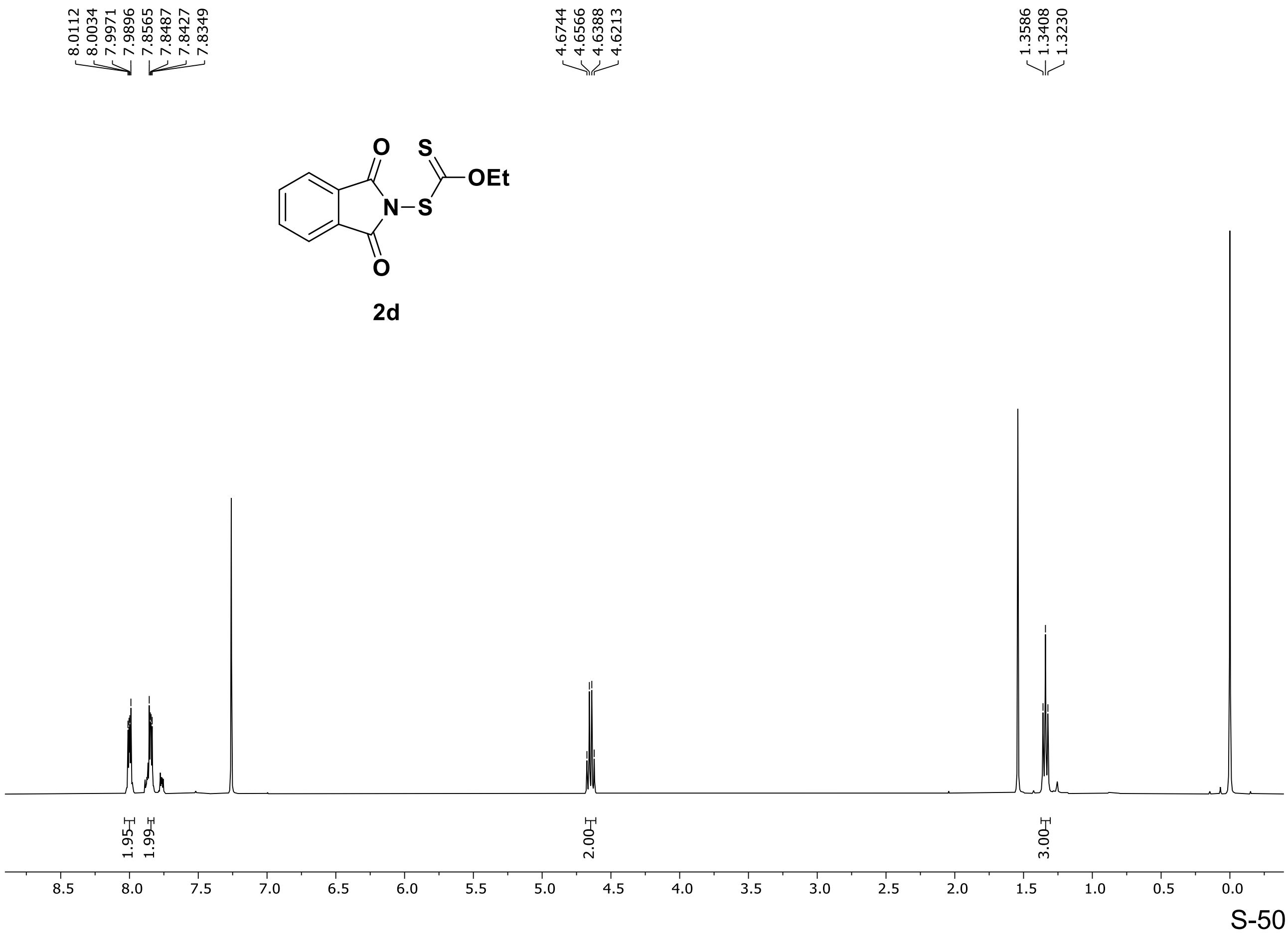

^{1}**H-NMR** (400 MHz, $CDCl_3$)

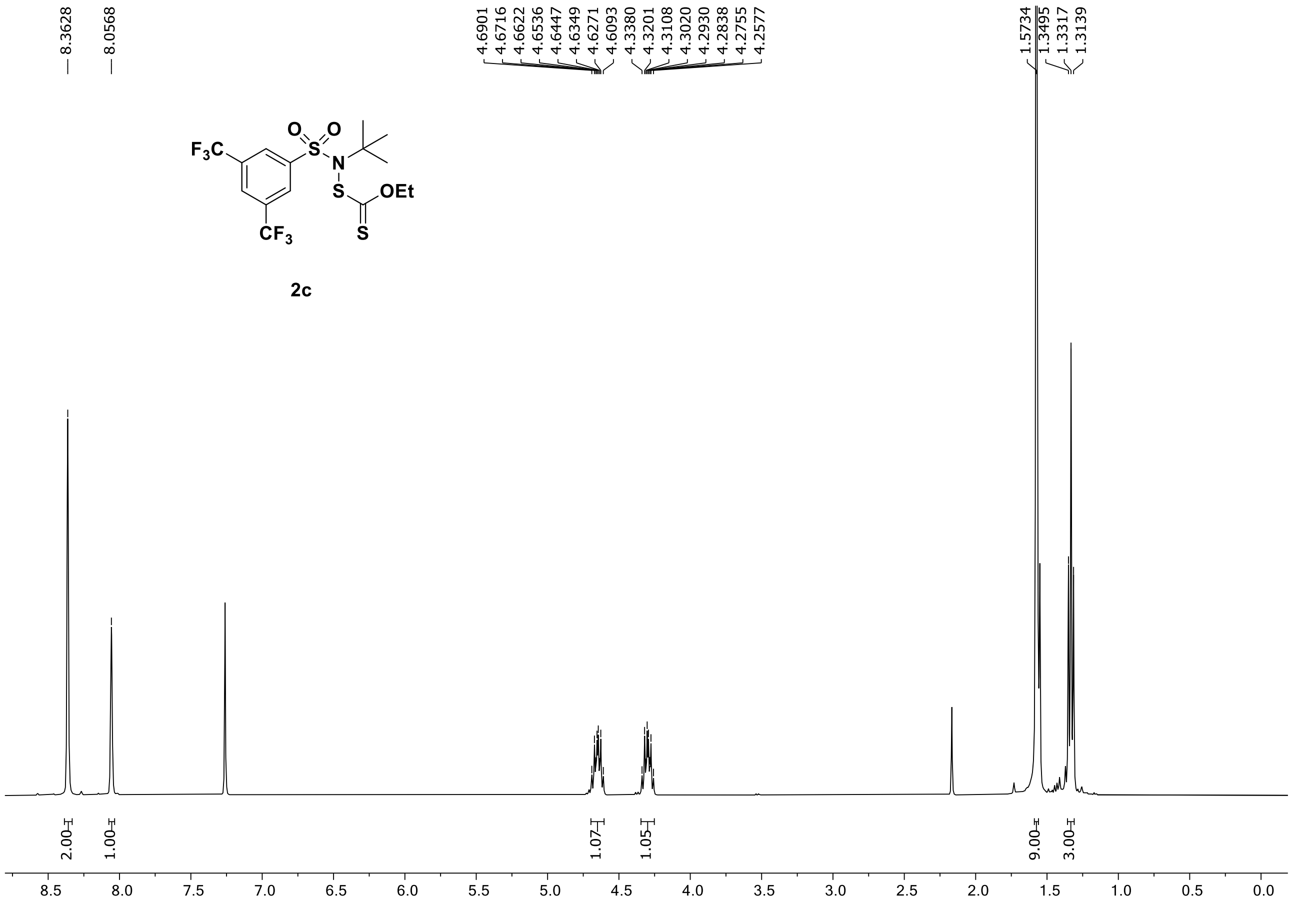

^{1}**H-NMR** (400 MHz, $CDCl_3$)

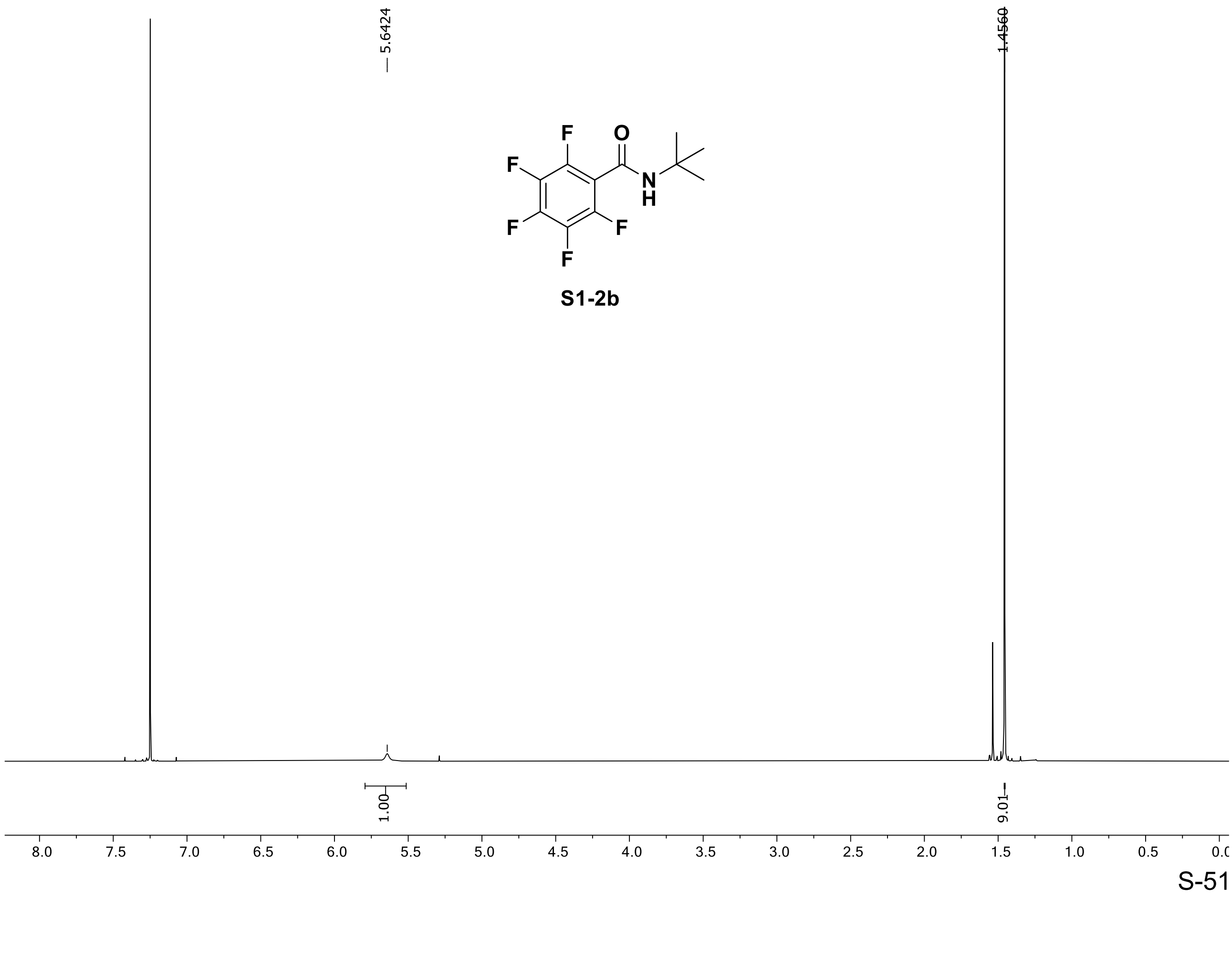

^{1}H-NMR (400 MHz, $CDCl_3$)

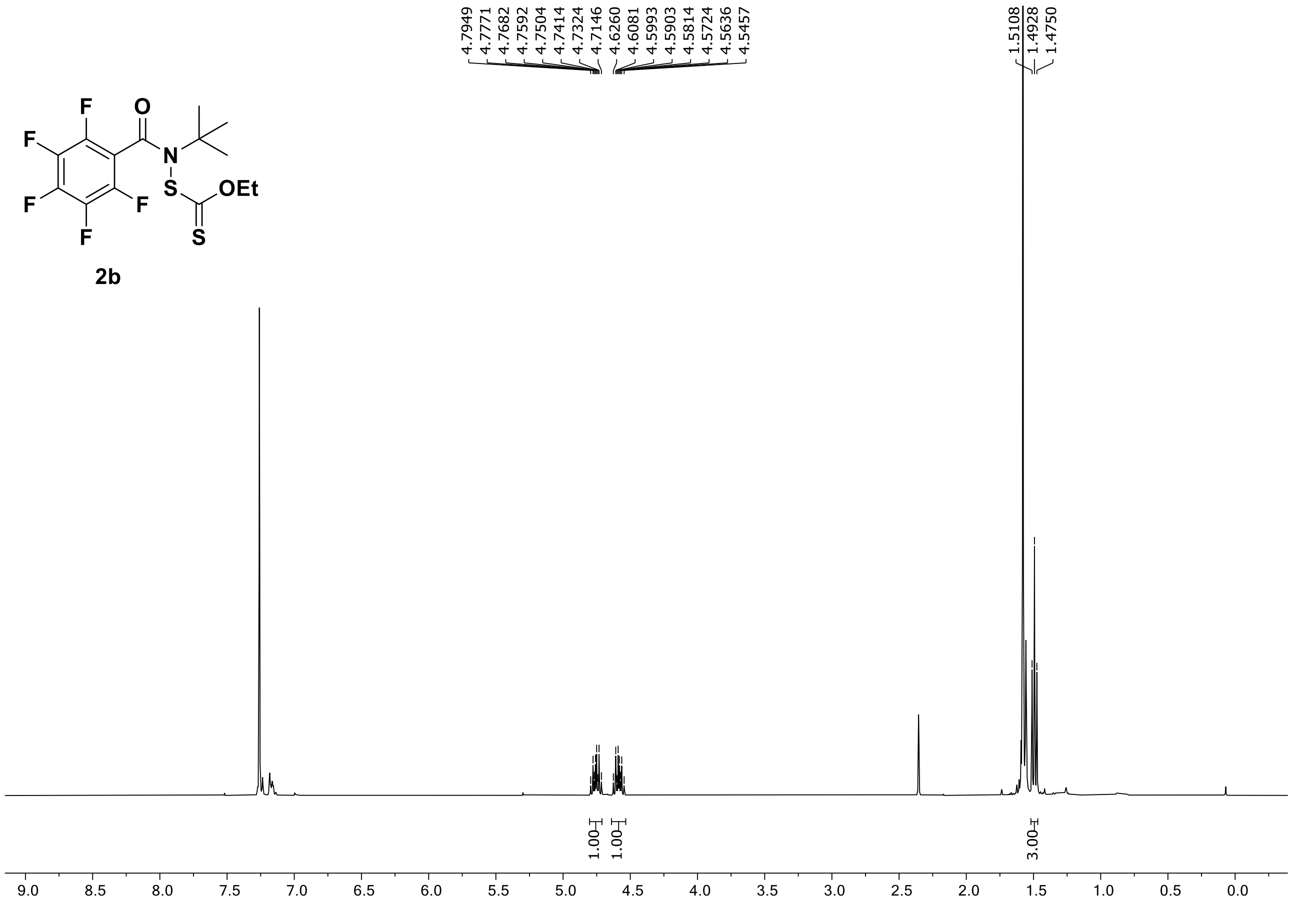

^{1}H-NMR (400 MHz, $CDCl_3$)

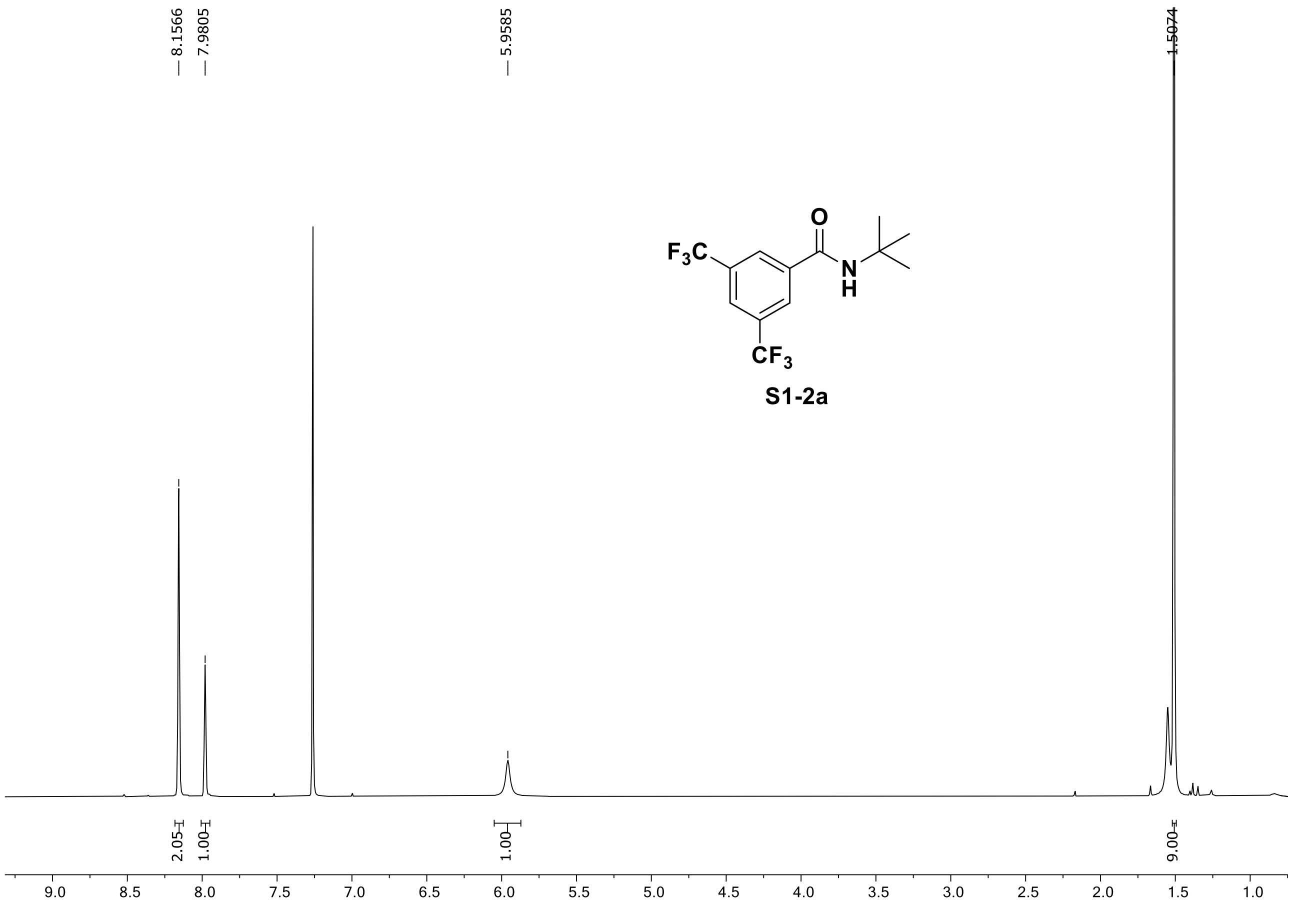

^{1}H-NMR (400 MHz, $CDCl_3$)

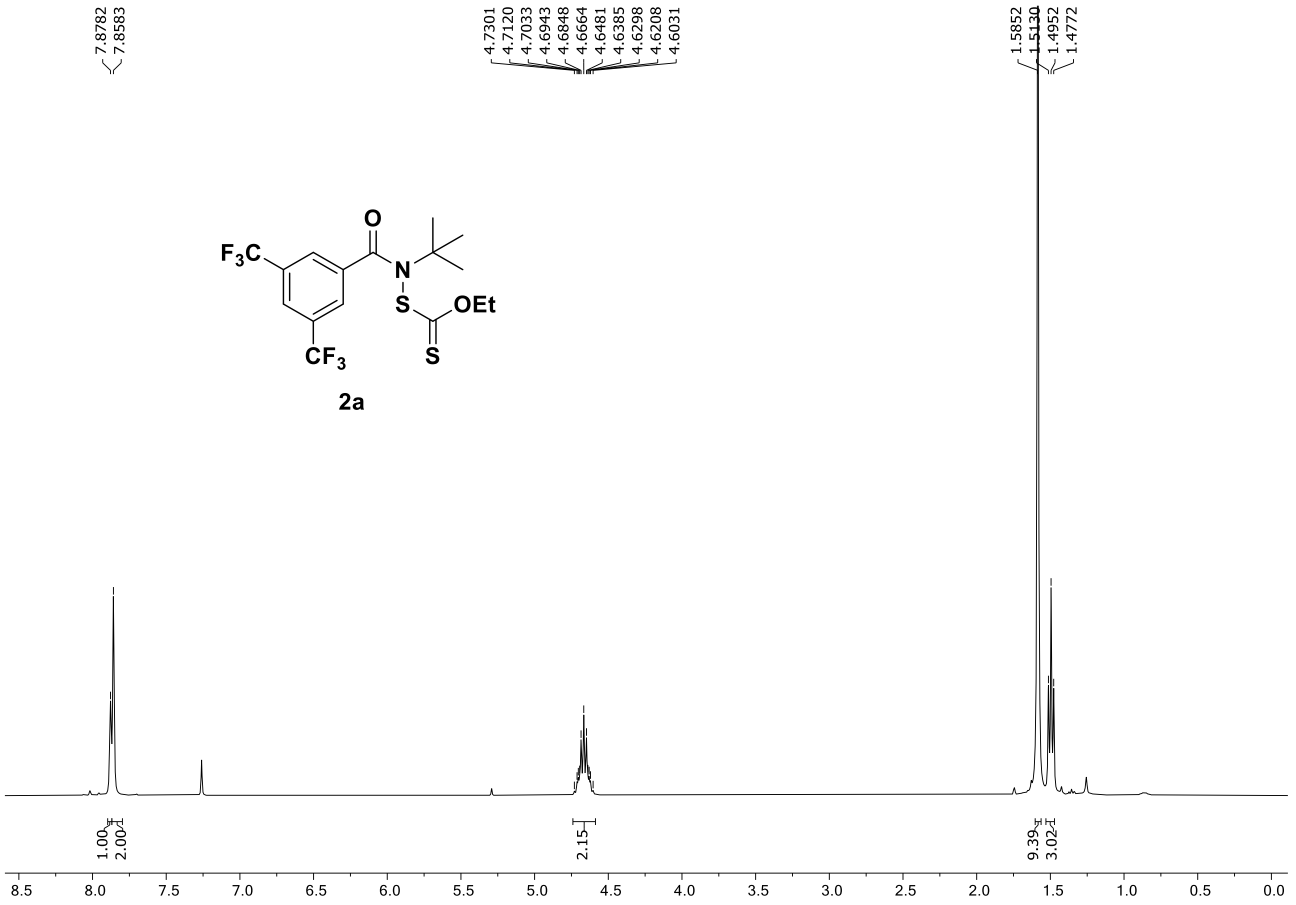

2a

^{1}H-NMR (400 MHz, $CDCl_3$)

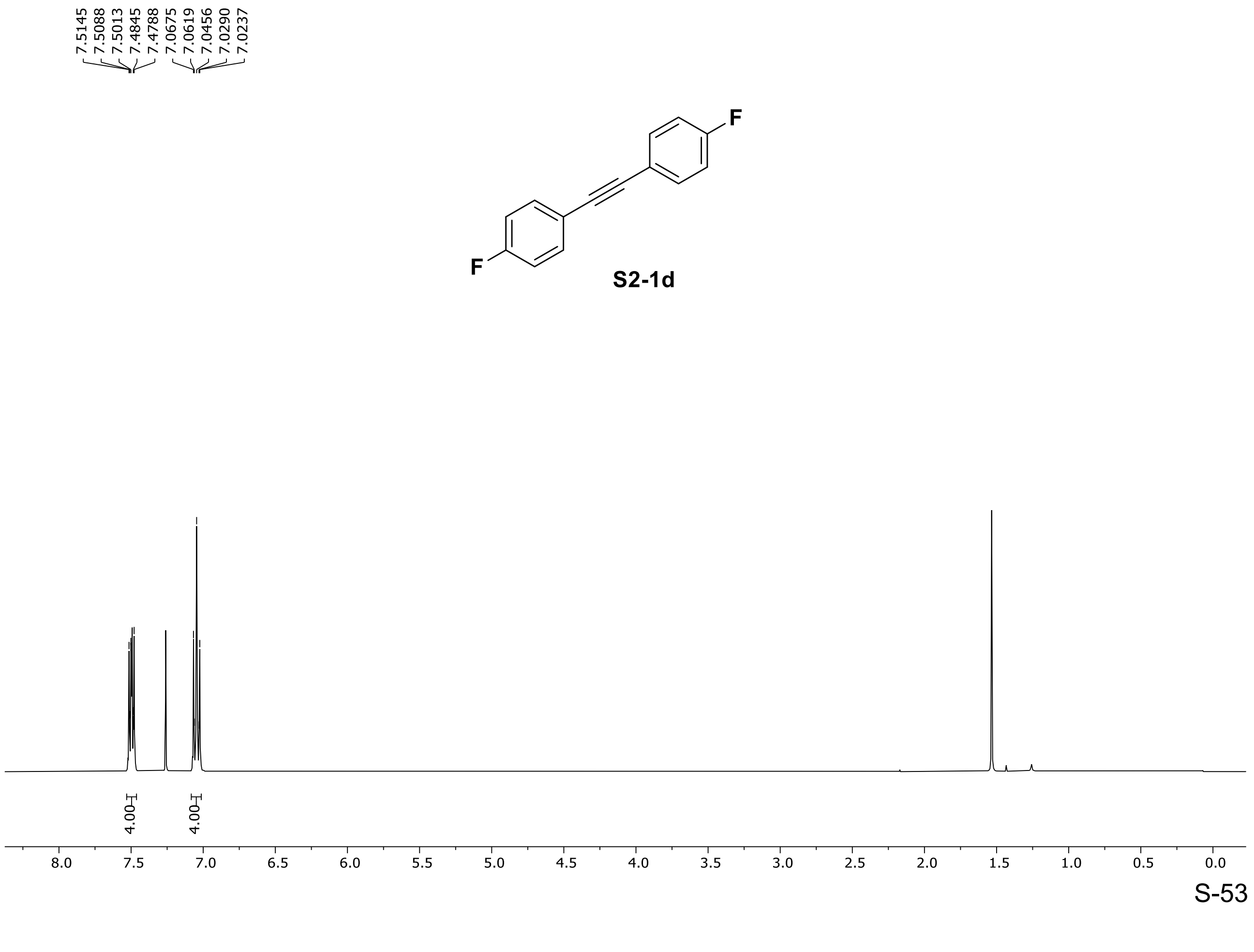

S2-1d

1H-NMR (400 MHz, $CDCl_3$)

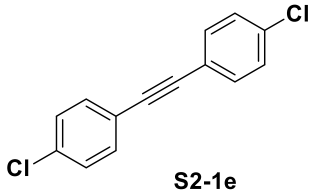

S2-1e

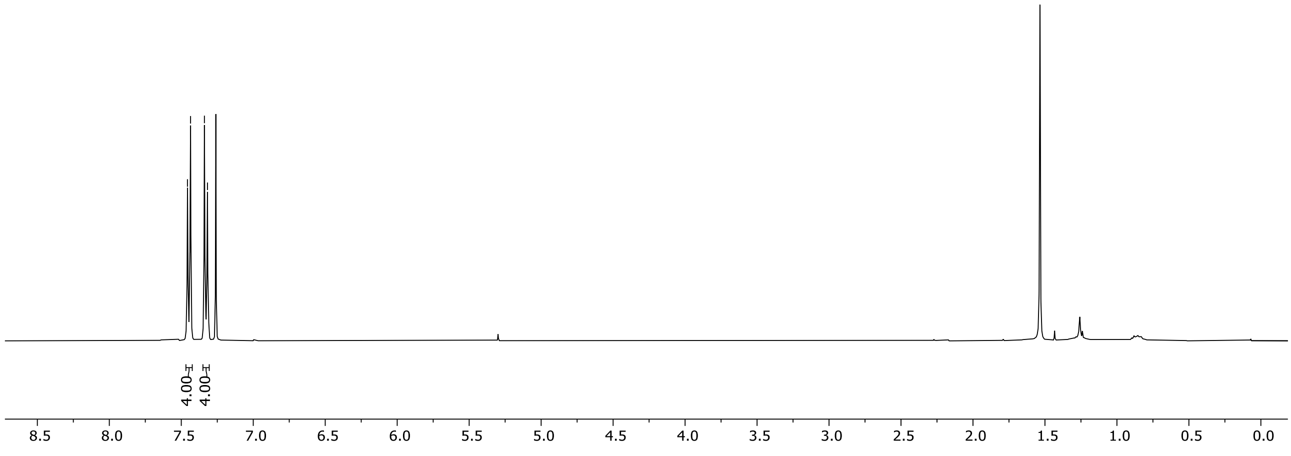

1H-NMR (400 MHz, $CDCl_3$)

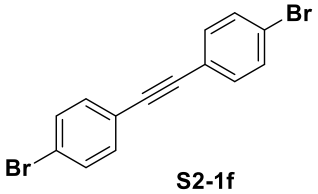

S2-1f

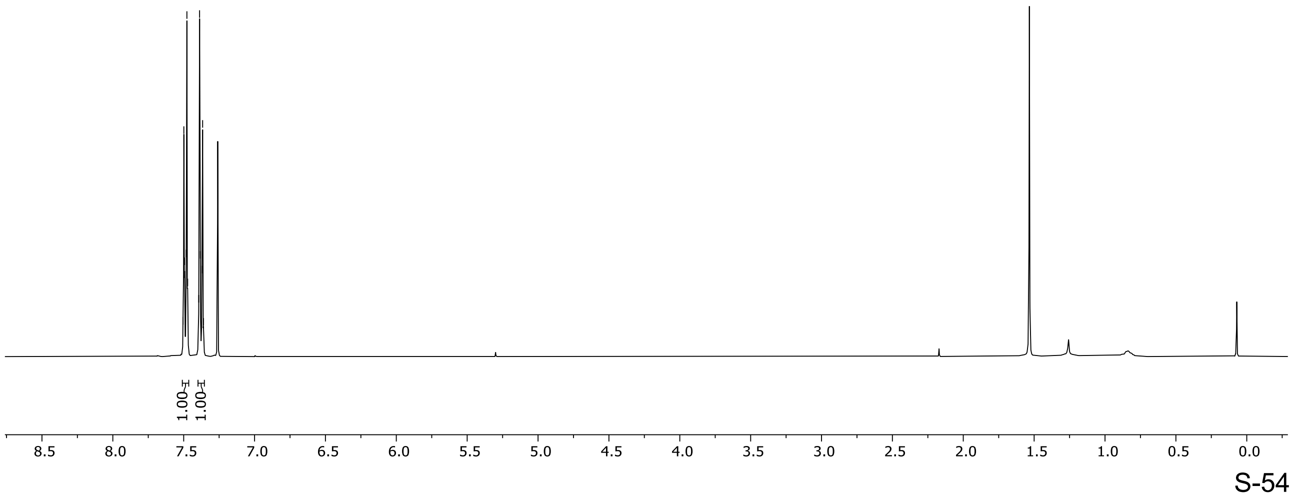

^{1}H-NMR (400 MHz, $CDCl_3$)

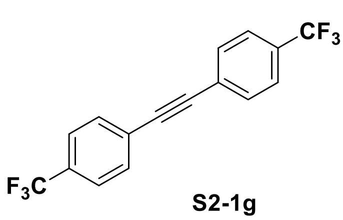

S2-1g

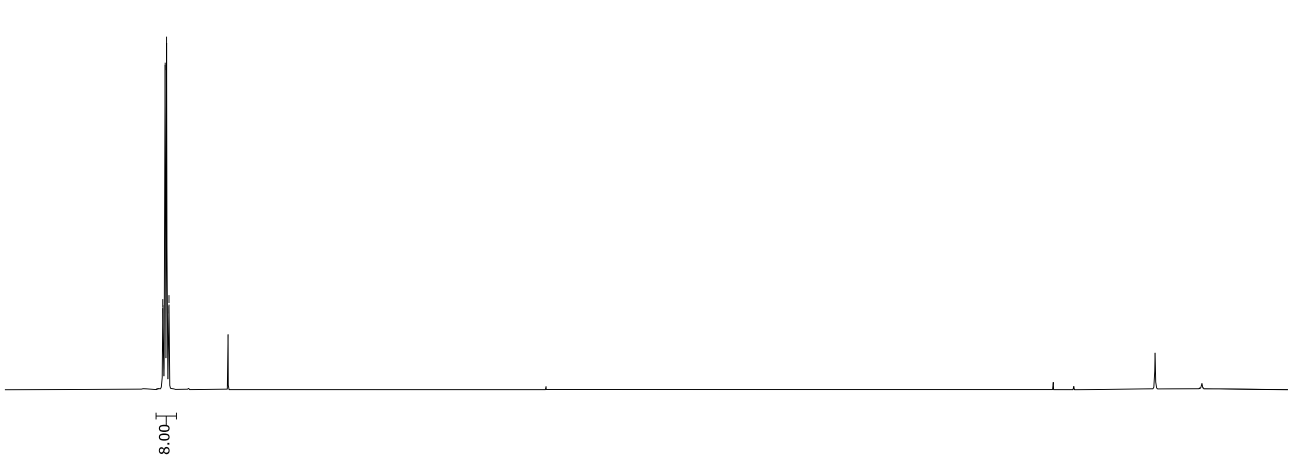

^{1}H-NMR (400 MHz, $CDCl_3$)

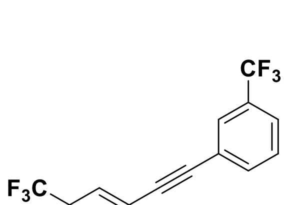

S2-1h

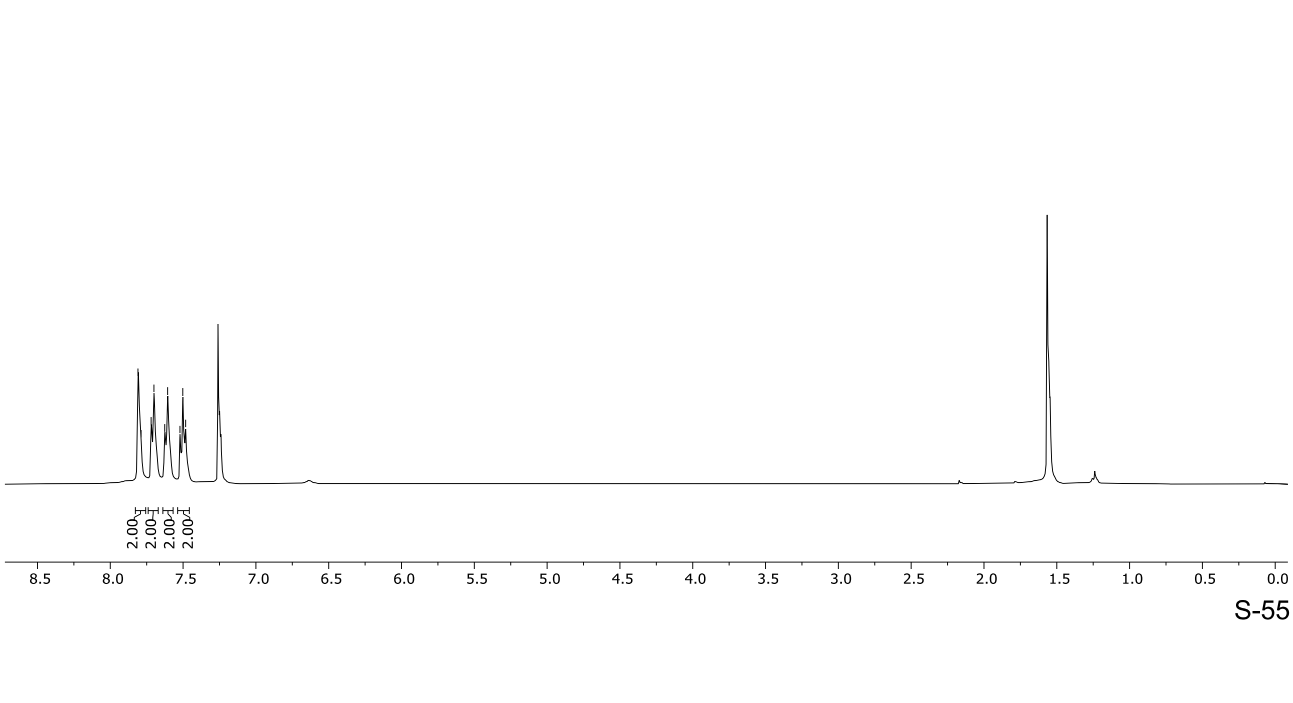

¹H-NMR (400 MHz, $CDCl_3$)

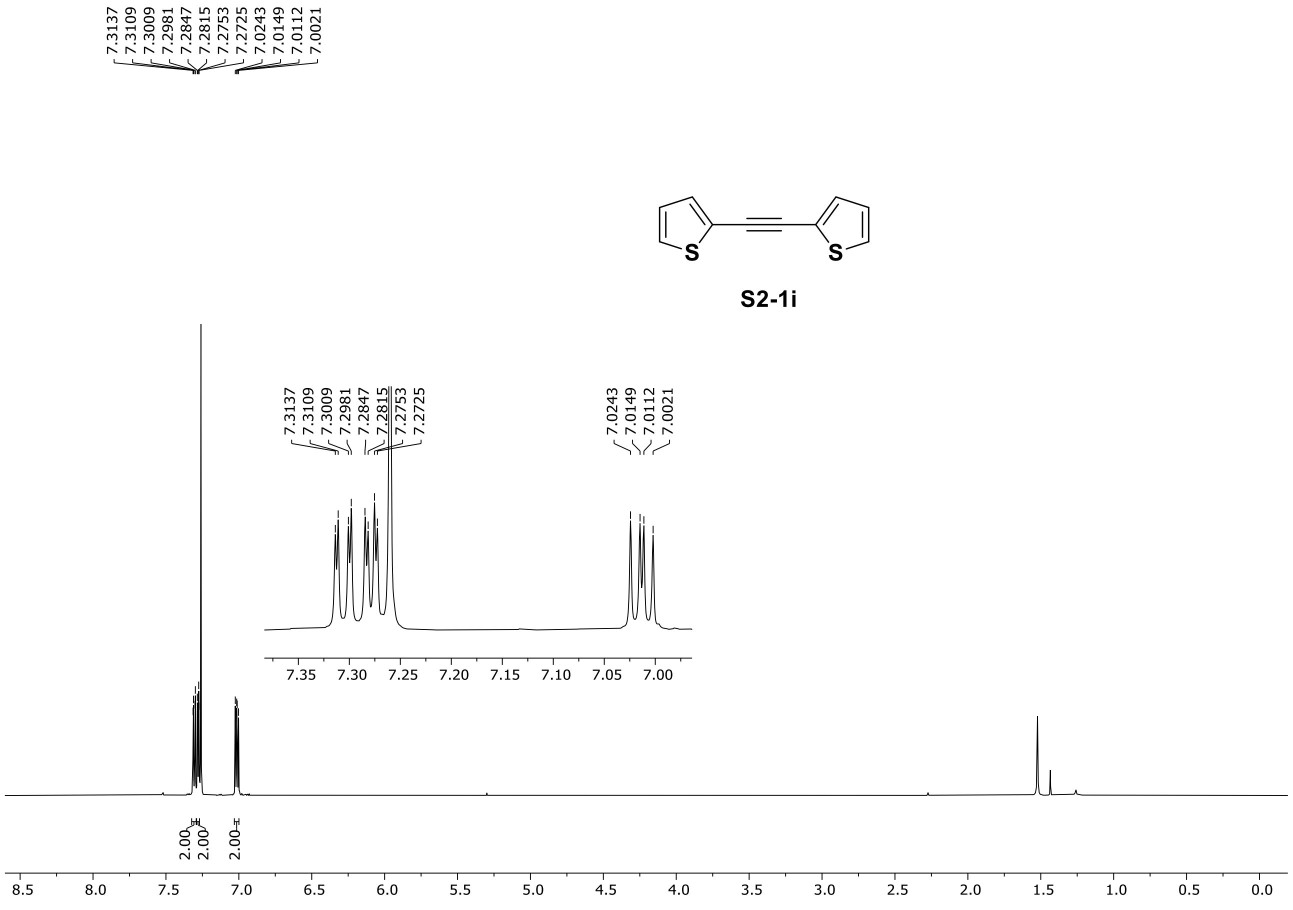

S2-1i

¹H-NMR (400 MHz, $CDCl_3$)

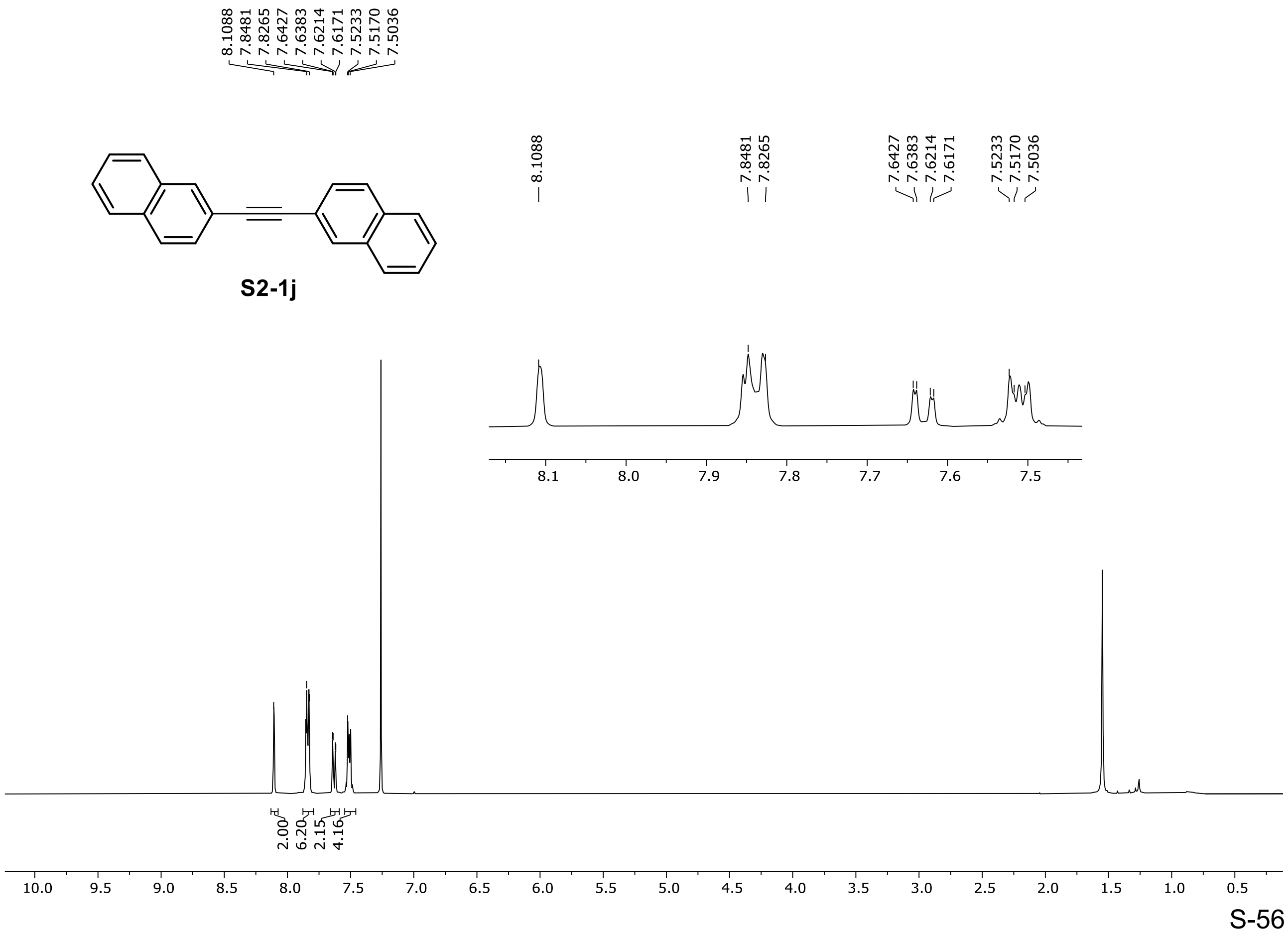

S2-1j

1H-NMR (400 MHz, $CDCl_3$)

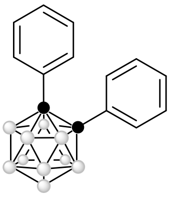

1c

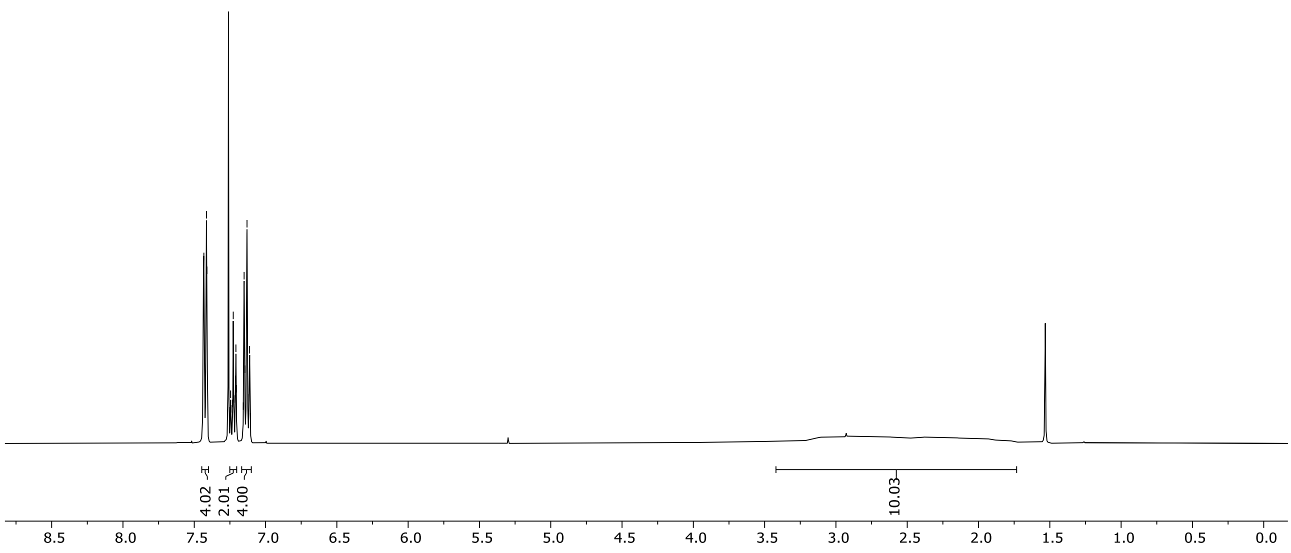

1H-NMR (400 MHz, $CDCl_3$)

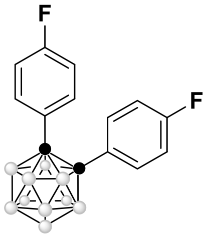

1d

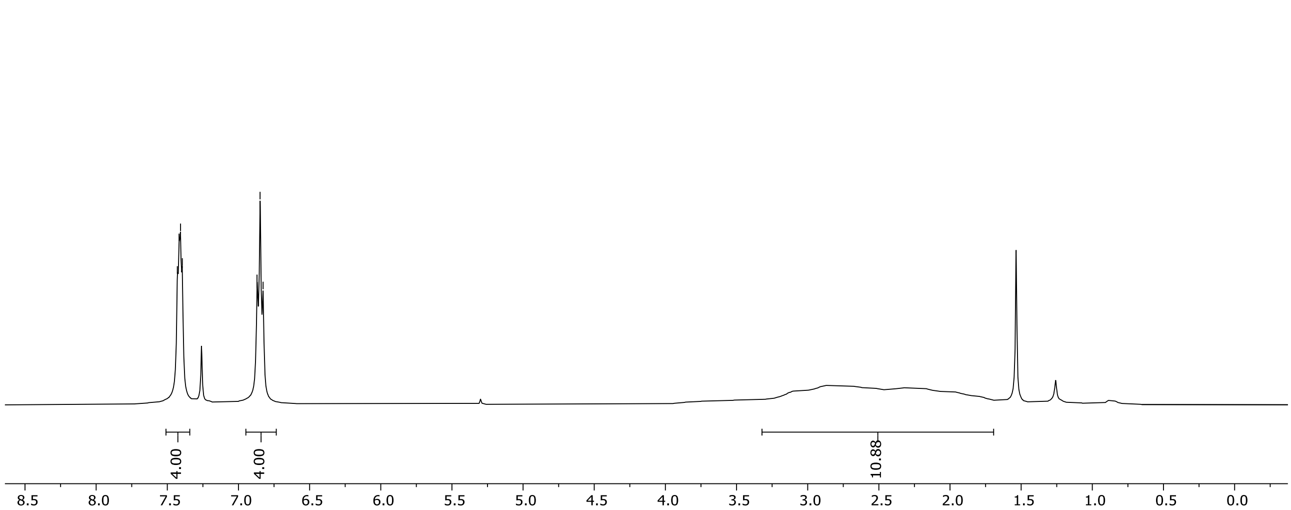

^{1}H-NMR (400 MHz, $CDCl_3$)

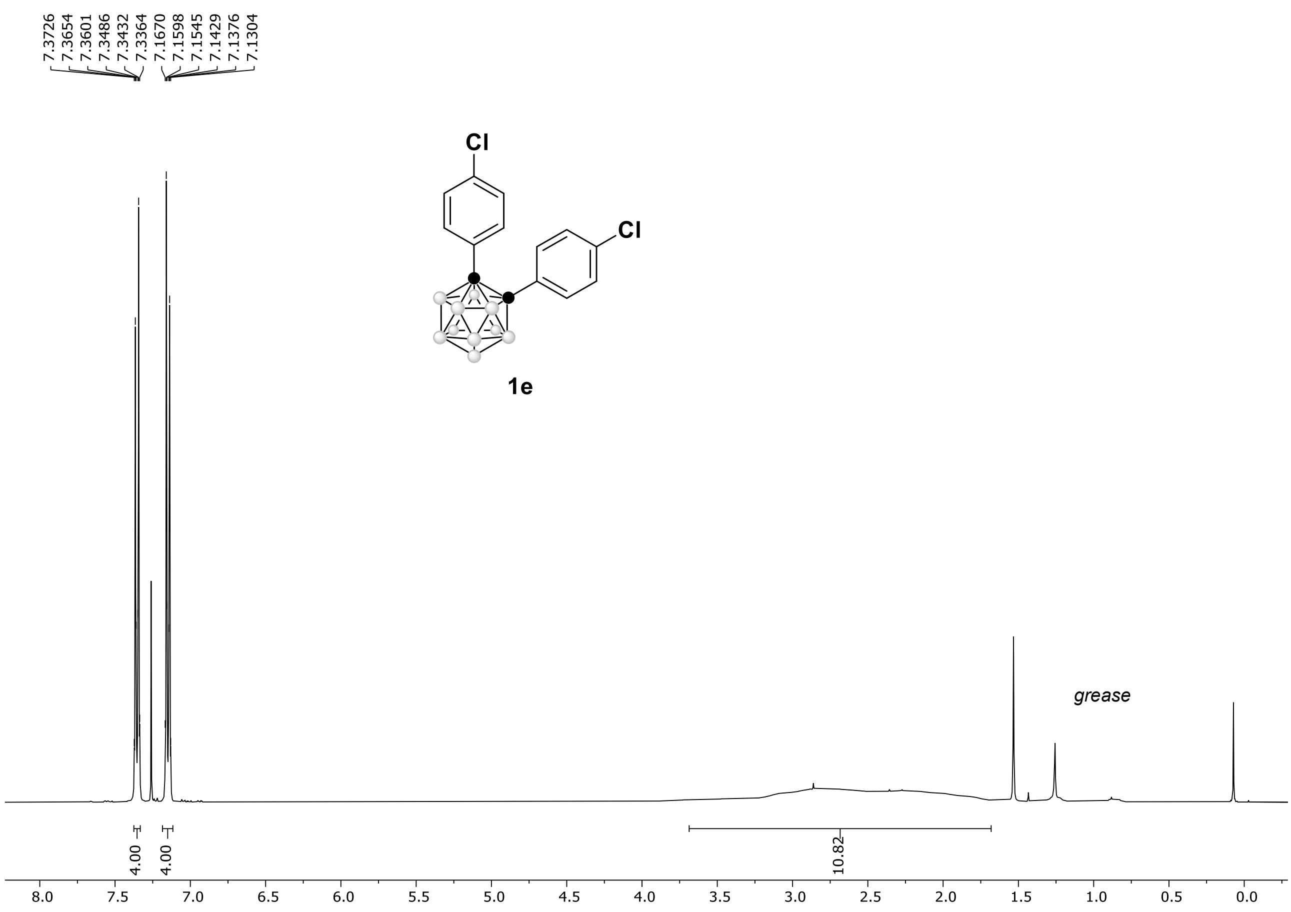

^{1}H-NMR (400 MHz, $CDCl_3$)

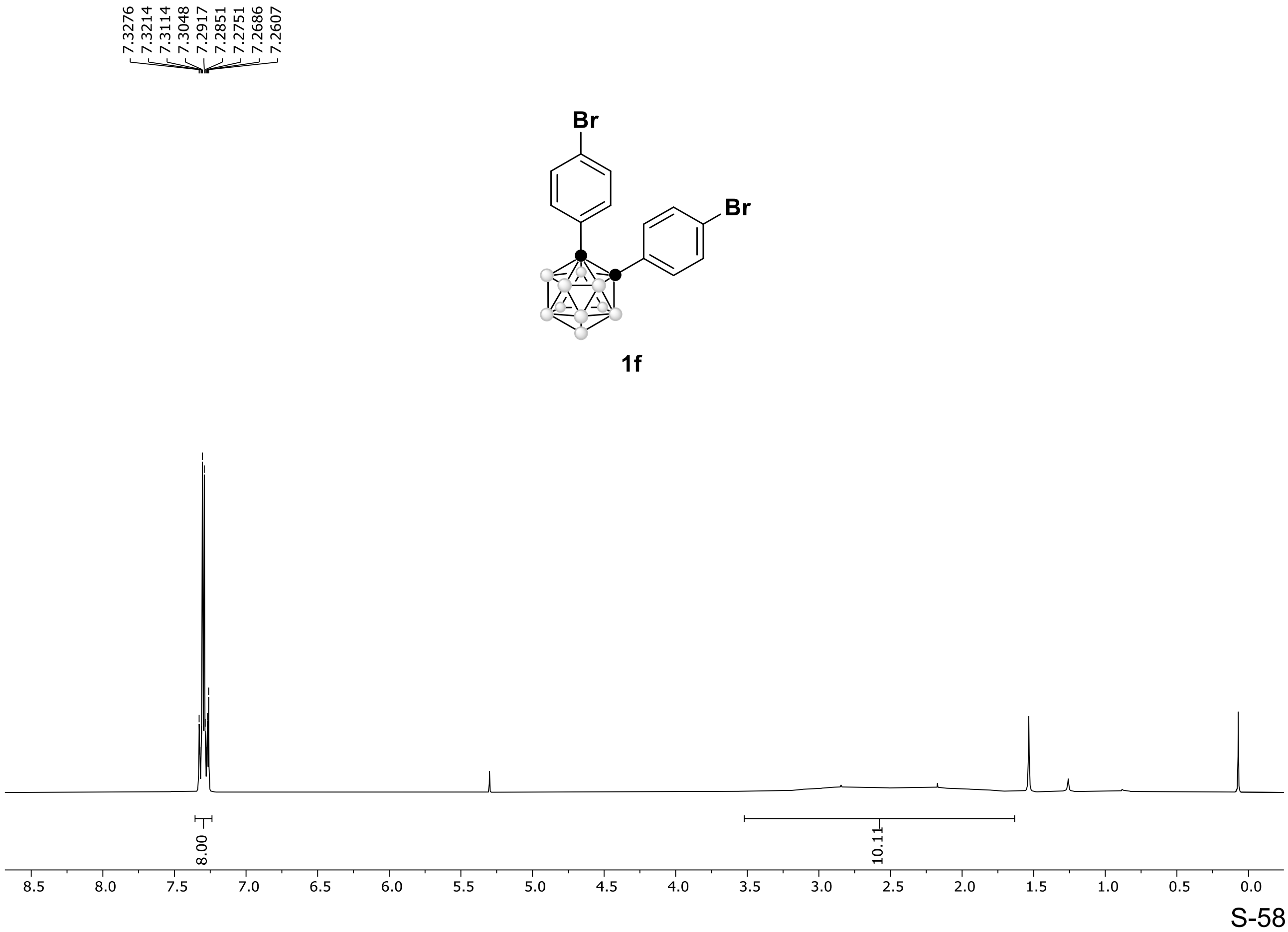

^{1}H-NMR (600 MHz, $CDCl_3$)

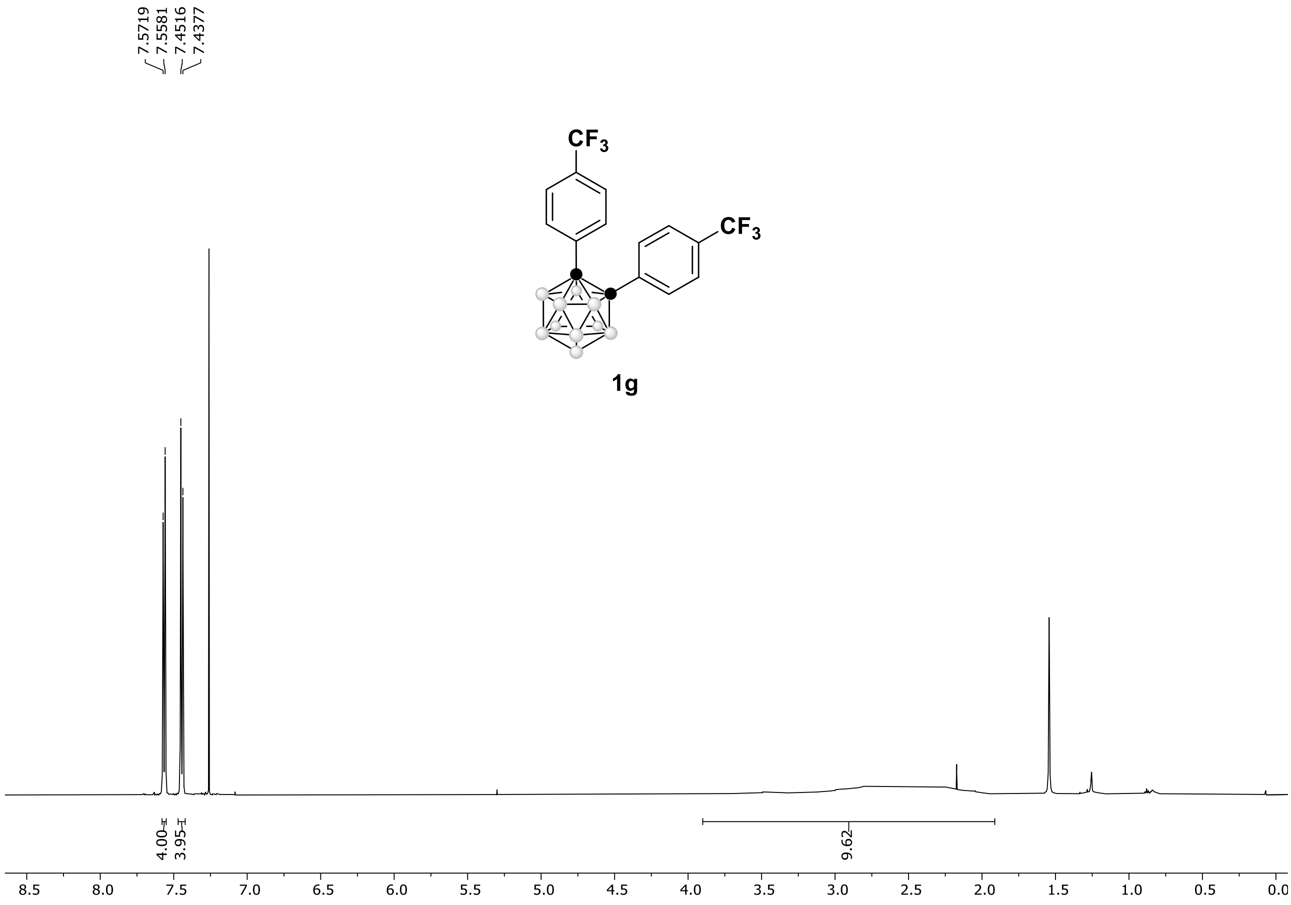

^{1}H-NMR (400 MHz, $CDCl_3$)

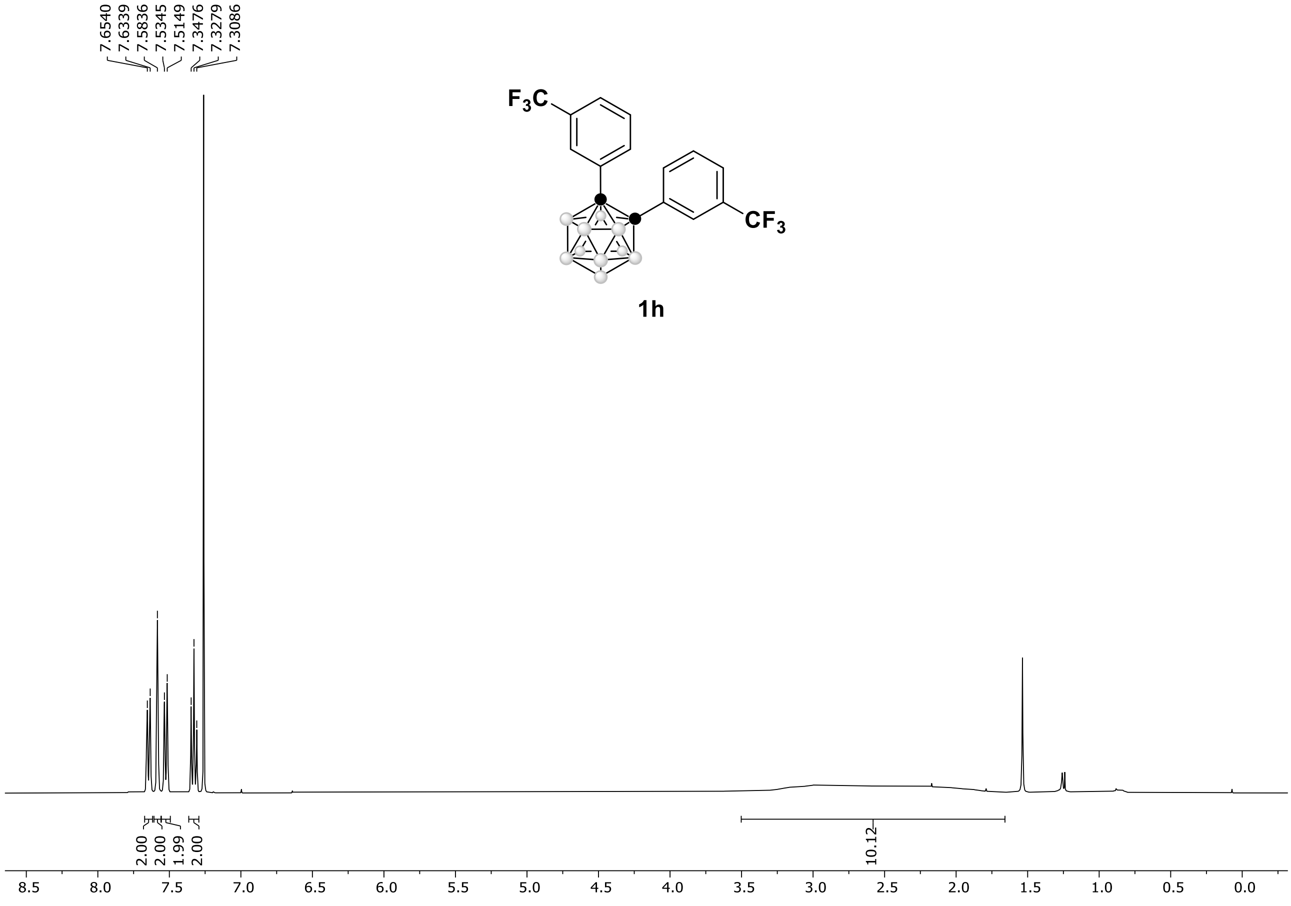

$^{13}C\{^{1}H\}$-NMR (100 MHz, $CDCl_3$)

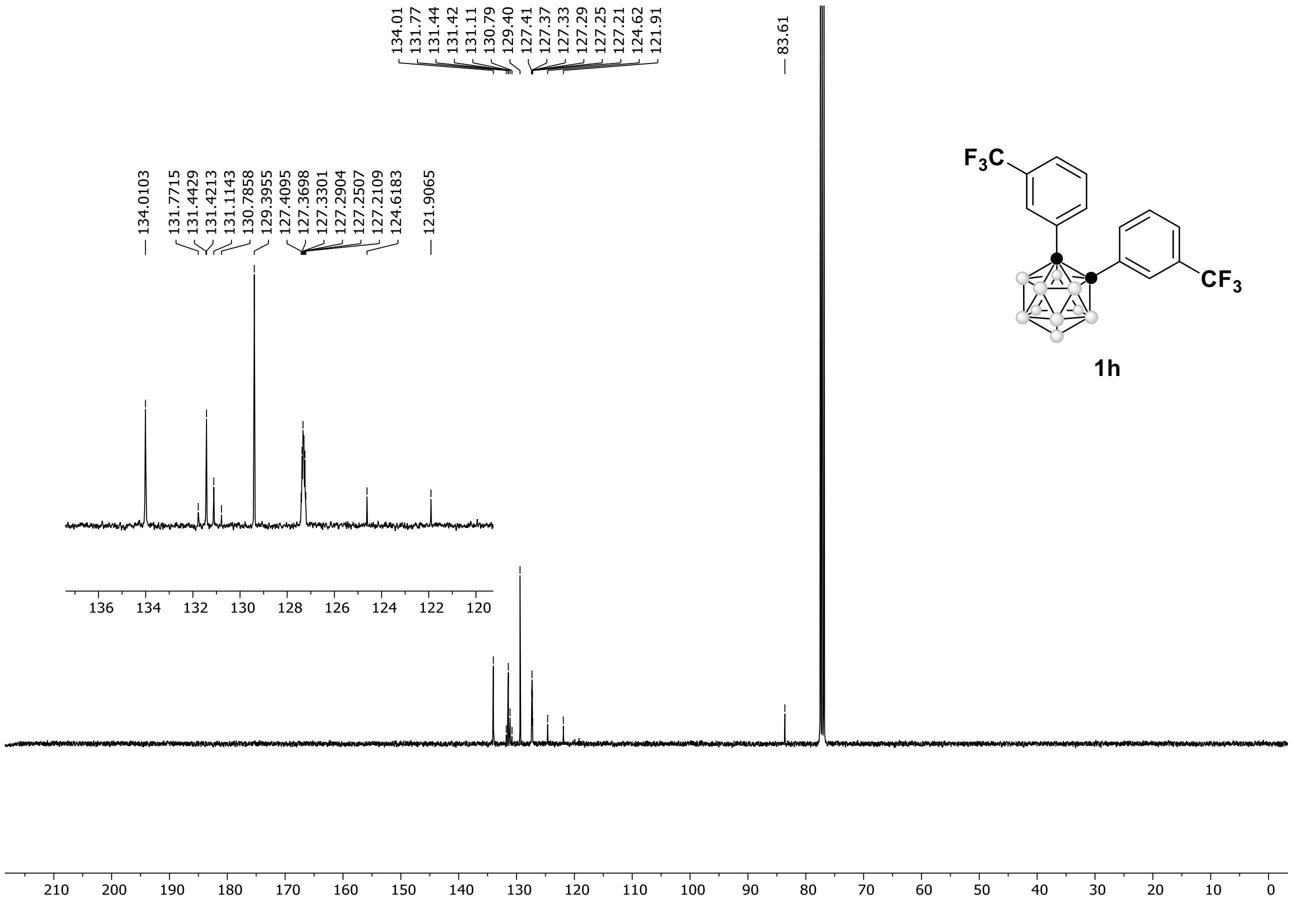

DEPT135 (100 MHz, $CDCl_3$)

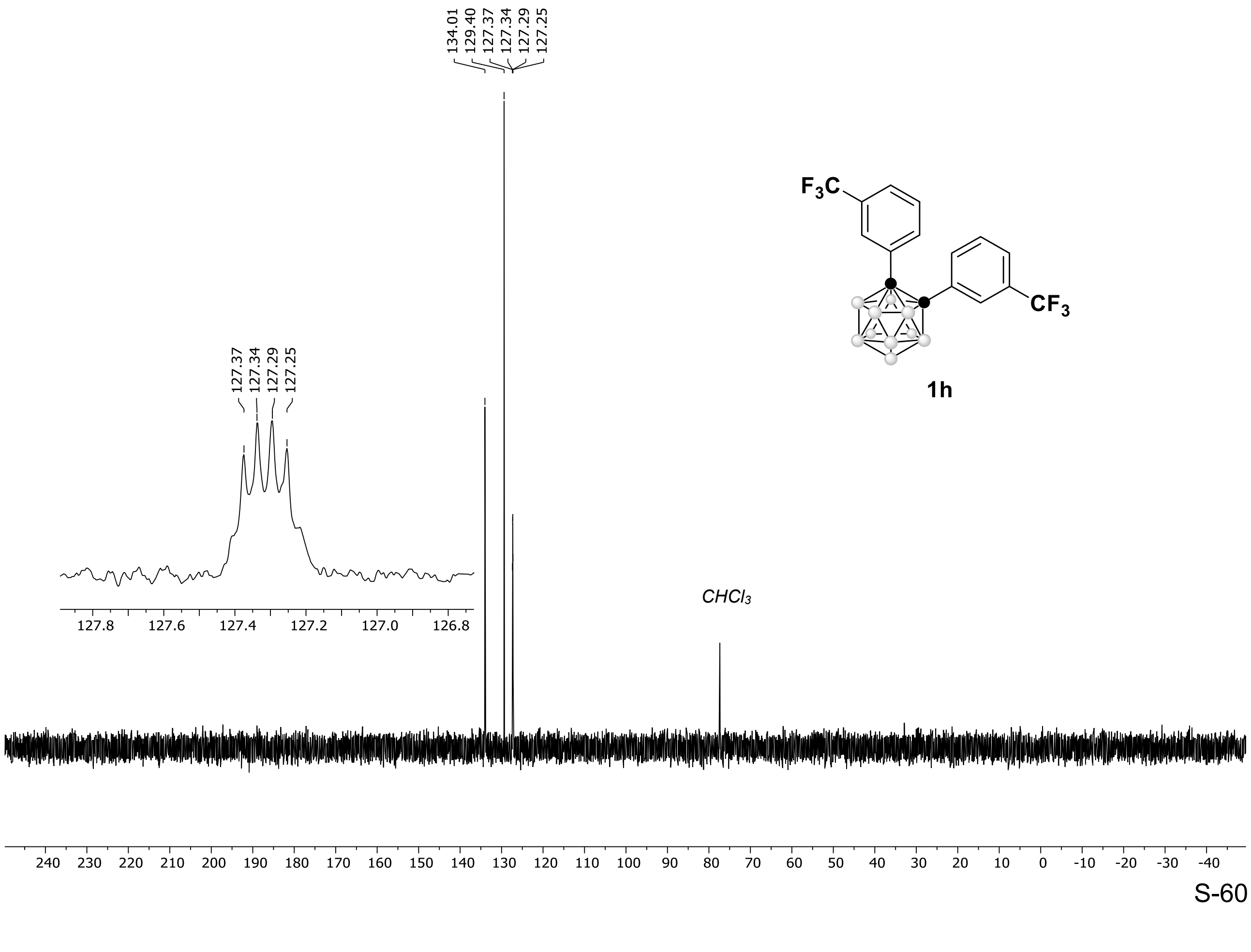

$^{11}B\{^{1}H\}$-NMR (128 MHz, $CDCl_3$)

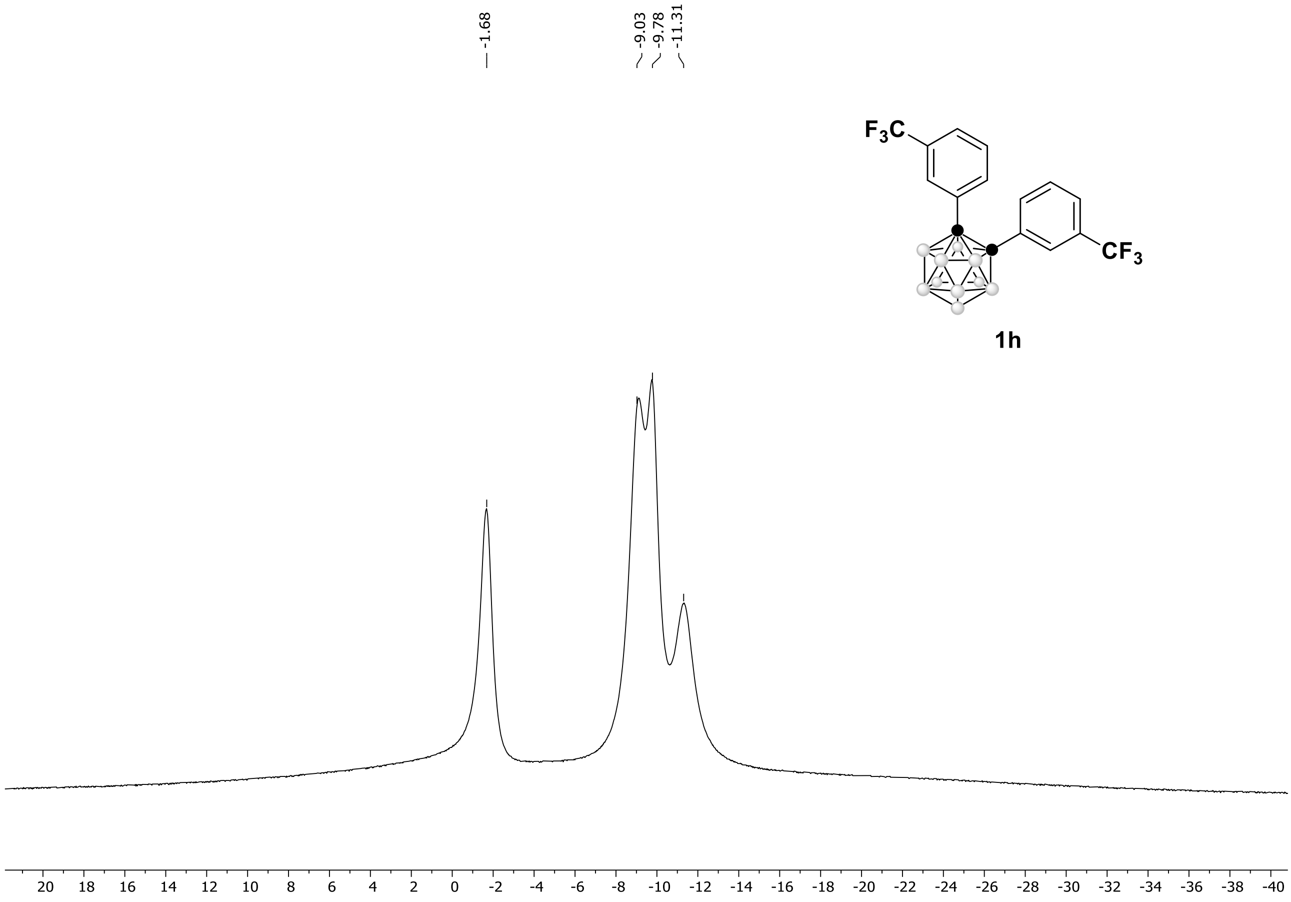

$^{19}F\{^{1}H\}$-NMR (376 MHz, $CDCl_3$)

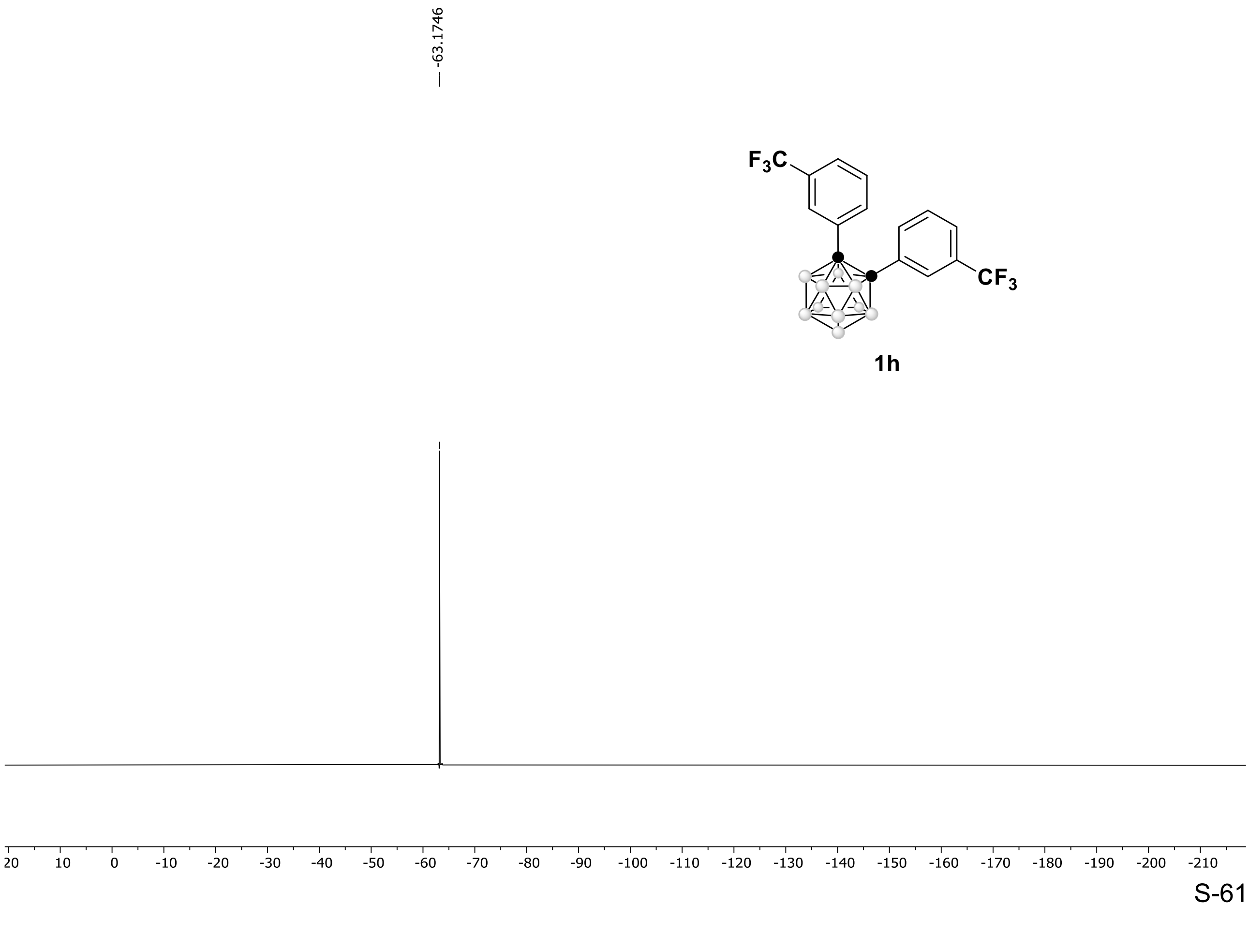

2D – COSY (400 MHz, $CDCl_3$)

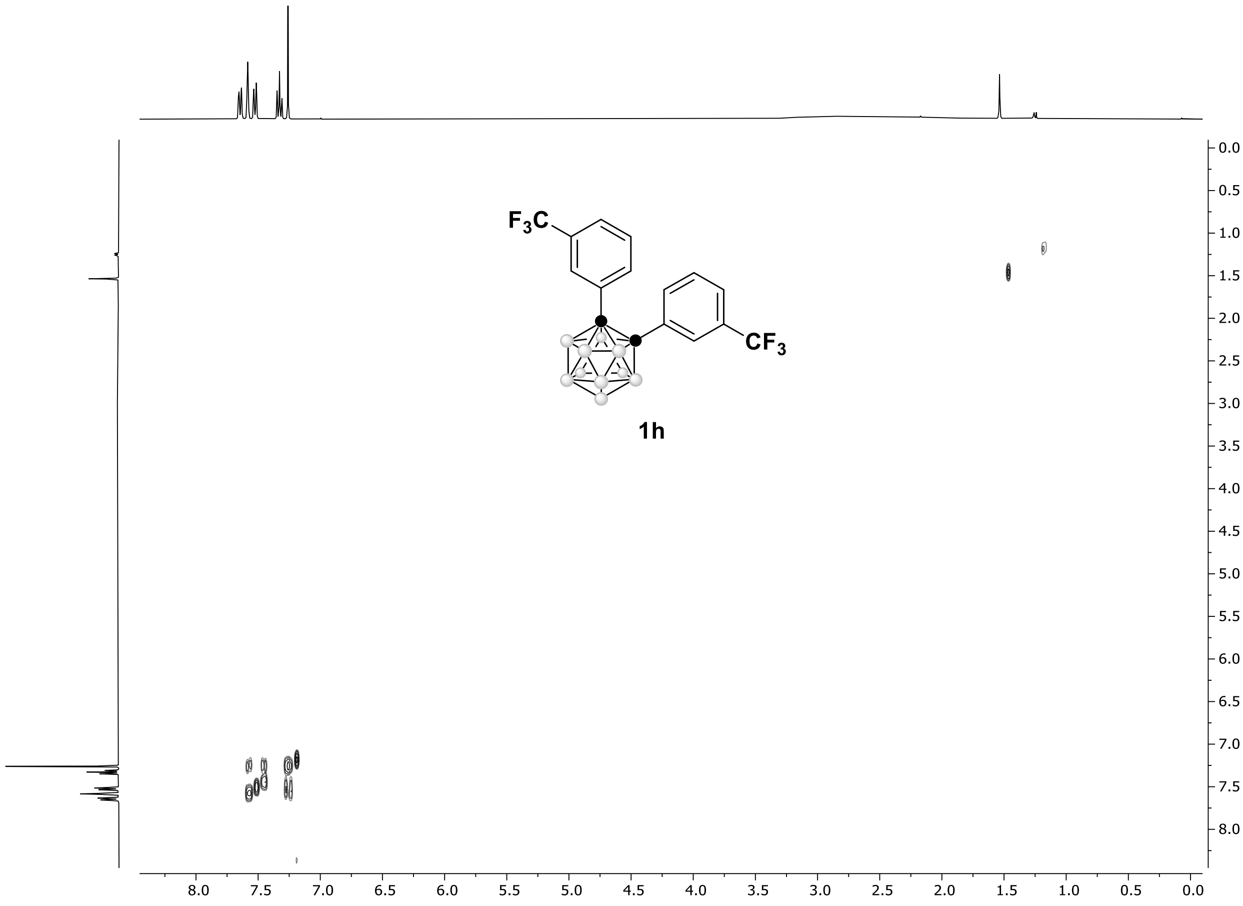

^{1}H-NMR (400 MHz, $CDCl_3$)

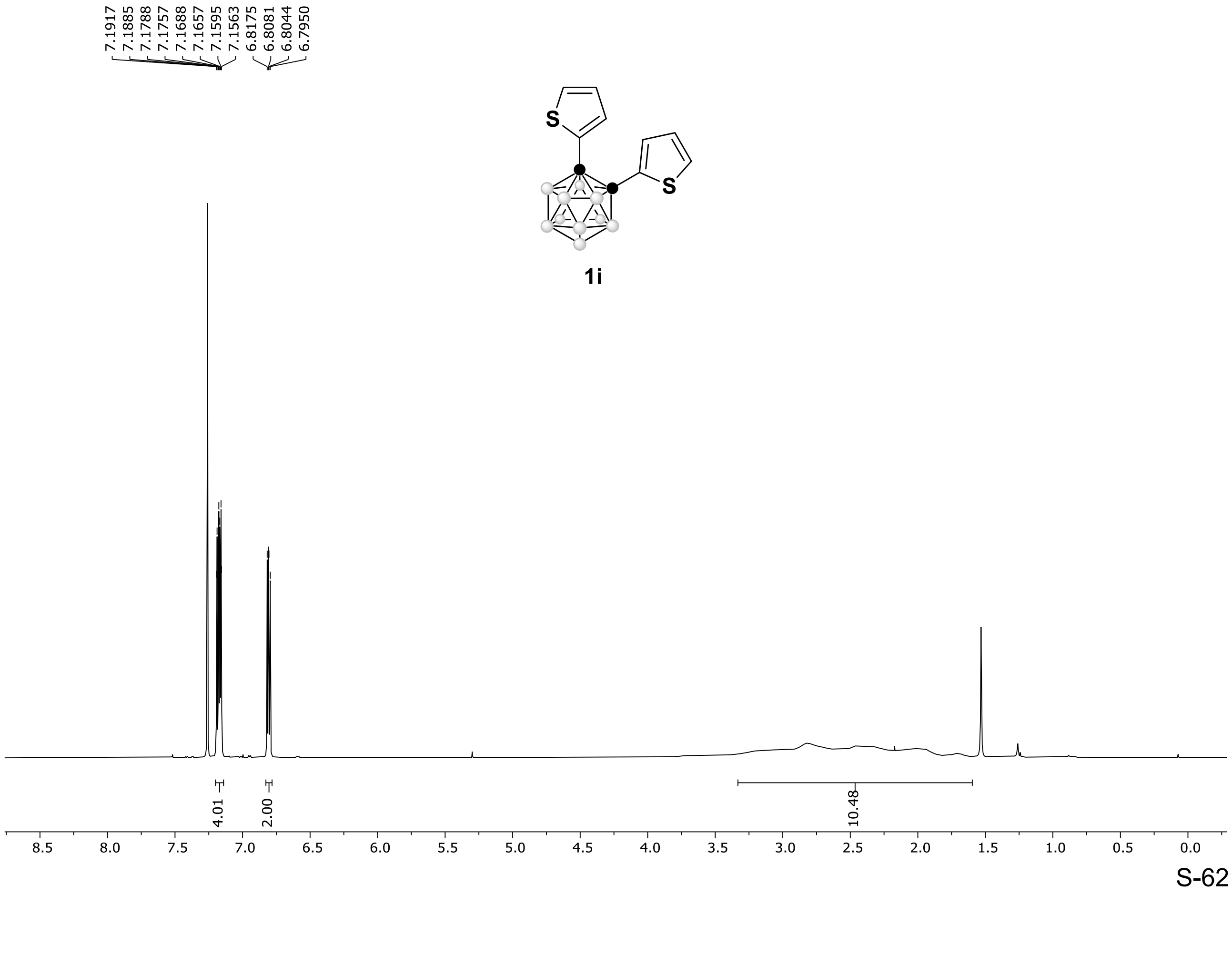

^{1}H-NMR (400 MHz, $CDCl_3$)

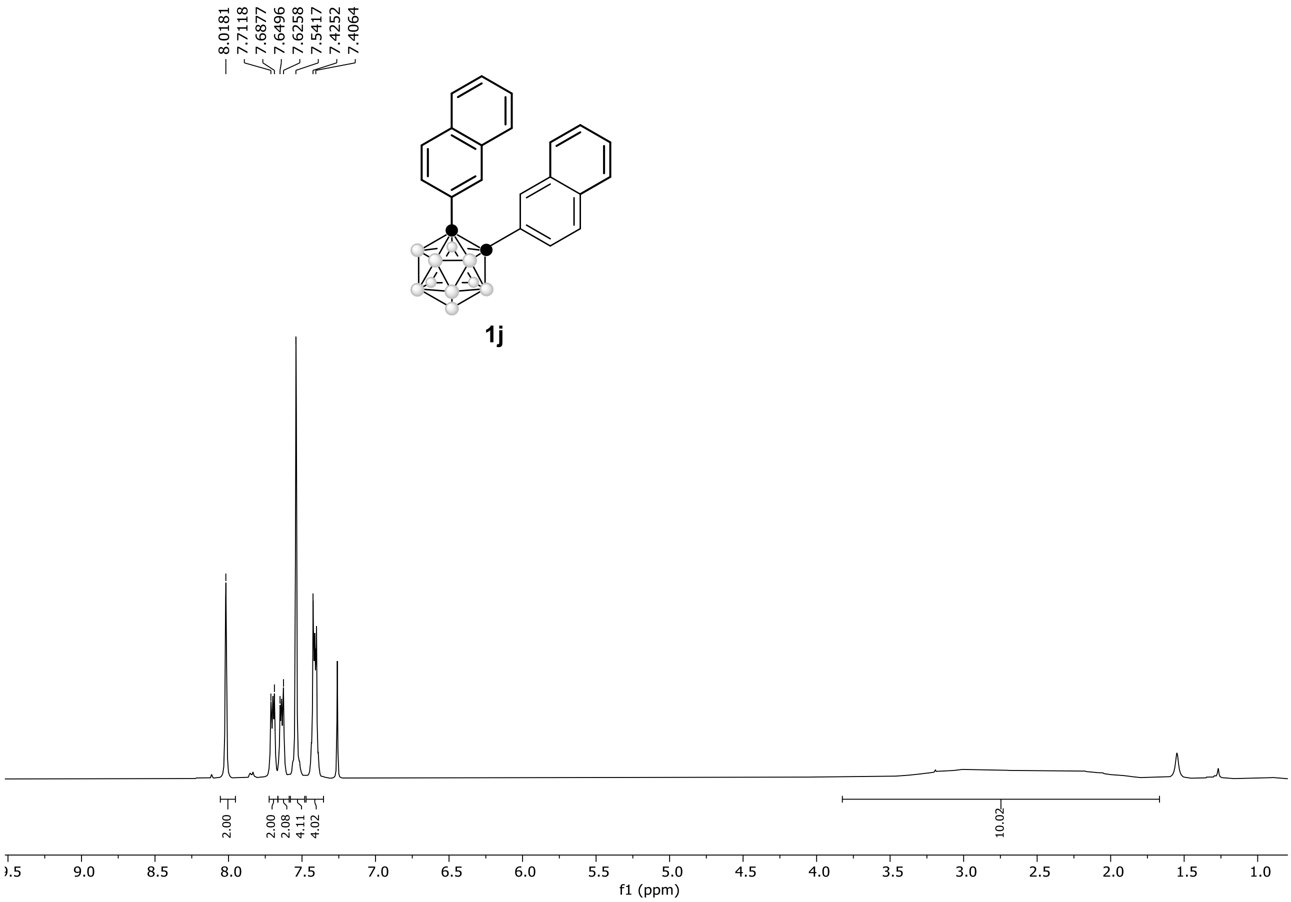

1j

^{1}H-NMR (400 MHz, $CDCl_3$)

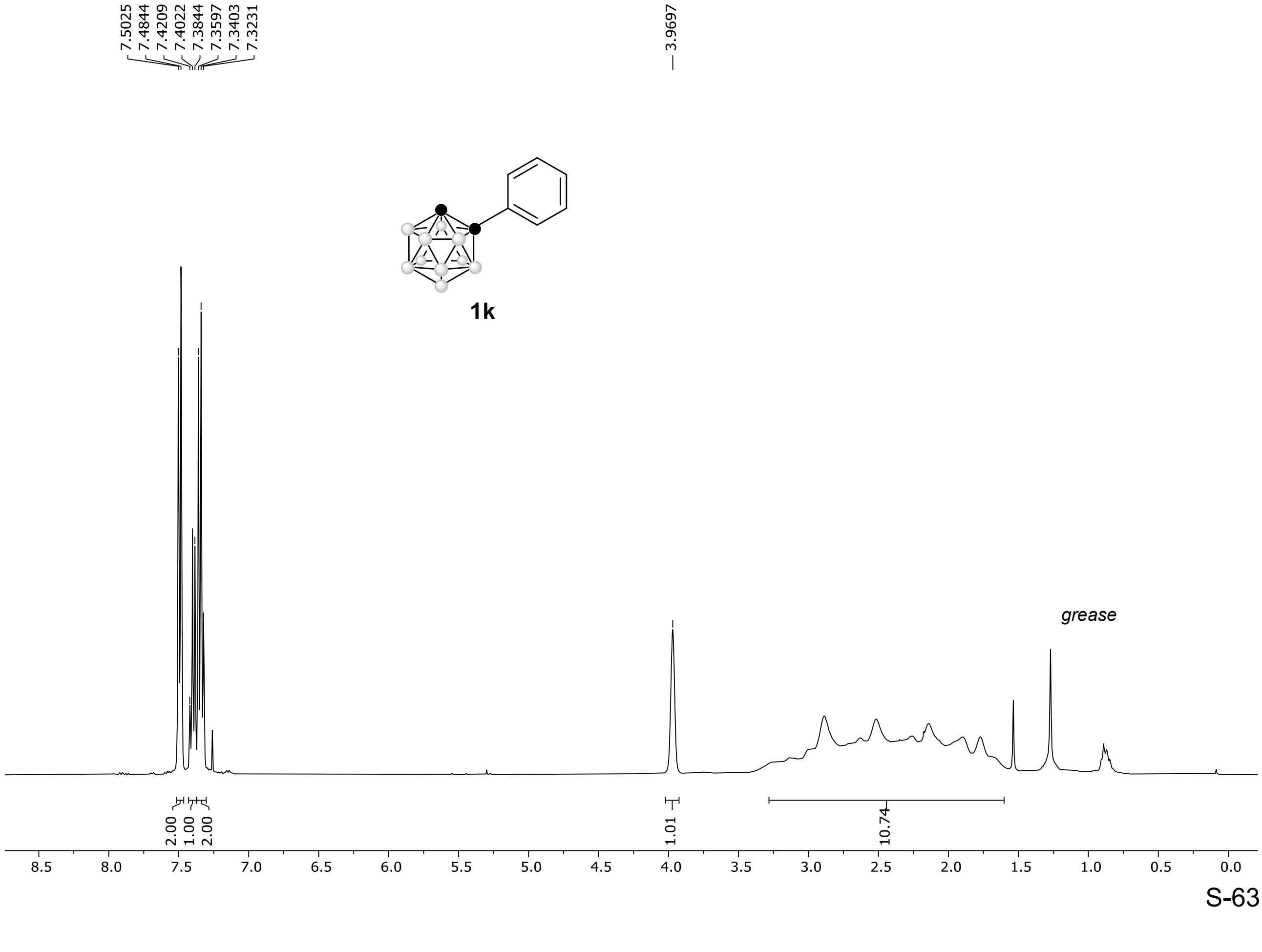

1k

¹H-NMR (400 MHz, $CDCl_3$)

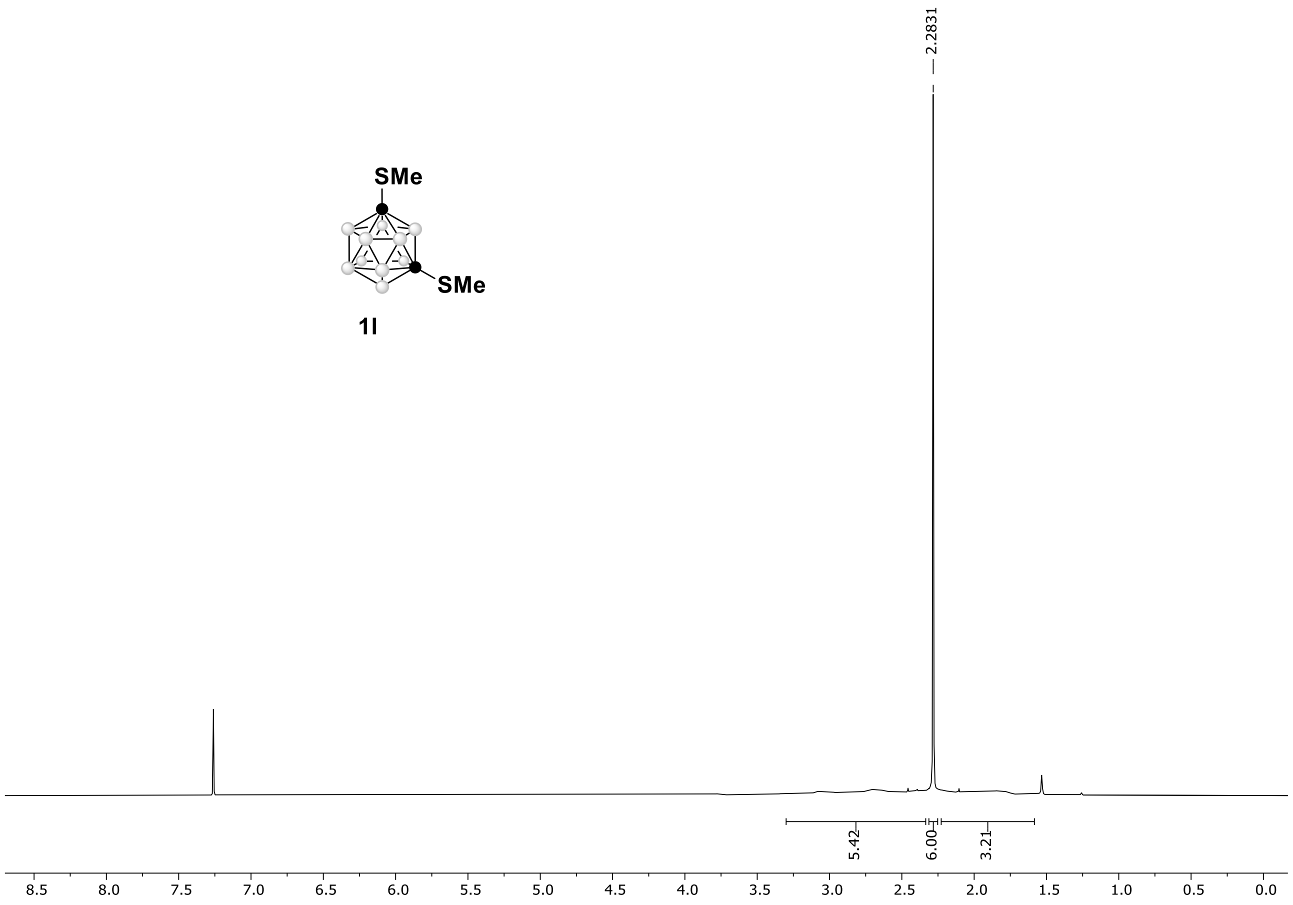

¹H-NMR (400 MHz, $CDCl_3$)

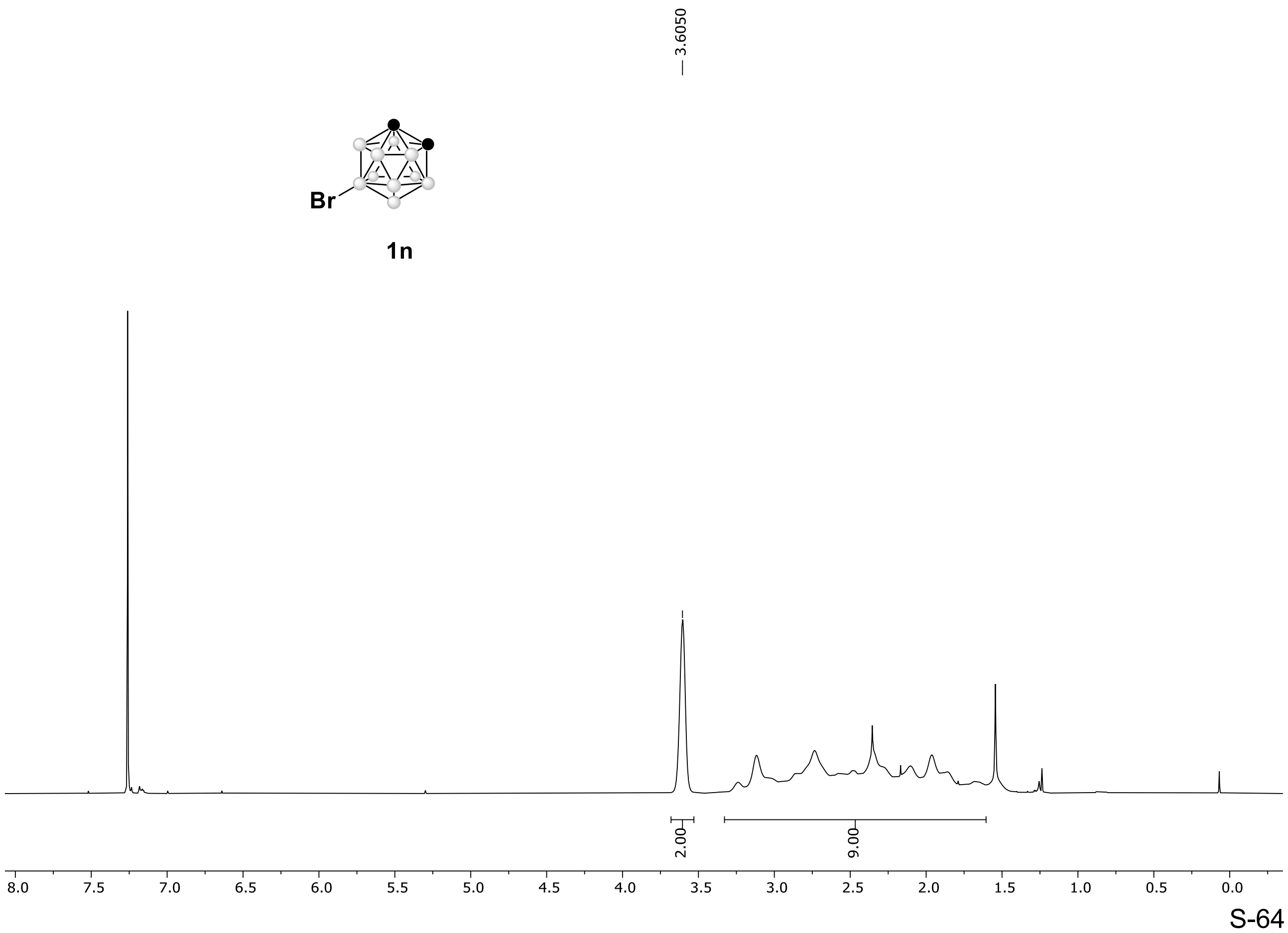

^{1}H-NMR (400 MHz, $CDCl_3$)

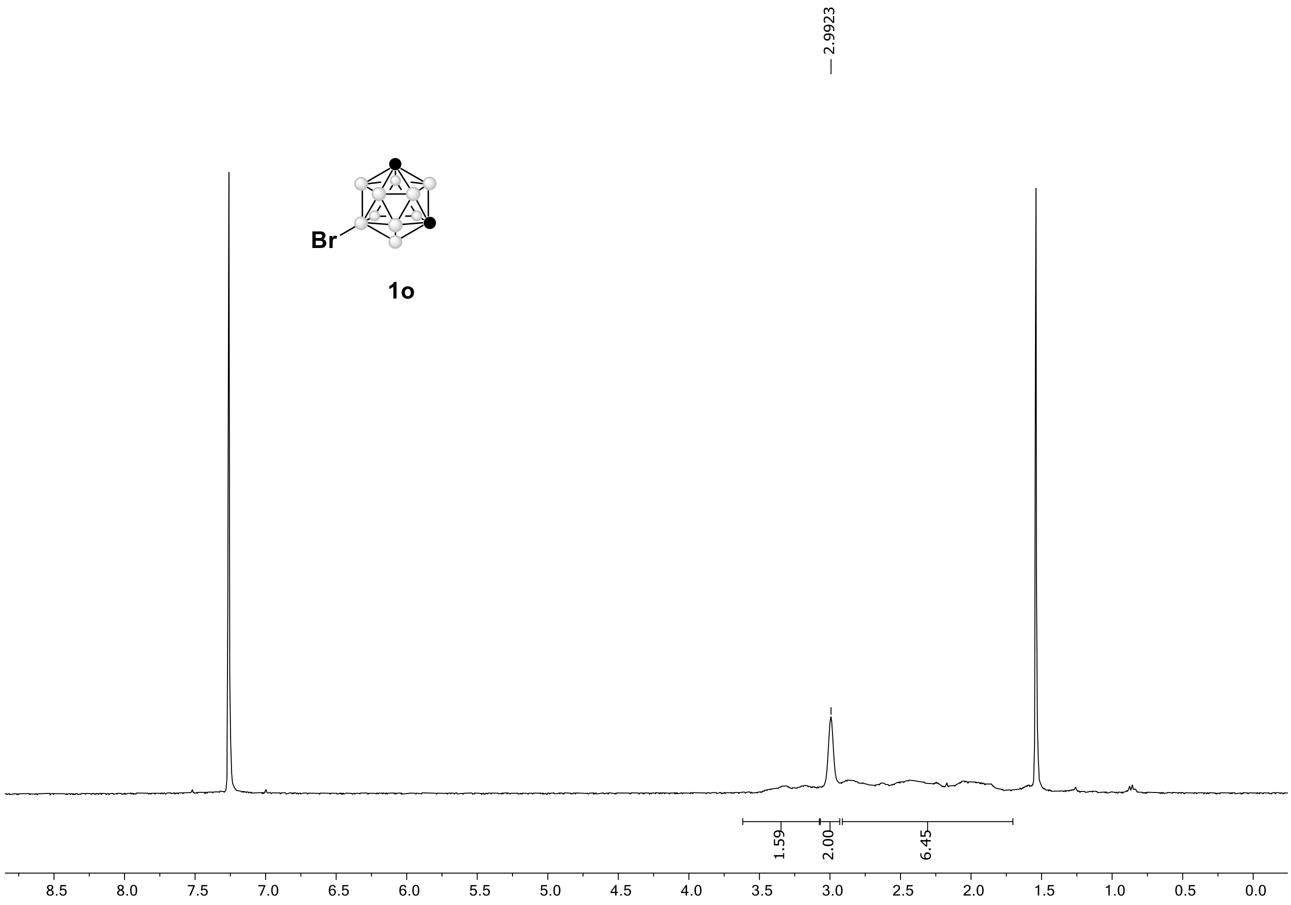

^{1}H-NMR (400 MHz, $CDCl_3$)

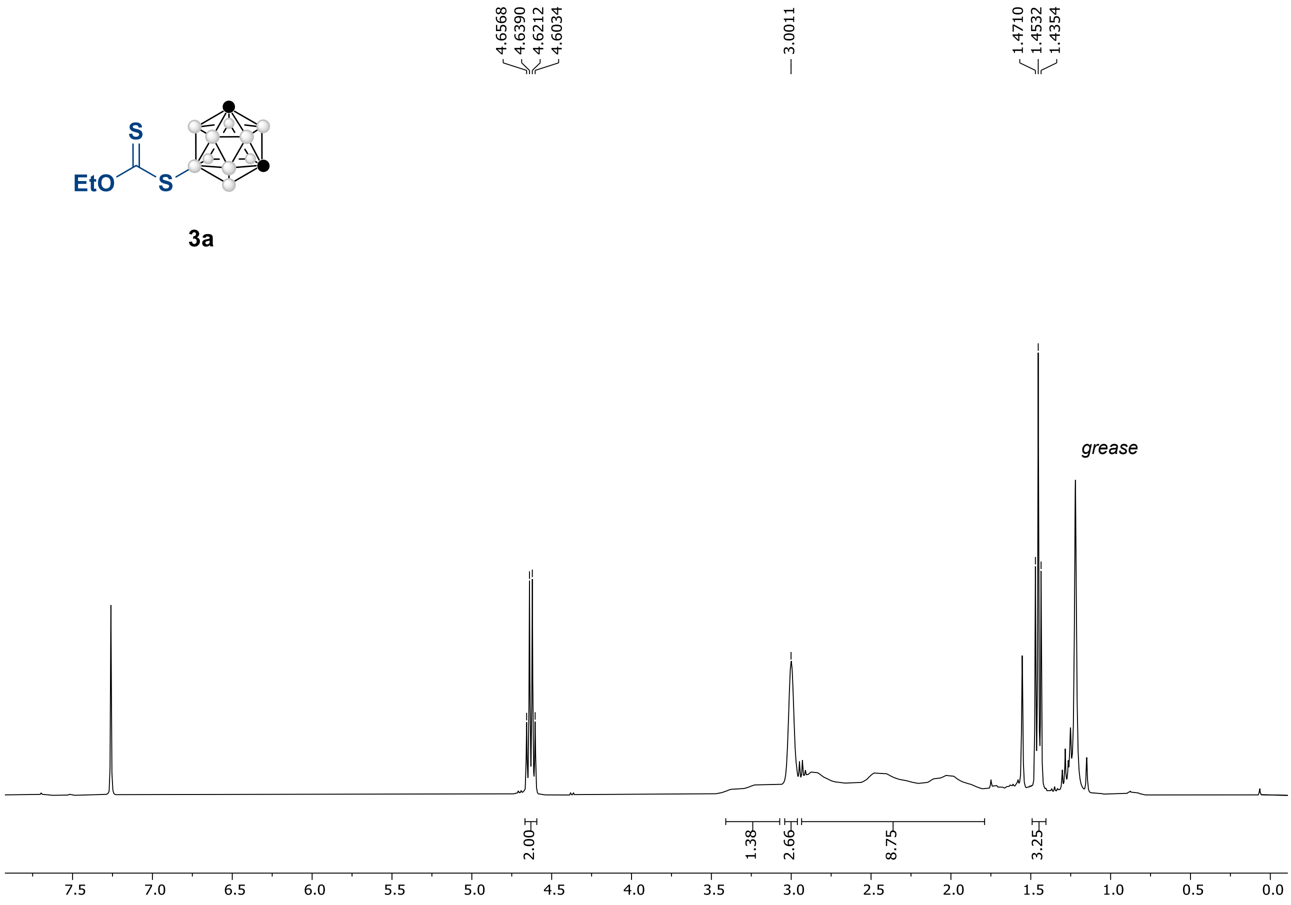

^{13}C{^{1}H}-NMR (100 MHz, $CDCl_3$)

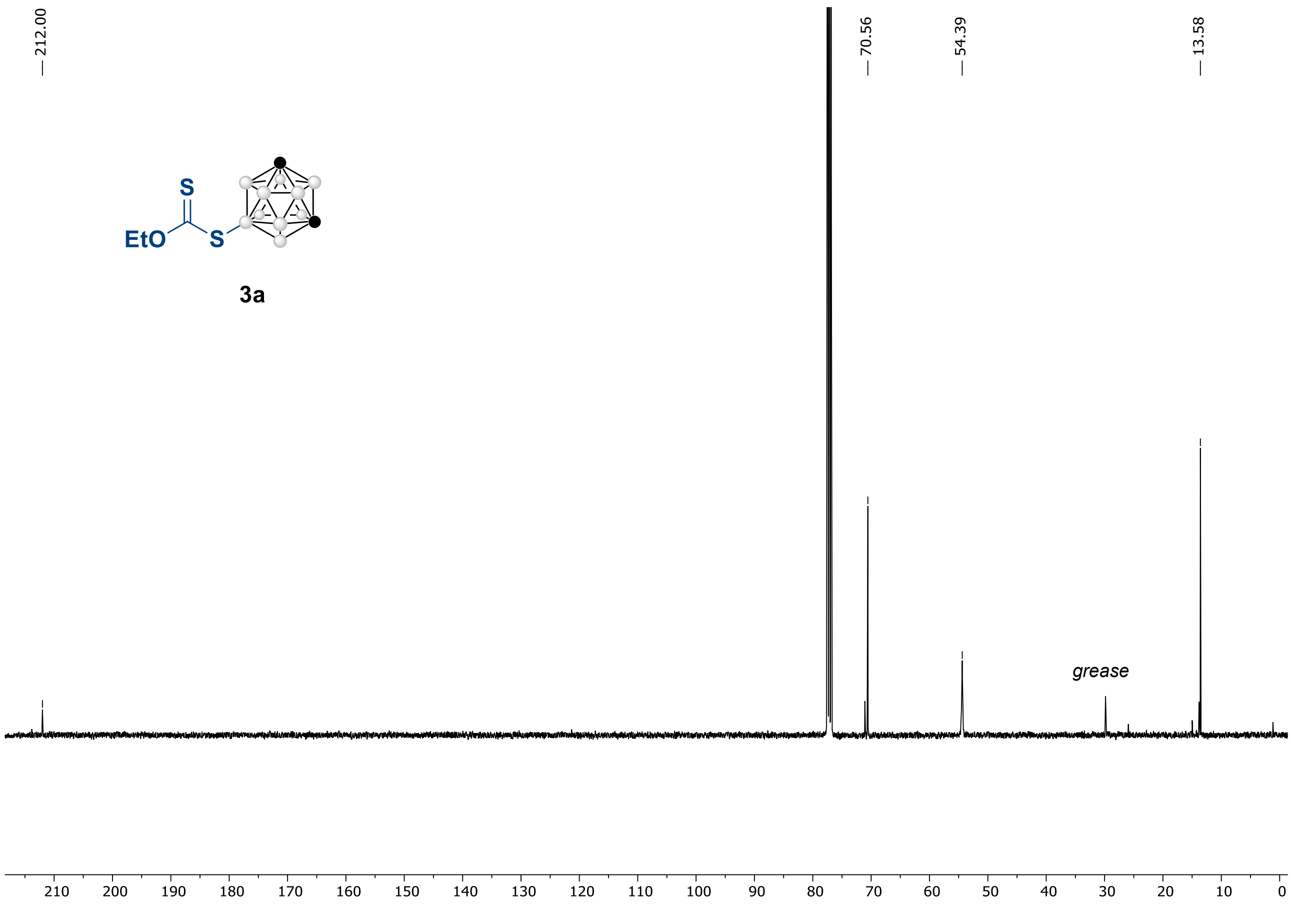

DEPT135 (100 MHz, $CDCl_3$)

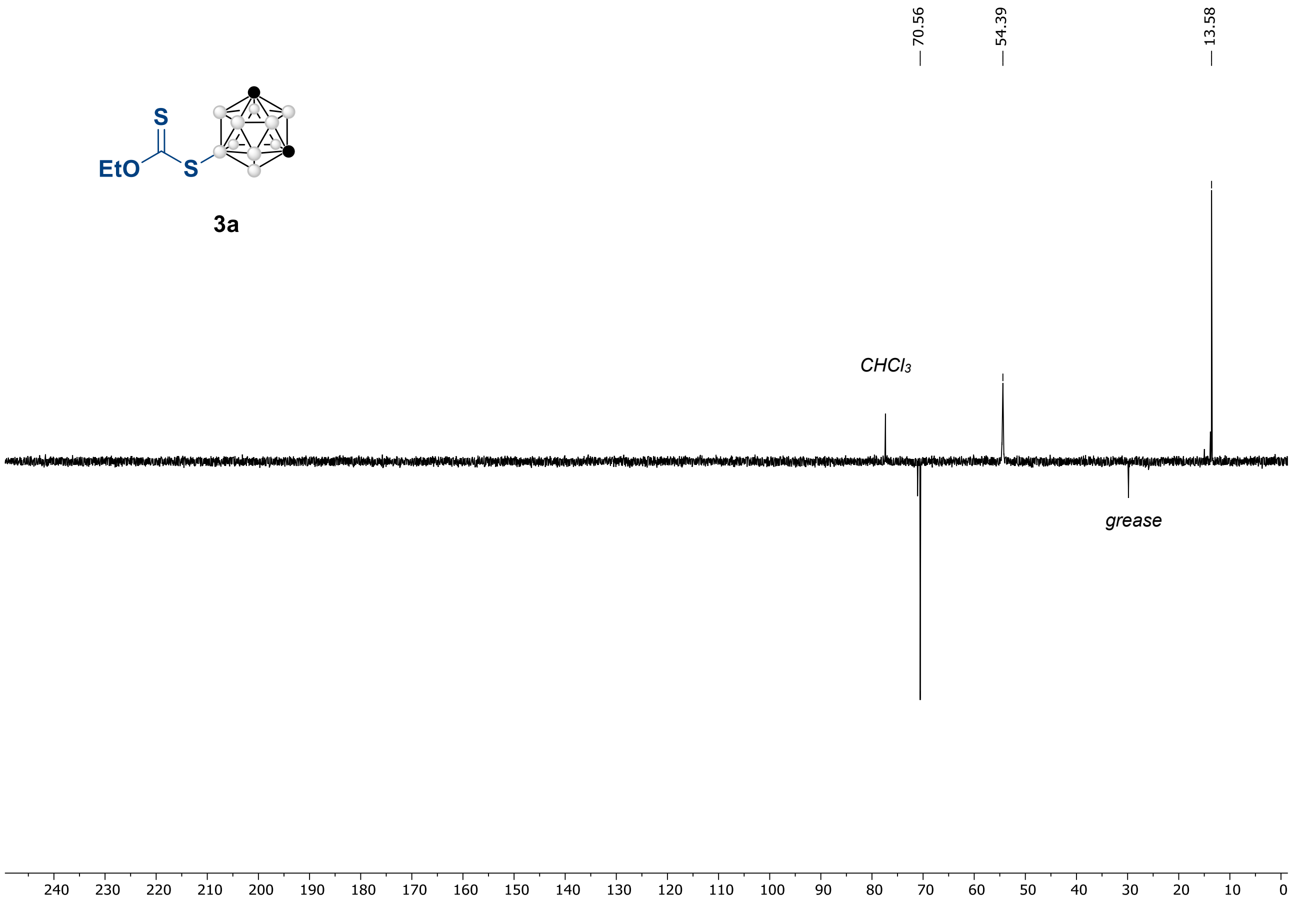

$^{11}B\{^{1}H\}$-NMR (128 MHz, $CDCl_3$)

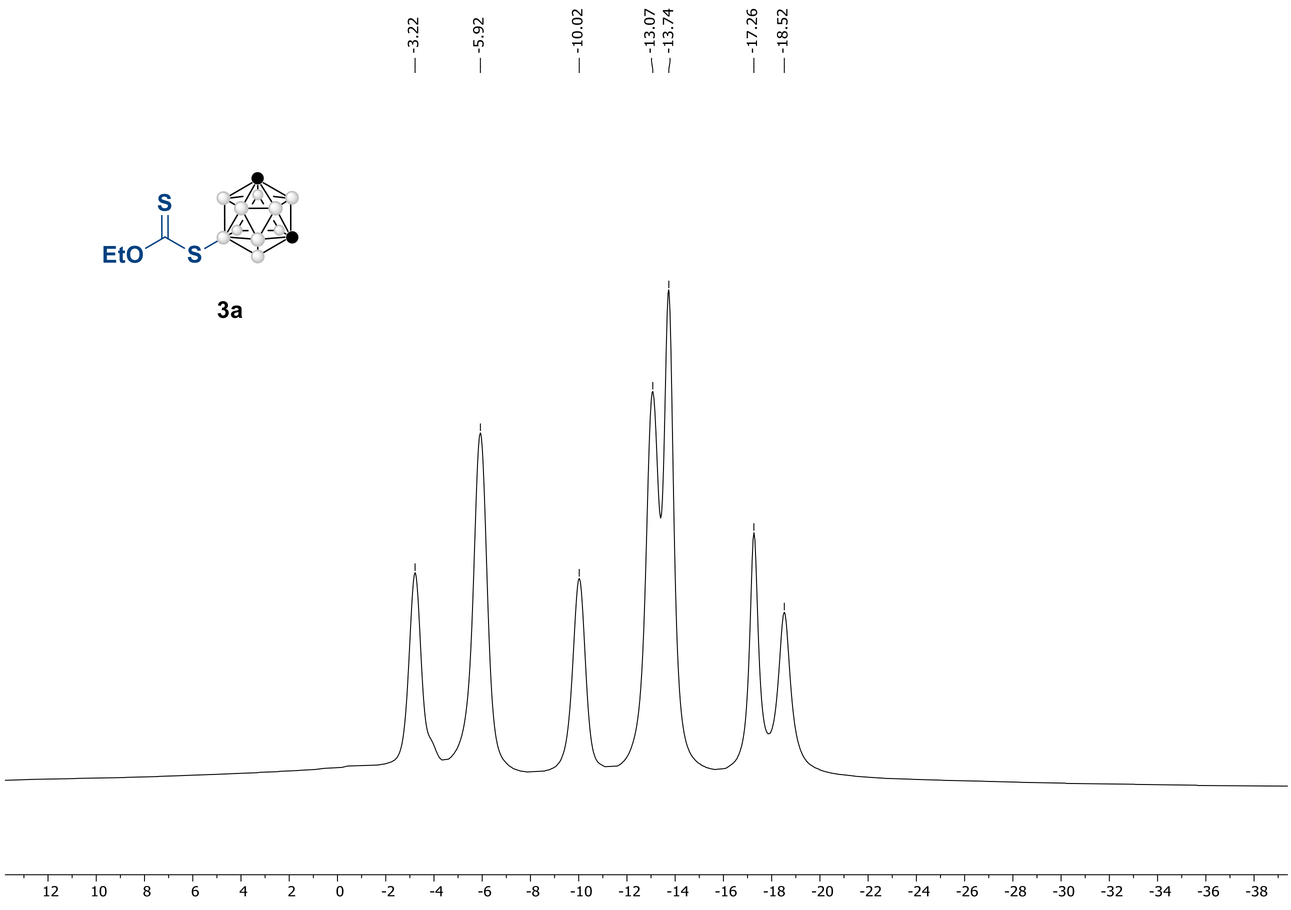

2D – COSY (100 MHz, $CDCl_3$)

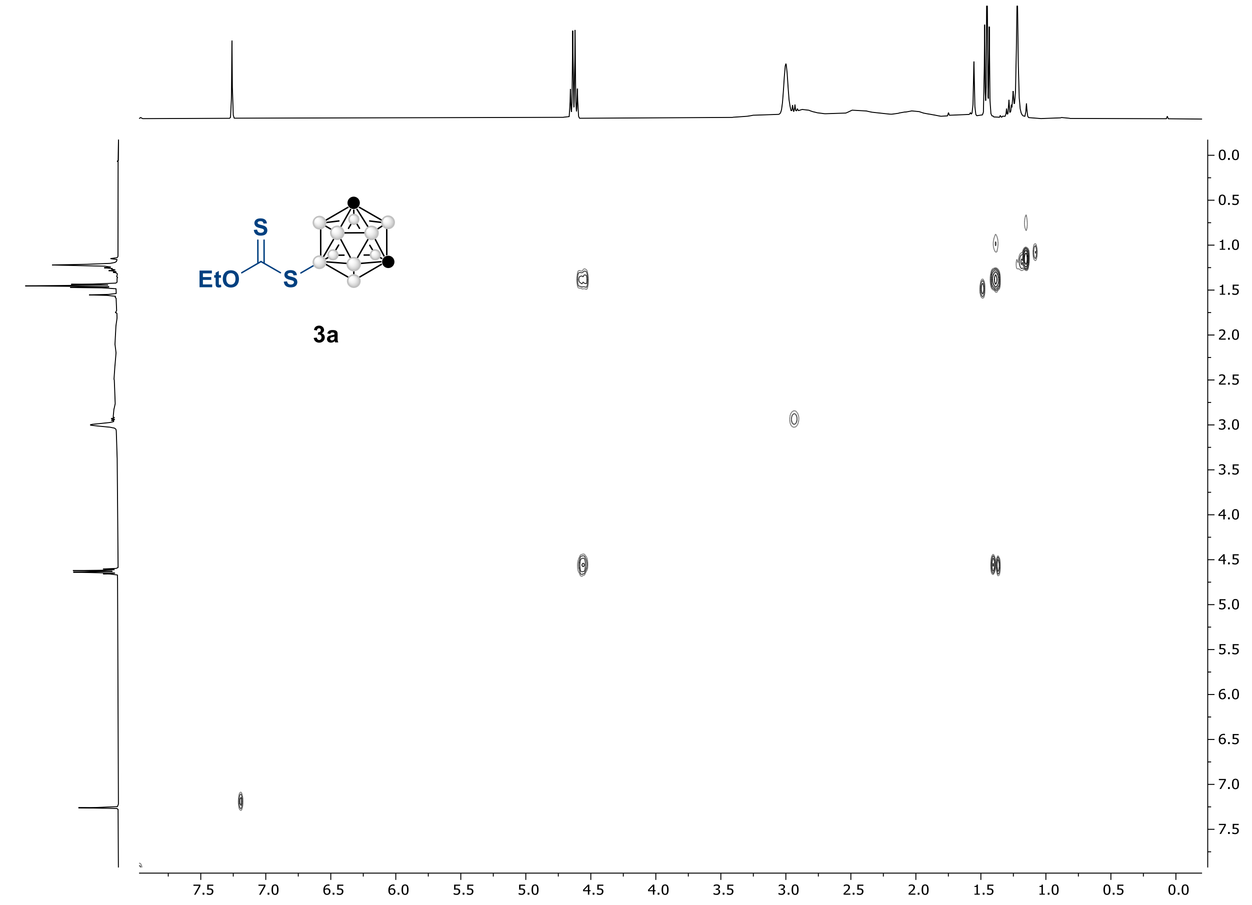

^{1}H-NMR (400 MHz, $CDCl_3$)

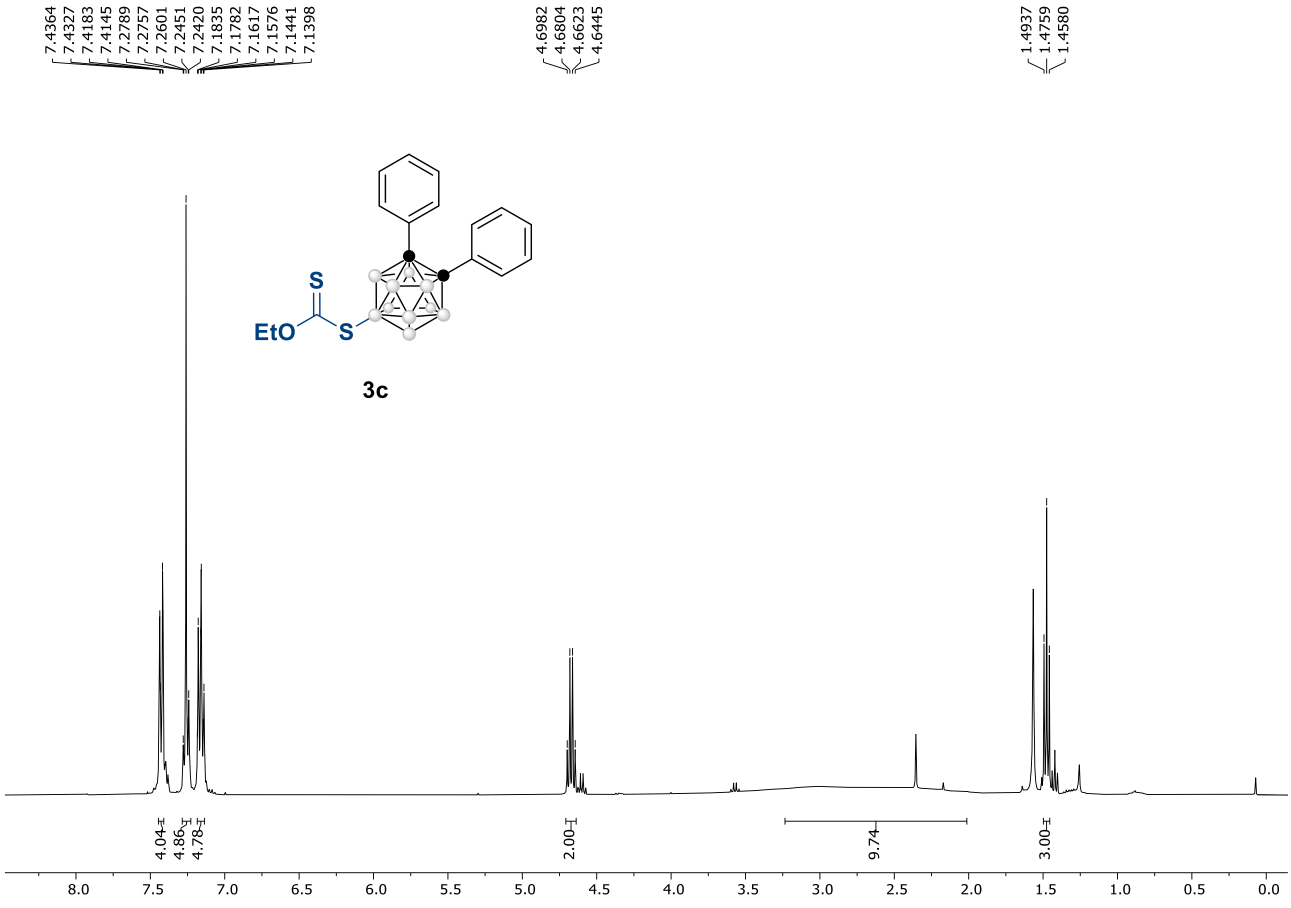

$^{13}C\{^{1}H\}$-NMR (100 MHz, $CDCl_3$)

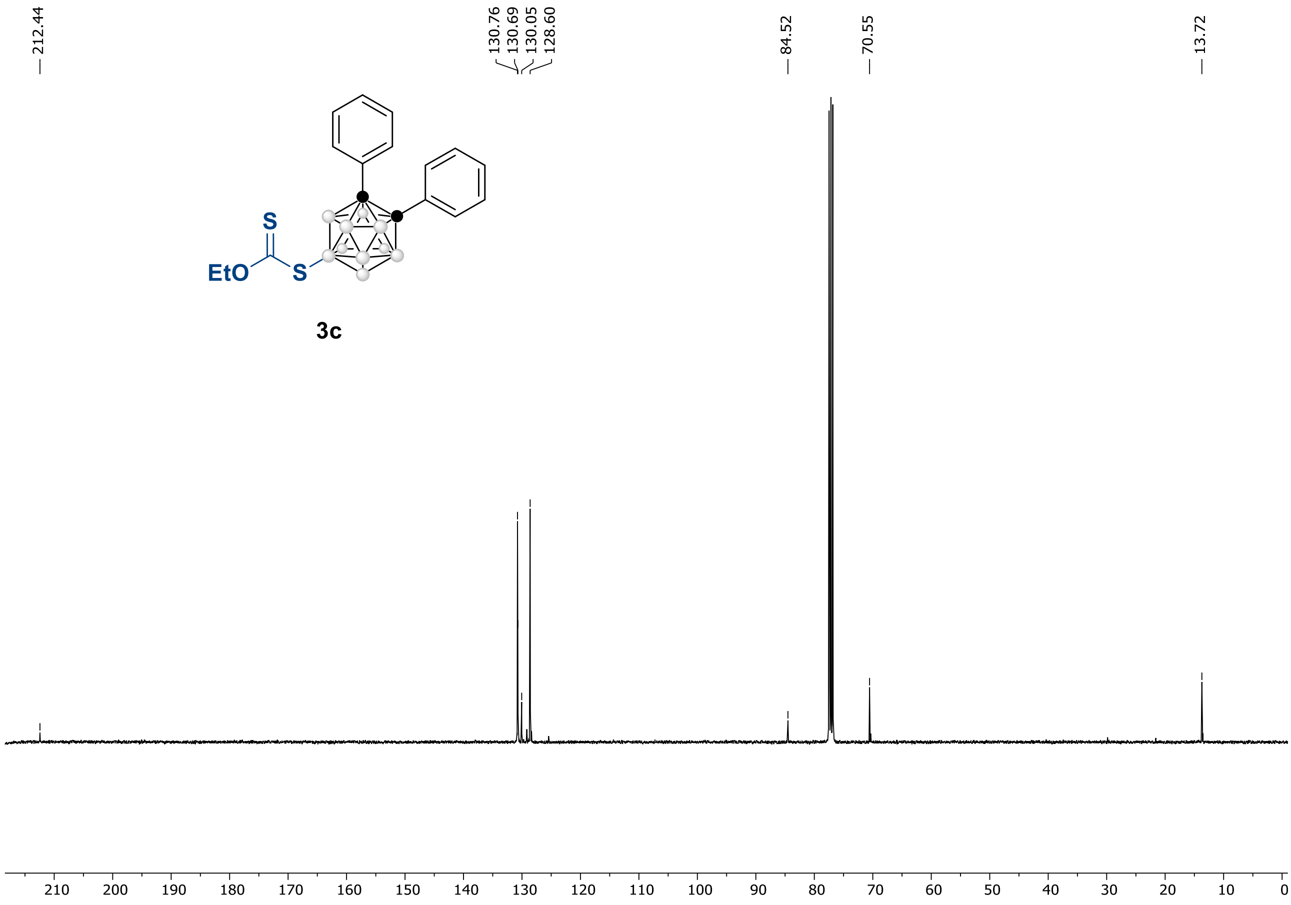

DEPT135 (100 MHz, $CDCl_3$)

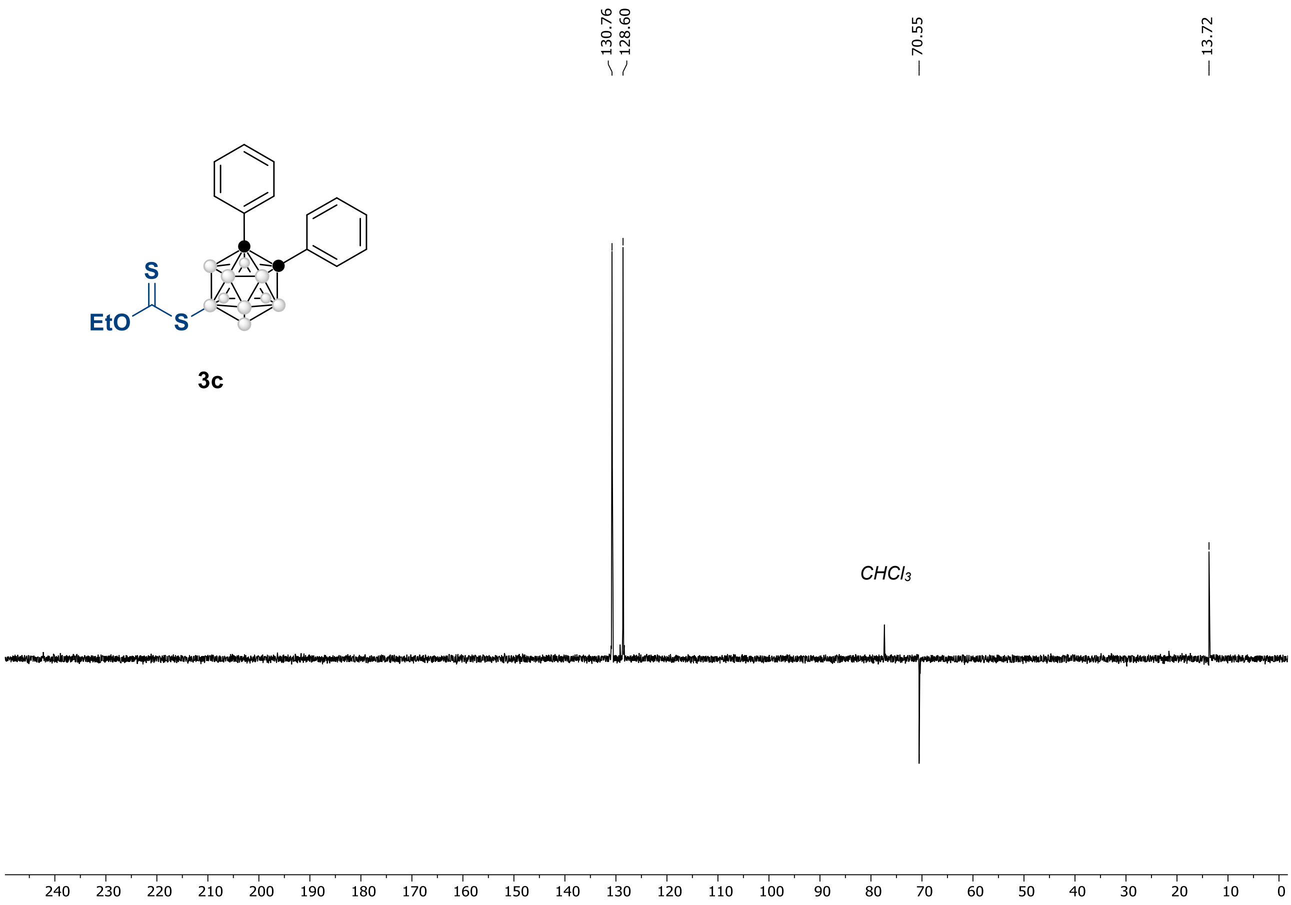

^{11}B{^{1}H}-NMR (128 MHz, $CDCl_3$)

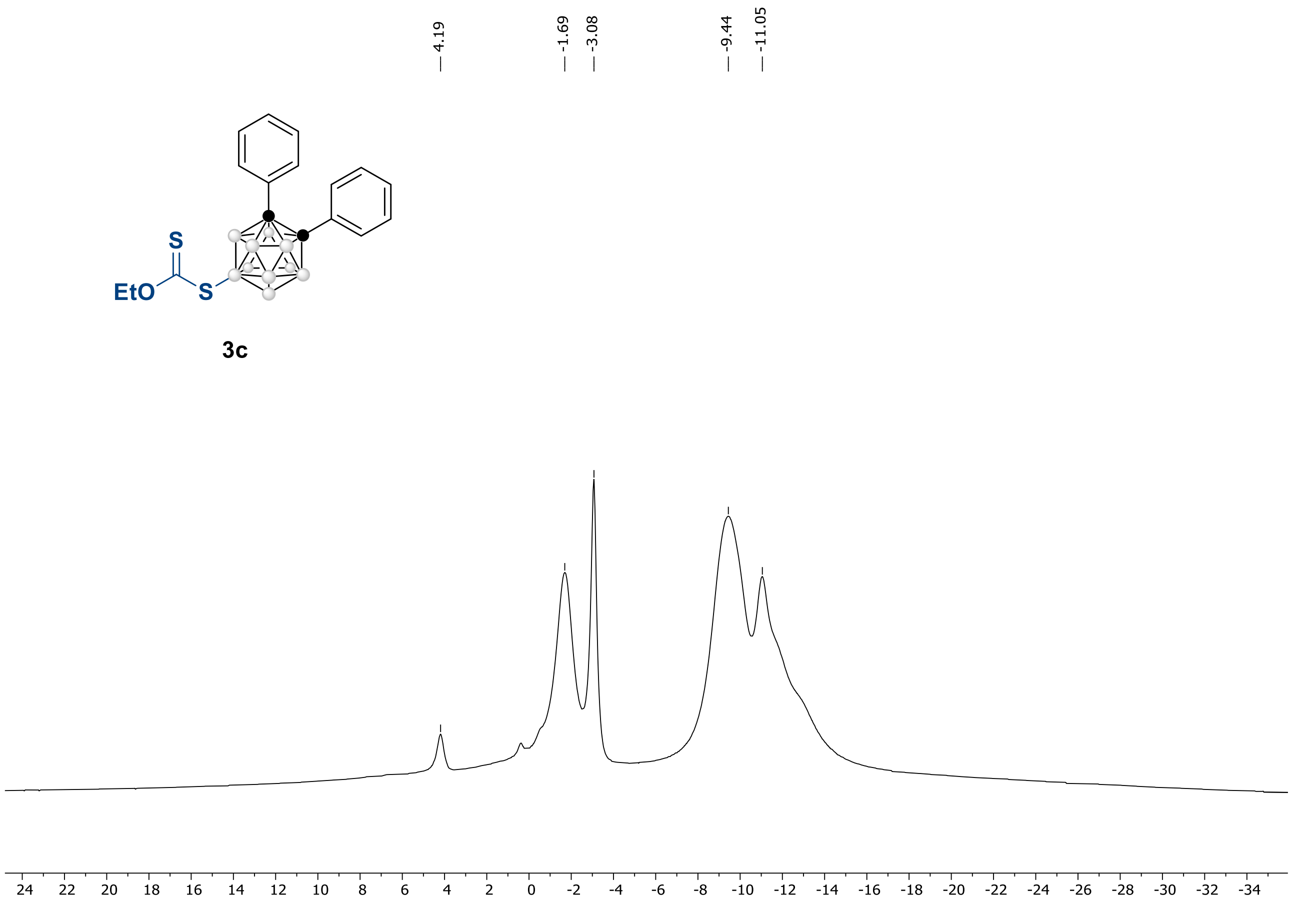

2D – COSY (100 MHz, $CDCl_3$)

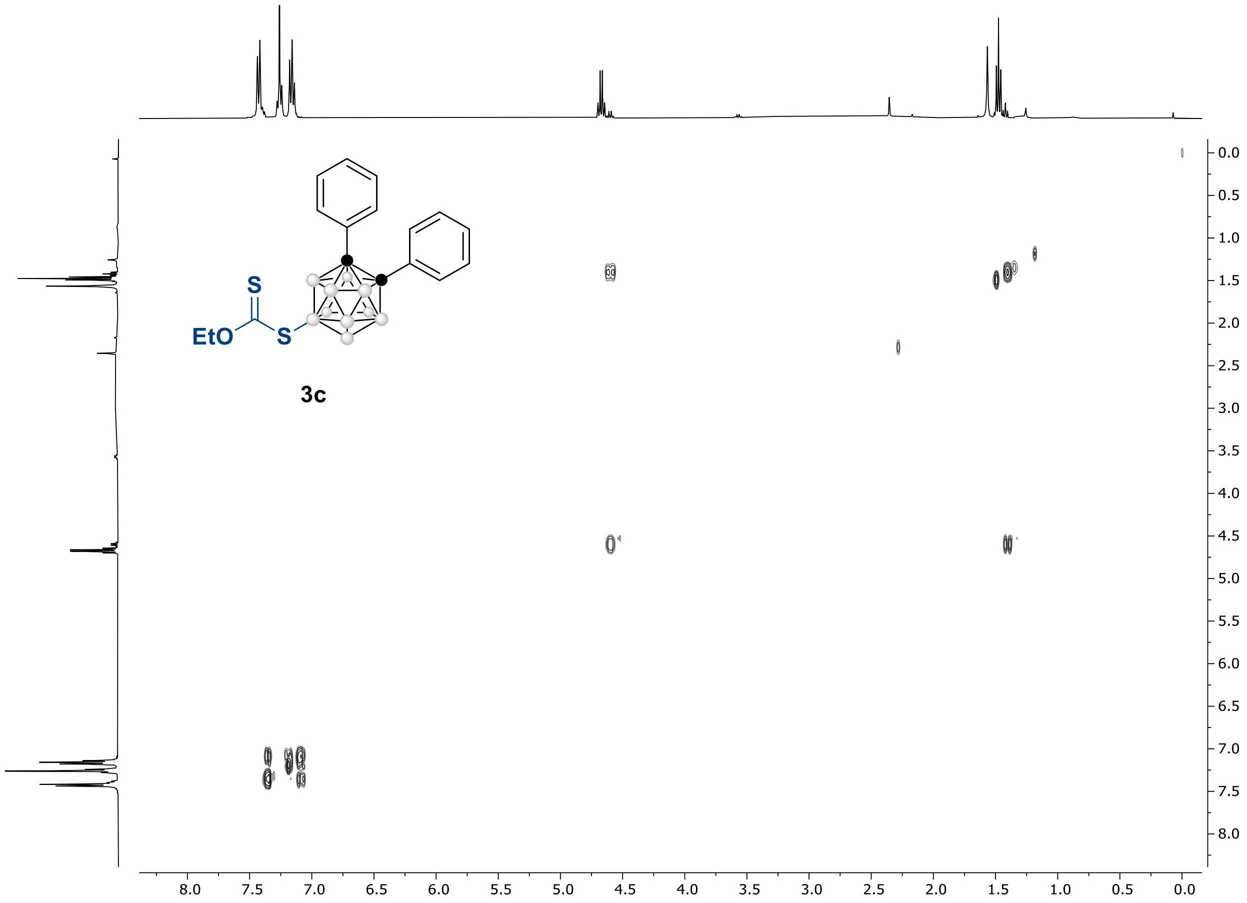

^{1}H-NMR (400 MHz, $CDCl_3$)

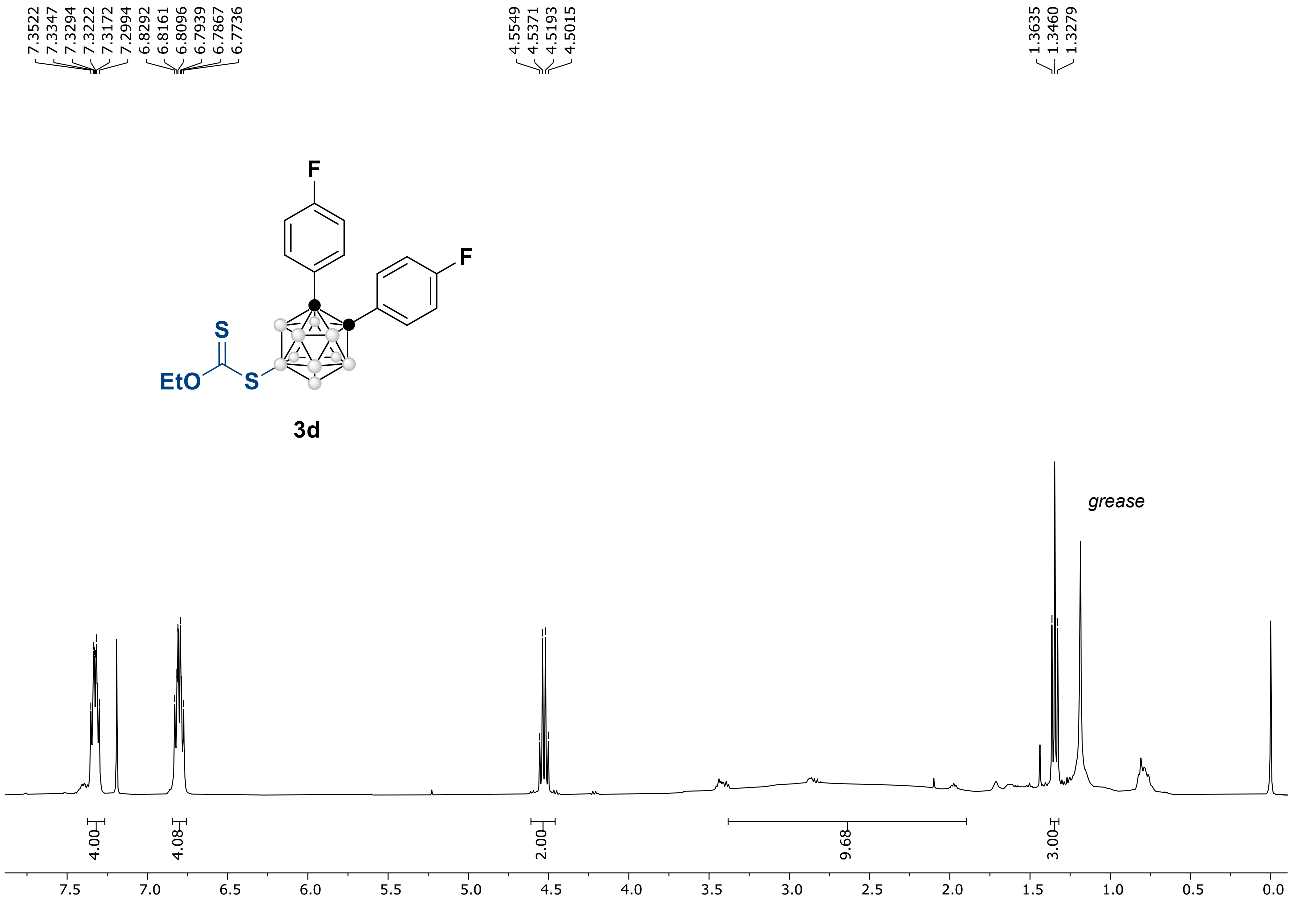

^{13}C{^{1}H}-NMR (100 MHz, $CDCl_3$)

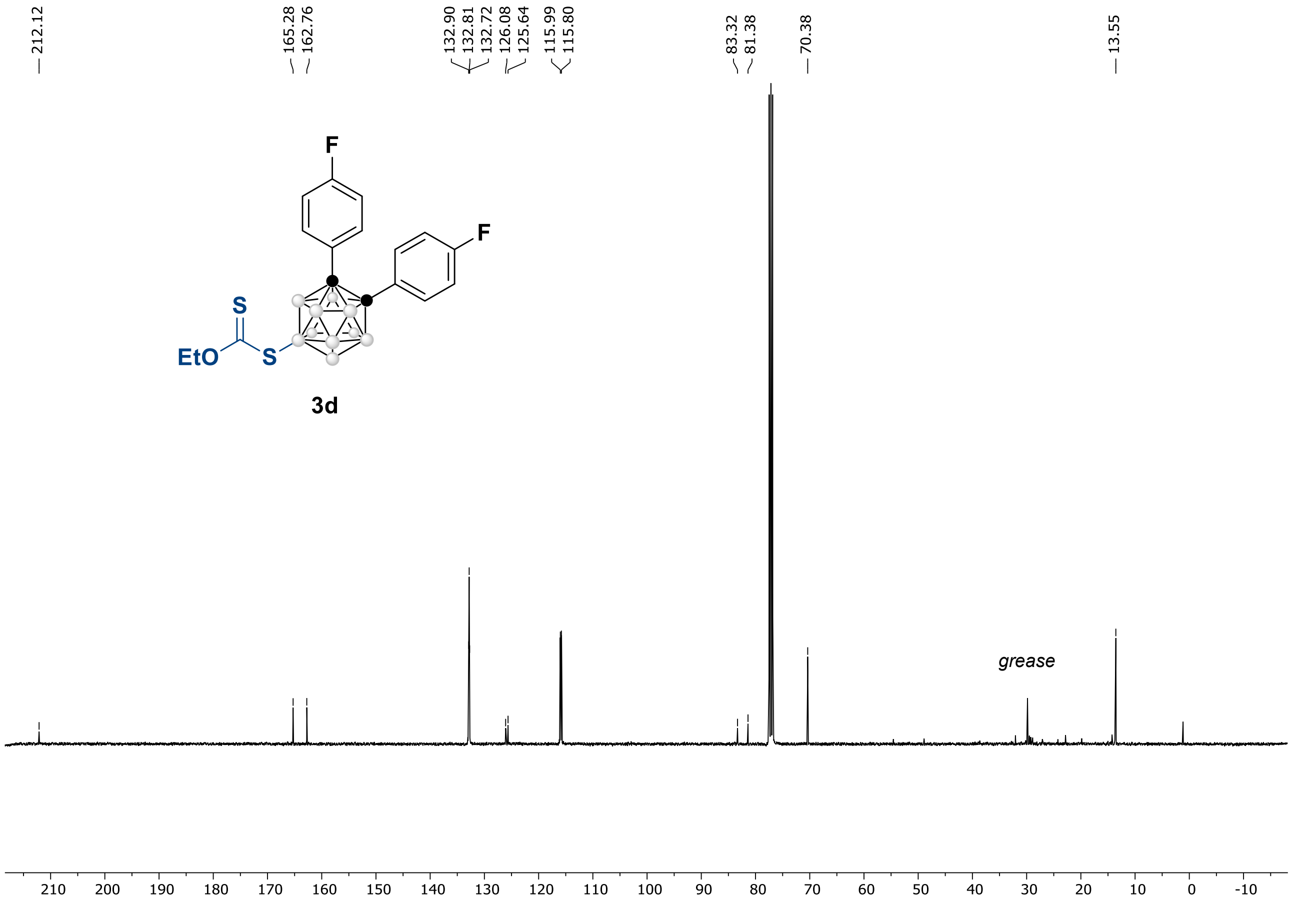

DEPT135 (100 MHz, $CDCl_3$)

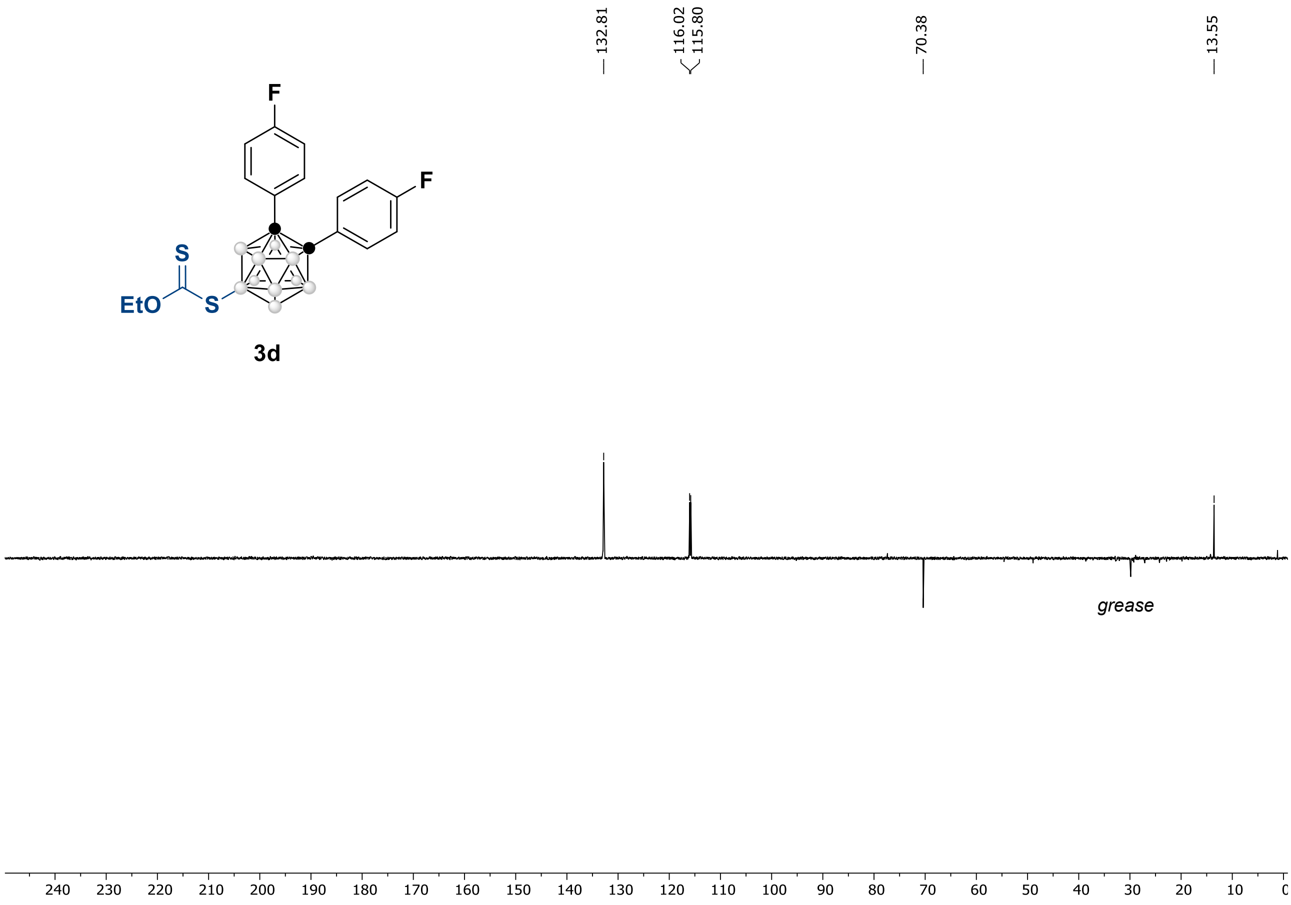

$^{11}B\{^1H\}$-NMR (128 MHz, $CDCl_3$)

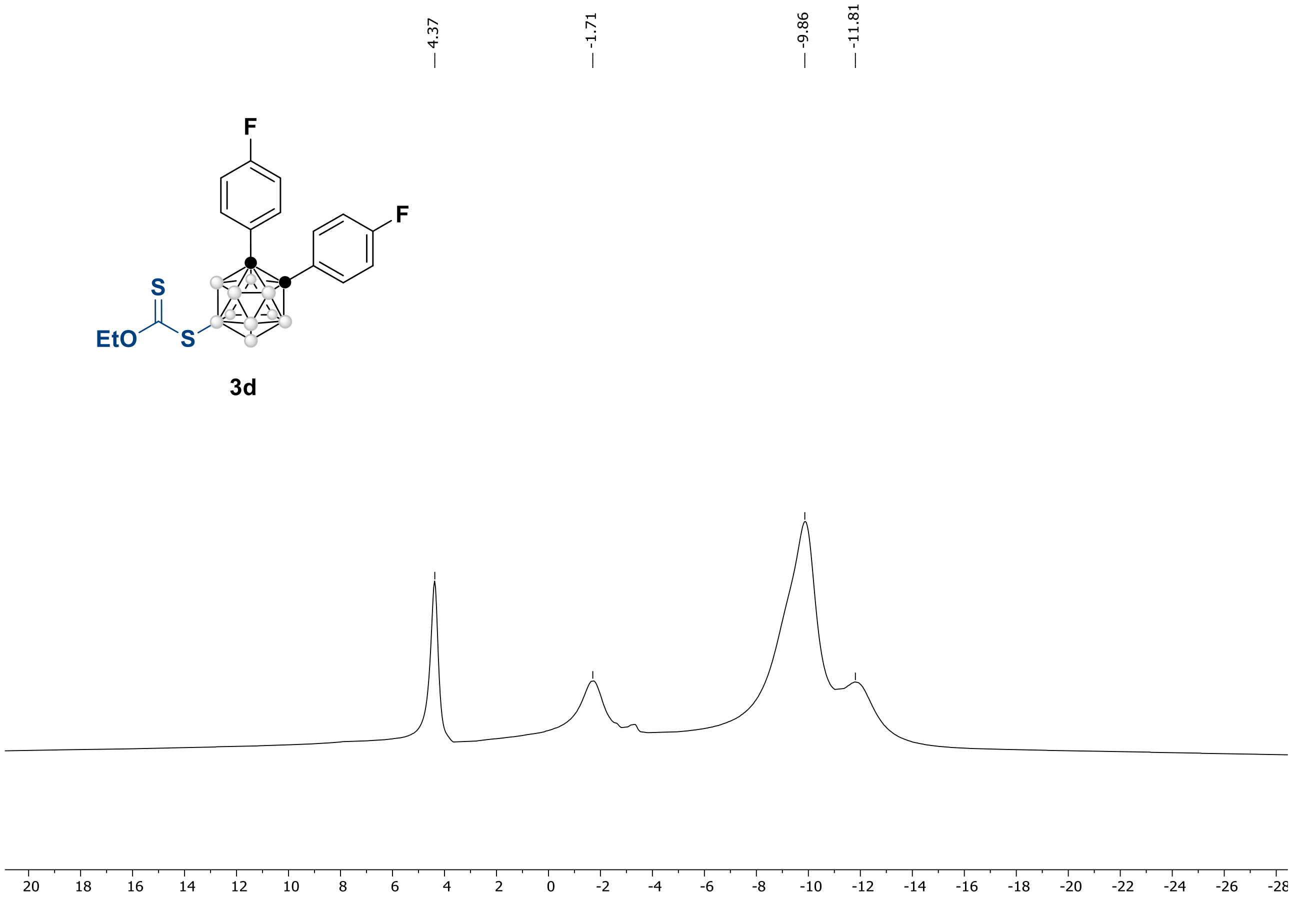

^{19}F{^{1}H}-NMR (376 MHz, CDCl$_3$)

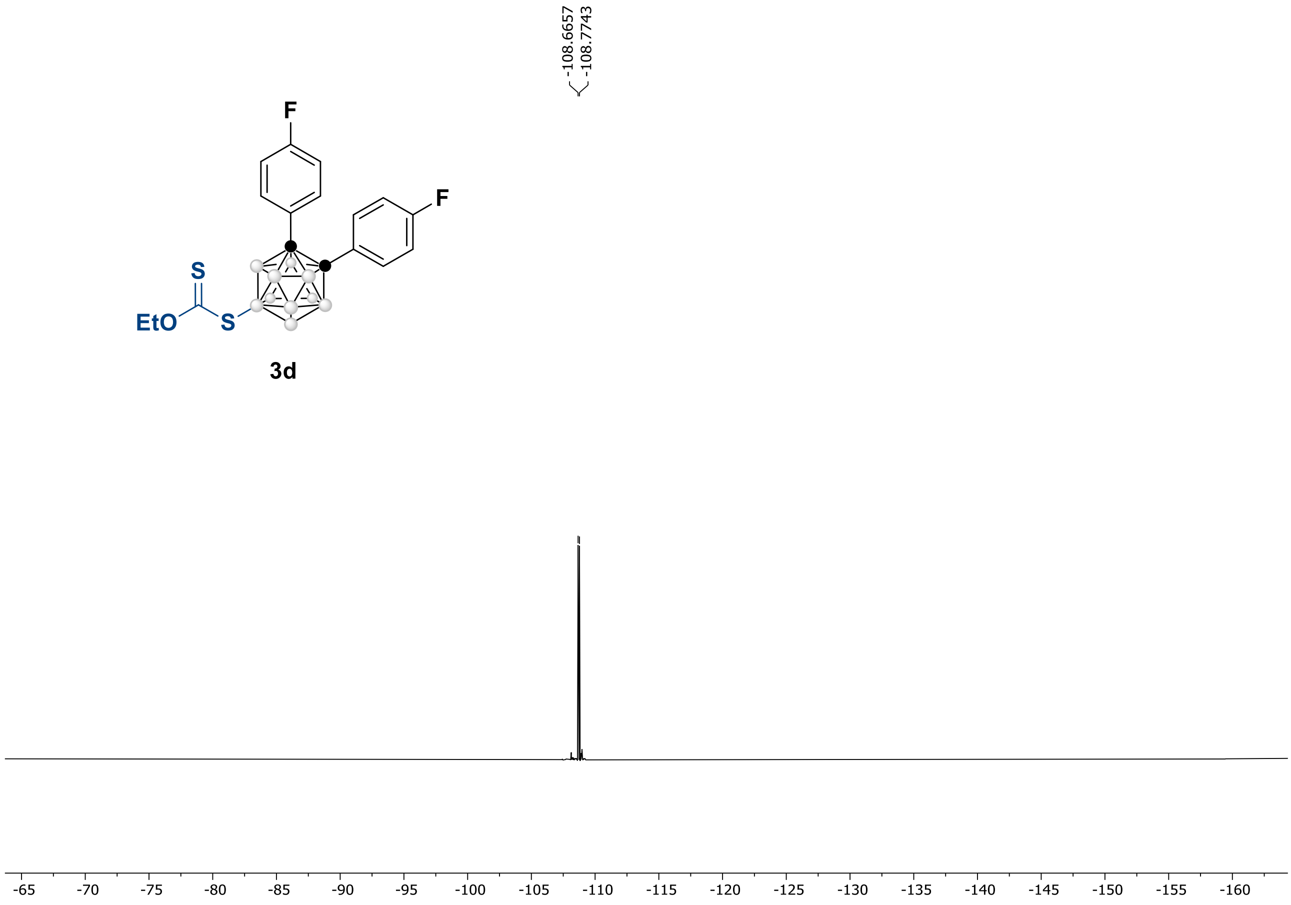

2D – COSY (400 MHz, CDCl$_3$)

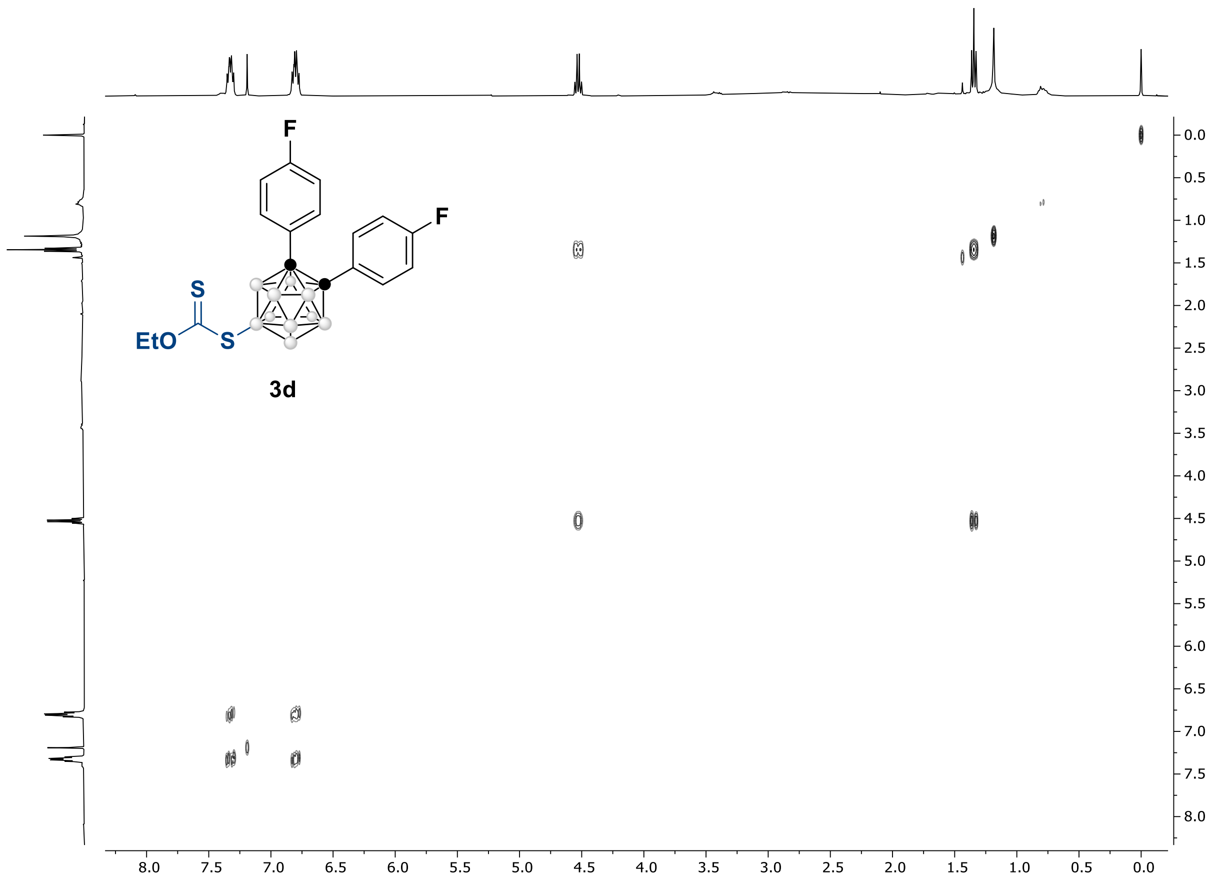

^{1}H-NMR (400 MHz, $CDCl_3$)

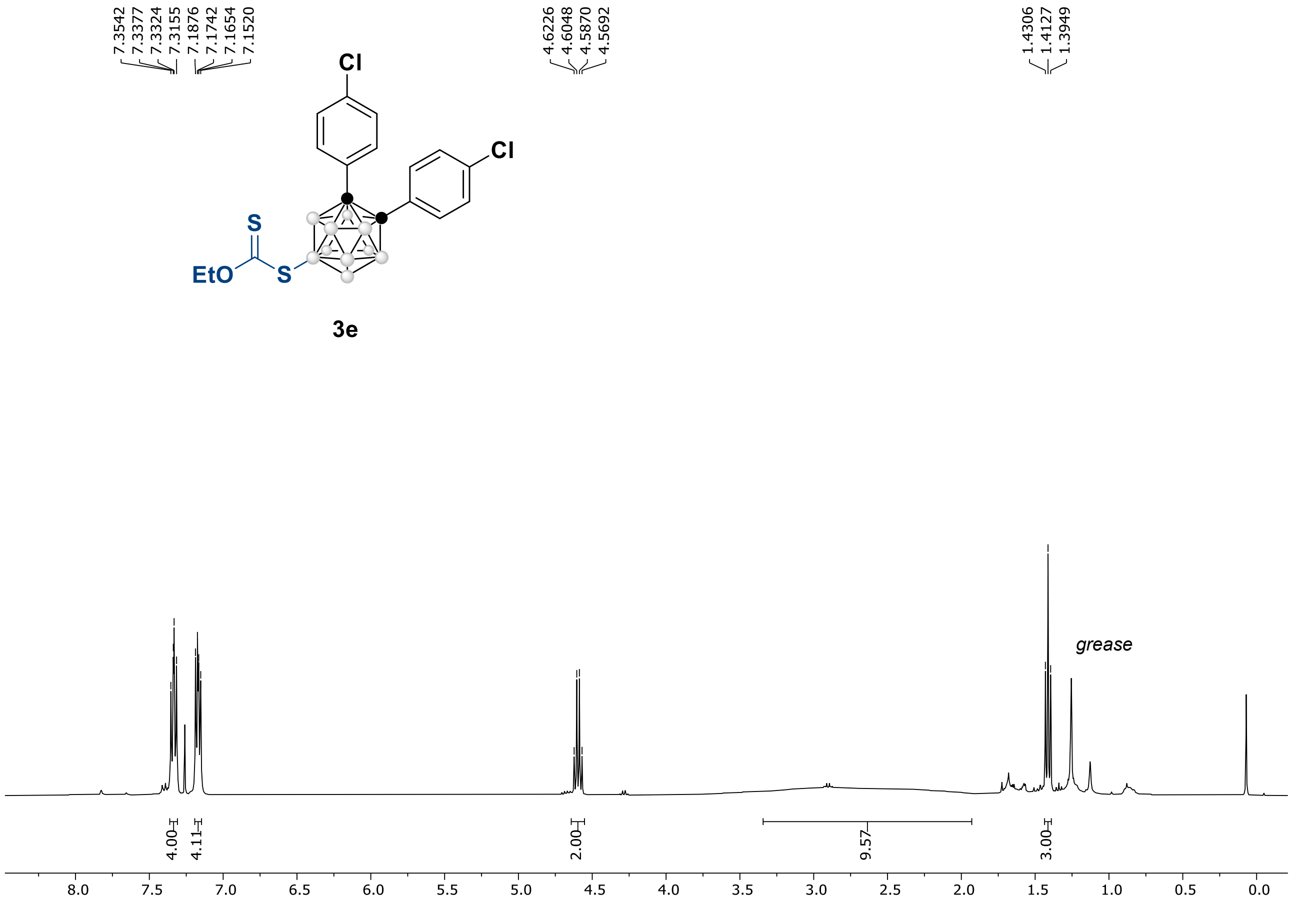

^{13}C{^{1}H}-NMR (100 MHz, $CDCl_3$)

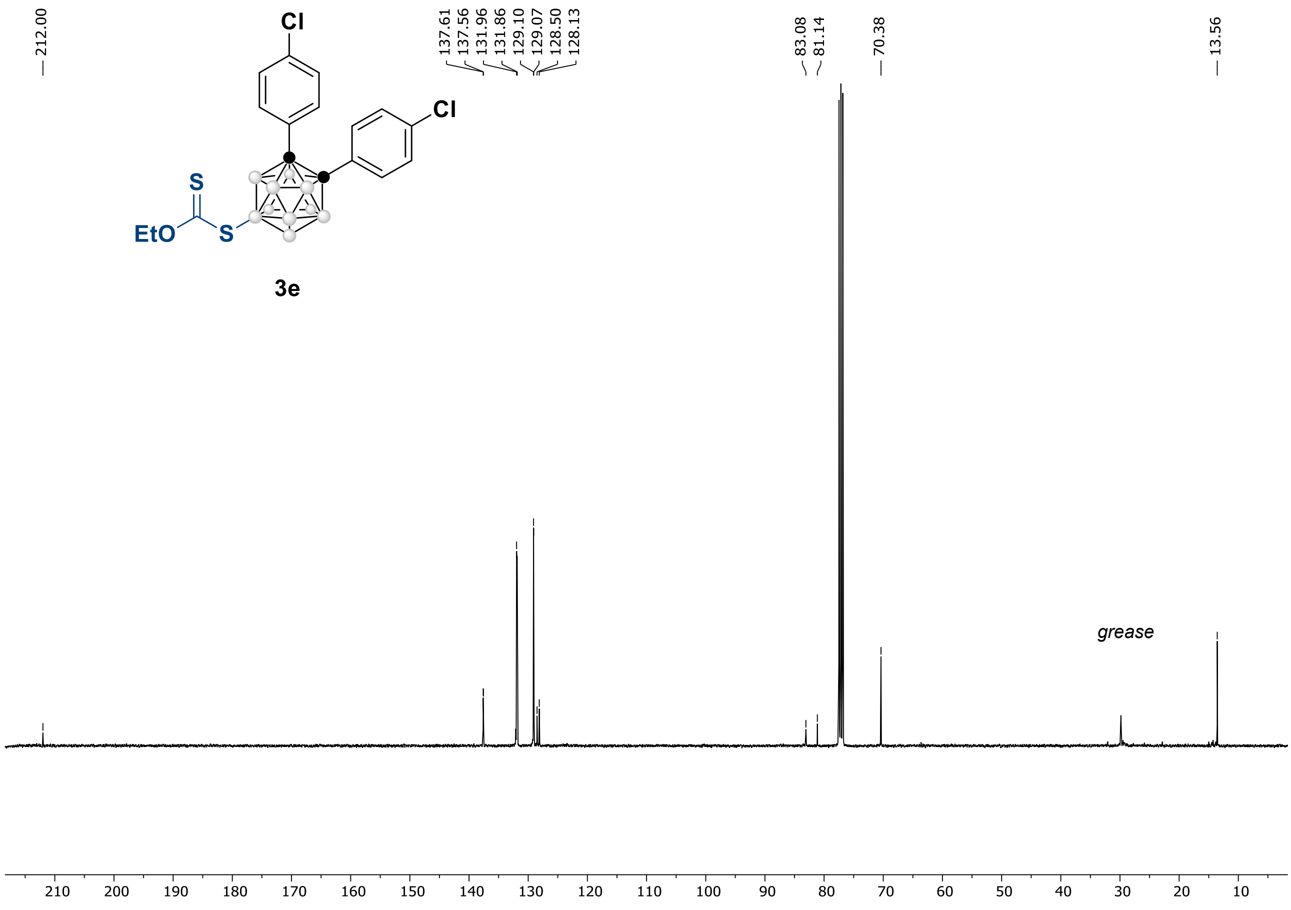

DEPT135 (100 MHz, $CDCl_3$)

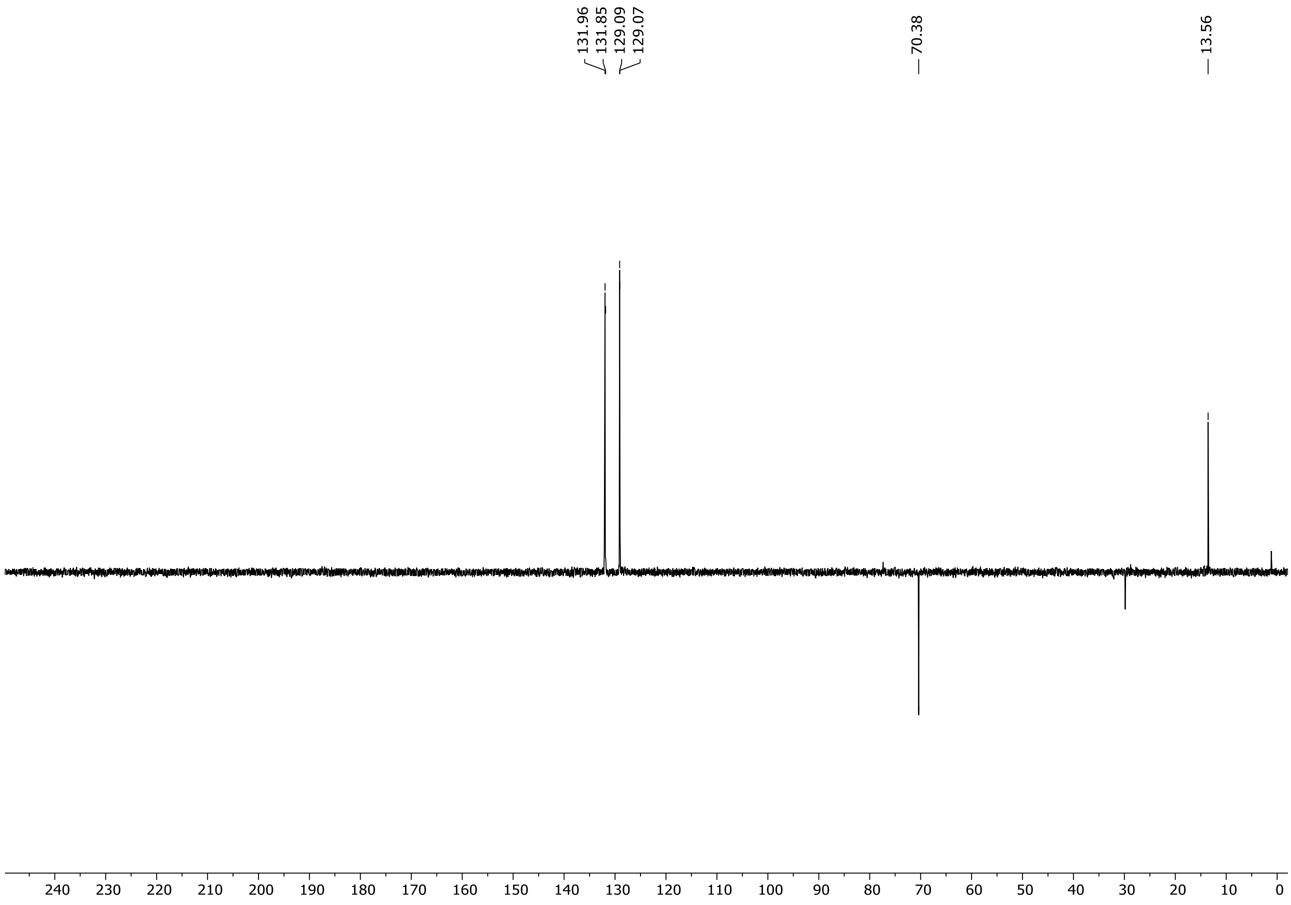

$^{11}B\{^1H\}$-NMR (128 MHz, $CDCl_3$)

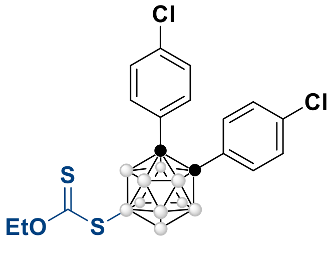

3e

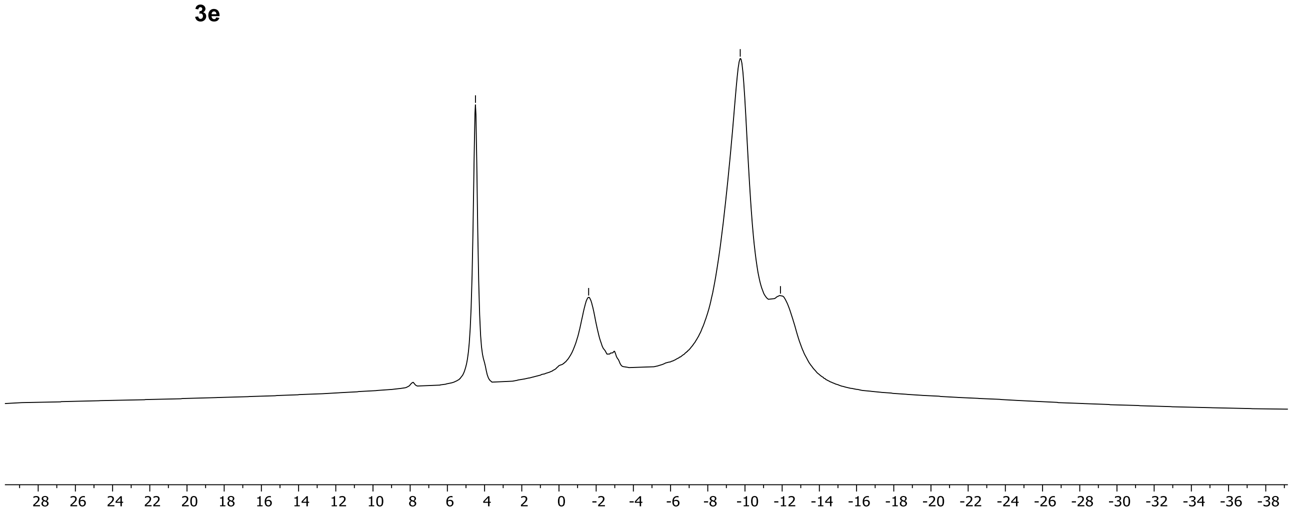

2D – COSY (400 MHz, $CDCl_3$)

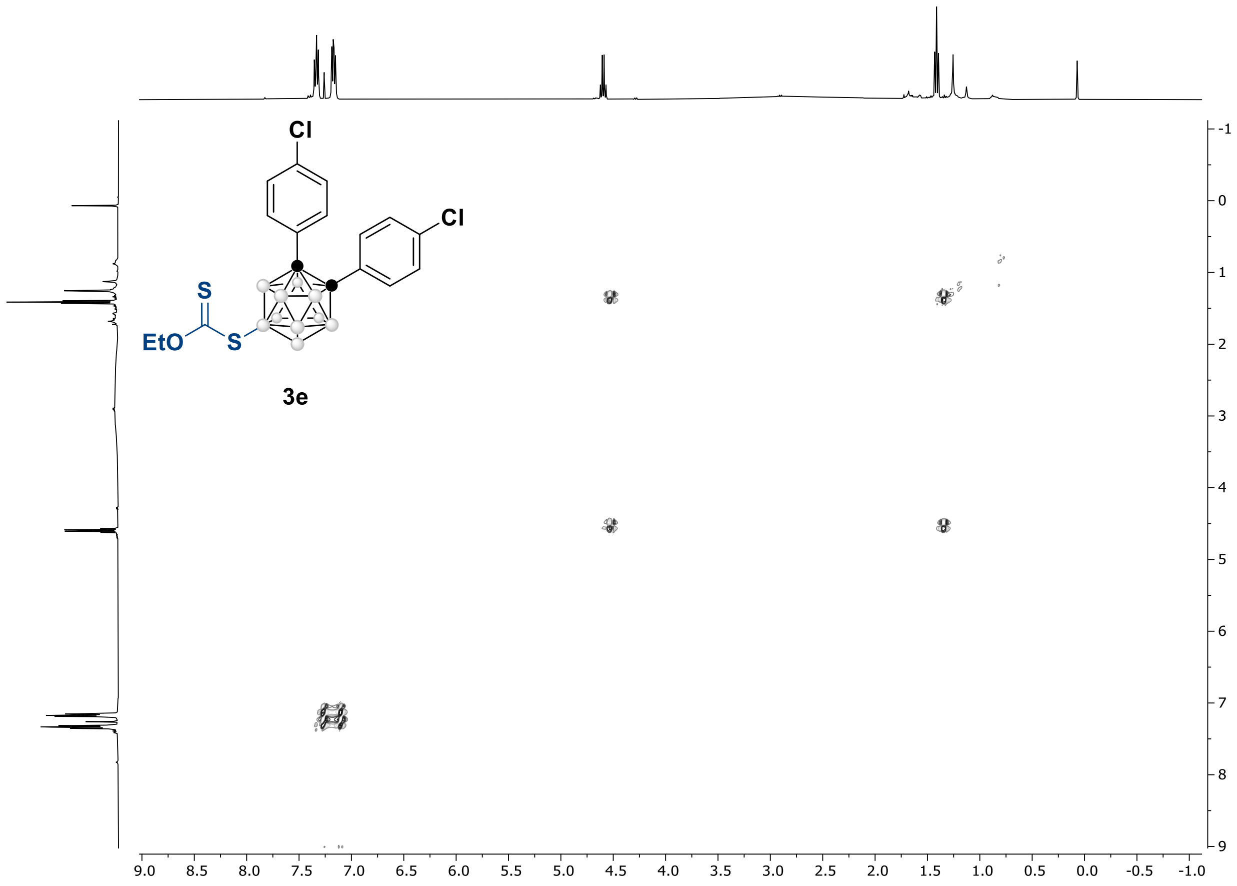

^{1}H-NMR (400 MHz, $CDCl_3$)

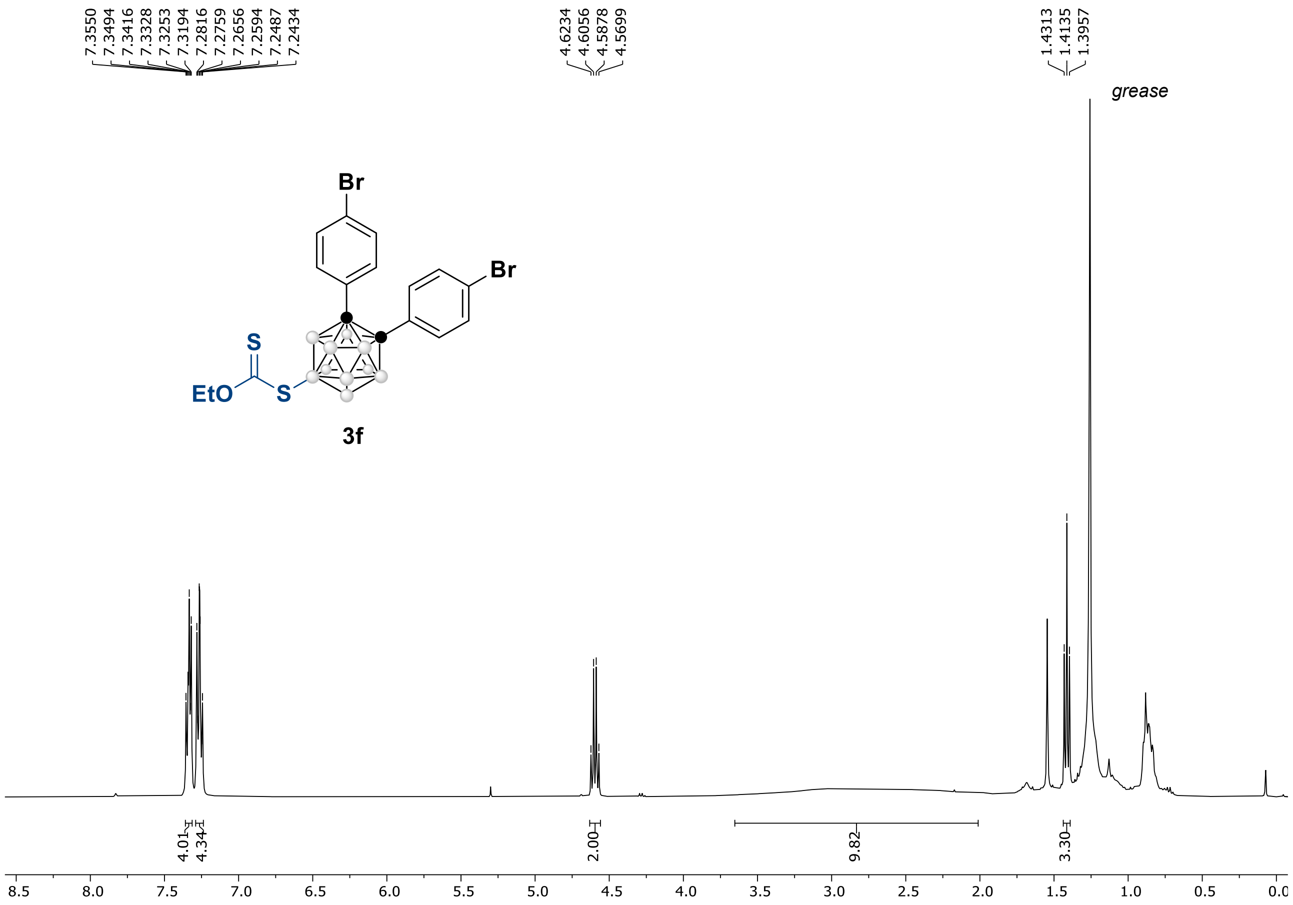

$^{13}C\{^{1}H\}$-NMR (100 MHz, $CDCl_3$)

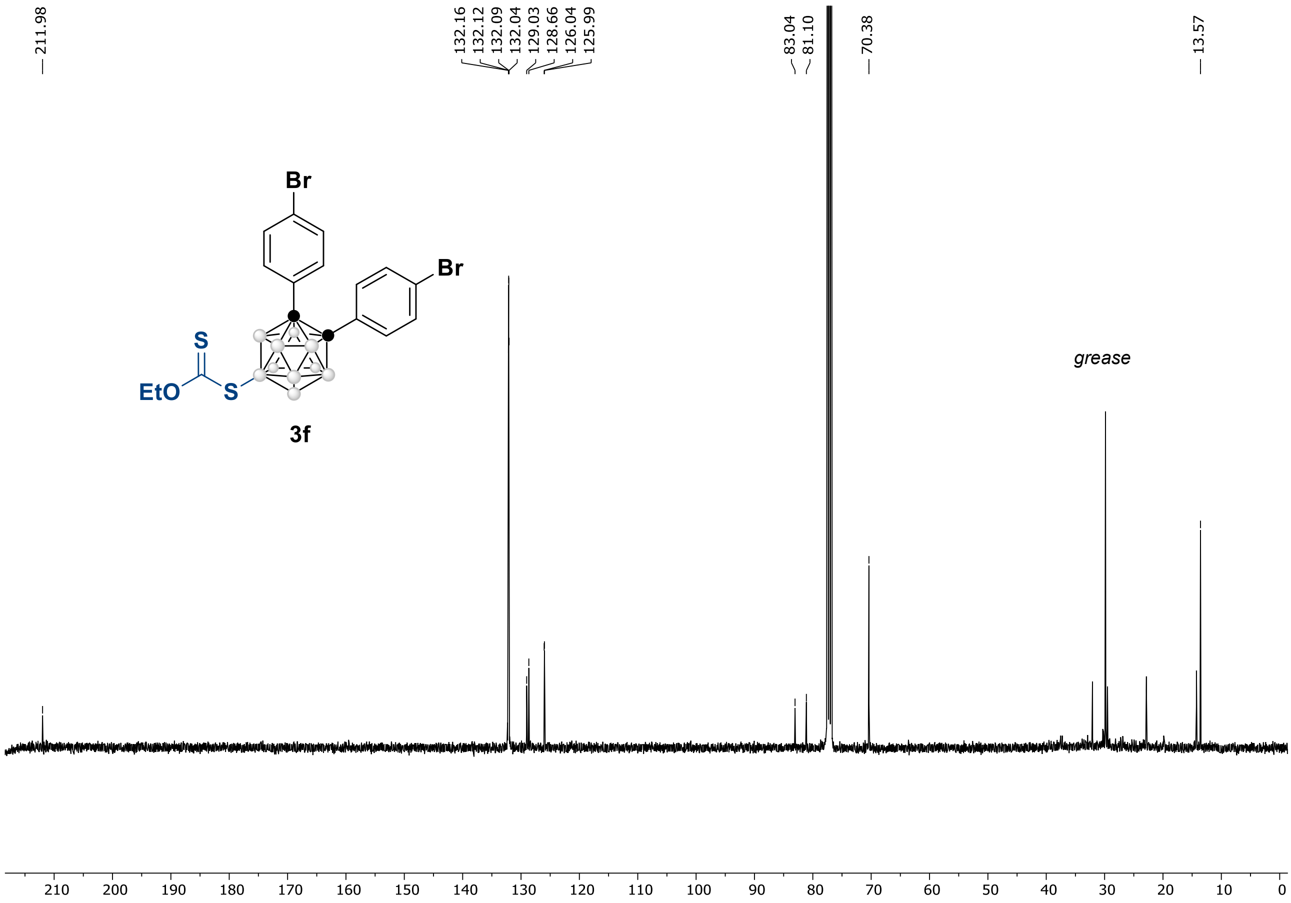

DEP135 (100 MHz, $CDCl_3$)

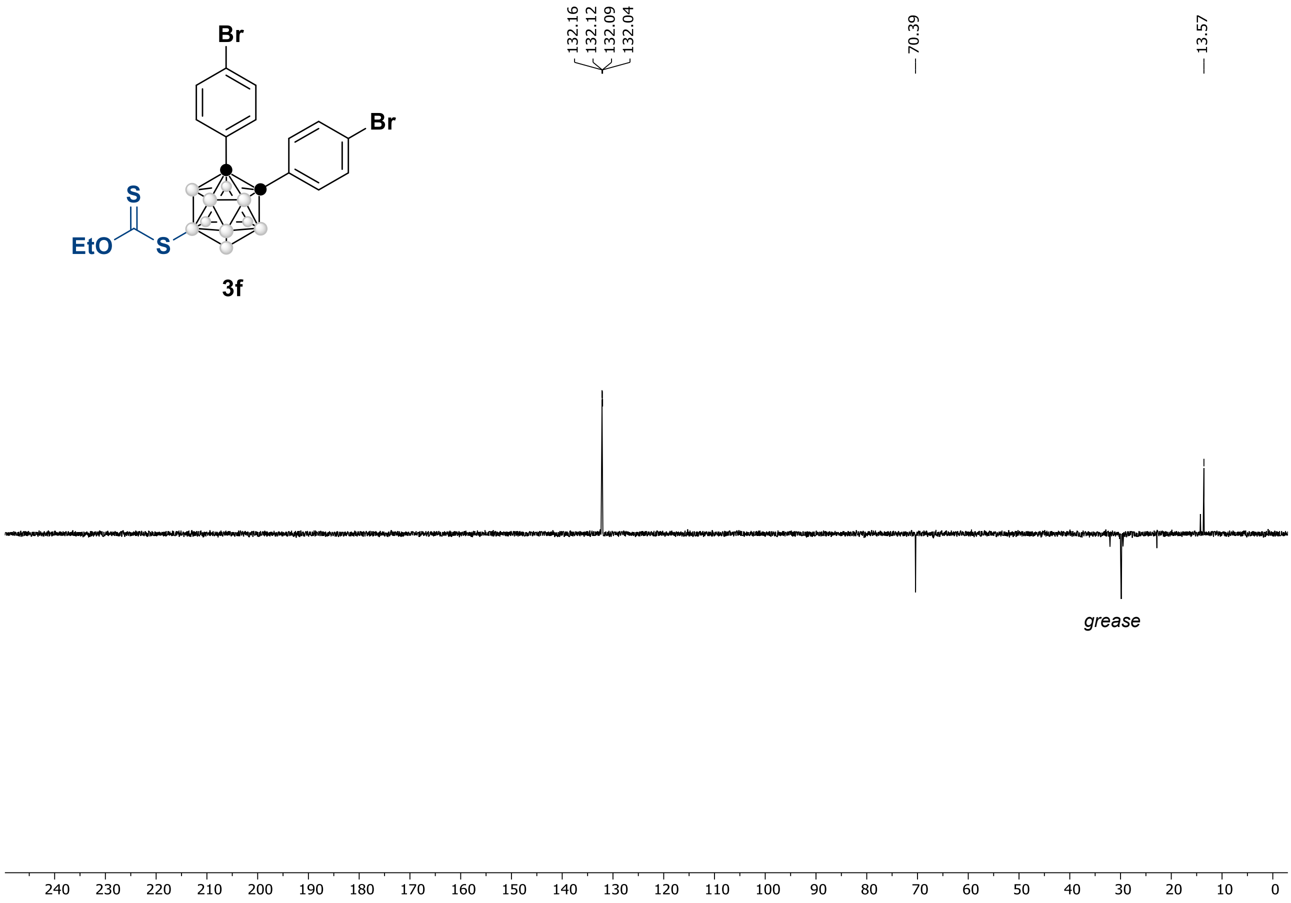

$^{11}B\{^{1}H\}$-NMR (128 MHz, $CDCl_3$)

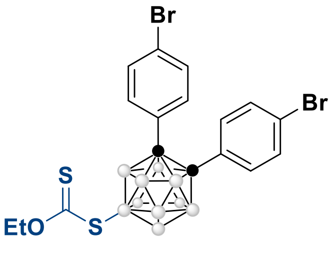

3f

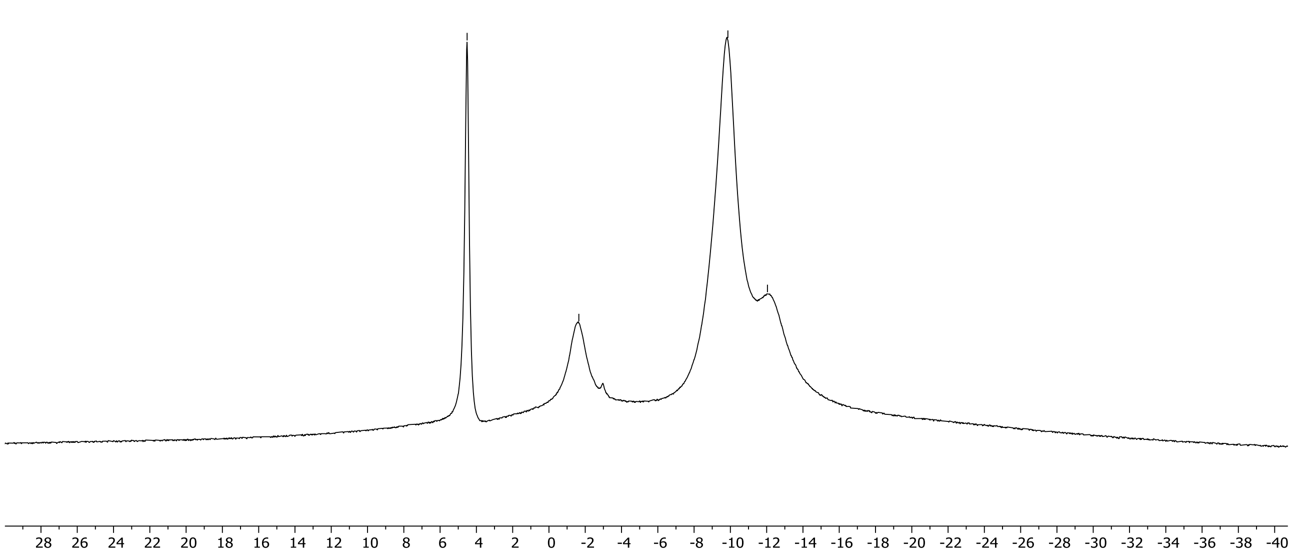

2D – COSY (400 MHz, $CDCl_3$)

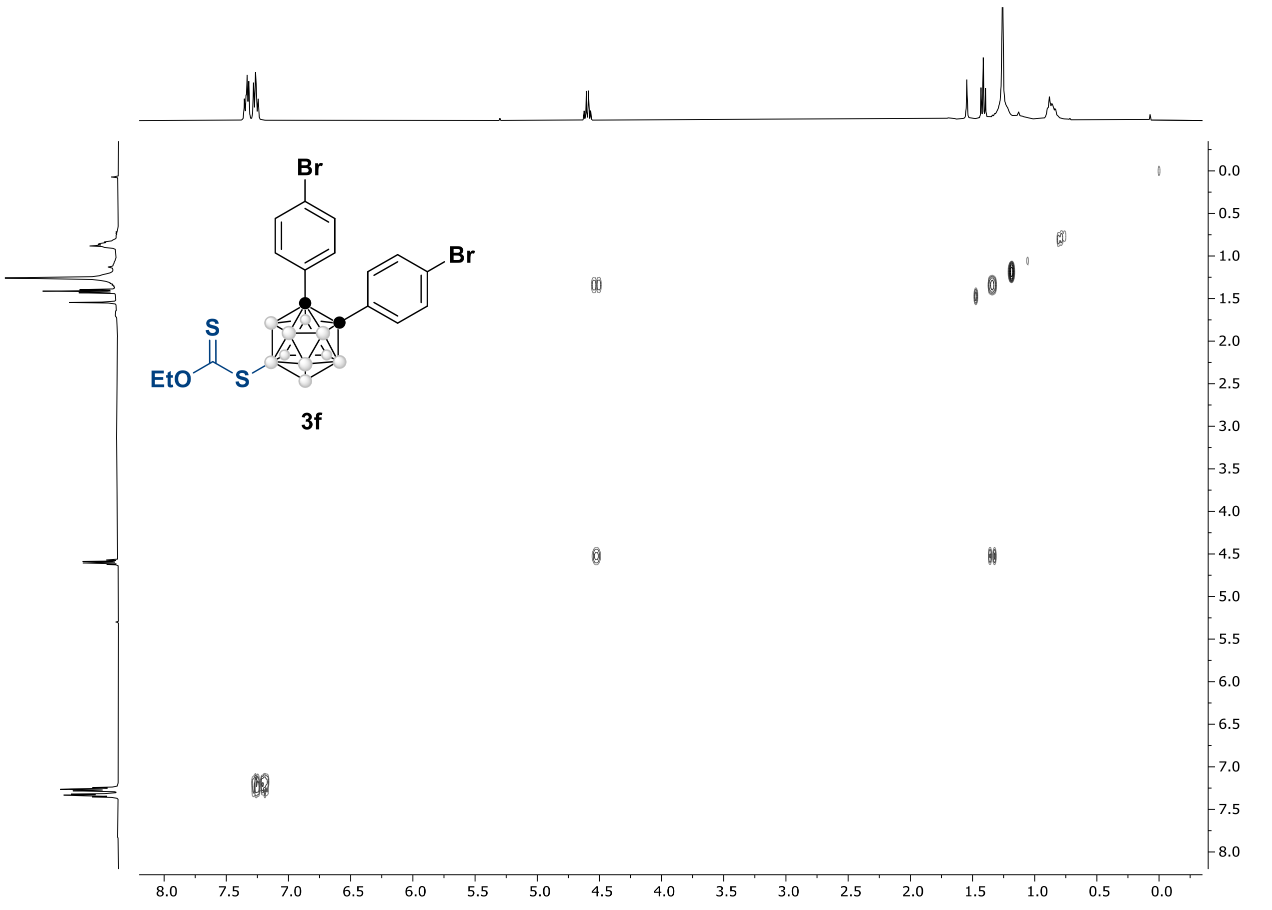

^{1}H-NMR (600 MHz, $CDCl_3$)

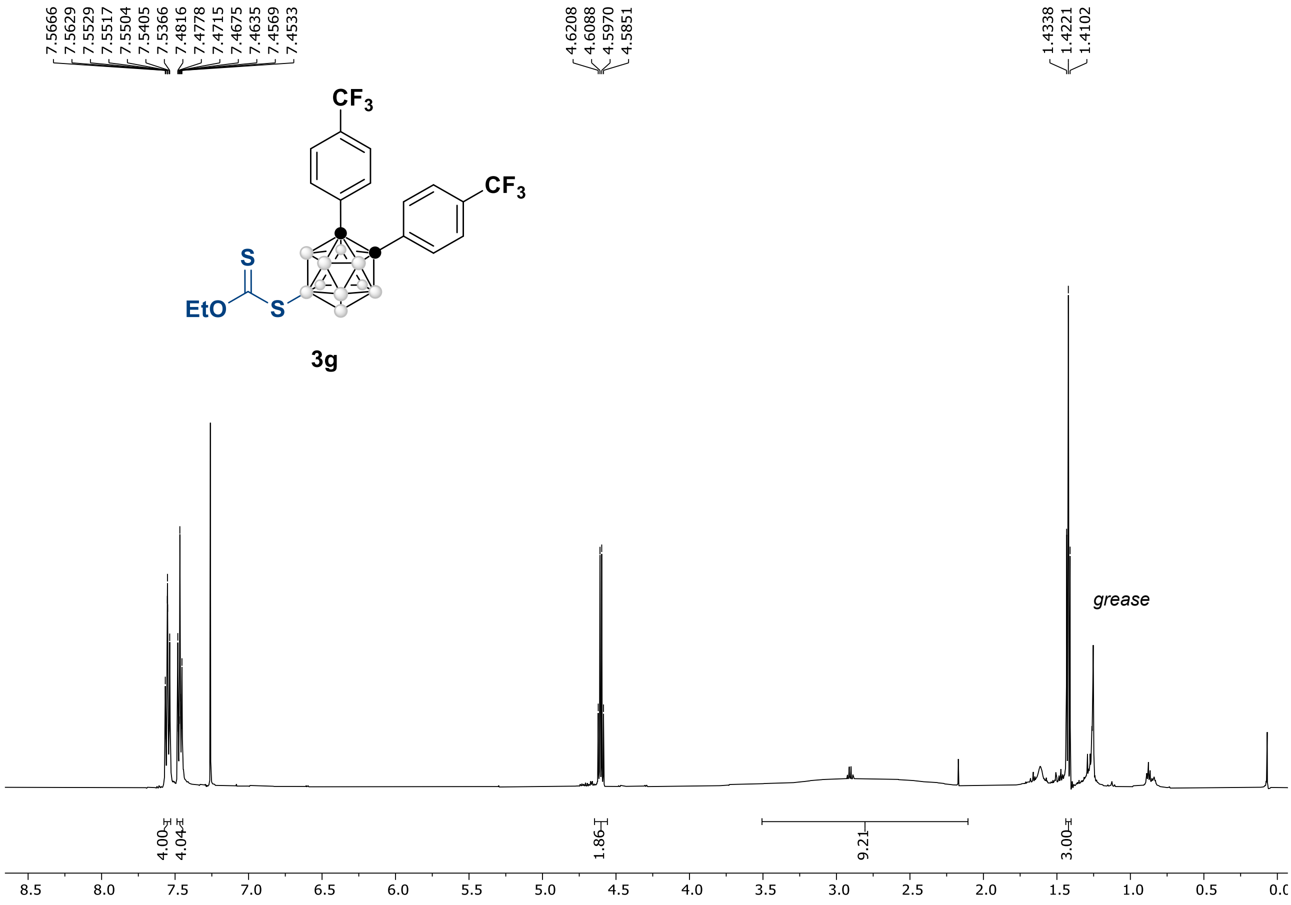

^{13}C{^{1}H}-NMR (150 MHz, $CDCl_3$)

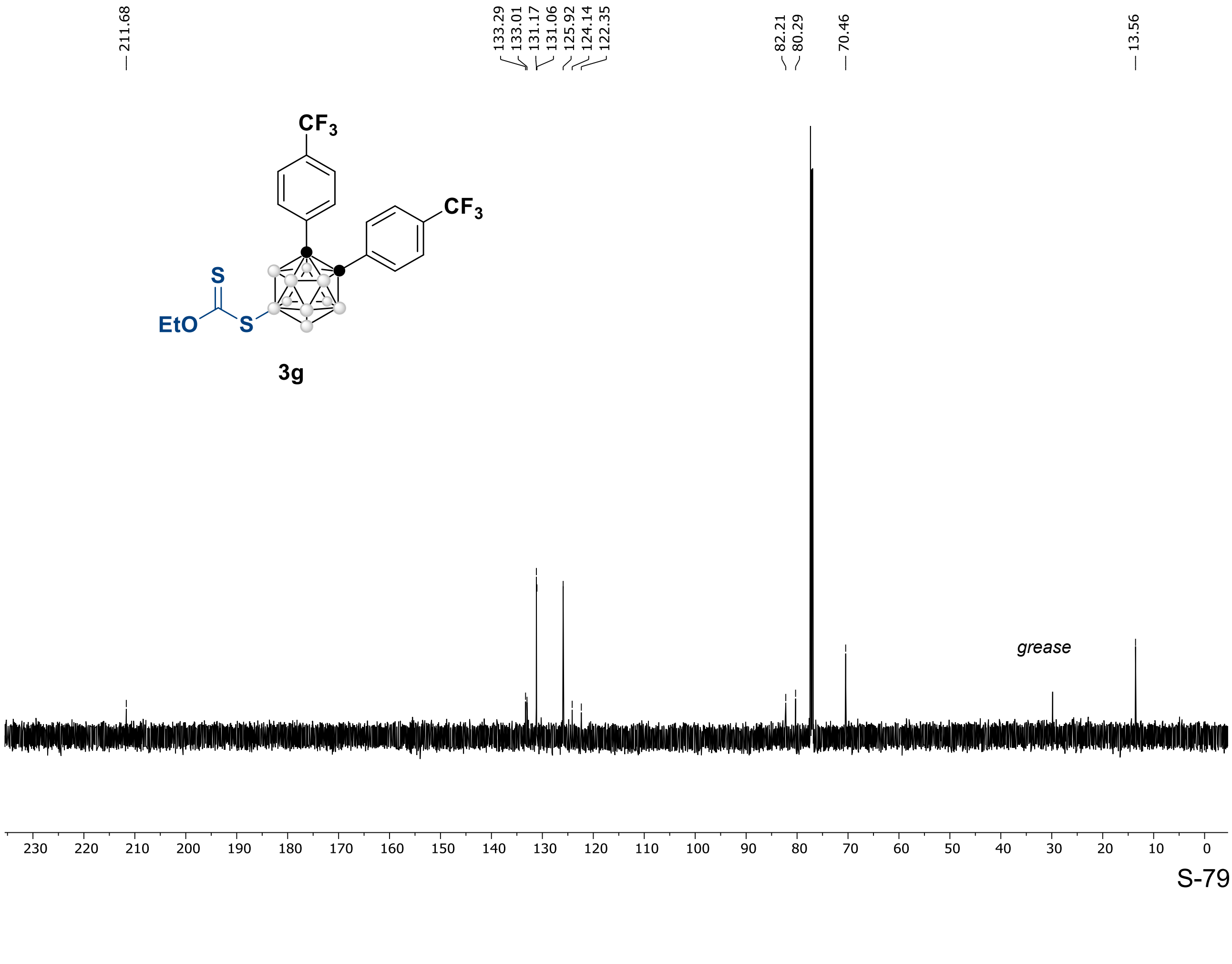

DEPT135 (150 MHz, $CDCl_3$)

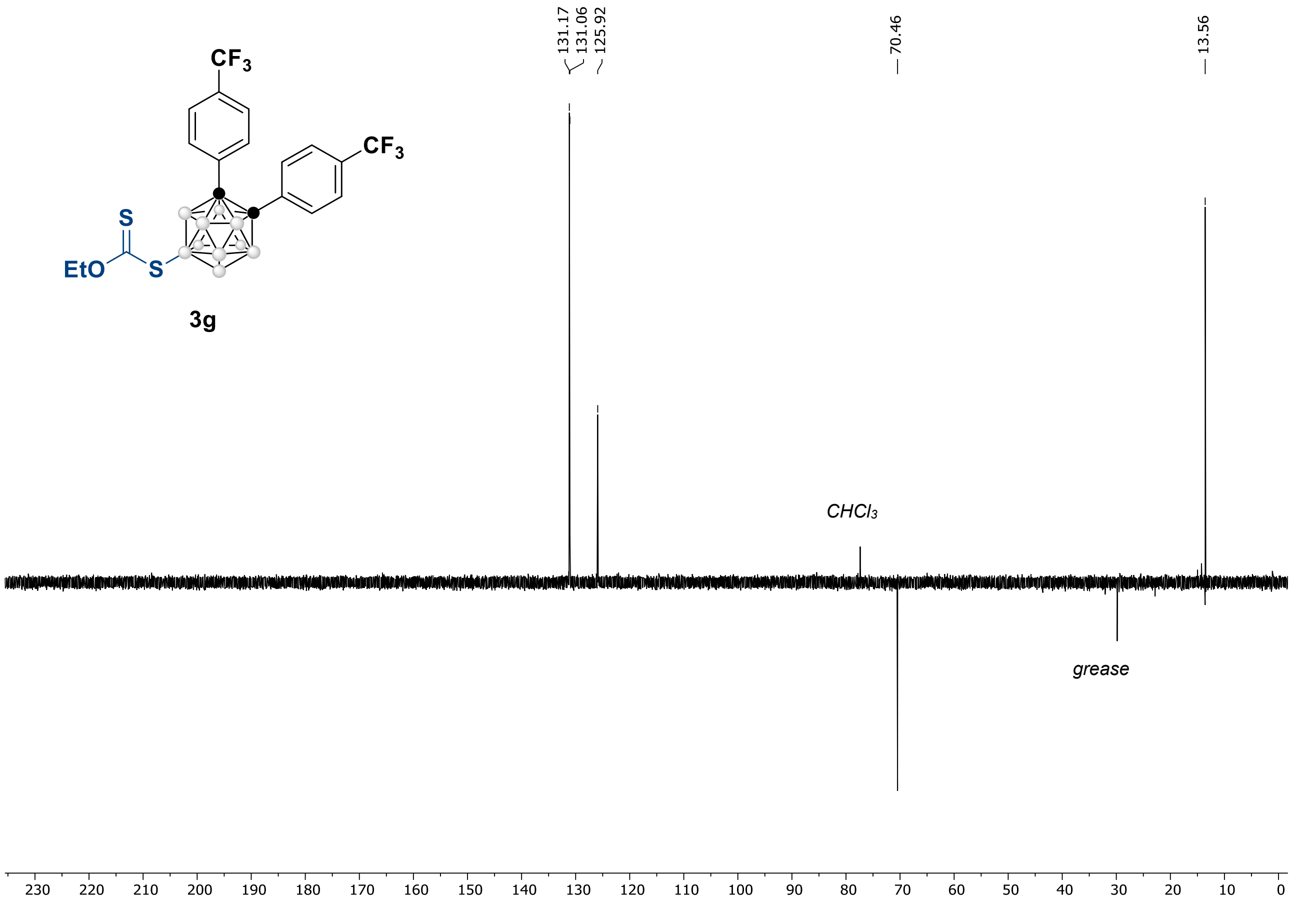

$^{11}B\{^1H\}$-NMR (192 MHz, $CDCl_3$)

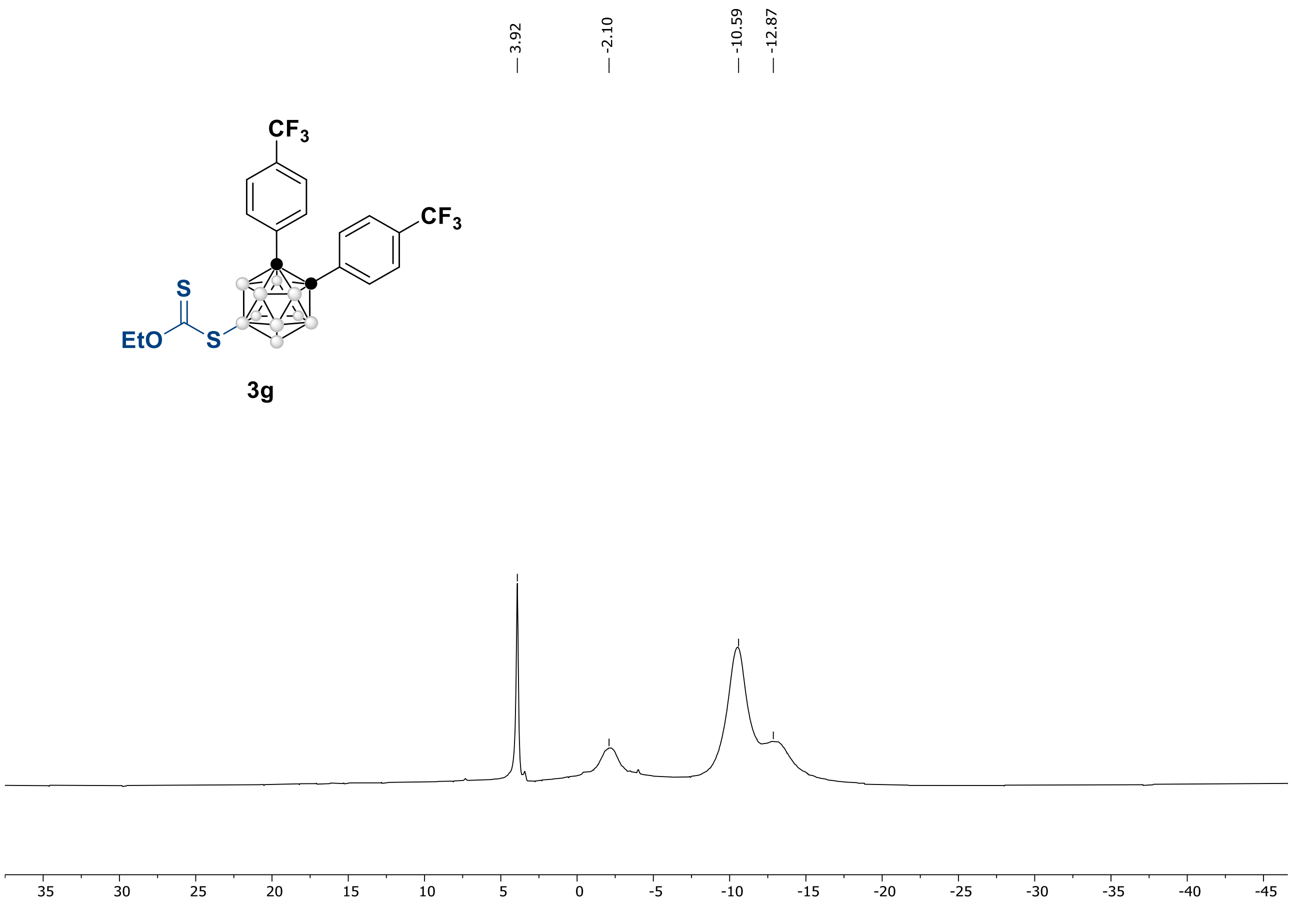

$^{19}F\{^1H\}$-NMR (564 MHz, $CDCl_3$)

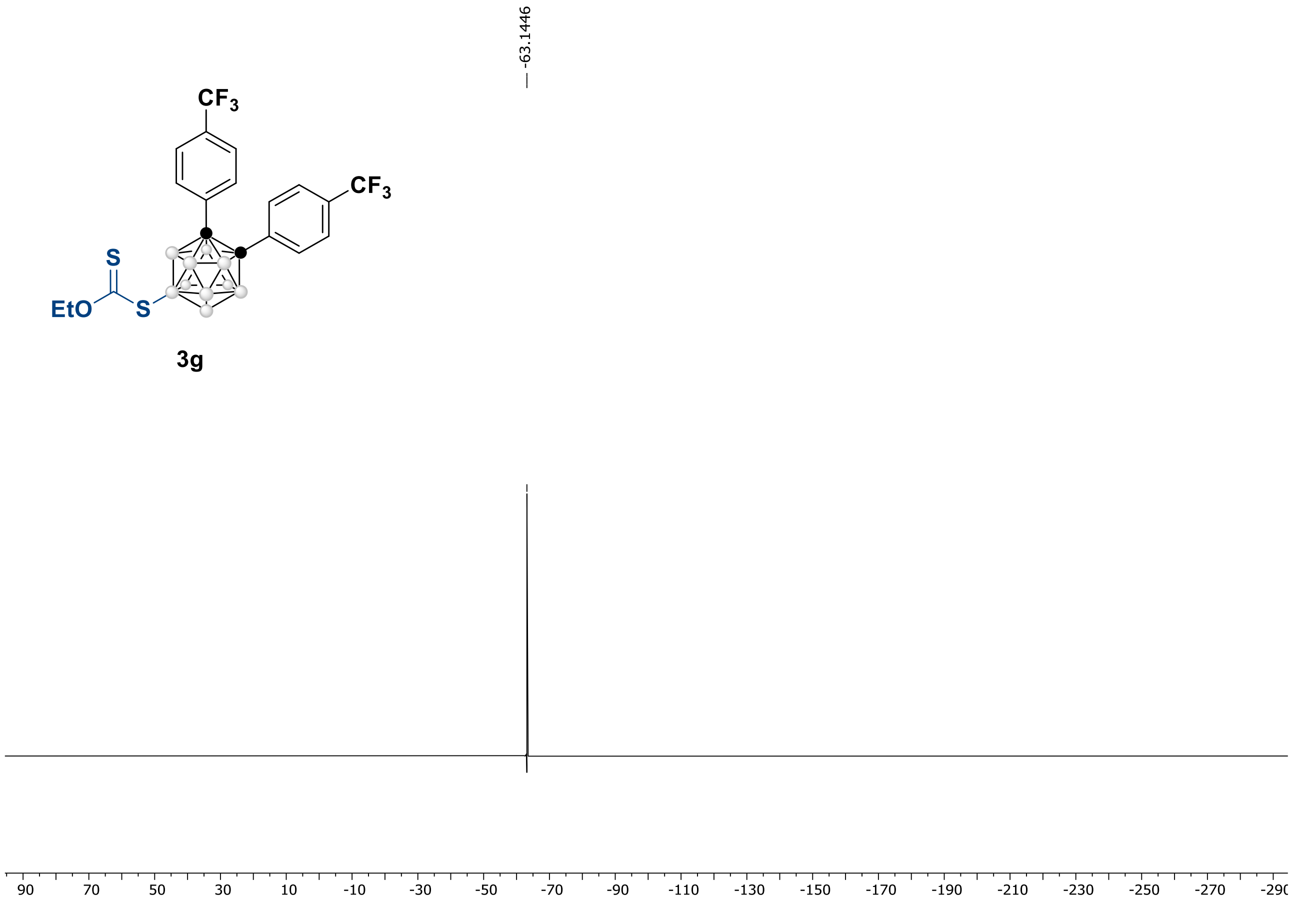

2D – COSY (600 MHz, $CDCl_3$)

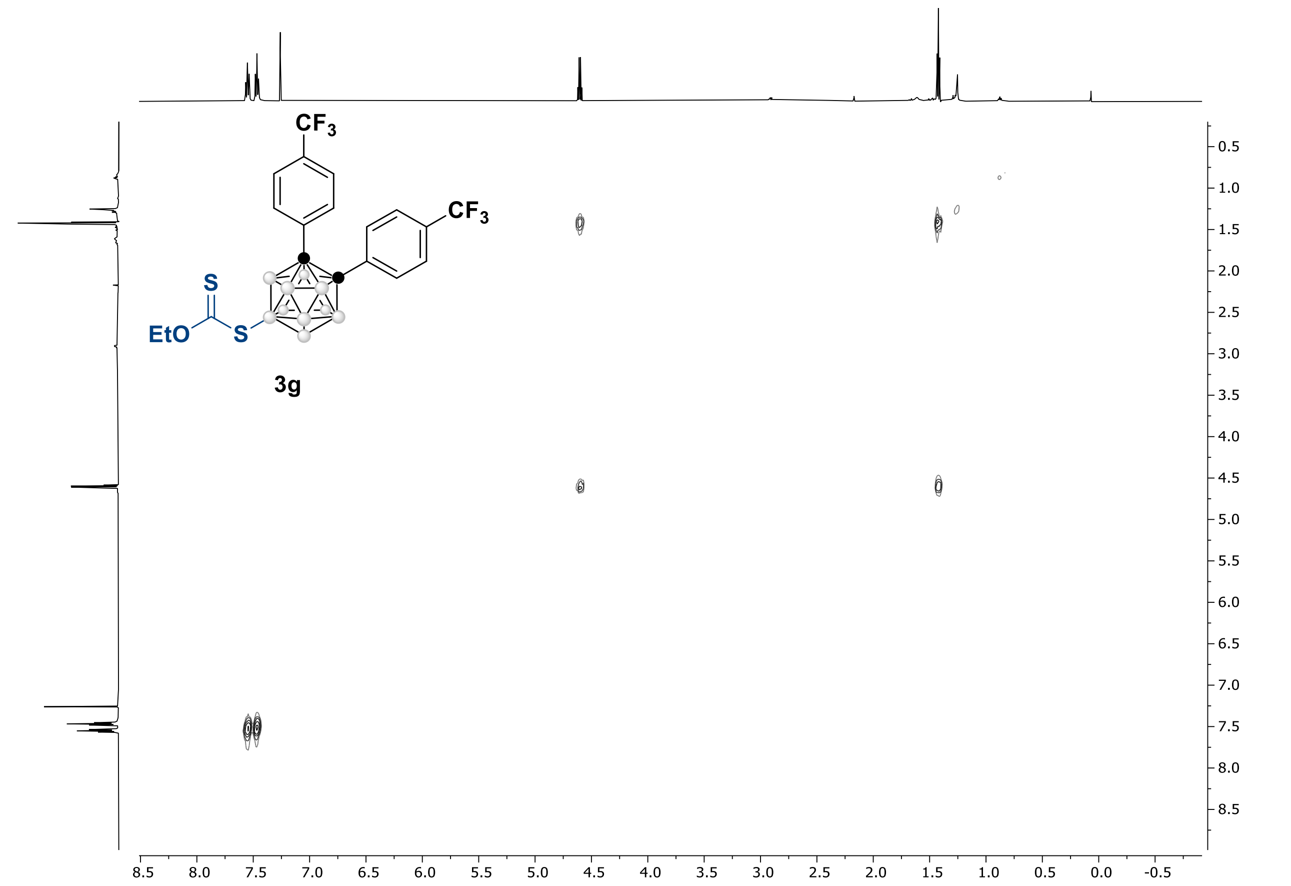

^{1}H-NMR (600 MHz, $CDCl_3$)

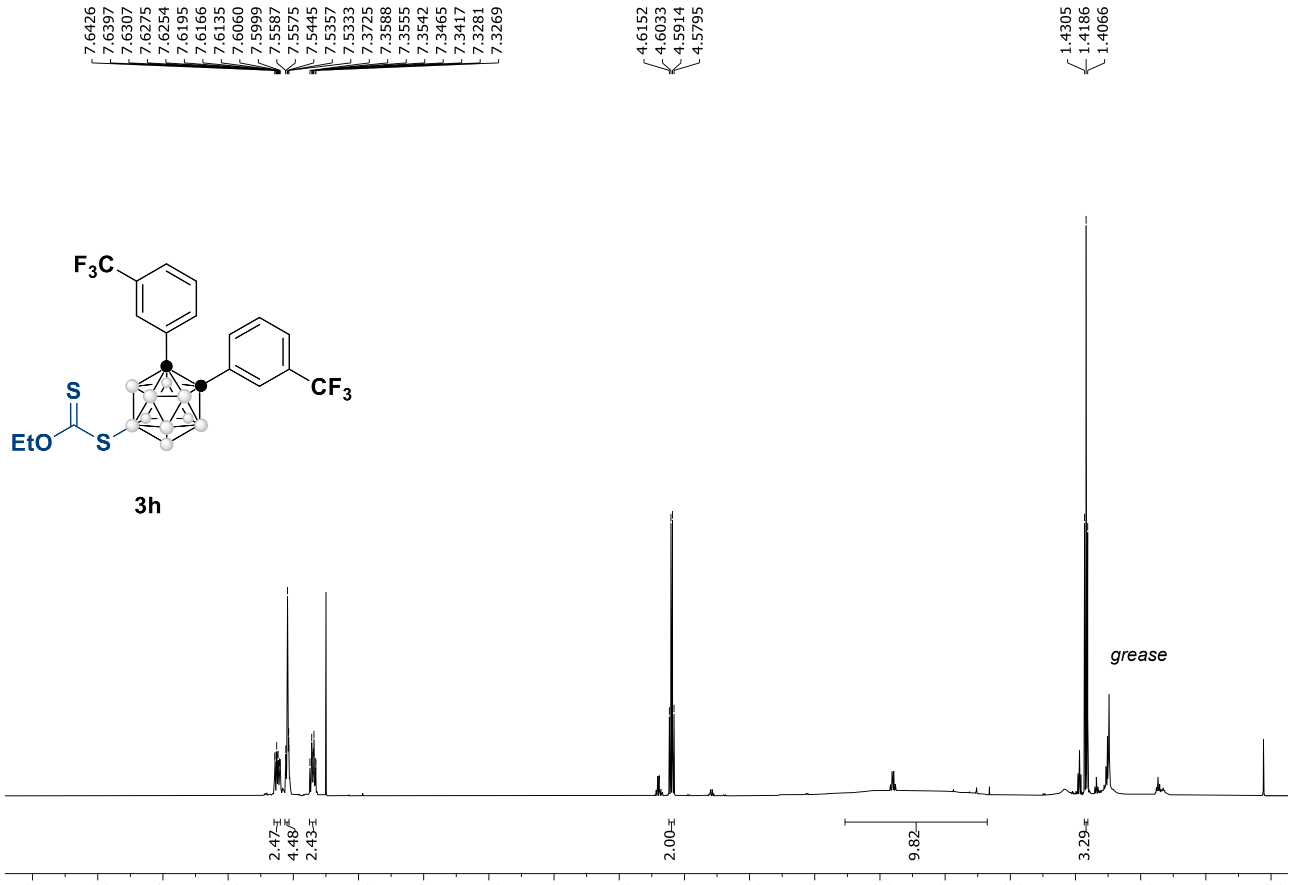

^{13}C{^{1}H}-NMR (150 MHz, $CDCl_3$)

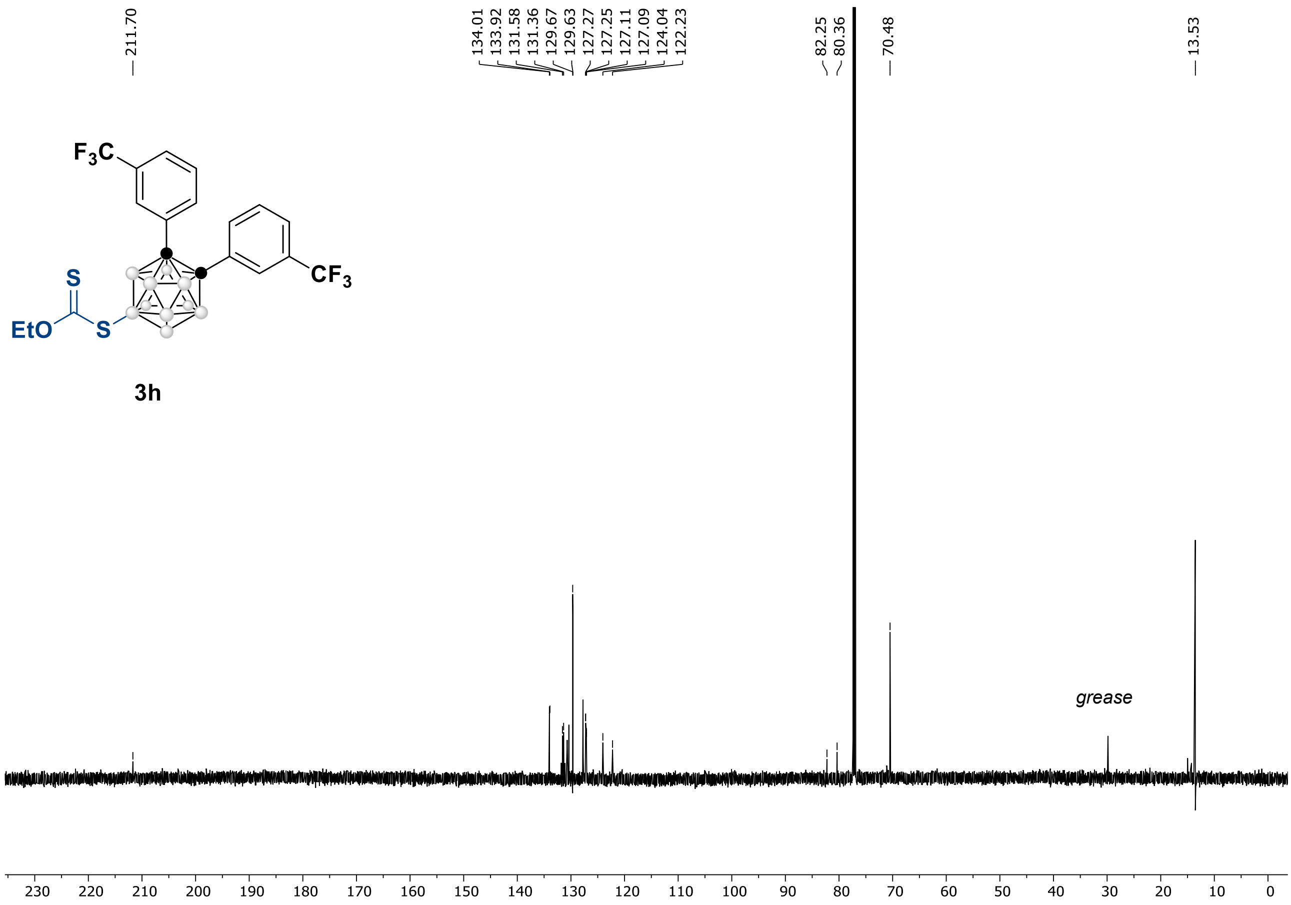

DEPT135 (150 MHz, $CDCl_3$)

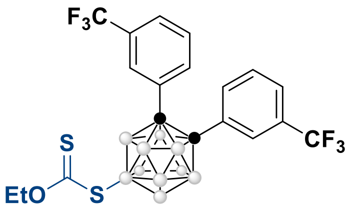

3h

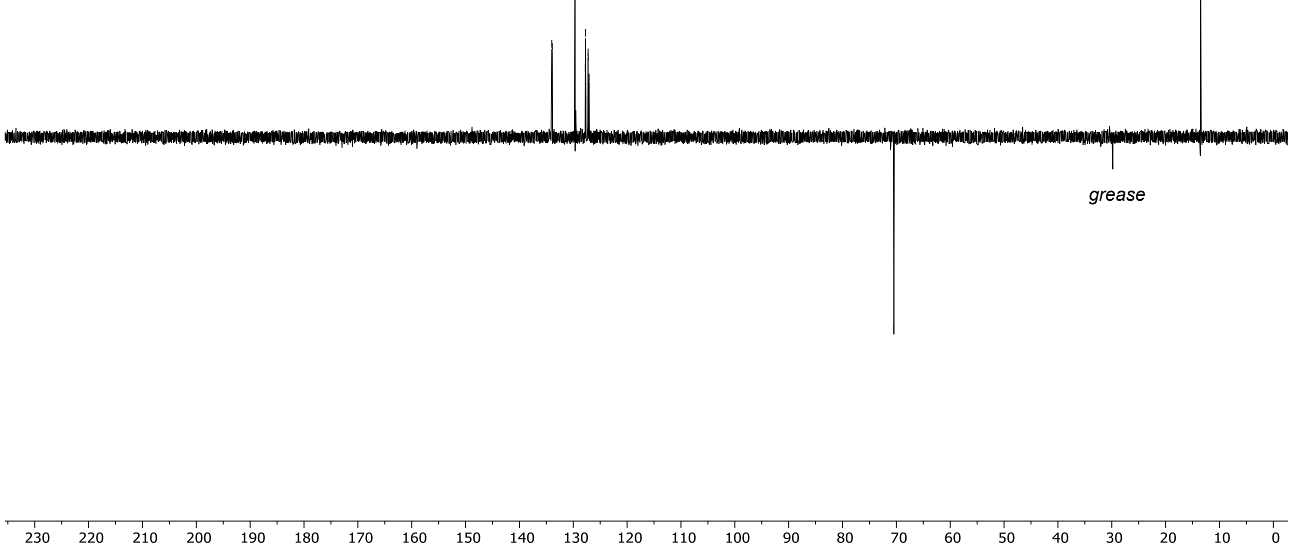

$^{11}B\{^1H\}$-NMR (192 MHz, $CDCl_3$)

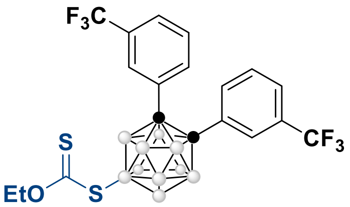

3h

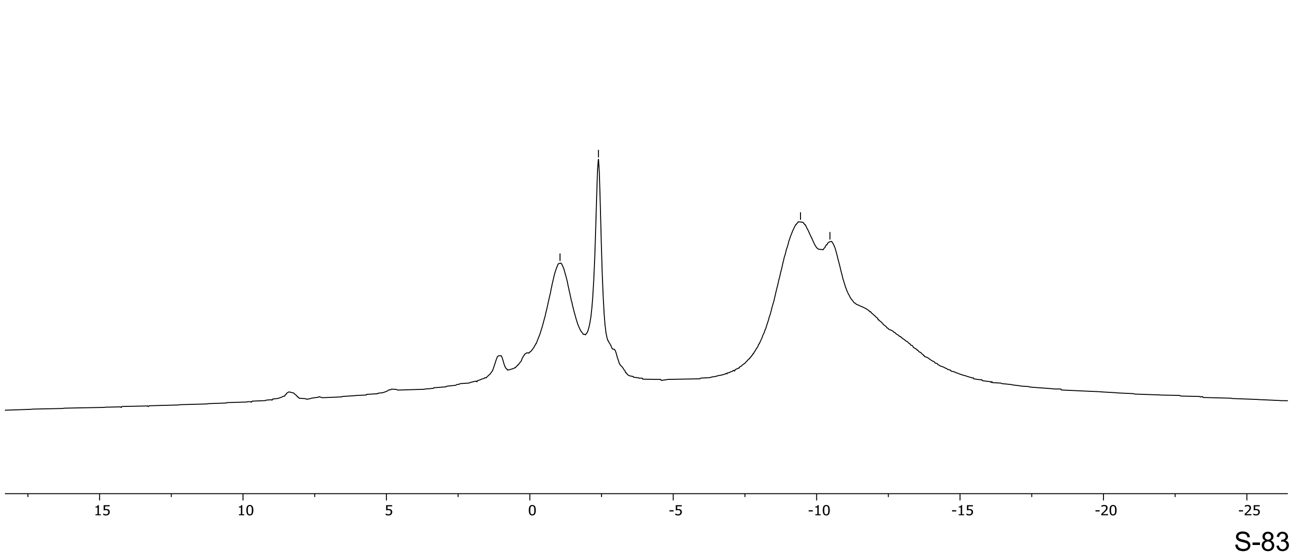

^{19}F{^{1}H}-NMR (564 MHz, $CDCl_3$)

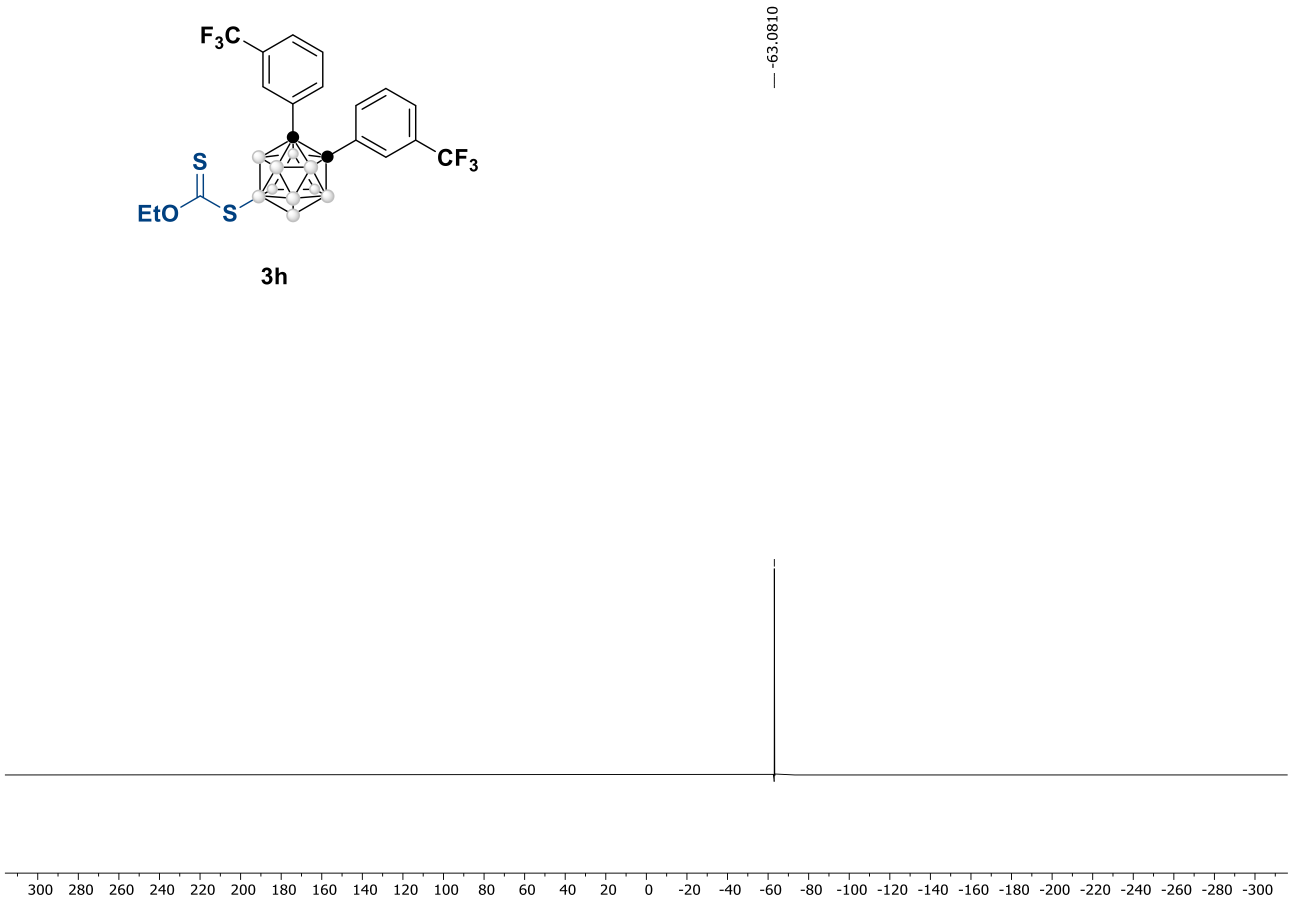

2D – COSY (600 MHz, $CDCl_3$)

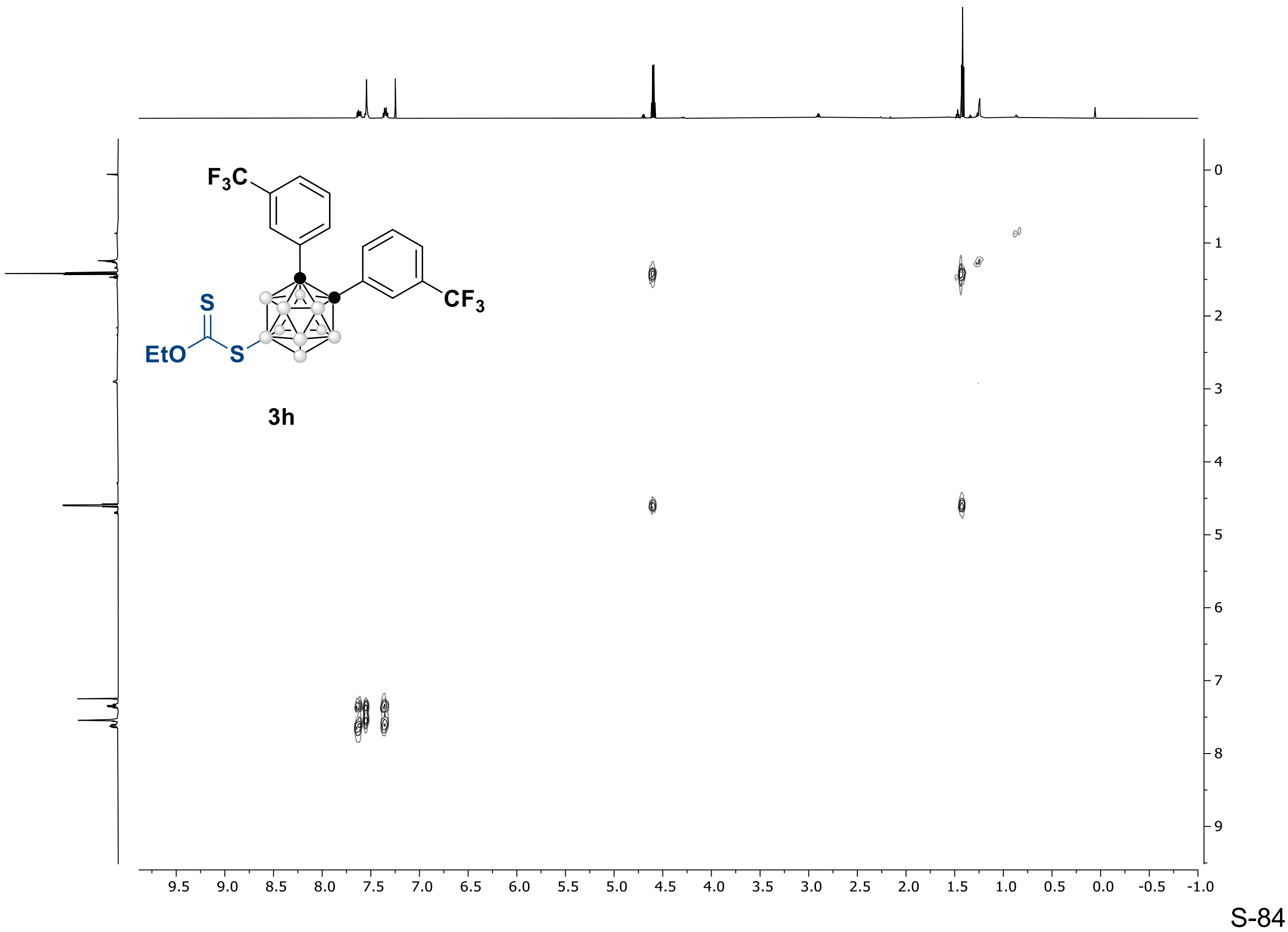

^{1}H-NMR (400 MHz, $CDCl_3$)

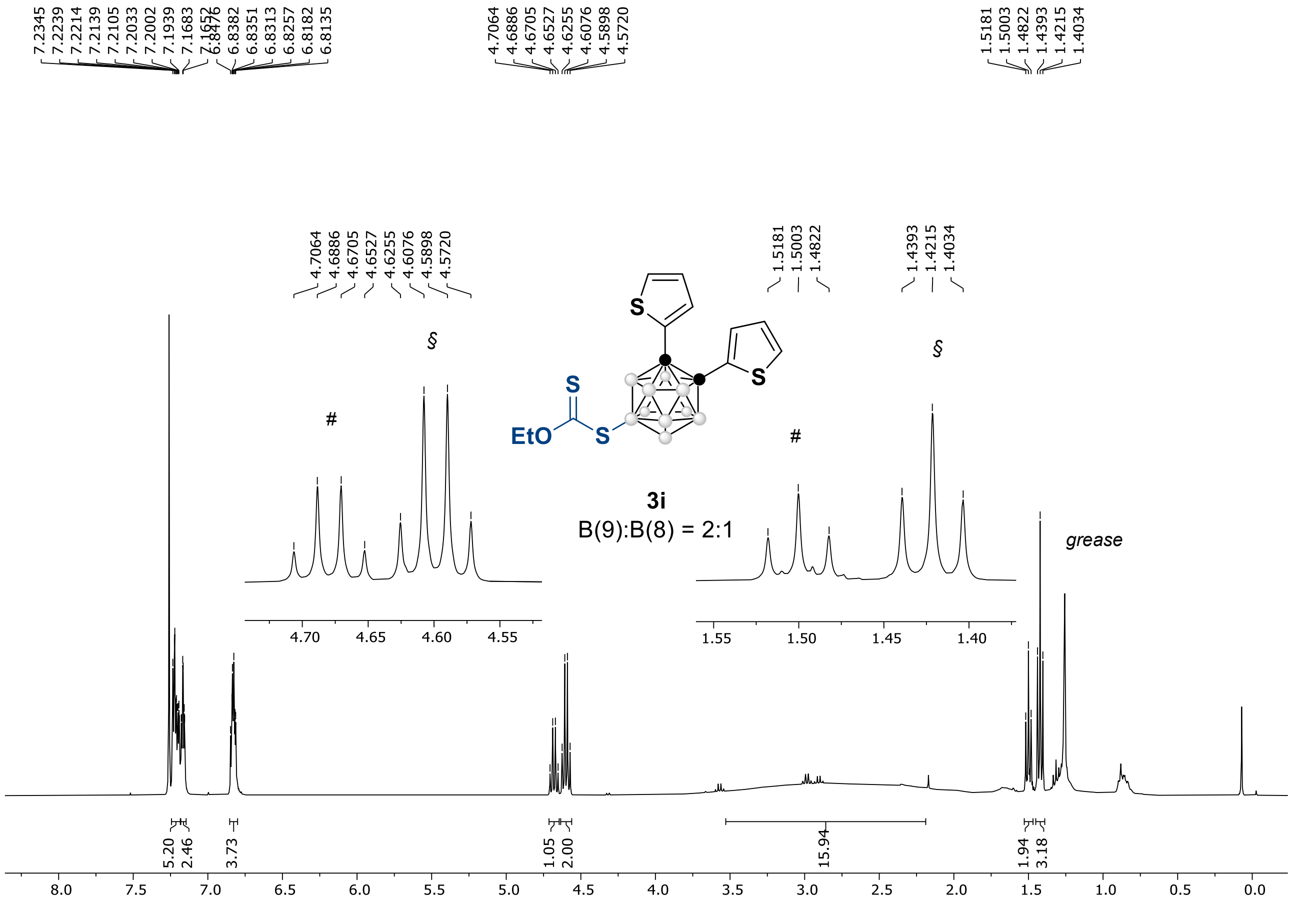

^{13}C{^{1}H}-NMR (100 MHz, $CDCl_3$)

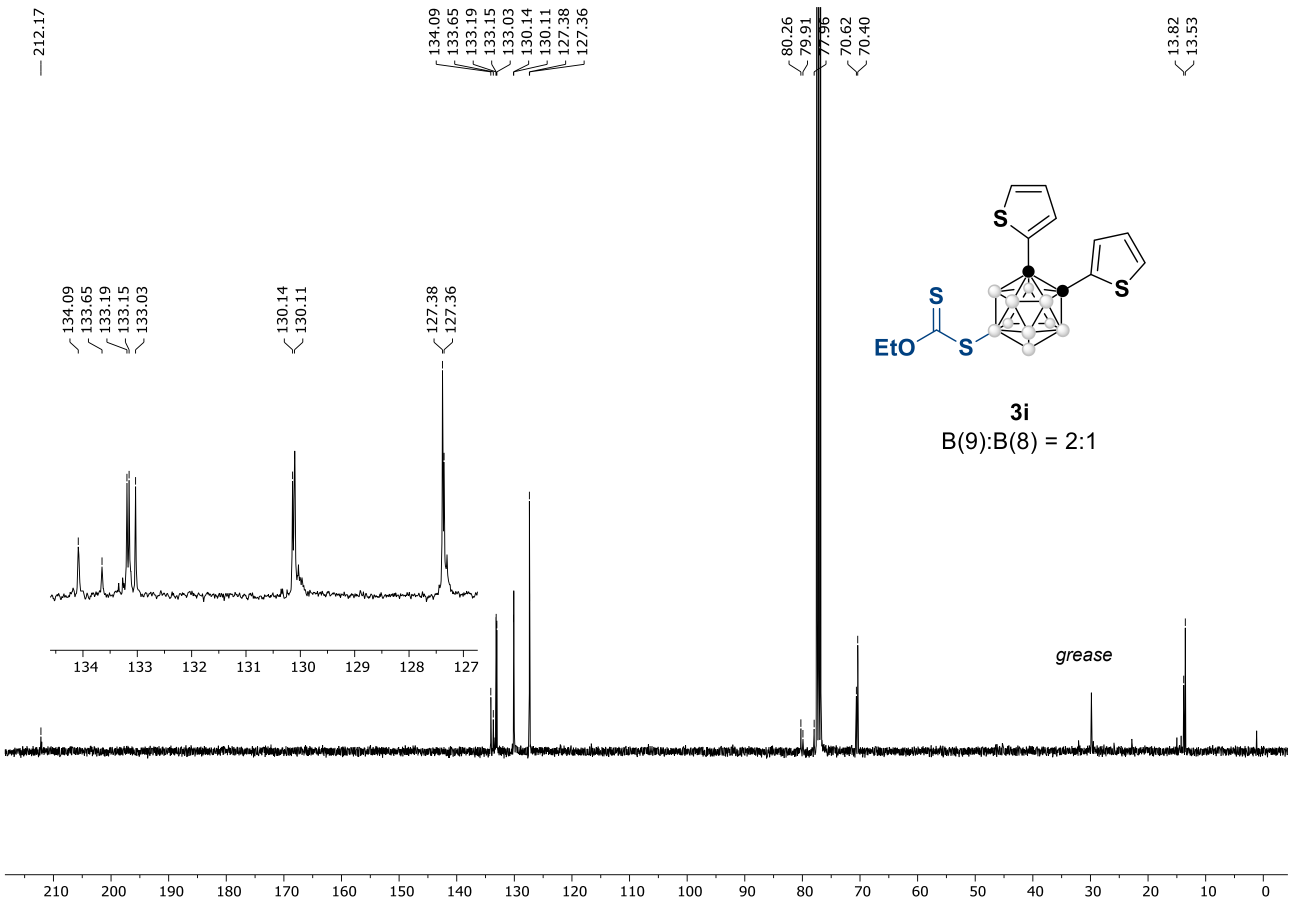

DEPT135 (100 MHz, $CDCl_3$)

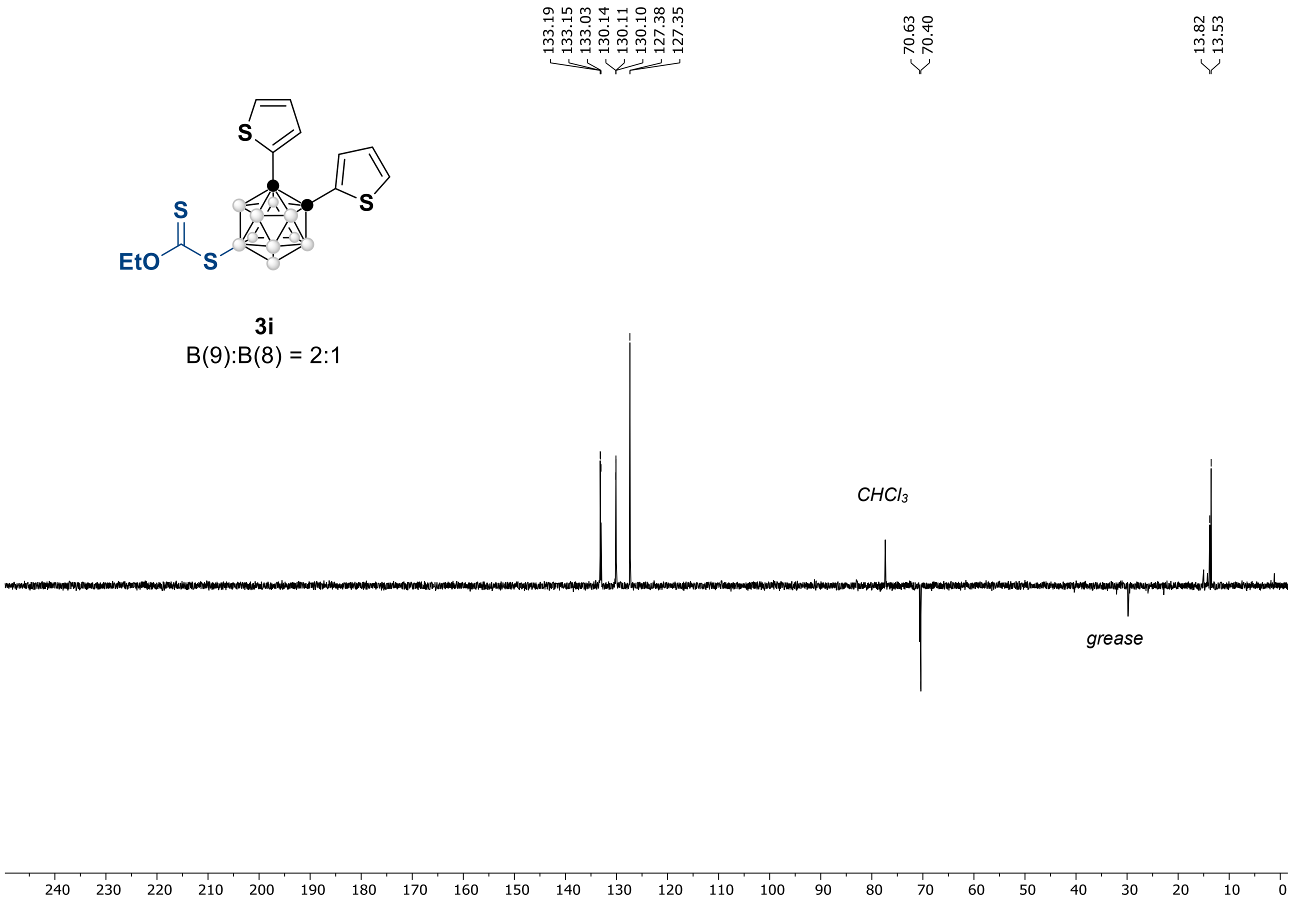

$^{11}B\{^1H\}$-NMR (128 MHz, $CDCl_3$)

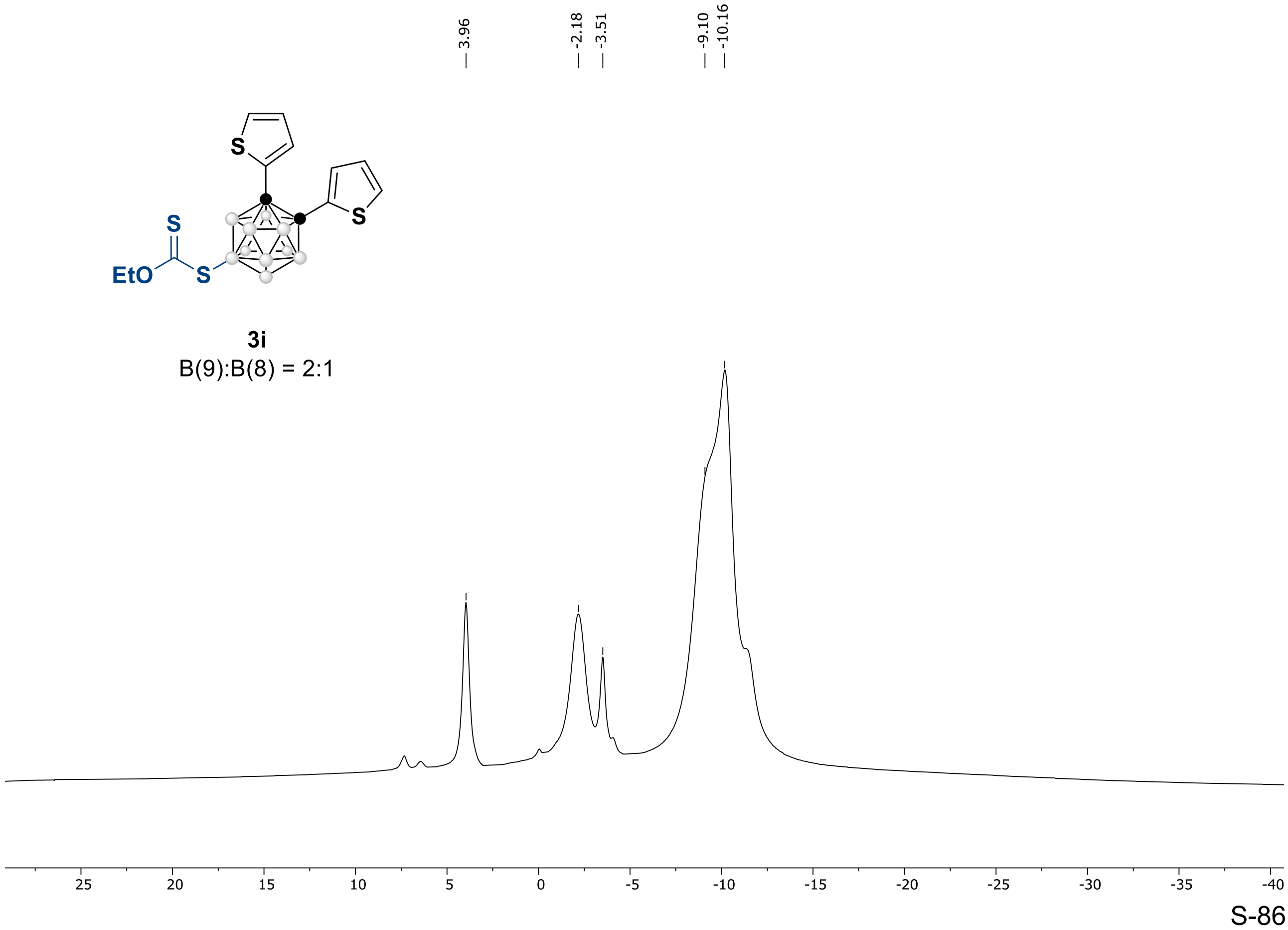

2D – COSY (400 MHz, $CDCl_3$)

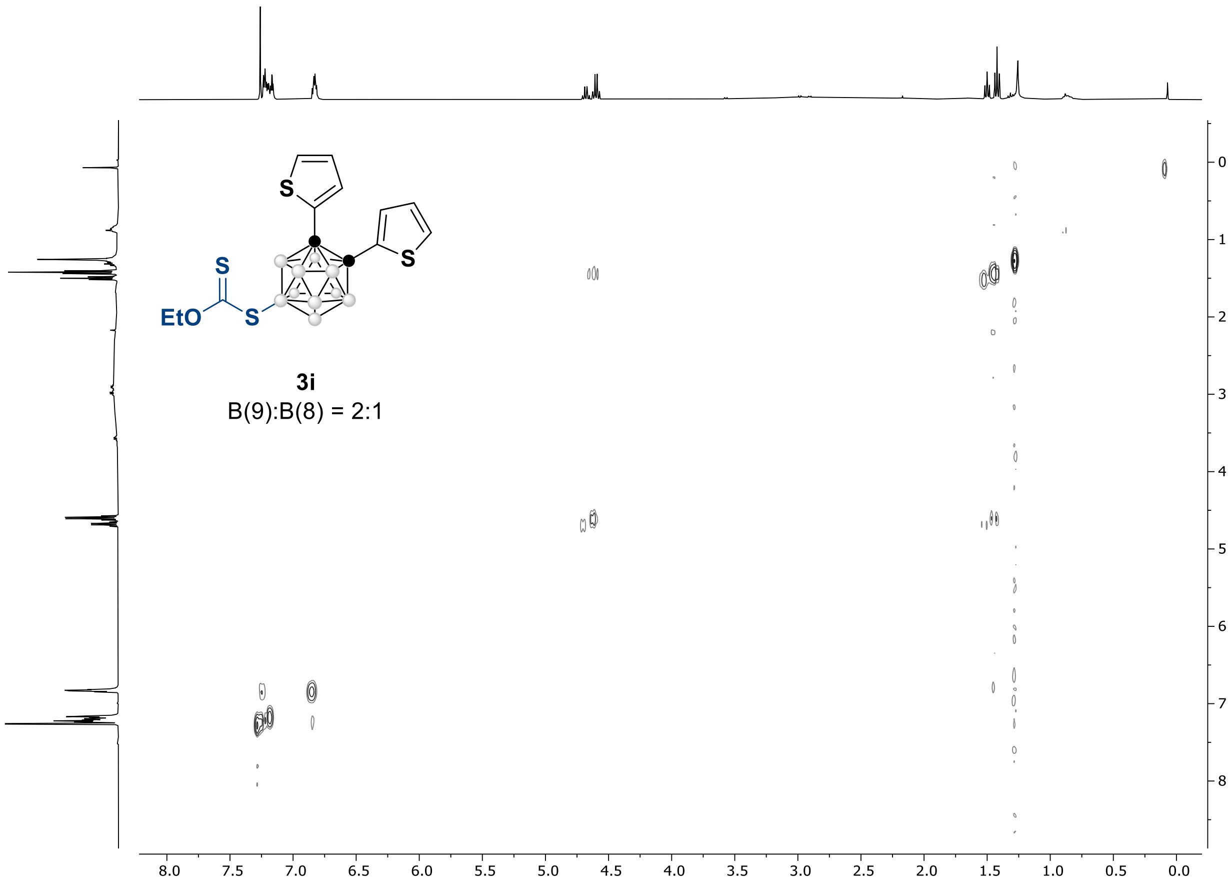

^{1}H-NMR (400 MHz, $CDCl_3$)

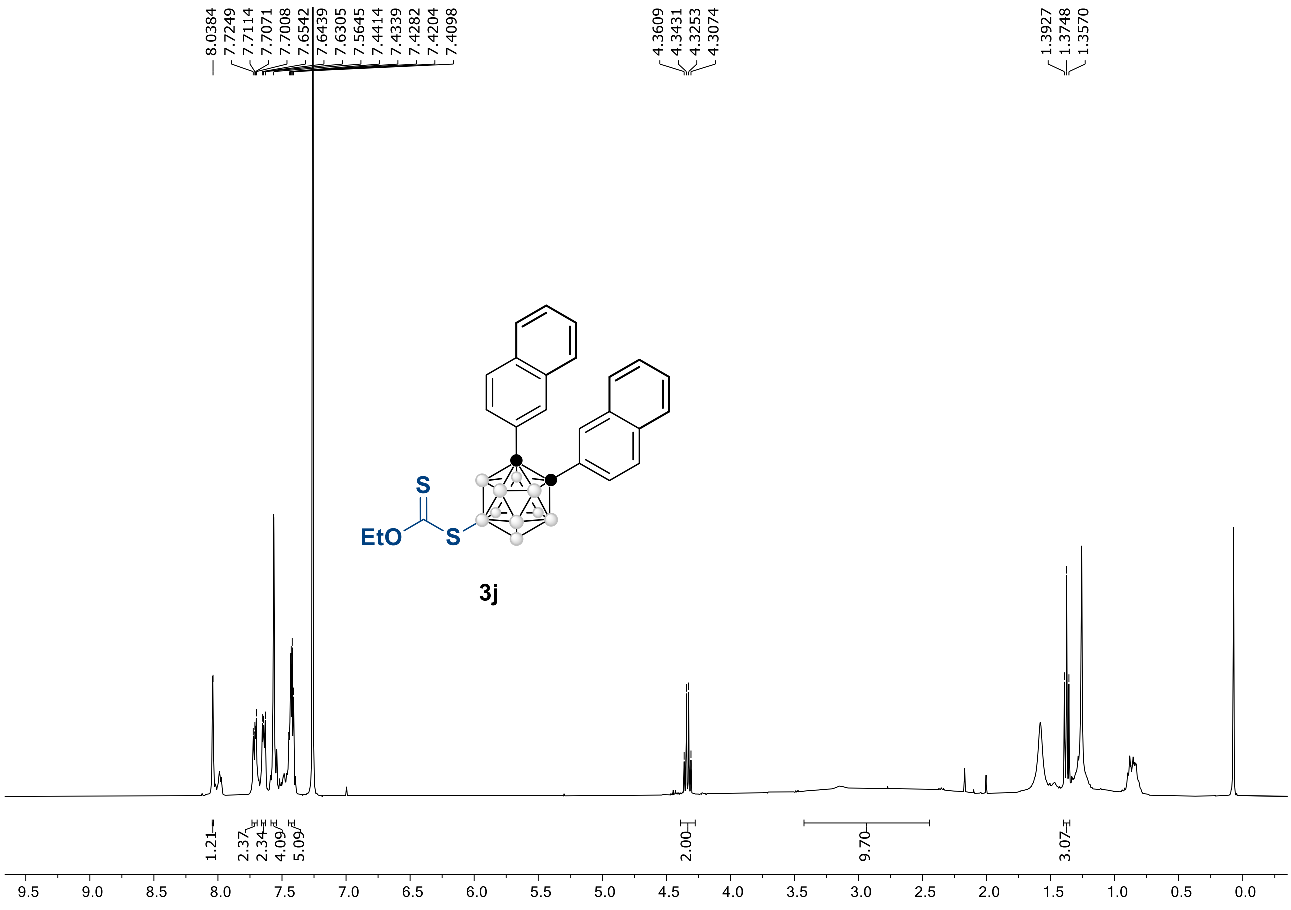

$^{13}C\{^1H\}$-NMR (100 MHz, $CDCl_3$)

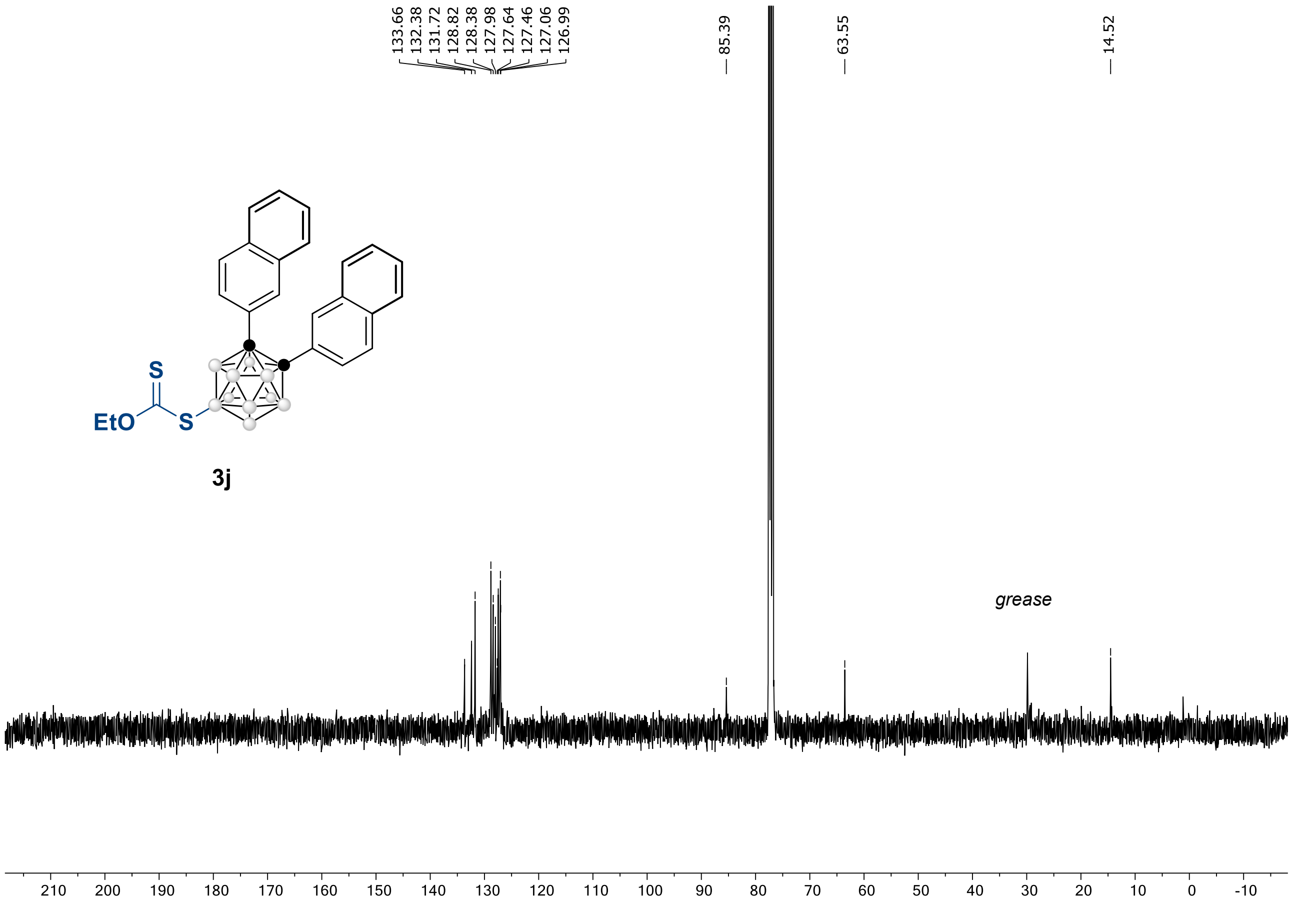

DEPT-135 (100 MHz, $CDCl_3$)

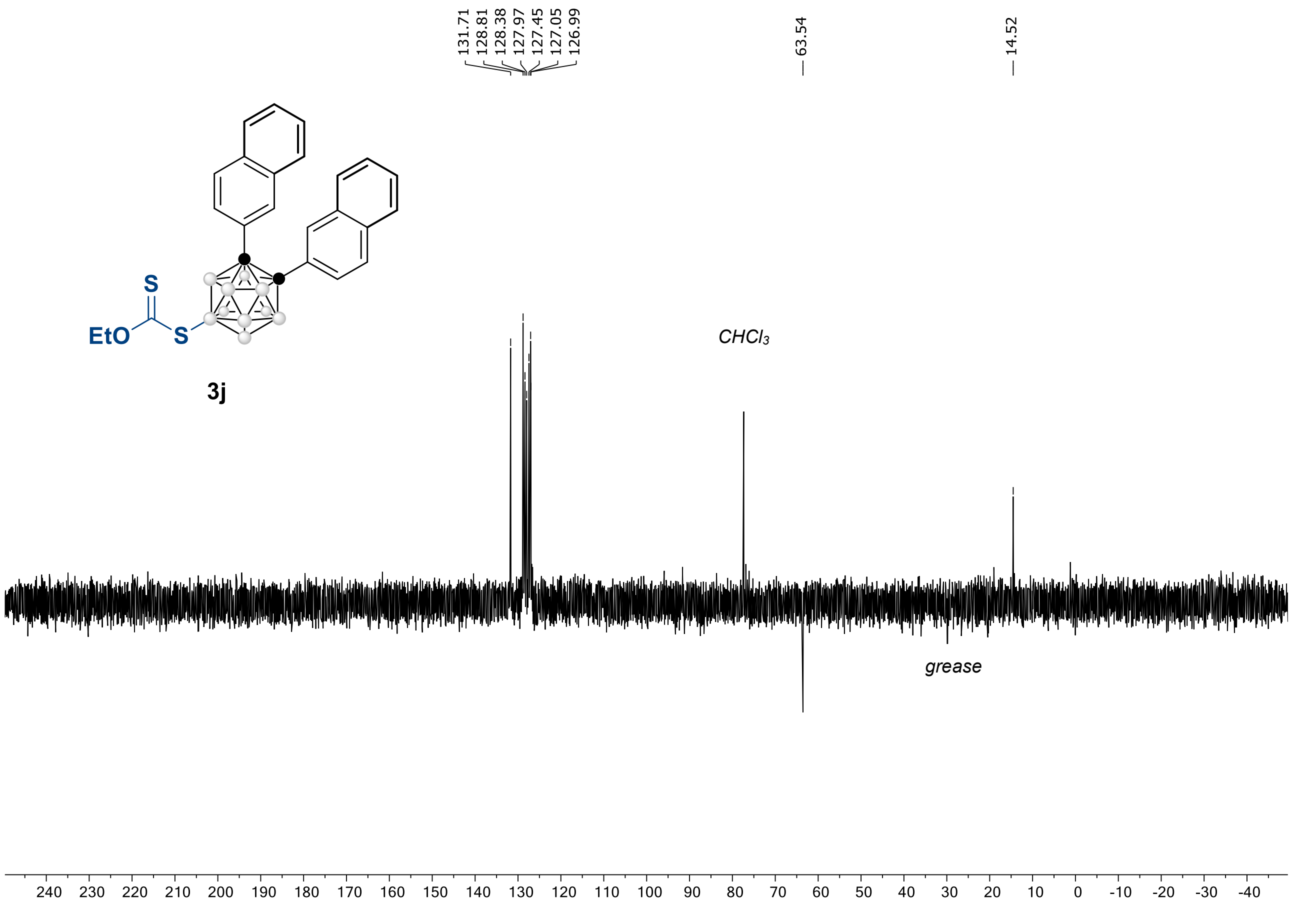

$^{11}B\{^{1}H\}$-NMR (128 MHz, $CDCl_3$)

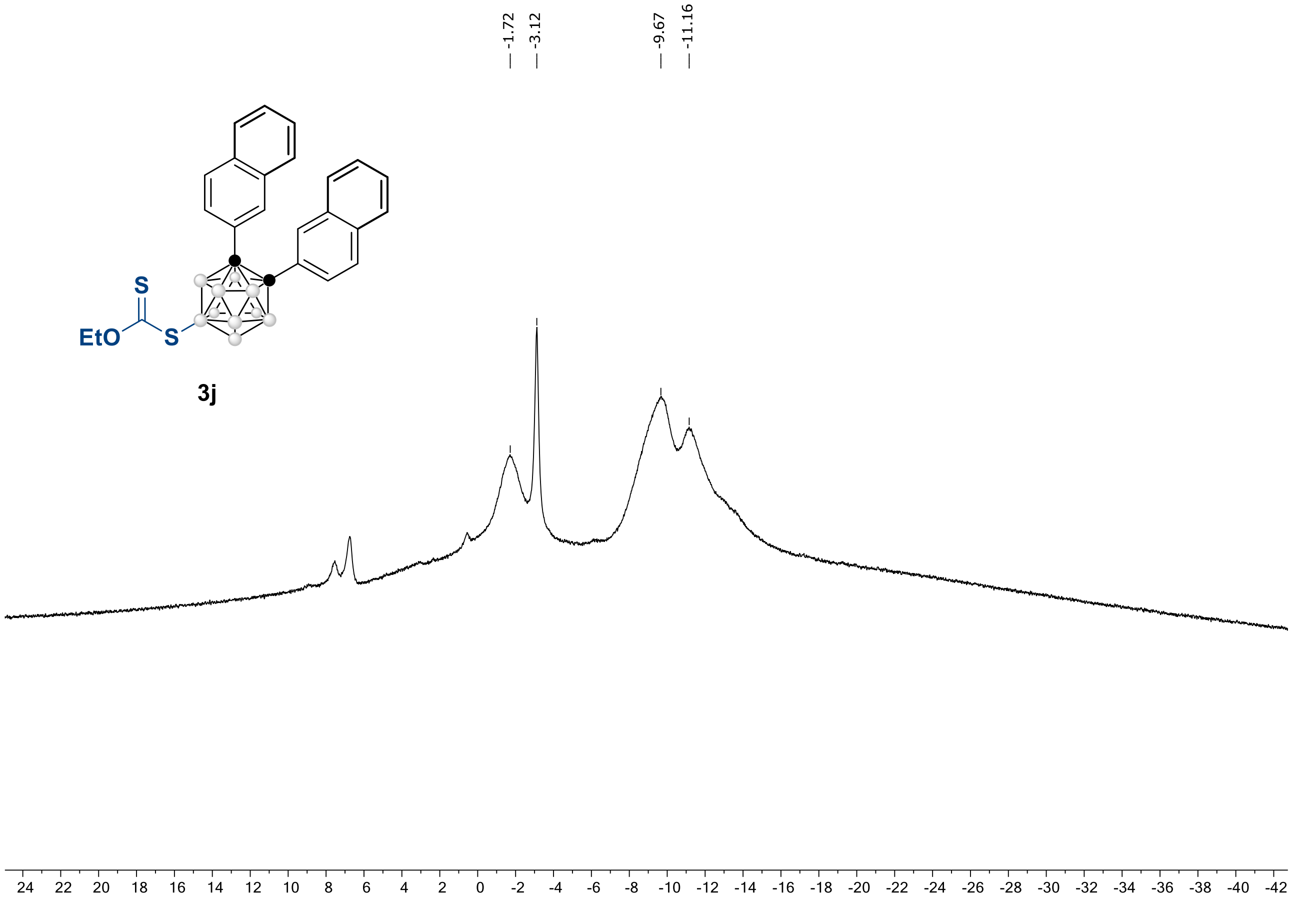

2D – COSY (400 MHz, $CDCl_3$)

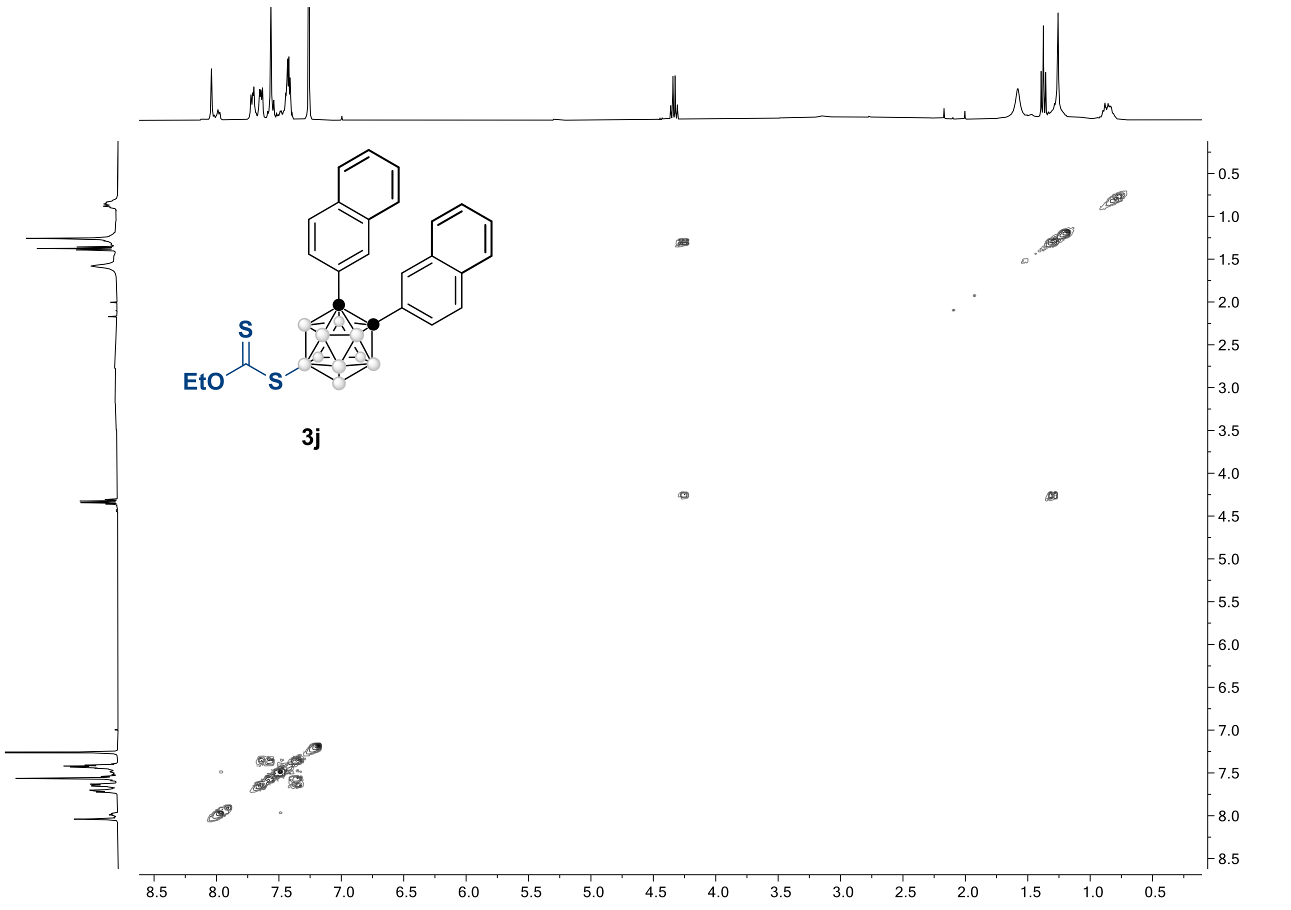

^{1}H-NMR (400 MHz, $CDCl_3$)

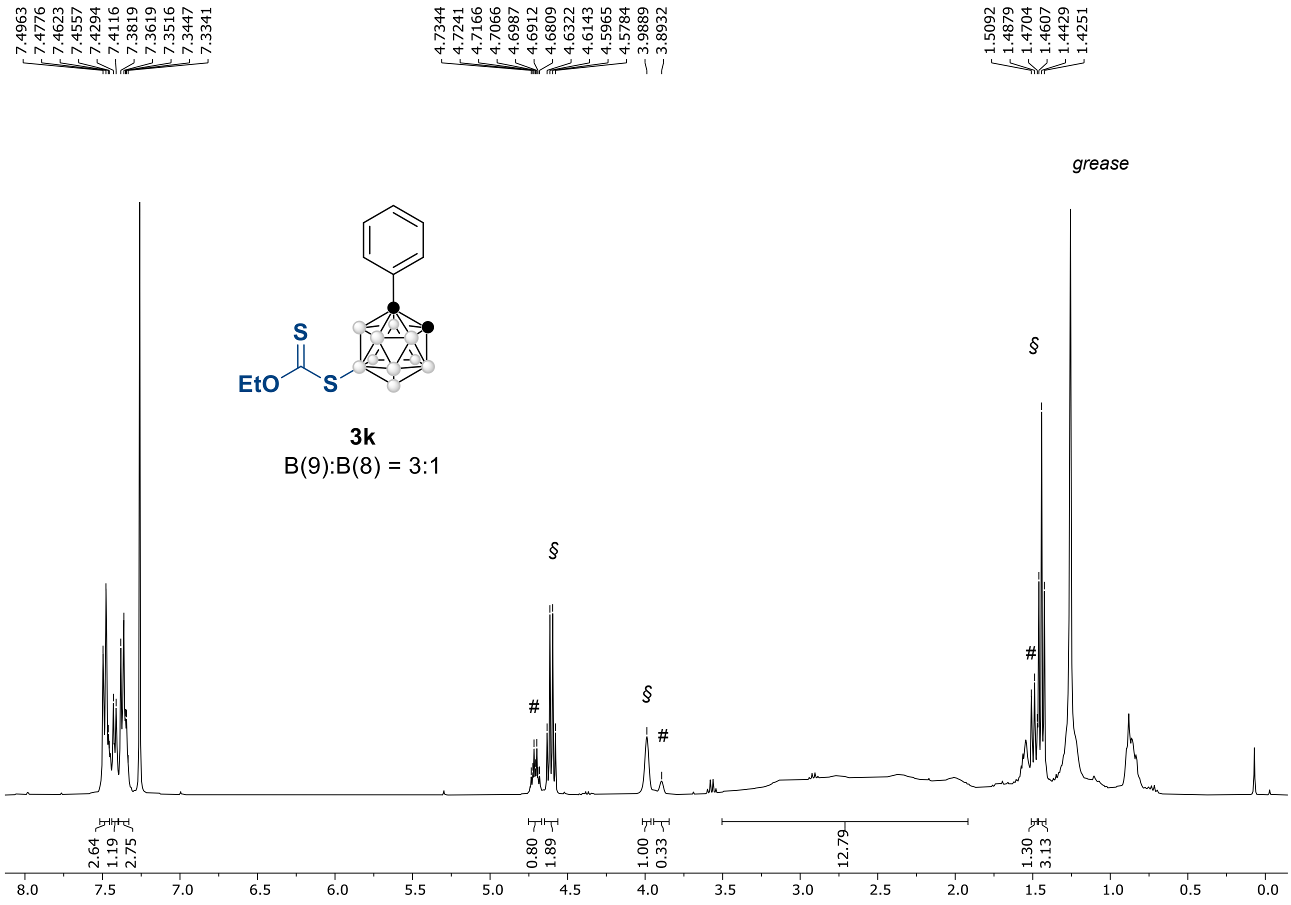

^{13}C{^{1}H}-NMR (100 MHz, $CDCl_3$)

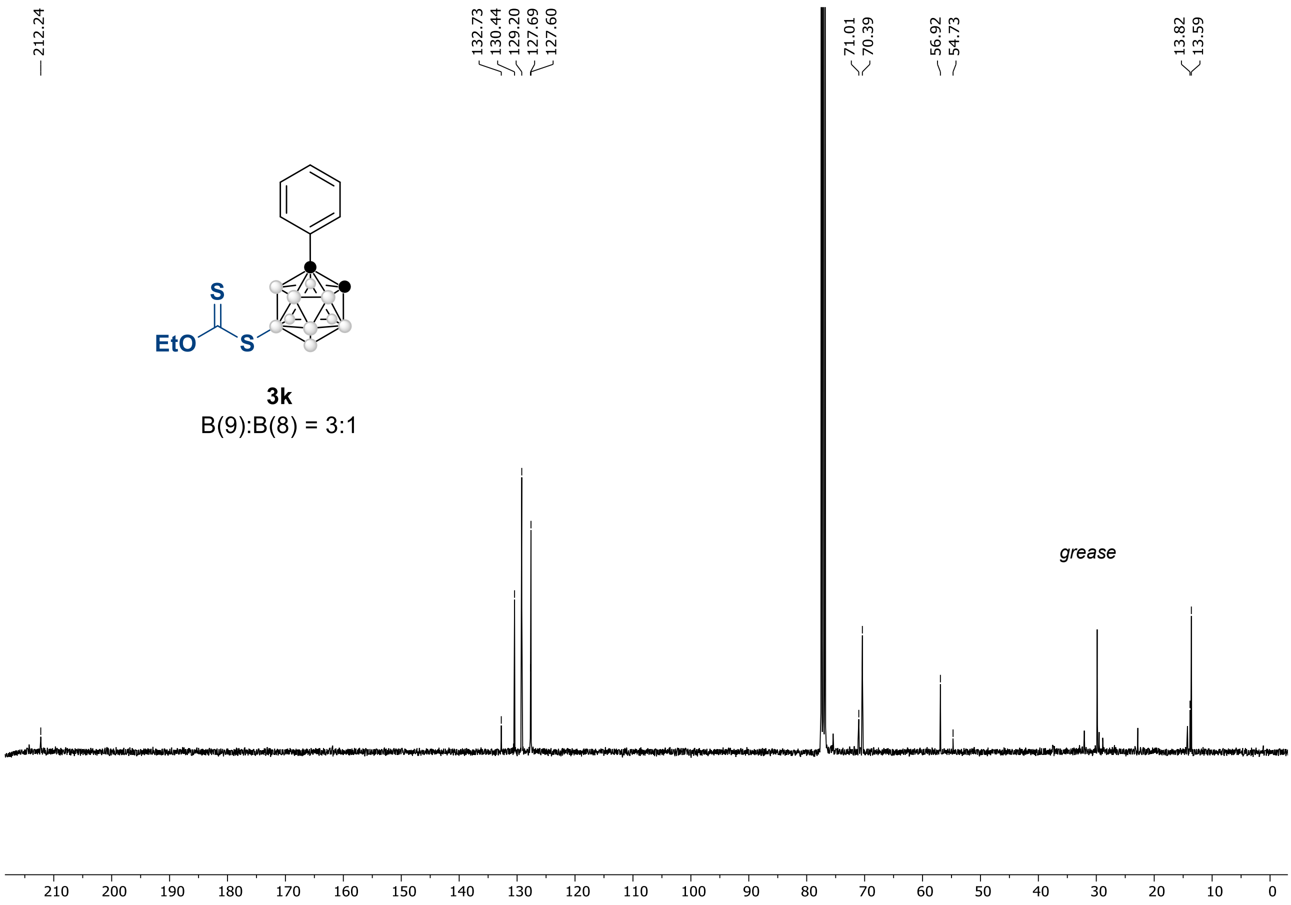

DEPT-135 (100 MHz, $CDCl_3$)

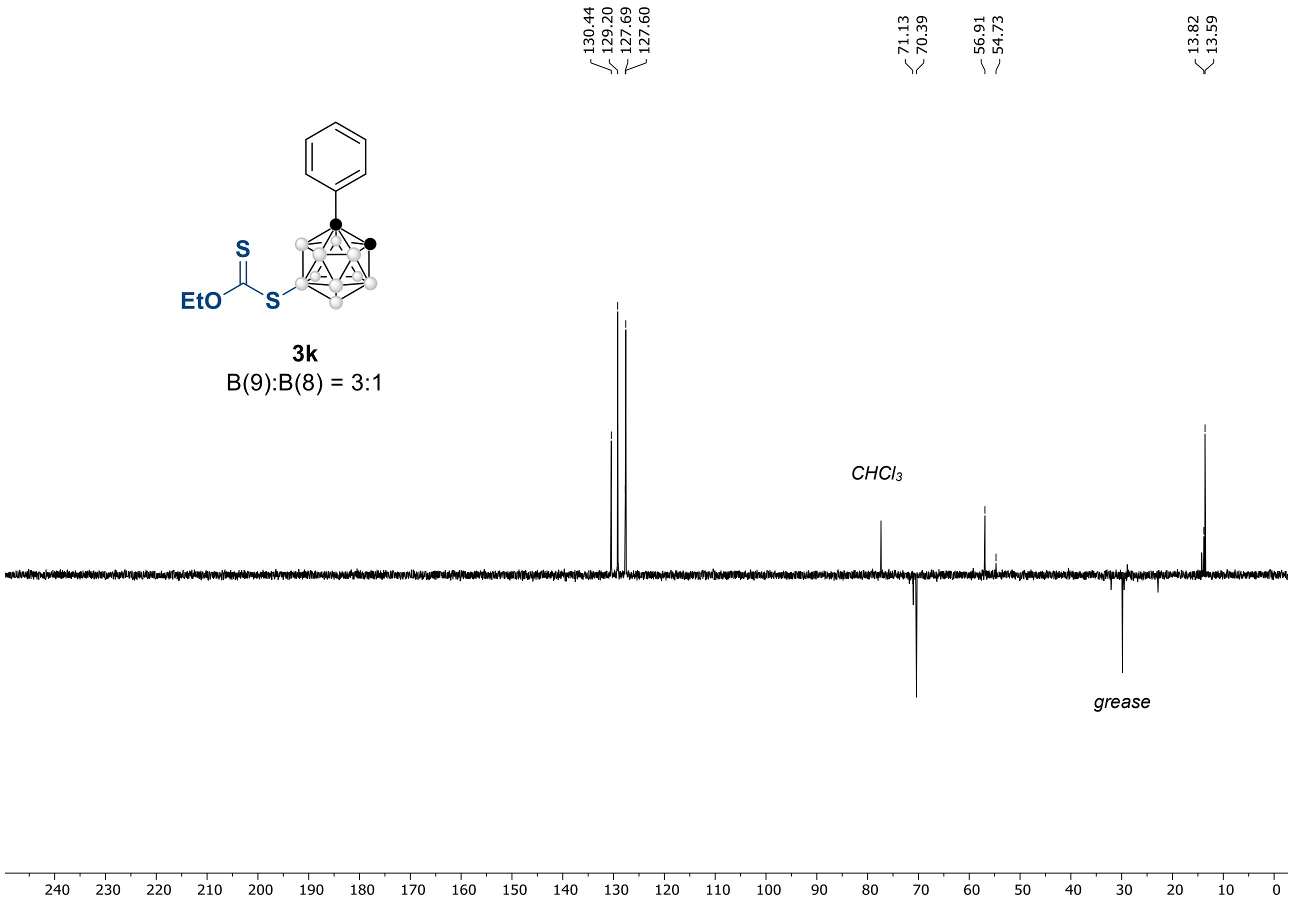

^{11}B{^{1}H}-NMR (128 MHz, $CDCl_3$)

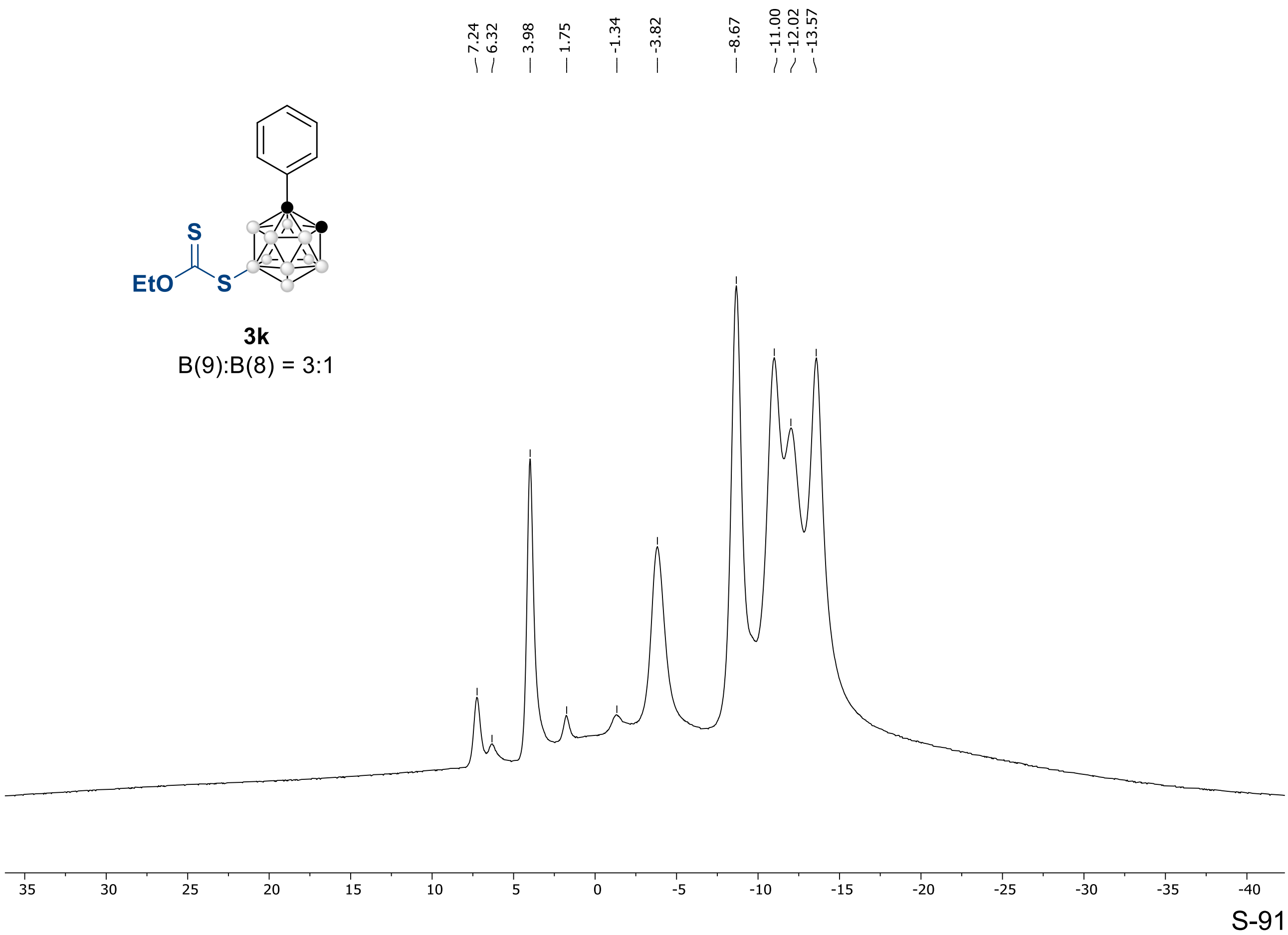

2D – COSY (400 MHz, $CDCl_3$)

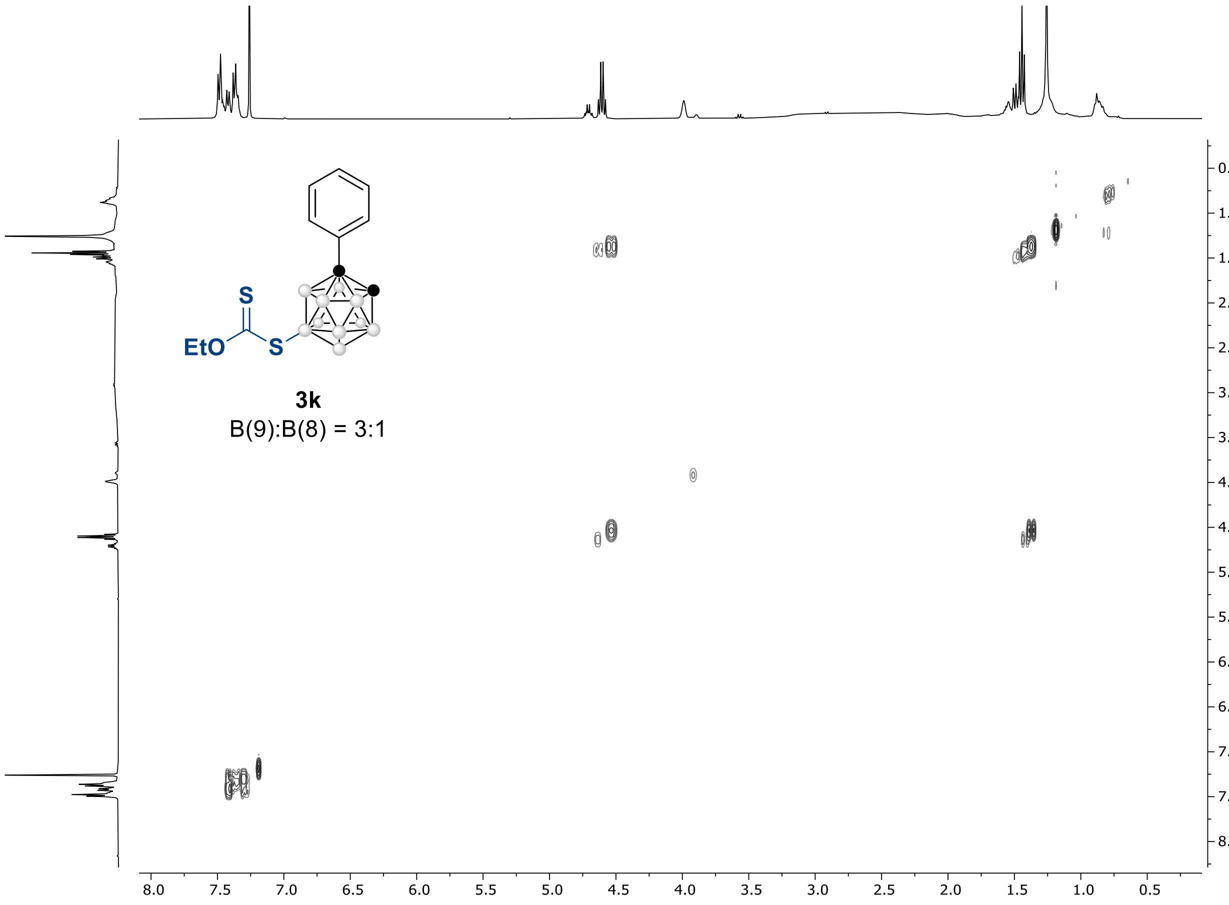

^{1}H-NMR (400 MHz, $CDCl_3$)

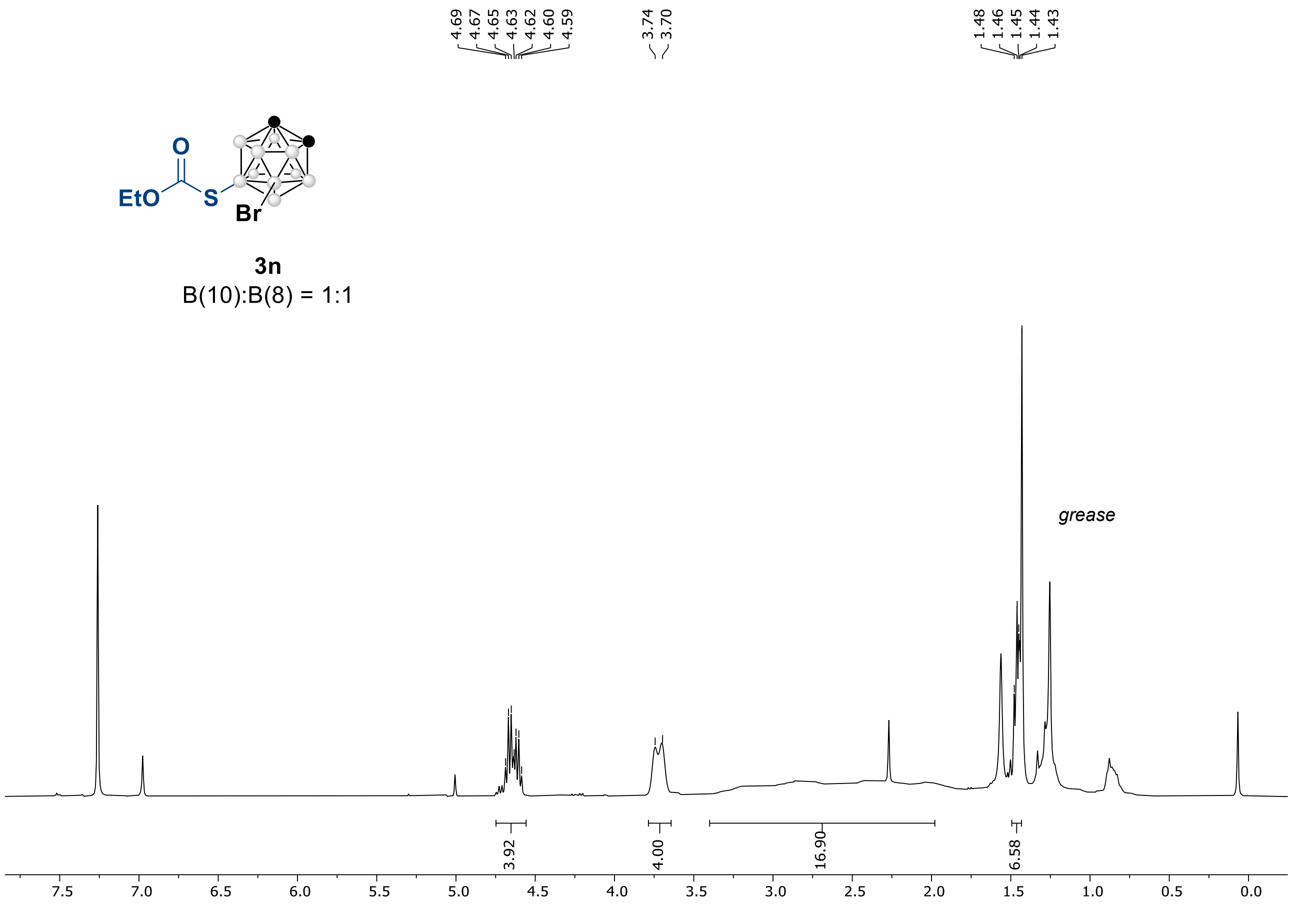

$^{13}C\{^1H\}$-NMR (100 MHz, $CDCl_3$)

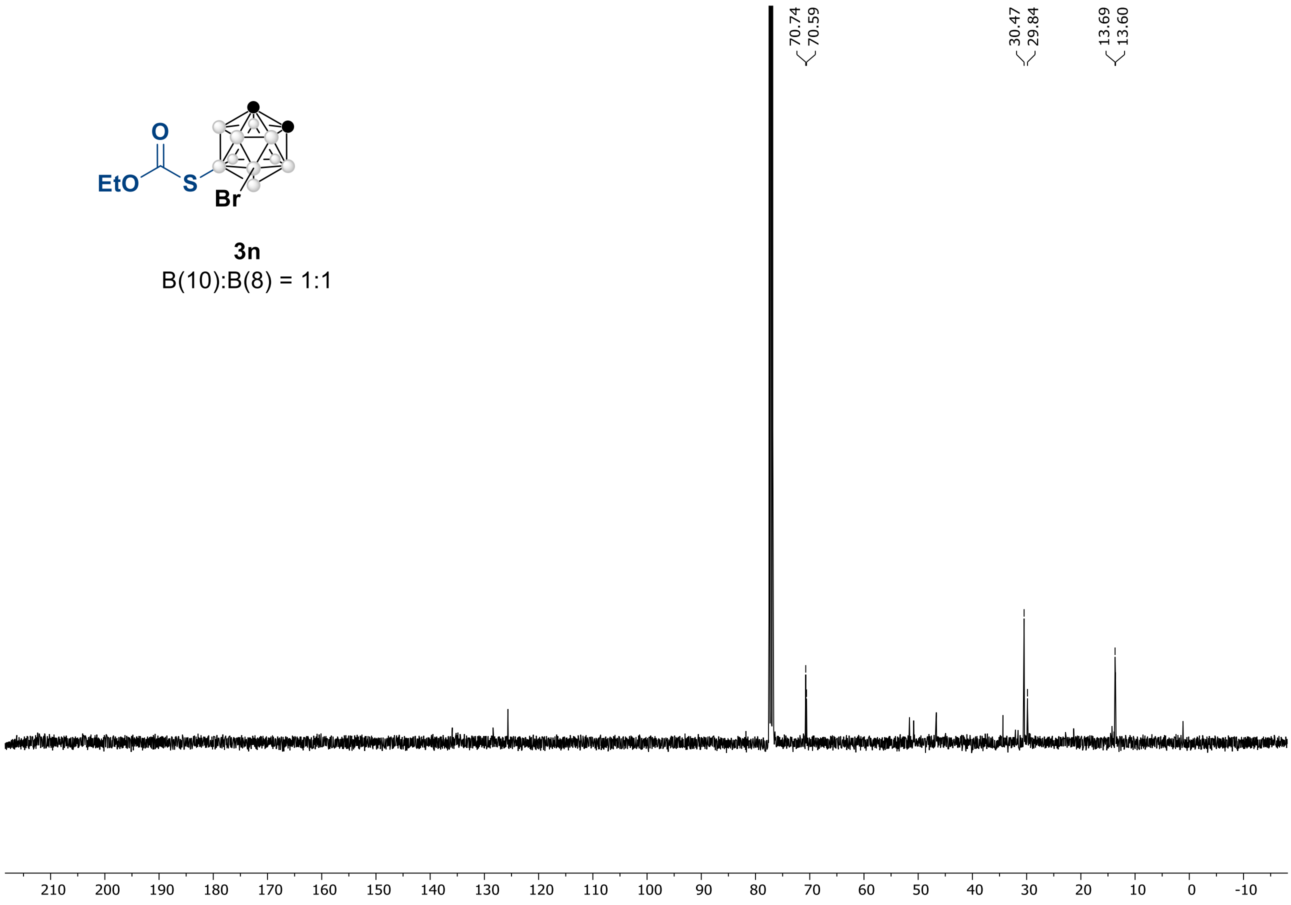

3n

B(10):B(8) = 1:1

DEPT135 (100 MHz, $CDCl_3$)

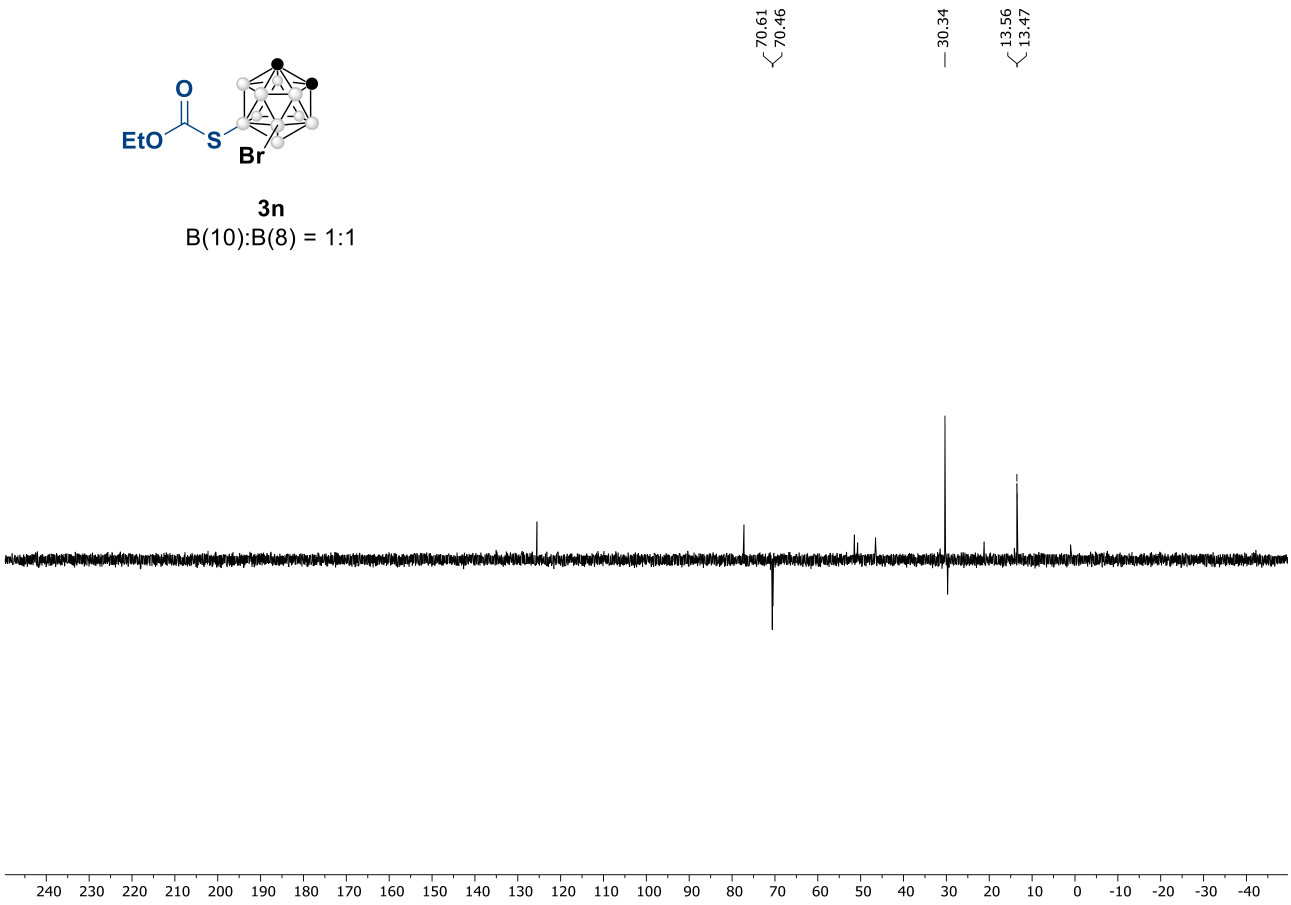

3n

B(10):B(8) = 1:1

^{11}B{^{1}H}-NMR (128 MHz, CDCl$_3$)

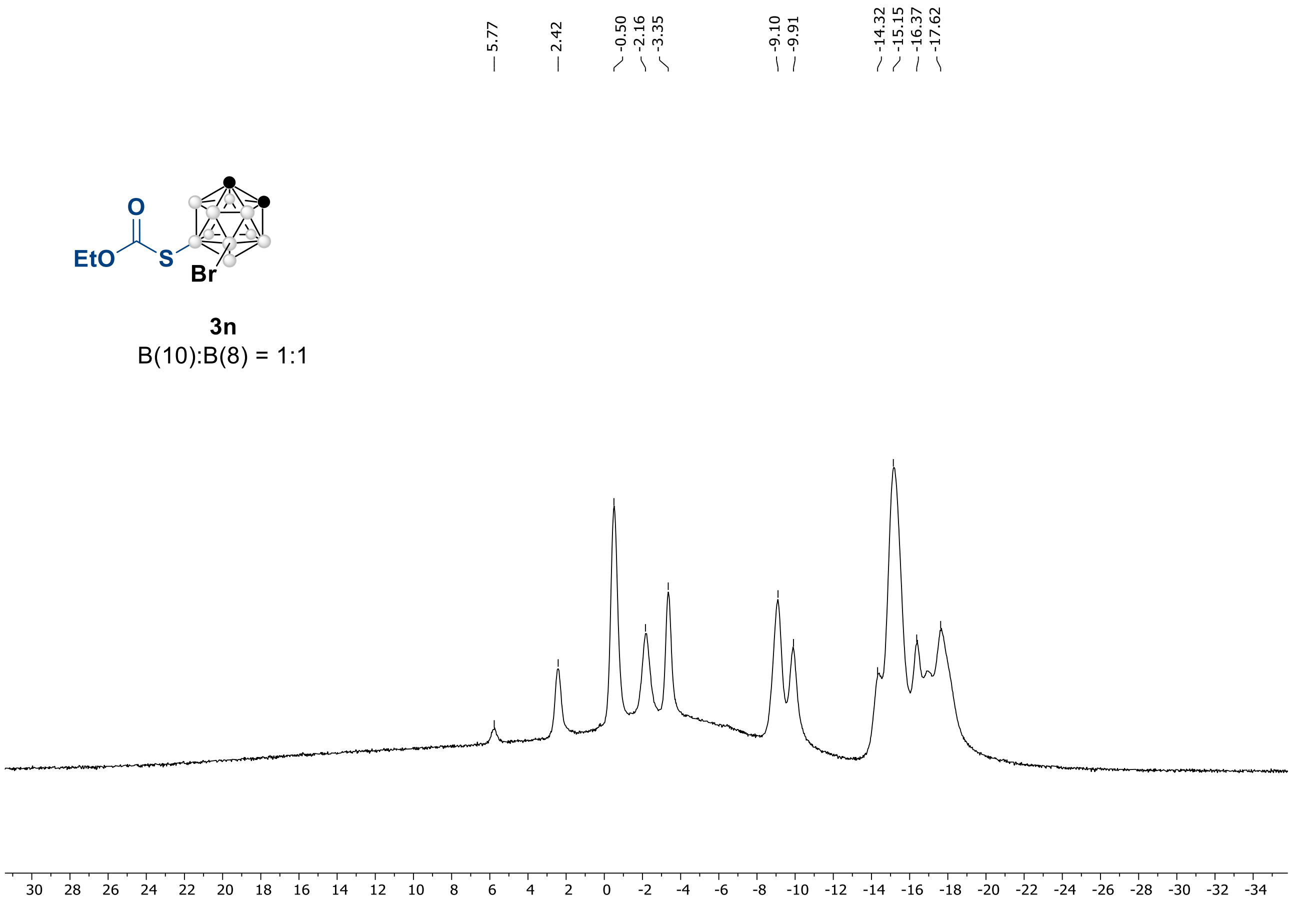

2D – COSY (100 MHz, CDCl$_3$)

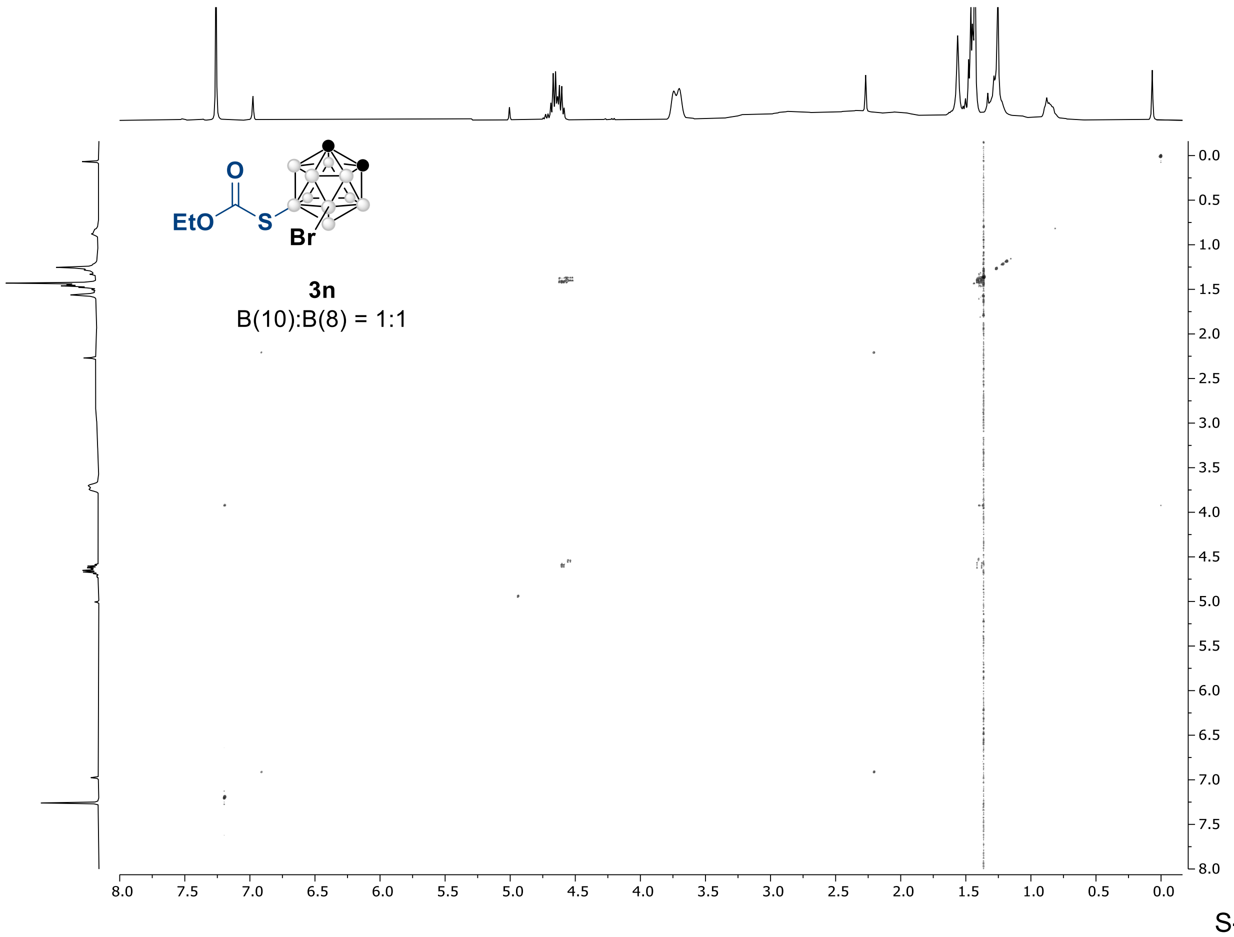

^{1}H-NMR (400 MHz, $CDCl_3$)

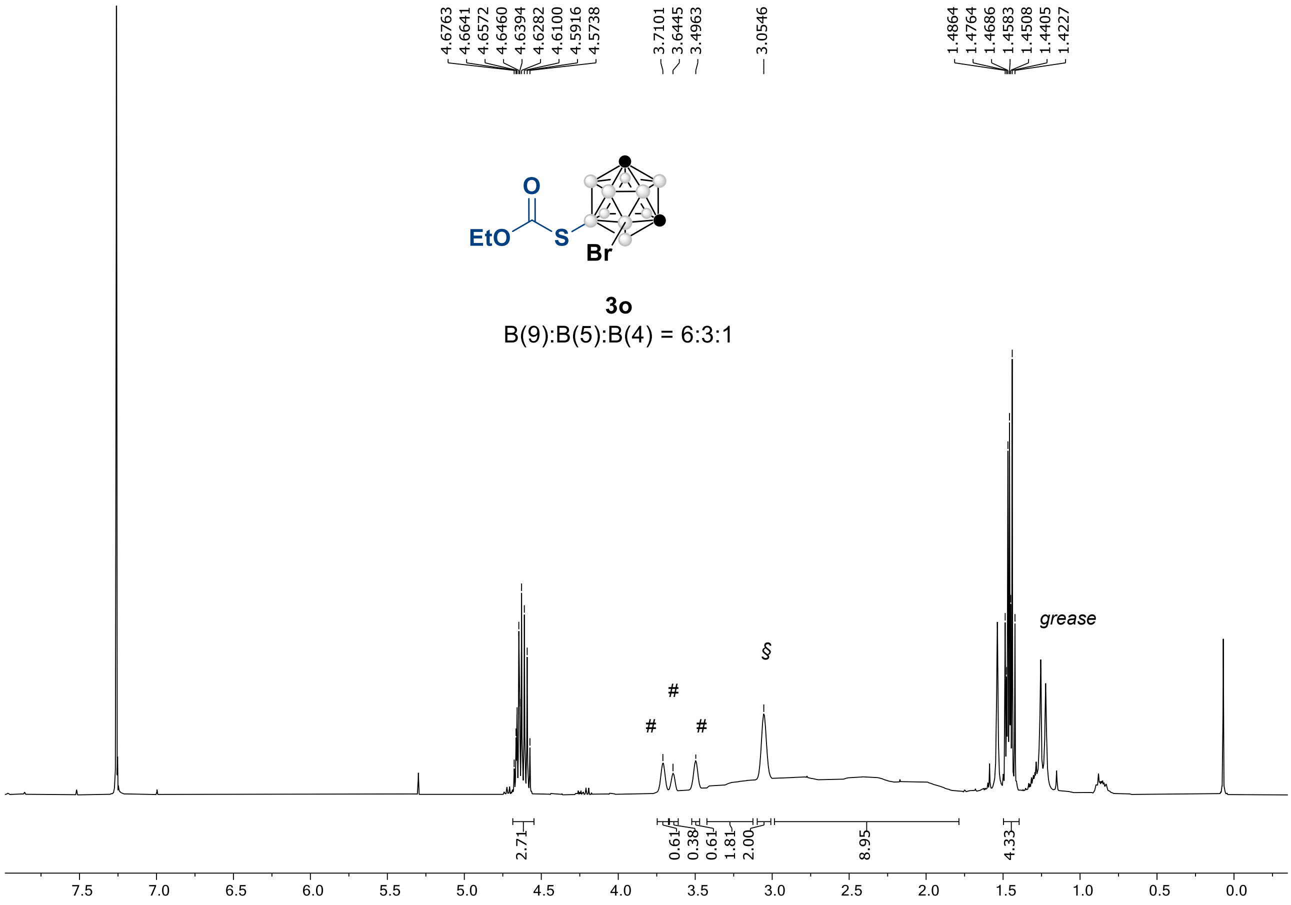

^{13}C{^{1}H}-NMR (100 MHz, $CDCl_3$)

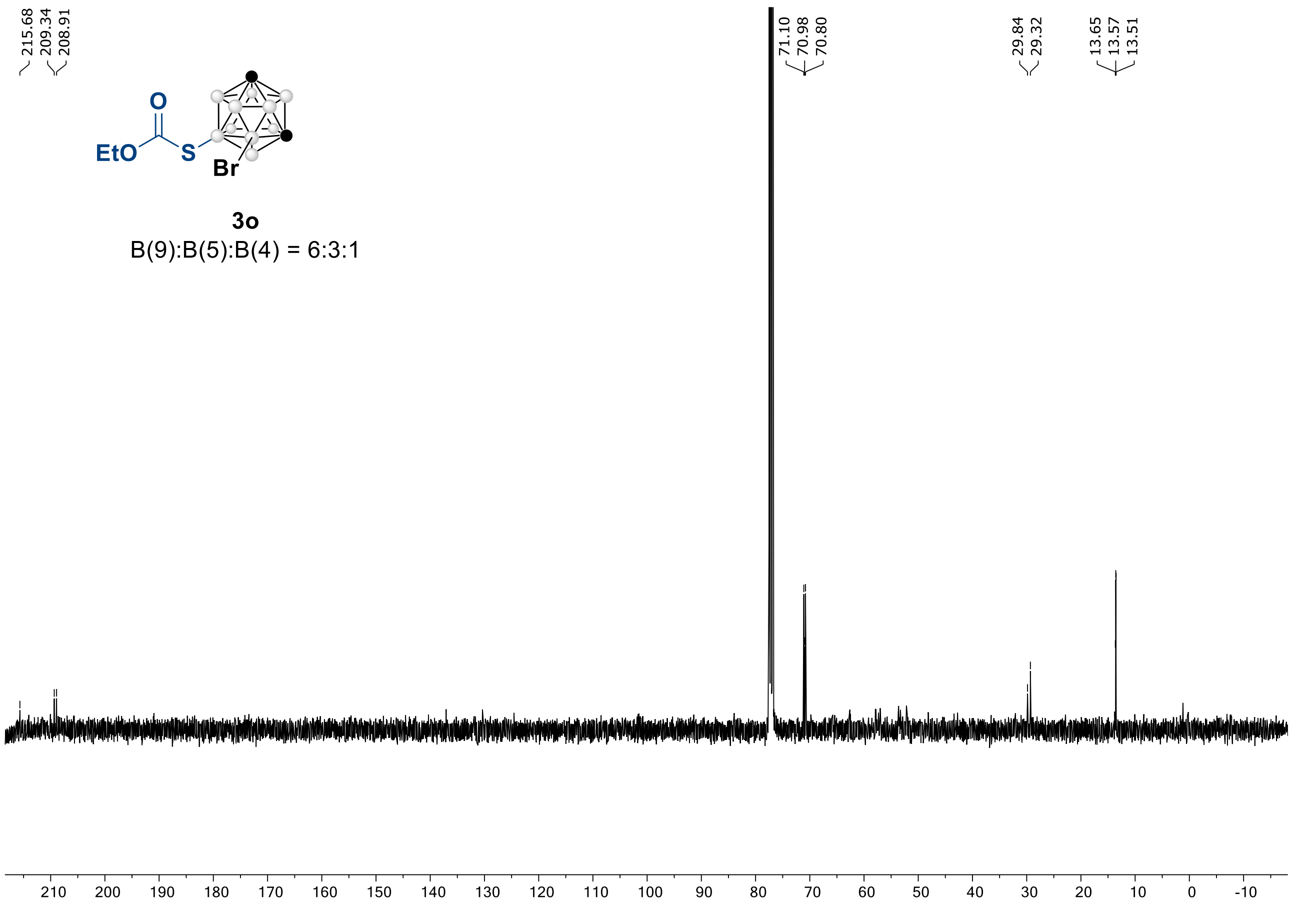

DEPT135 (100 MHz, $CDCl_3$)

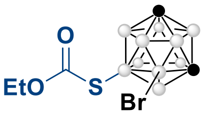

3o
B(9):B(5):B(4) = 6:3:1

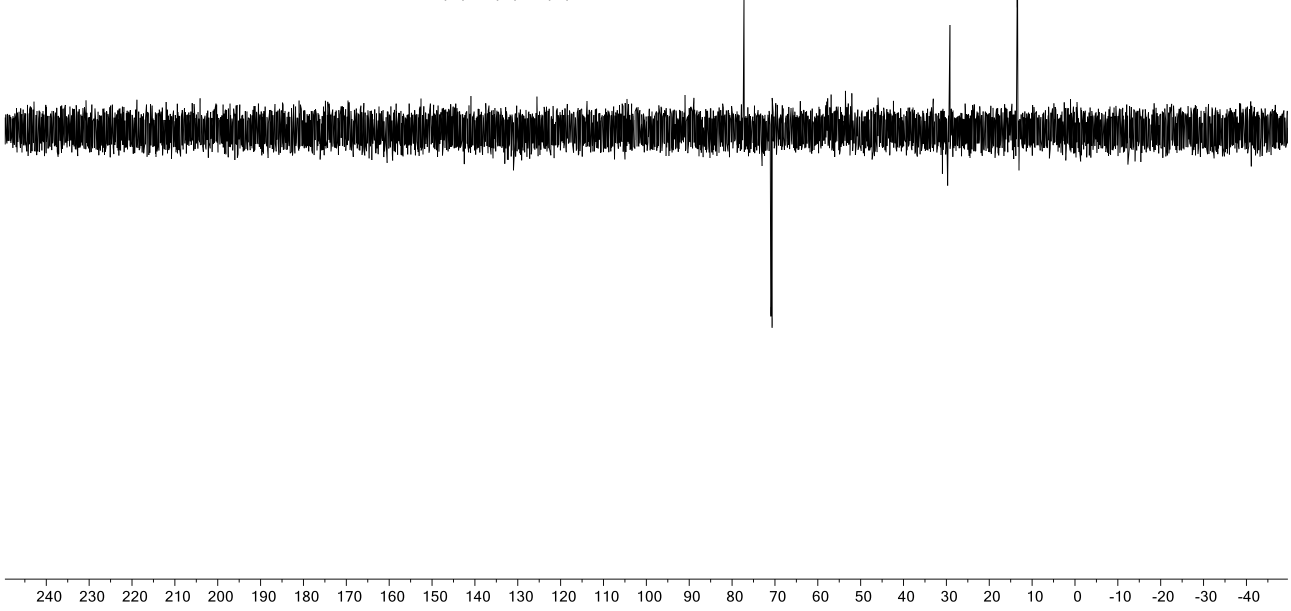

$^{11}B\{^1H\}$-NMR (128 MHz, $CDCl_3$)

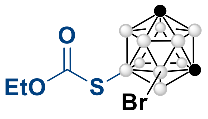

3o
B(9):B(5):B(4) = 6:3:1

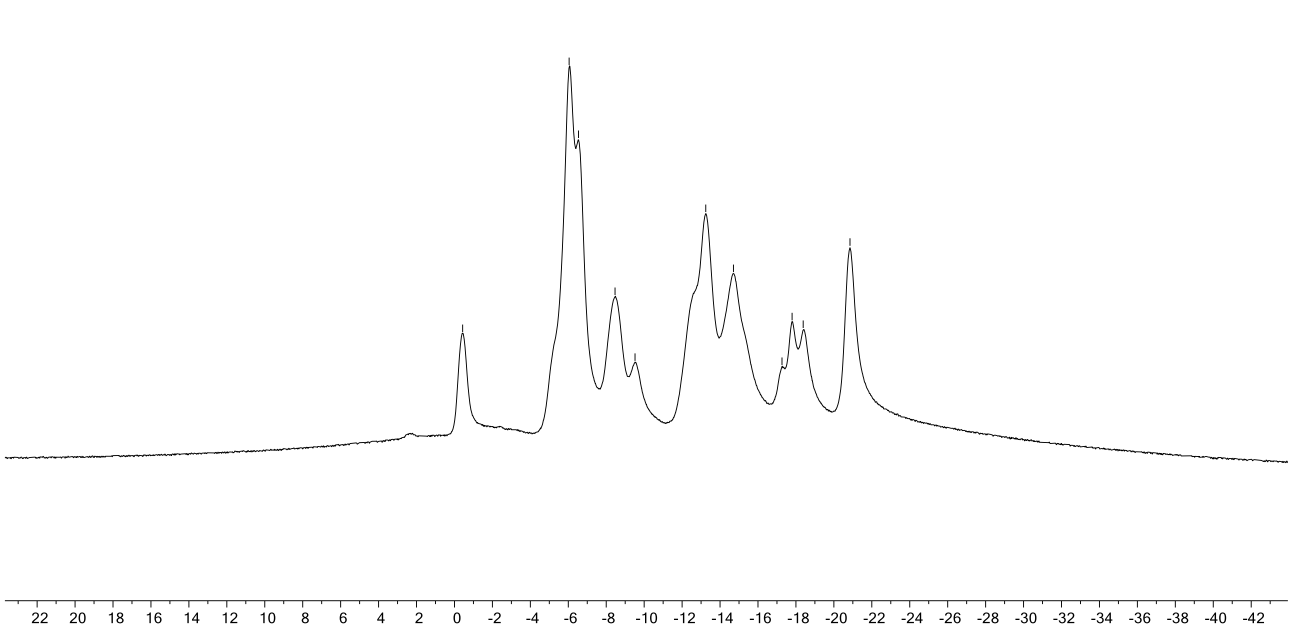

2D – COSY (100 MHz, $CDCl_3$)

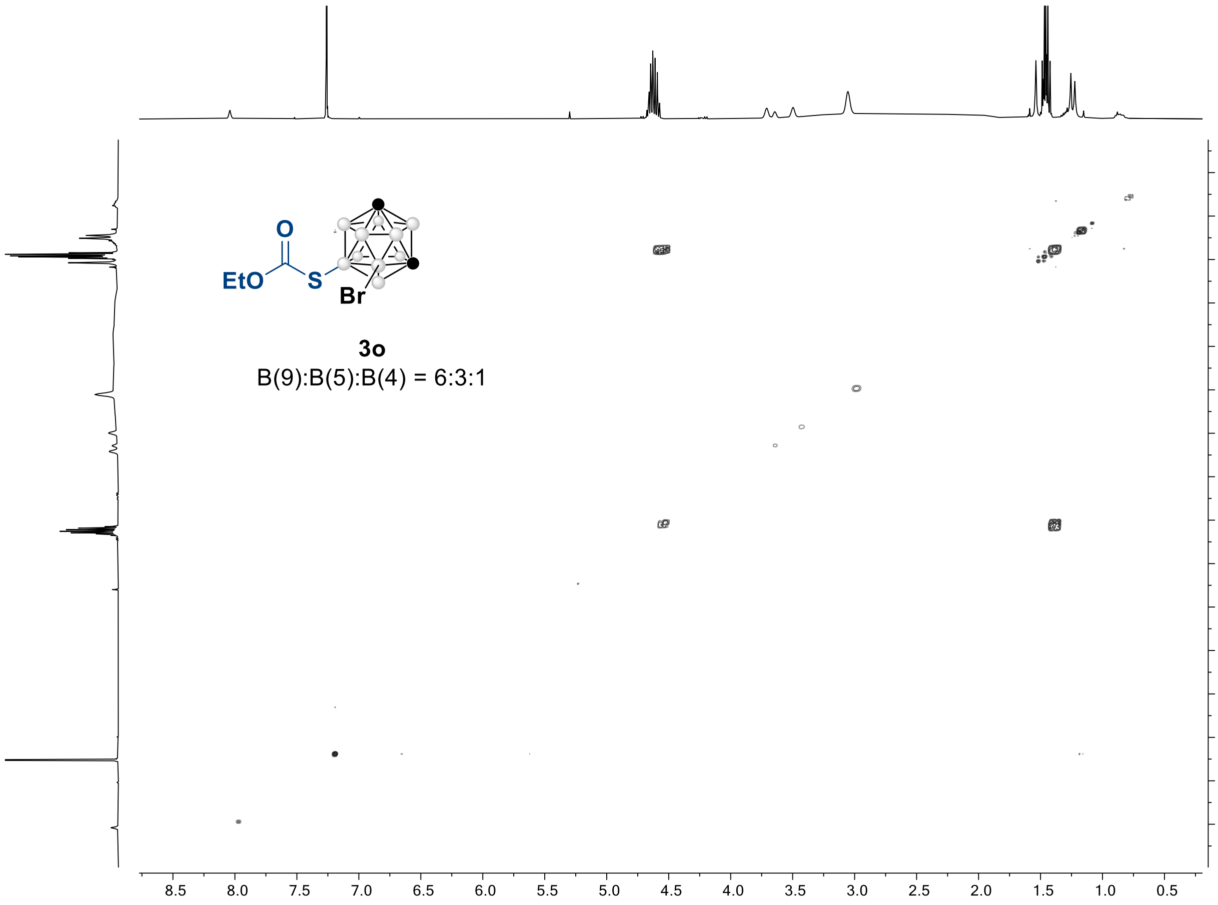

^{1}H-NMR (400 MHz, $CDCl_3$)

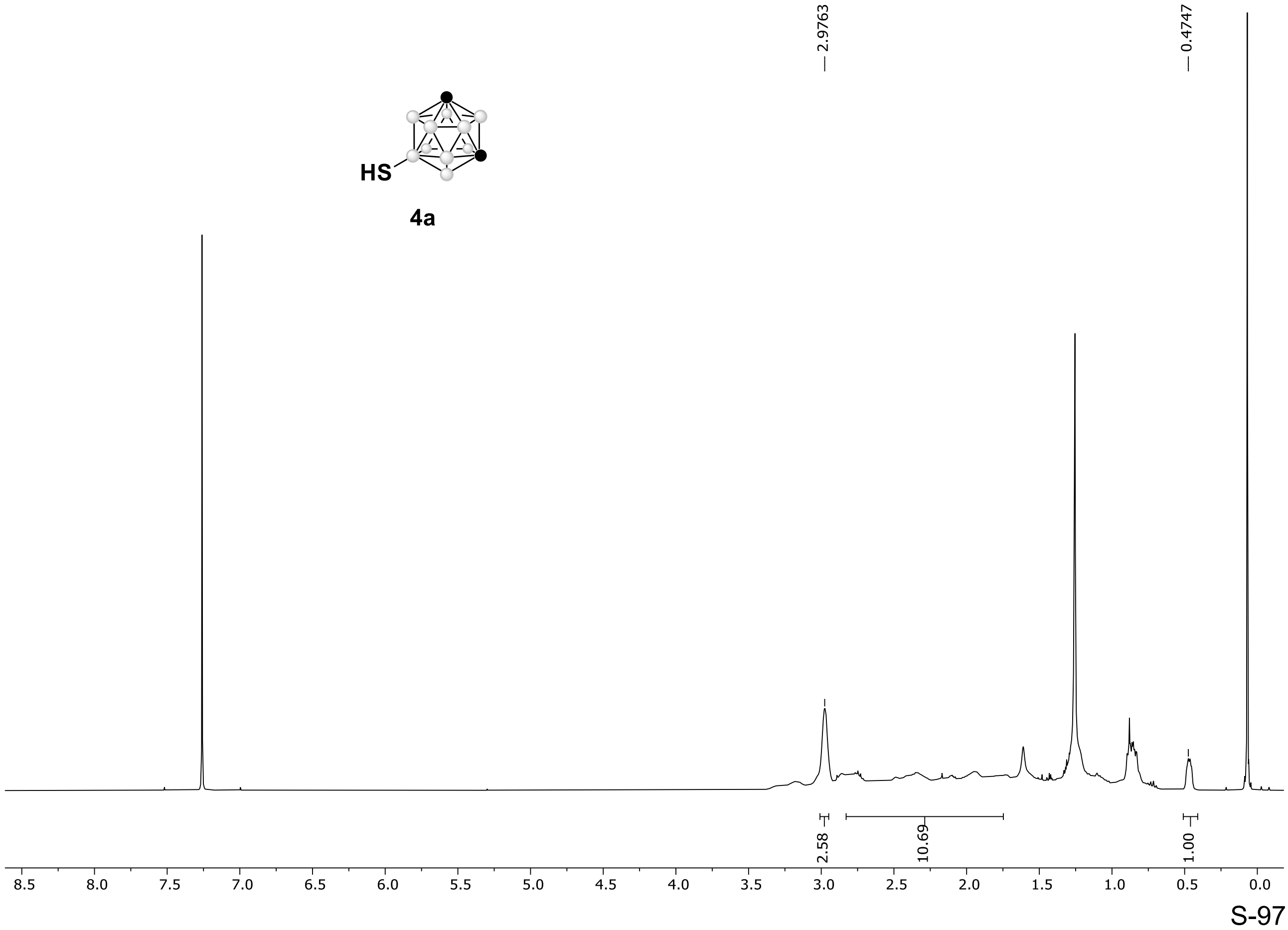

^{1}H-NMR (400 MHz, $CDCl_3$)

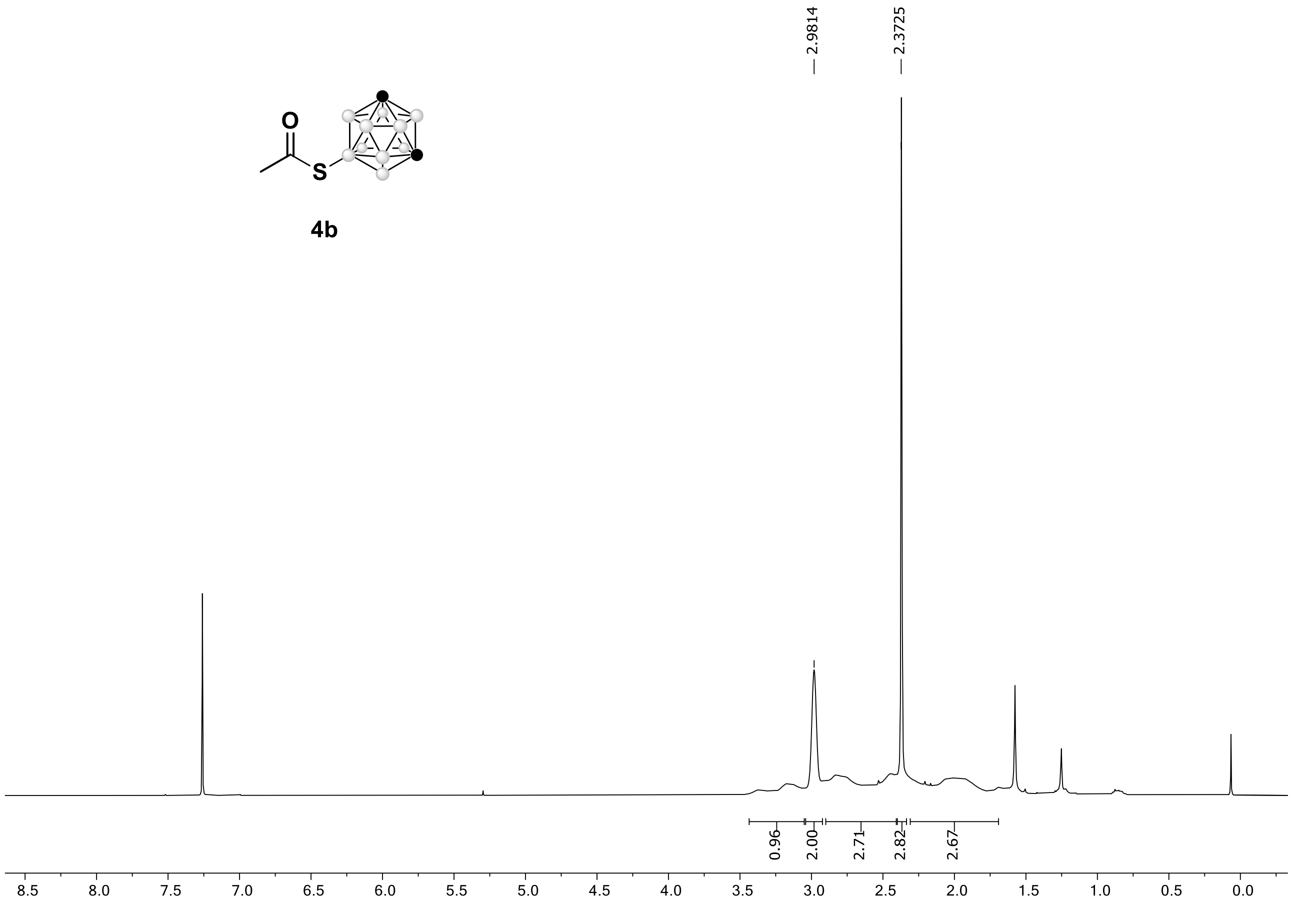

^{13}C{^{1}H}-NMR (100 MHz, $CDCl_3$)

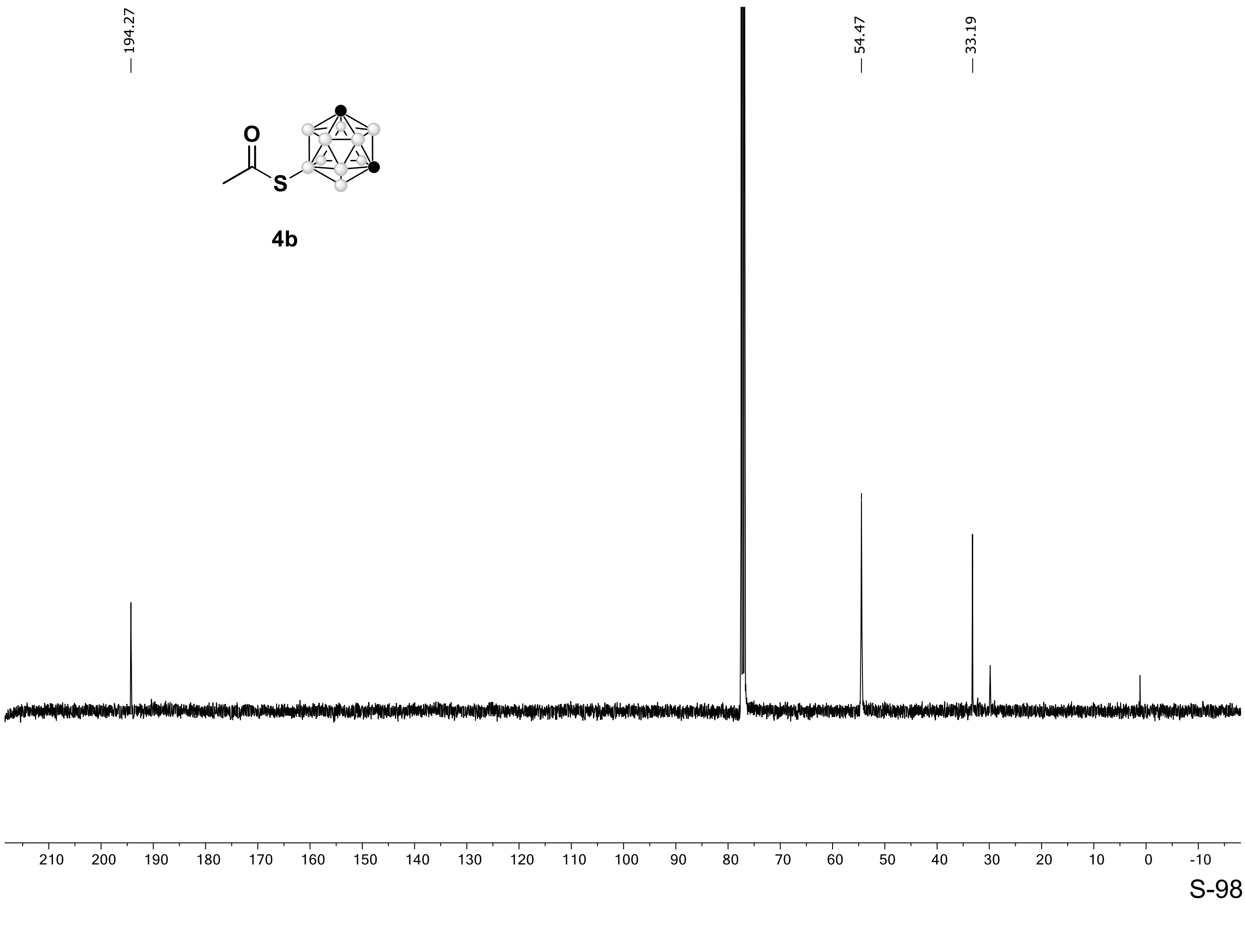

DEPT-135 (100 MHz, $CDCl_3$)

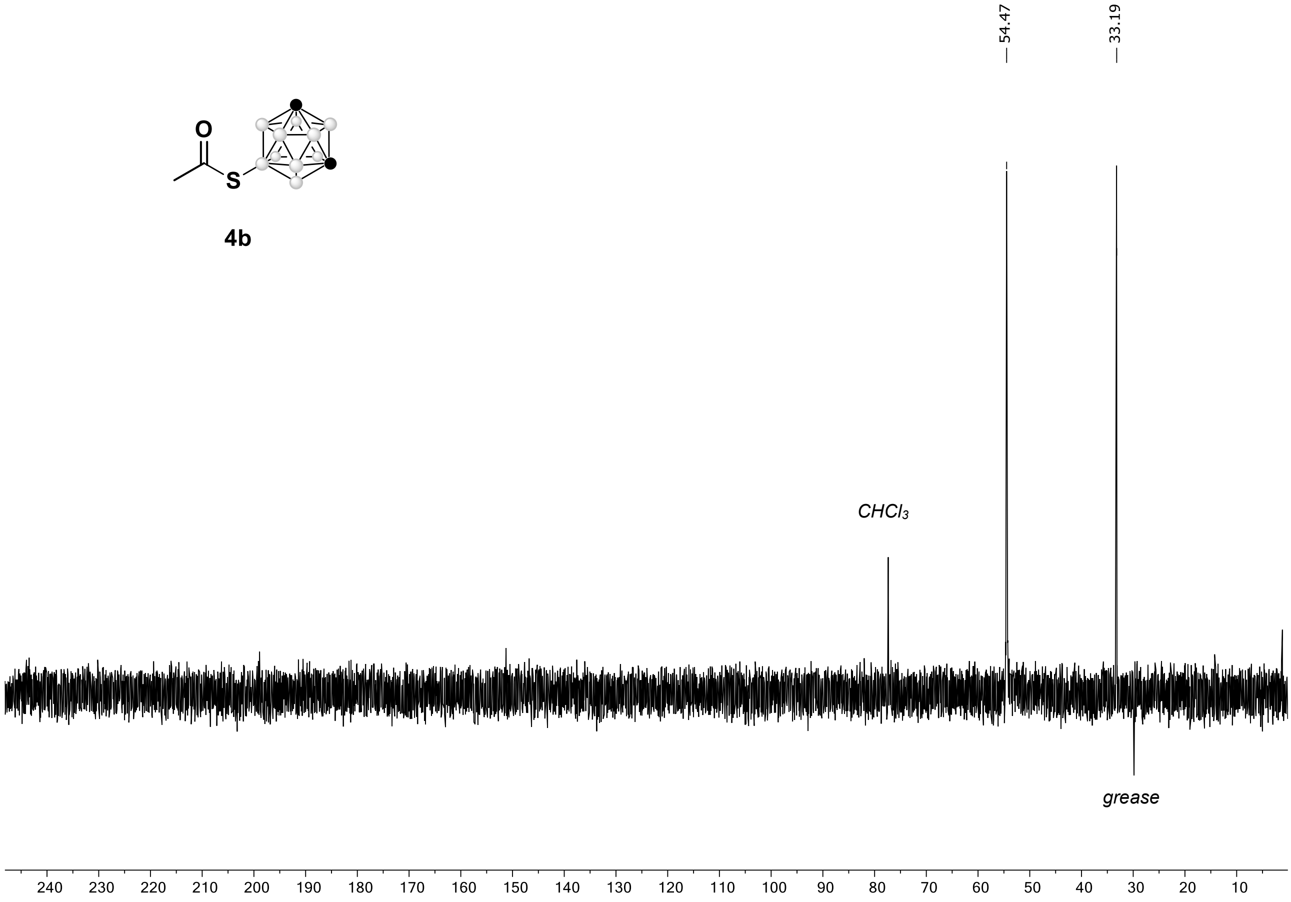

$^{11}B\{^1H\}$-NMR (128 MHz, $CDCl_3$)

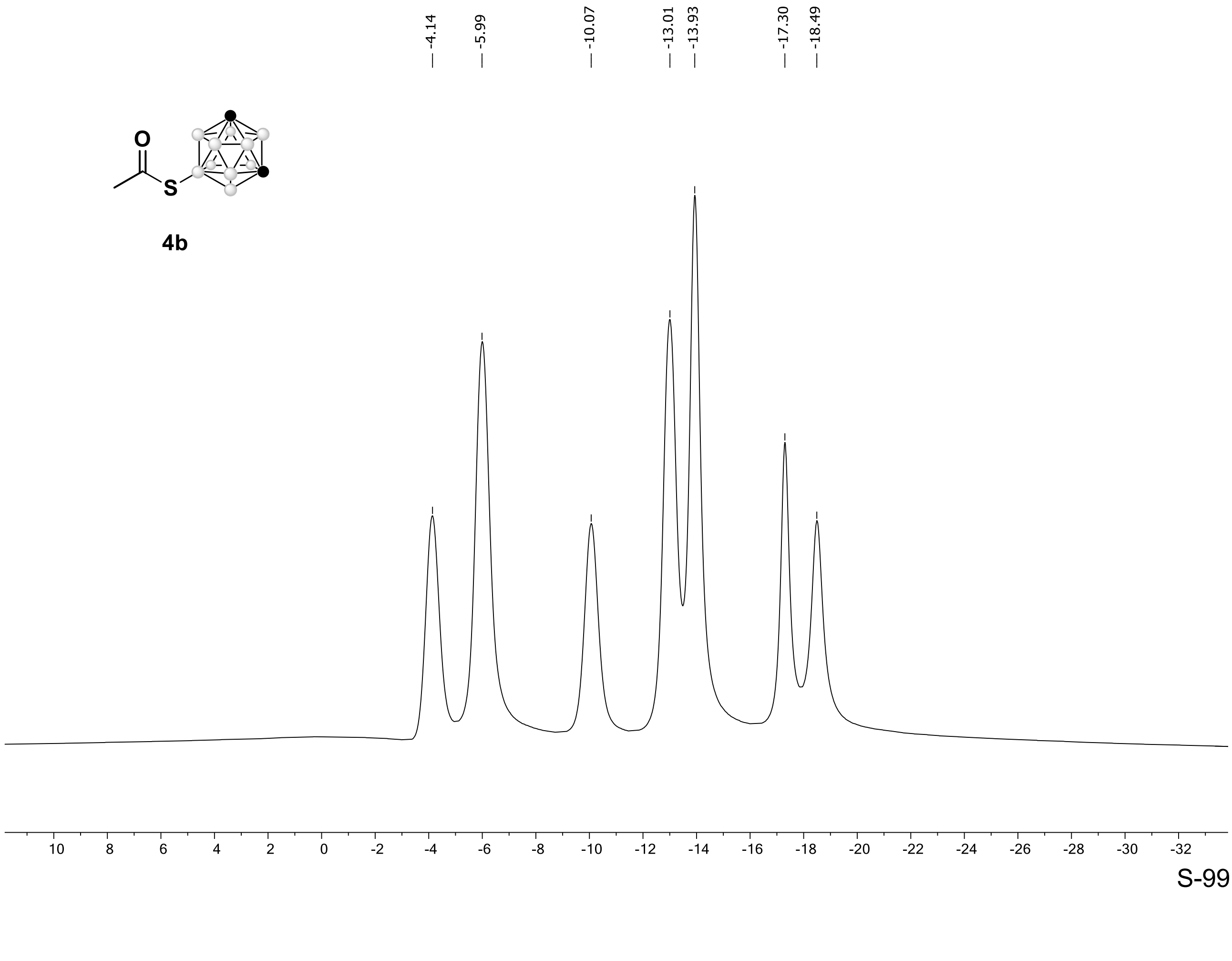

2D – COSY (400 MHz, $CDCl_3$)

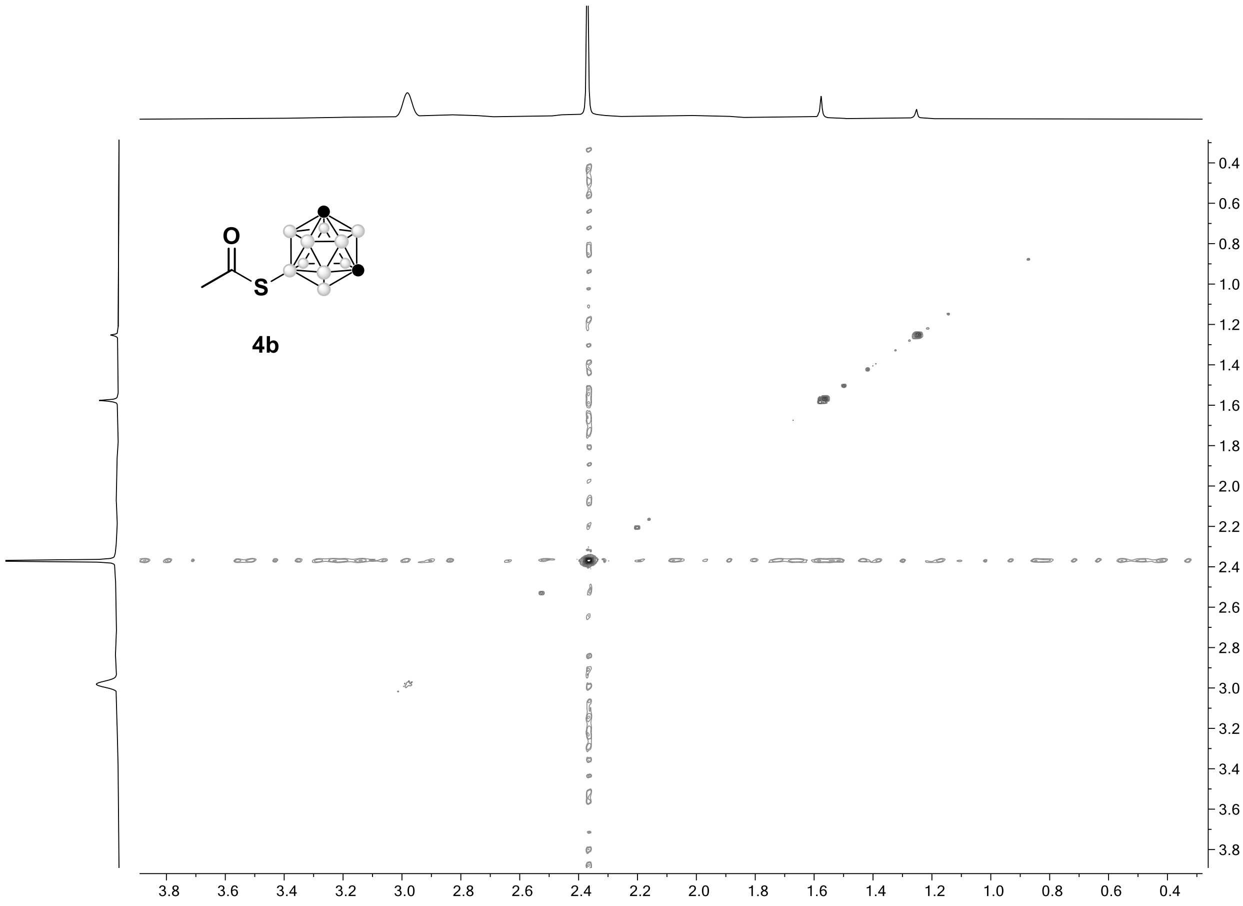

^{1}H-NMR (400 MHz, $CDCl_3$)

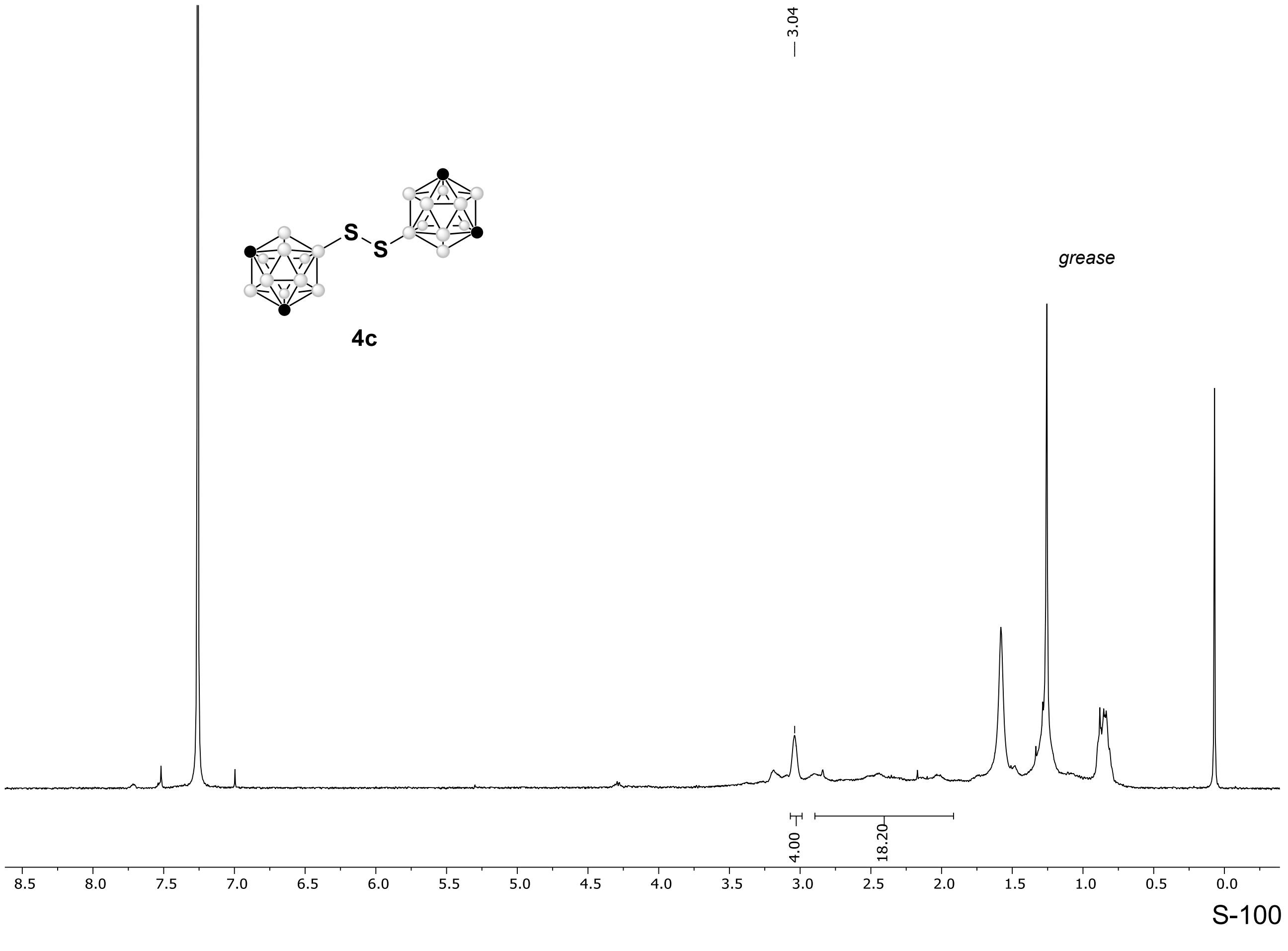

^{1}H-NMR (400 MHz, $CDCl_3$)

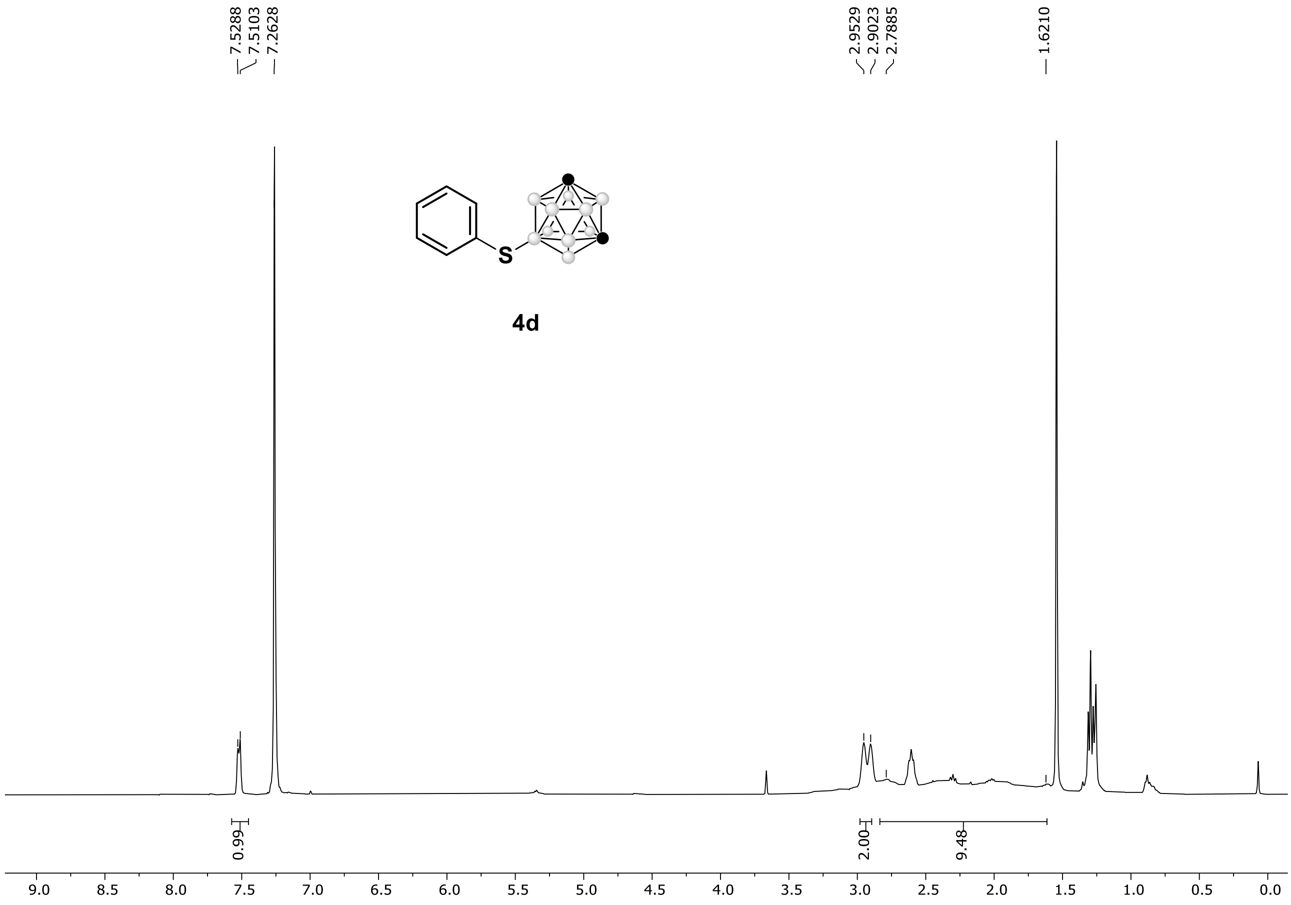

^{1}H-NMR (400 MHz, $CDCl_3$)

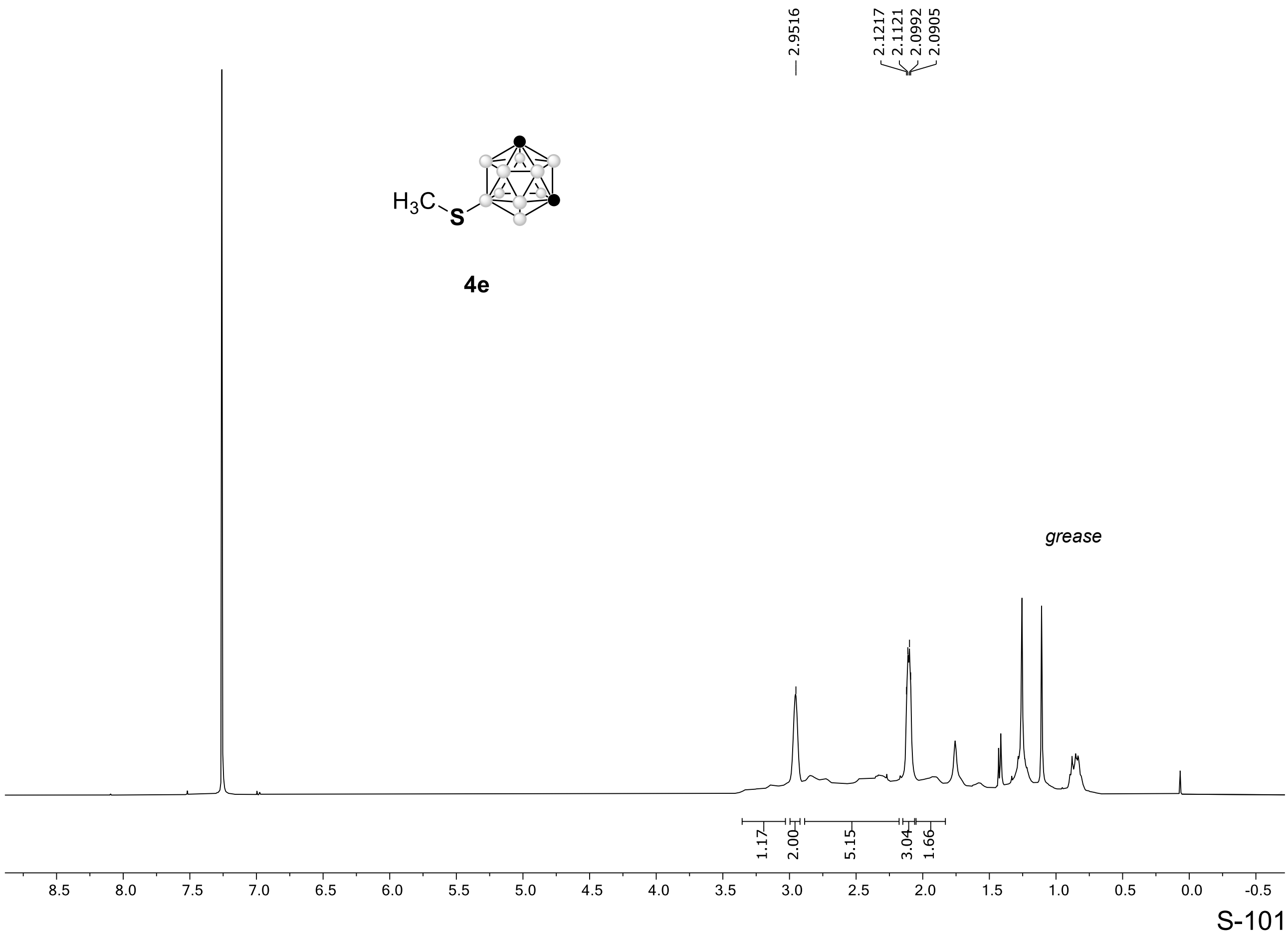

¹H-NMR (400 MHz, $CDCl_3$)

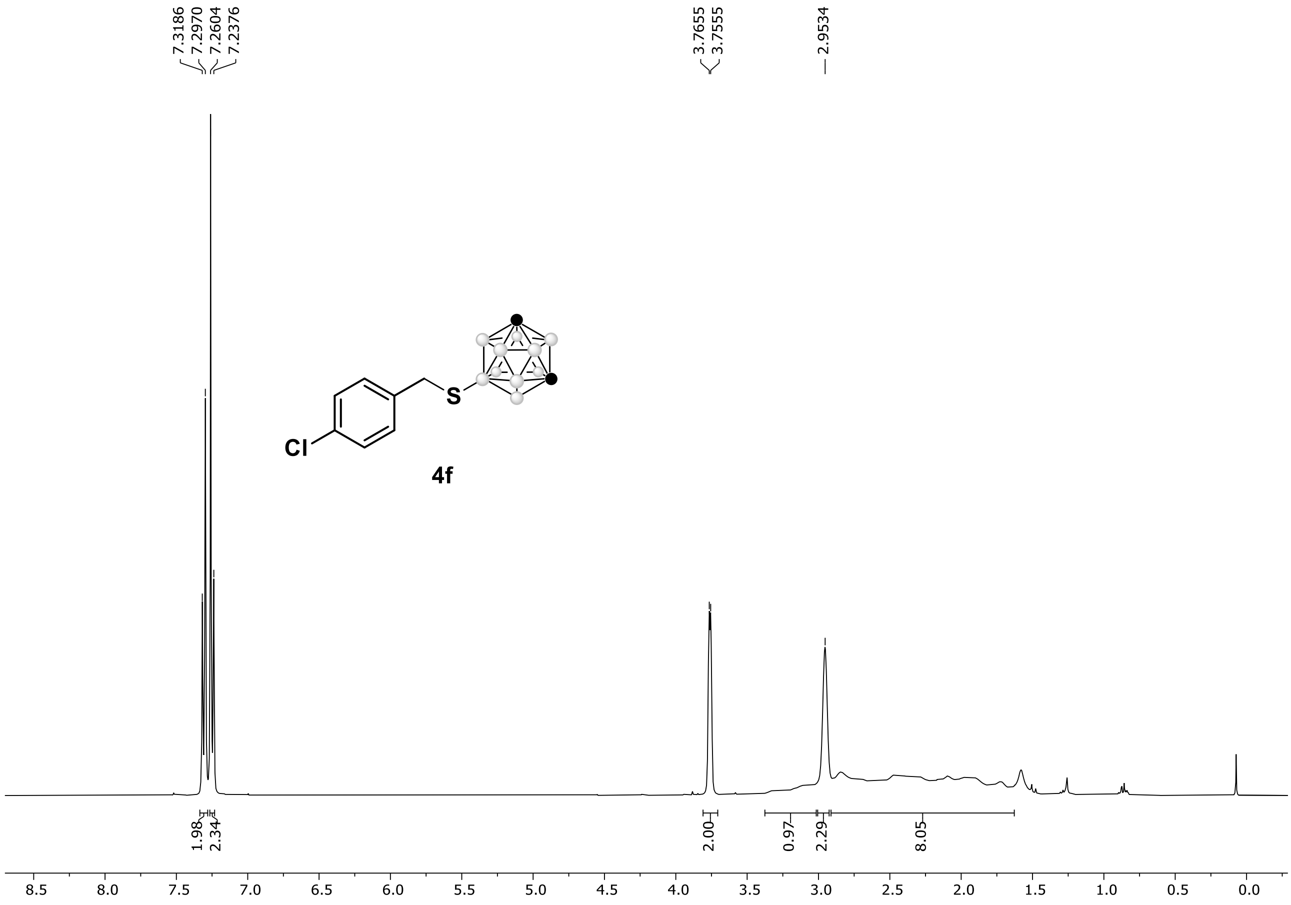

$^{13}C\{^1H\}$-NMR (100 MHz, $CDCl_3$)

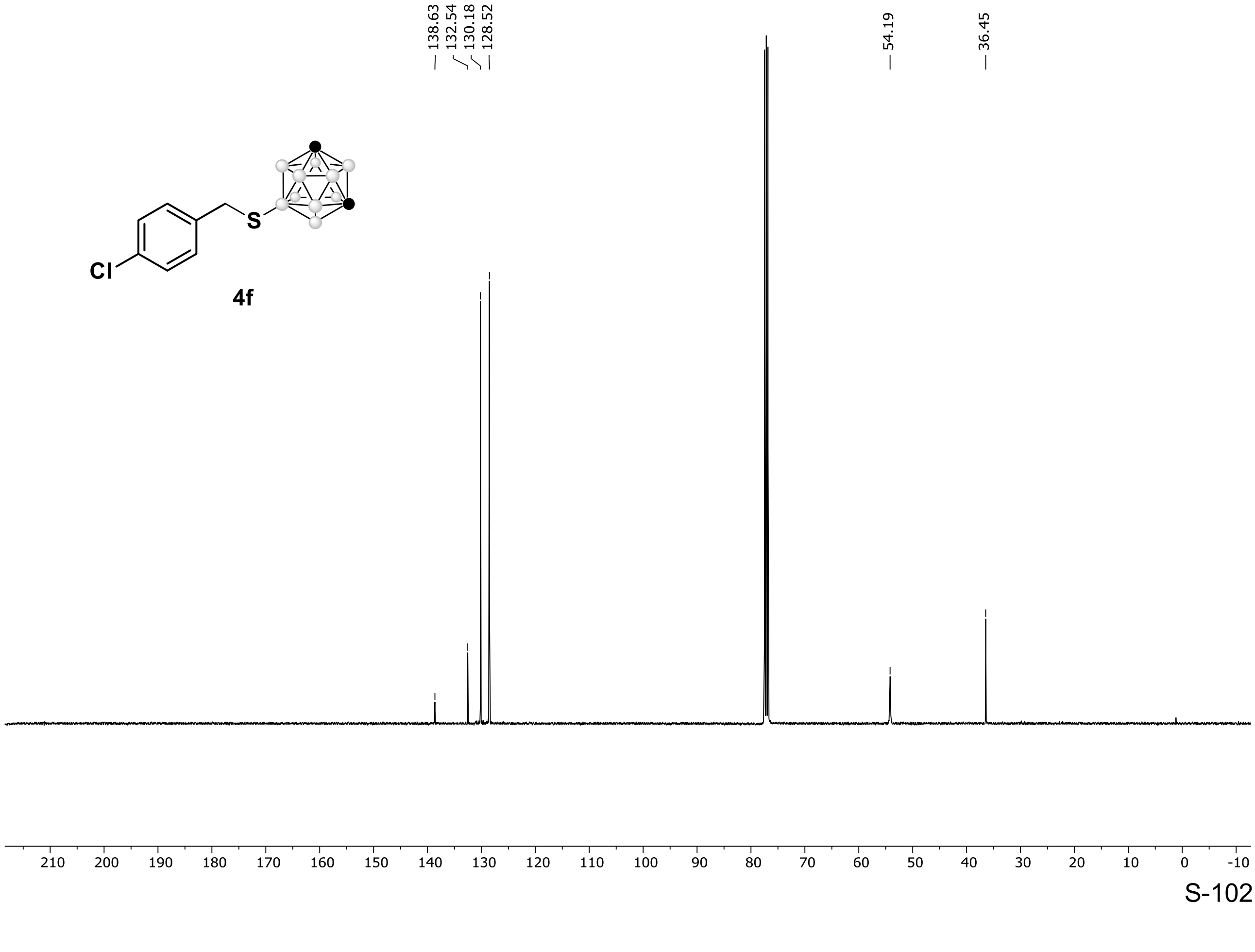

DEPT-135 (100 MHz, $CDCl_3$)

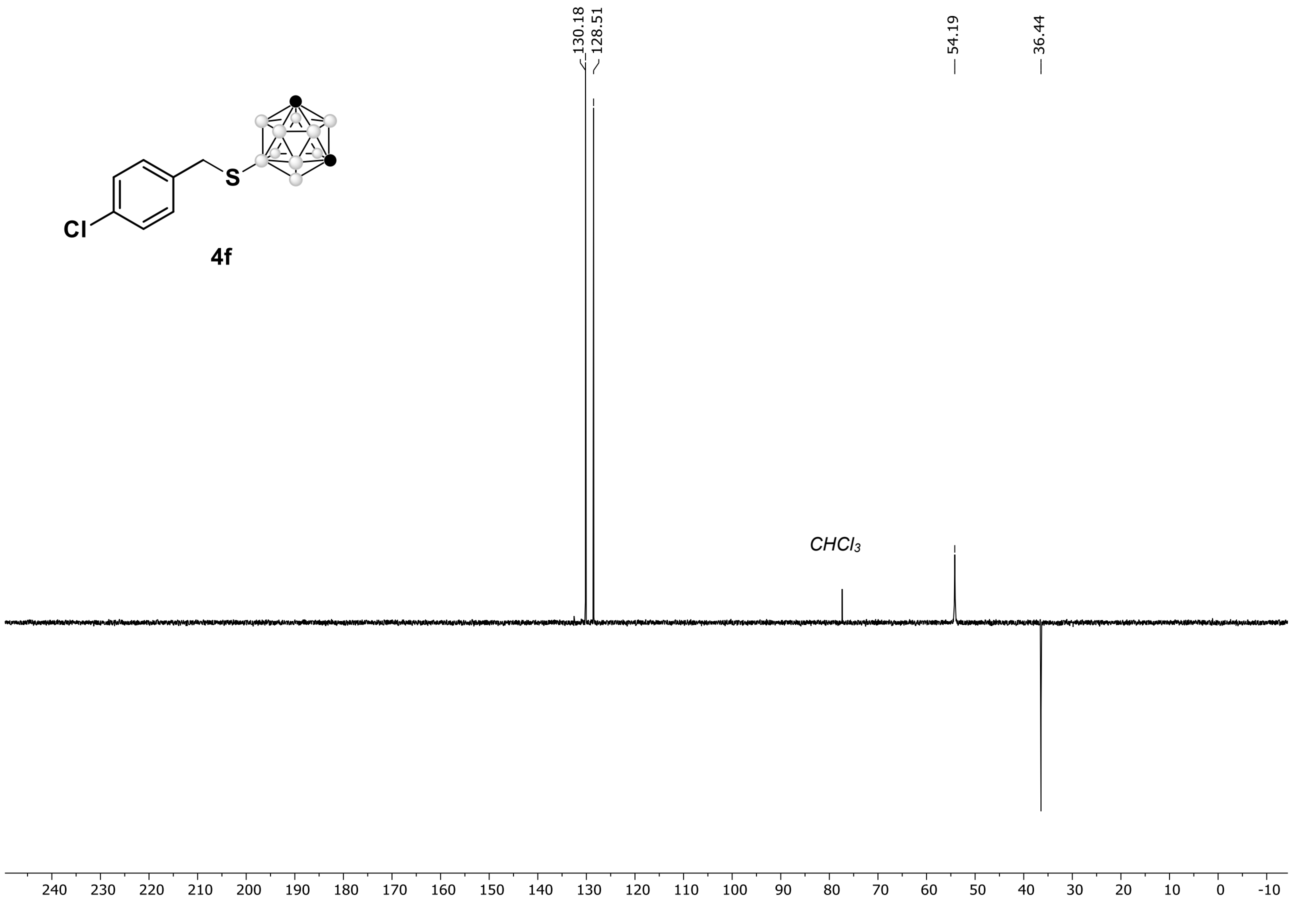

$^{11}B\{^{1}H\}$-NMR (128 MHz, $CDCl_3$)

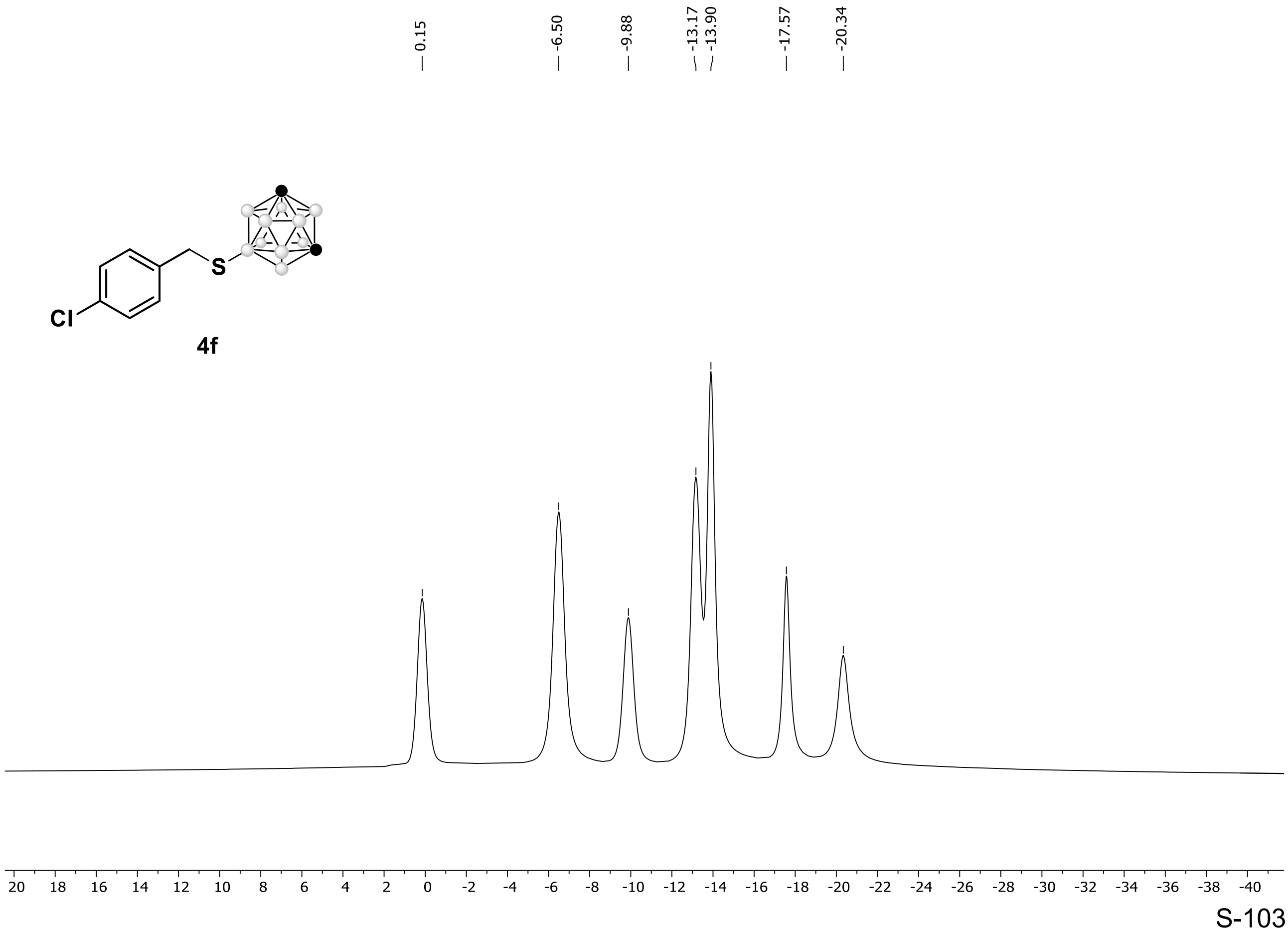

2D – COSY (400 MHz, $CDCl_3$)

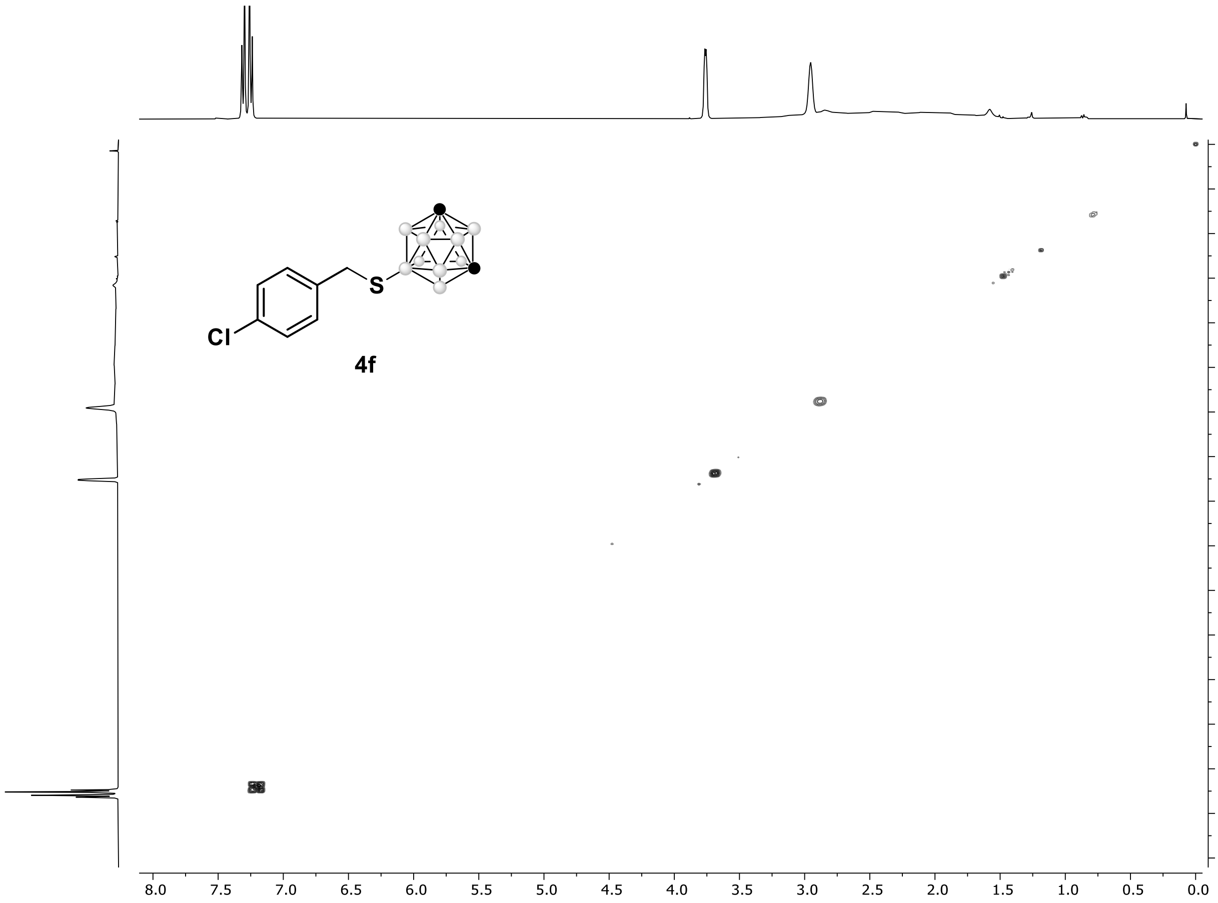

^{1}H-NMR (400 MHz, $CDCl_3$)

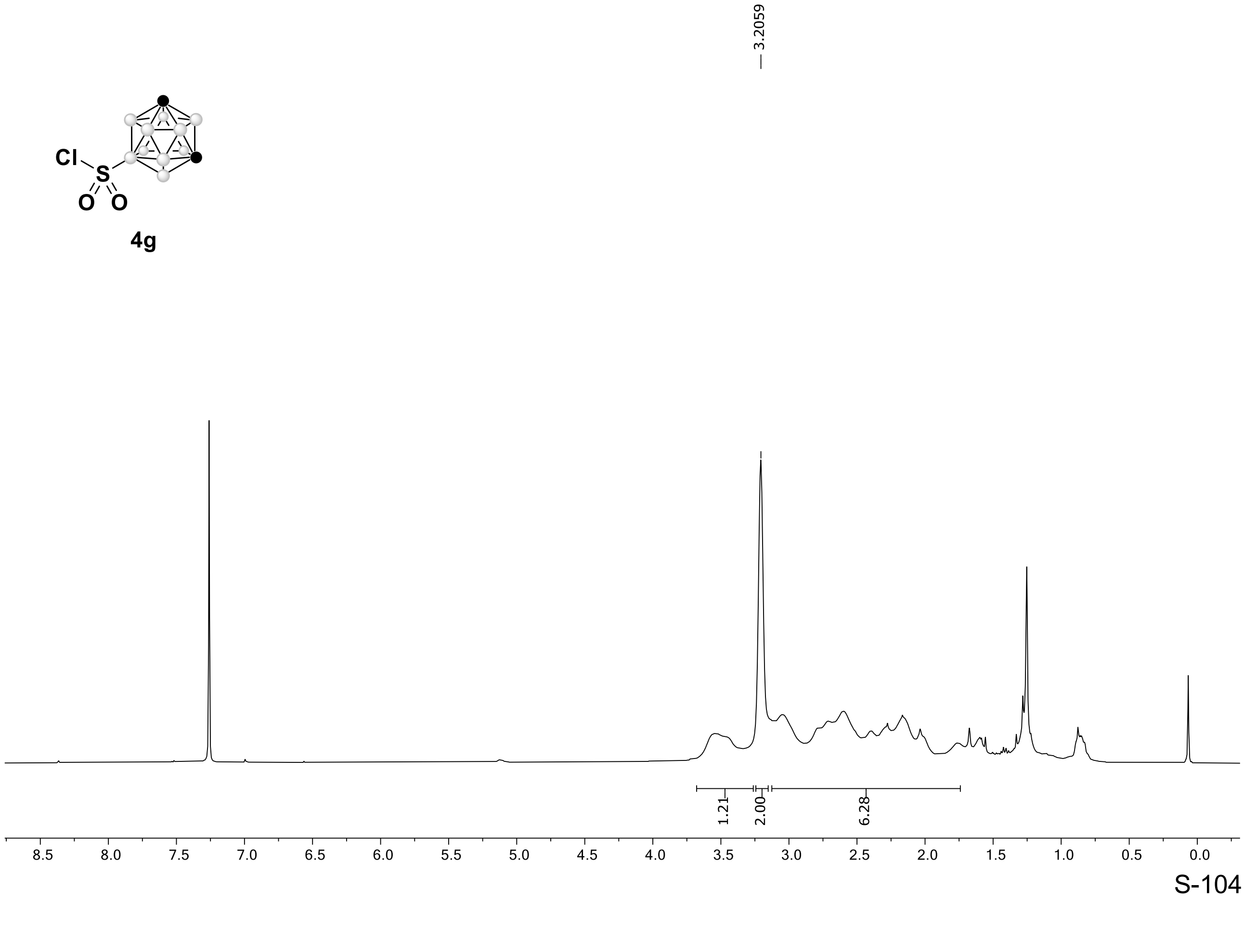

$^{13}C\{^1H\}$**-NMR** (100 MHz, $CDCl_3$)

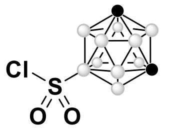

4g

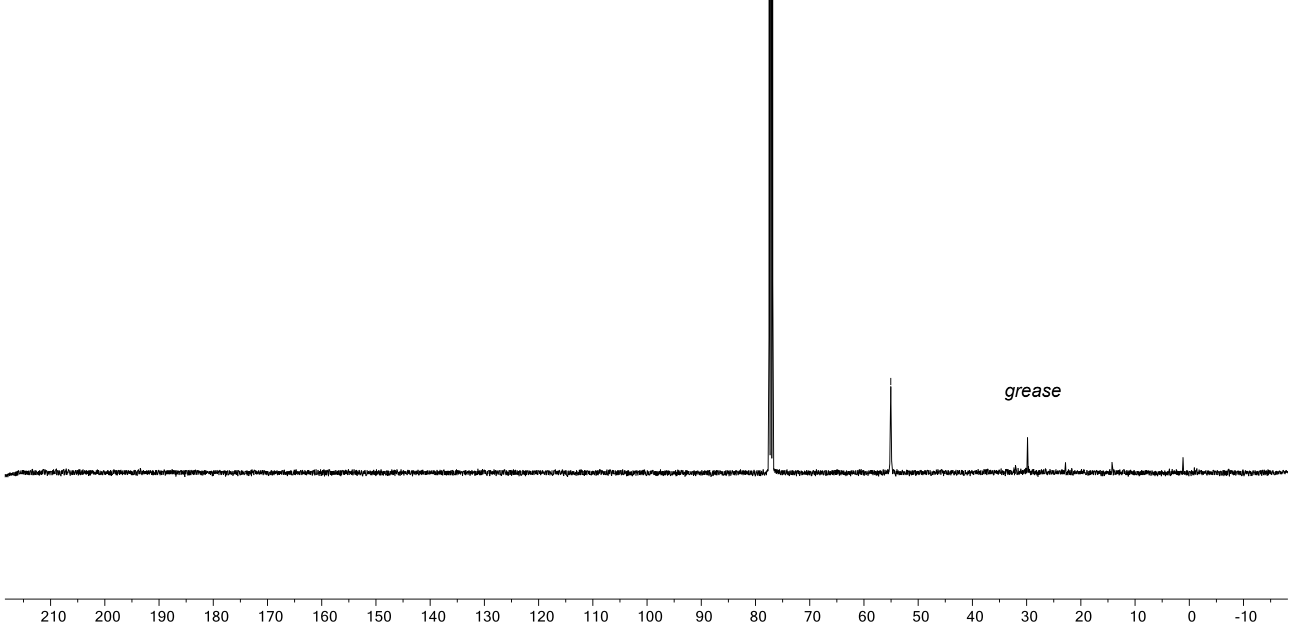

DEPT-135 (100 MHz, $CDCl_3$)

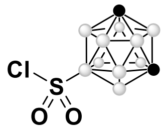

4g

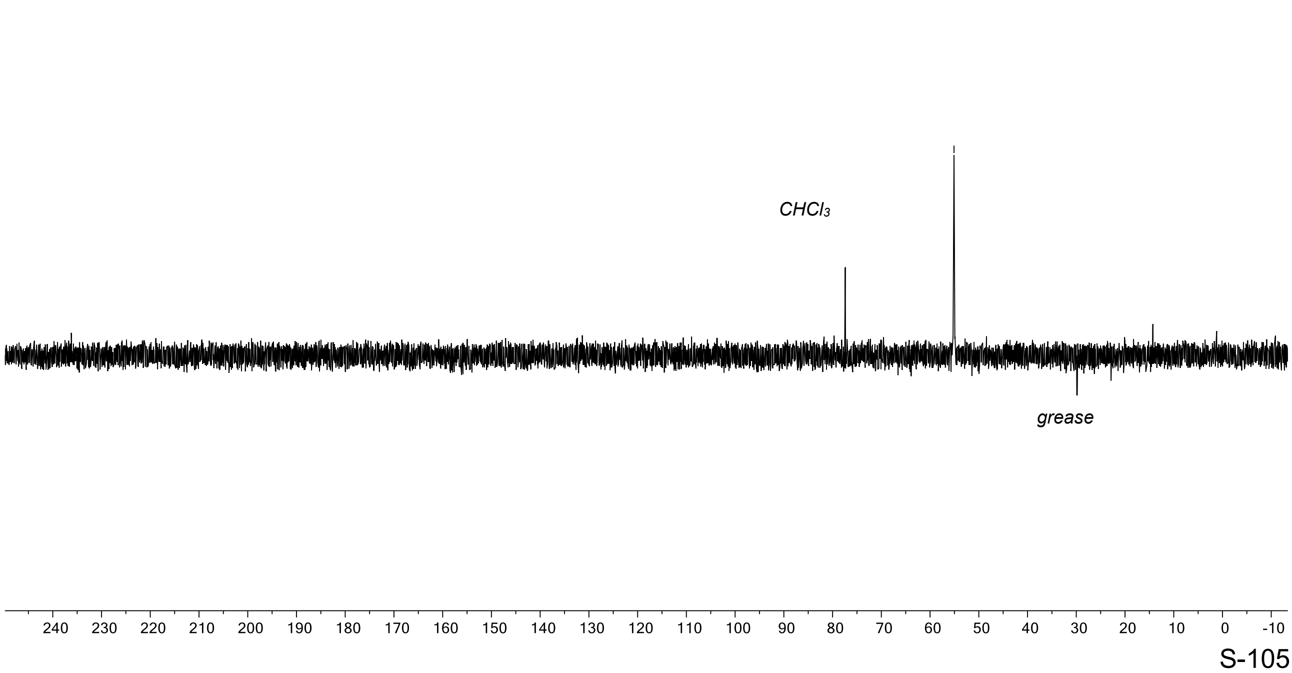

$^{11}B\{^1H\}$-NMR (128 MHz, $CDCl_3$)

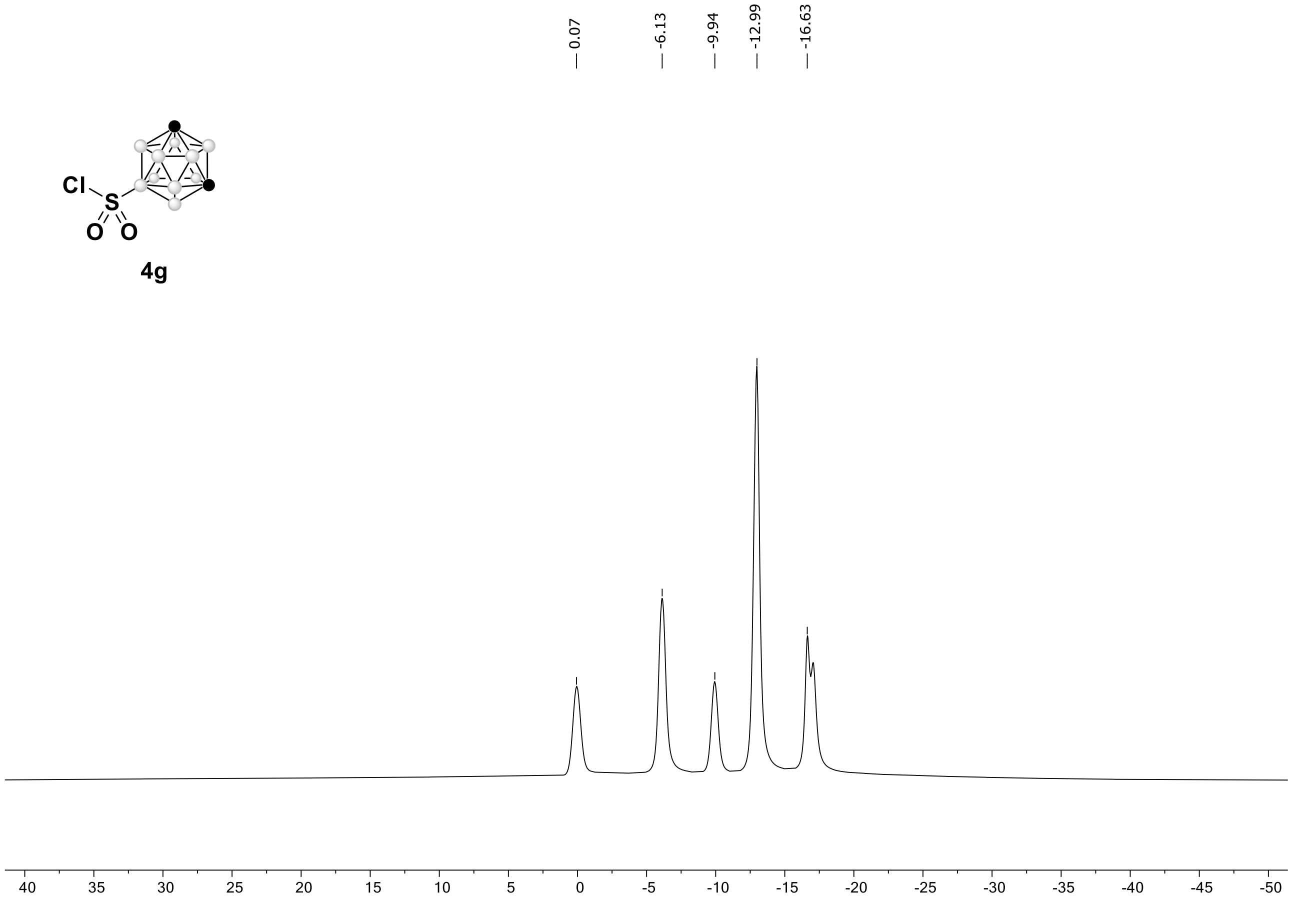

2D – COSY (400 MHz, $CDCl_3$)

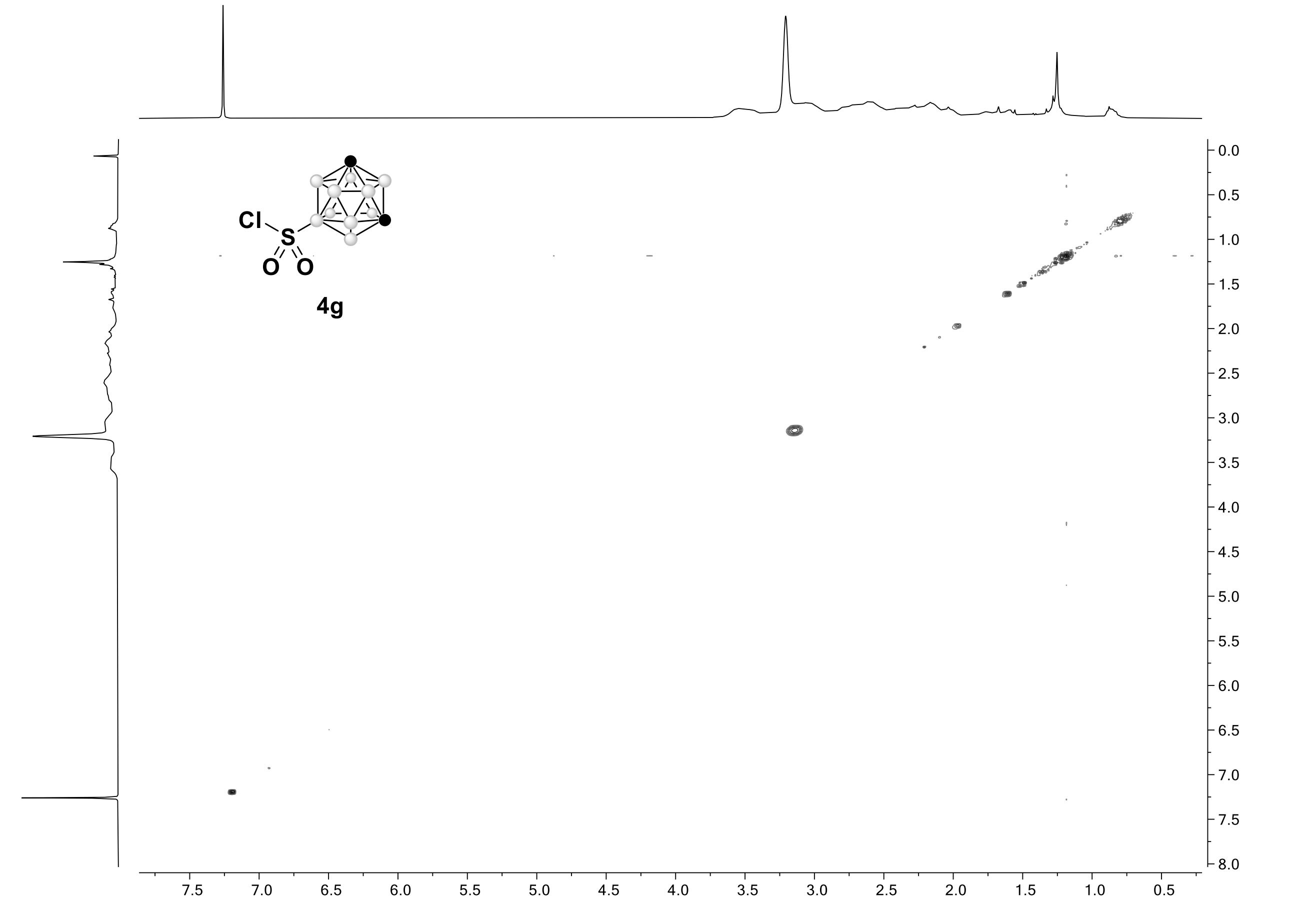

^{1}H-NMR (400 MHz, $CDCl_3$)

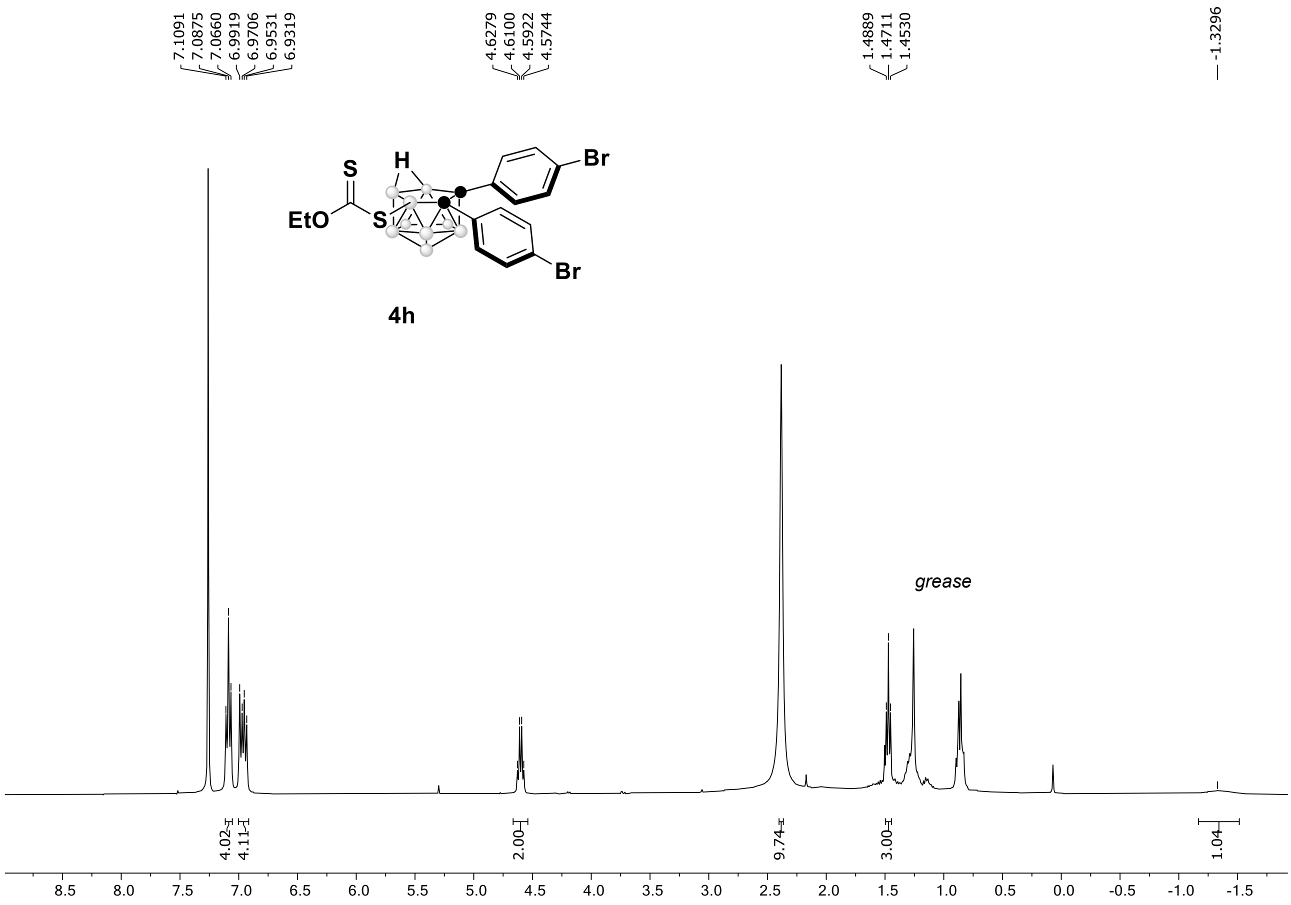

^{13}C{^{1}H}-NMR (100 MHz, $CDCl_3$)

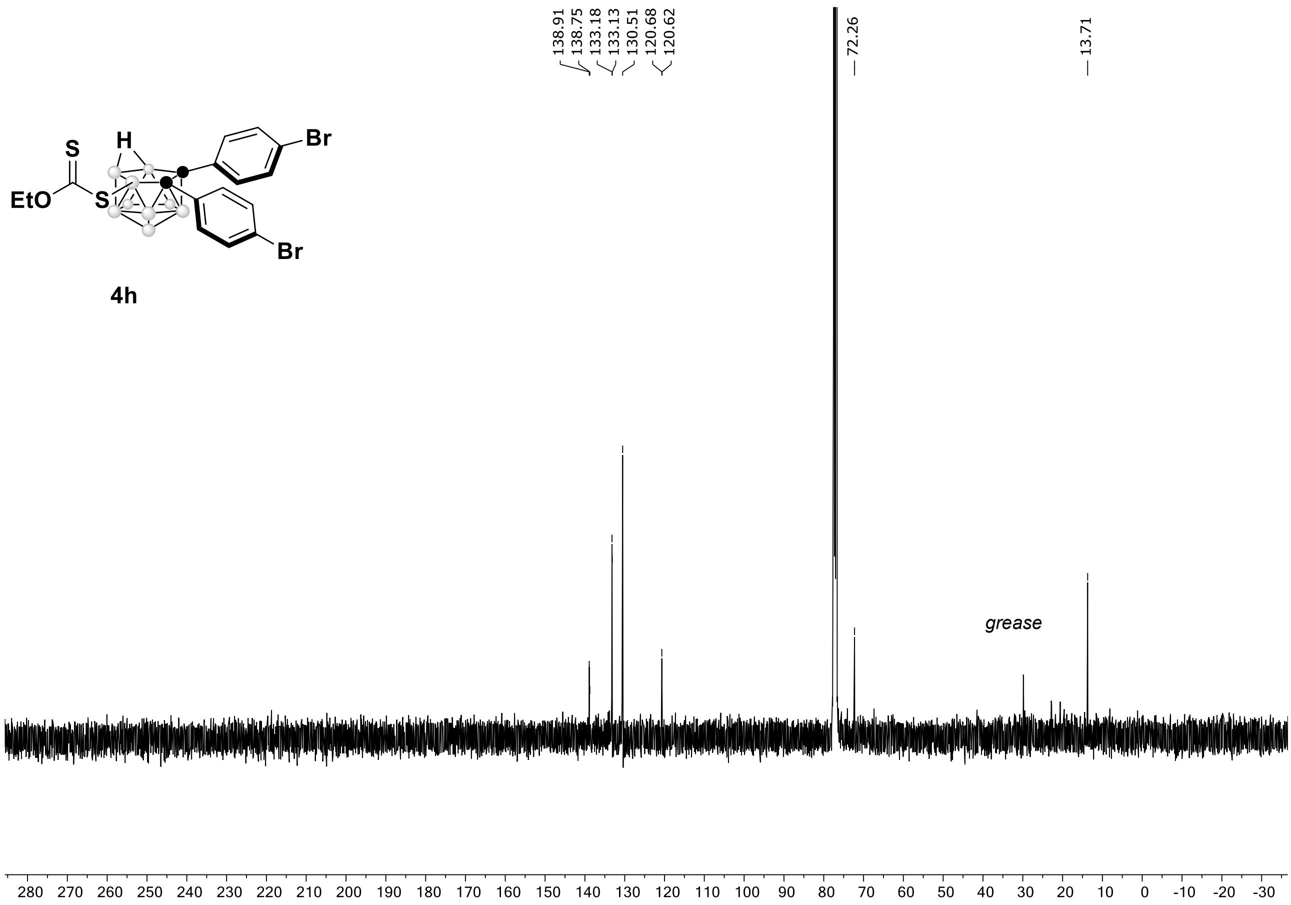

DEPT-135 (100 MHz, $CDCl_3$)

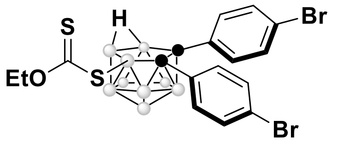

4h

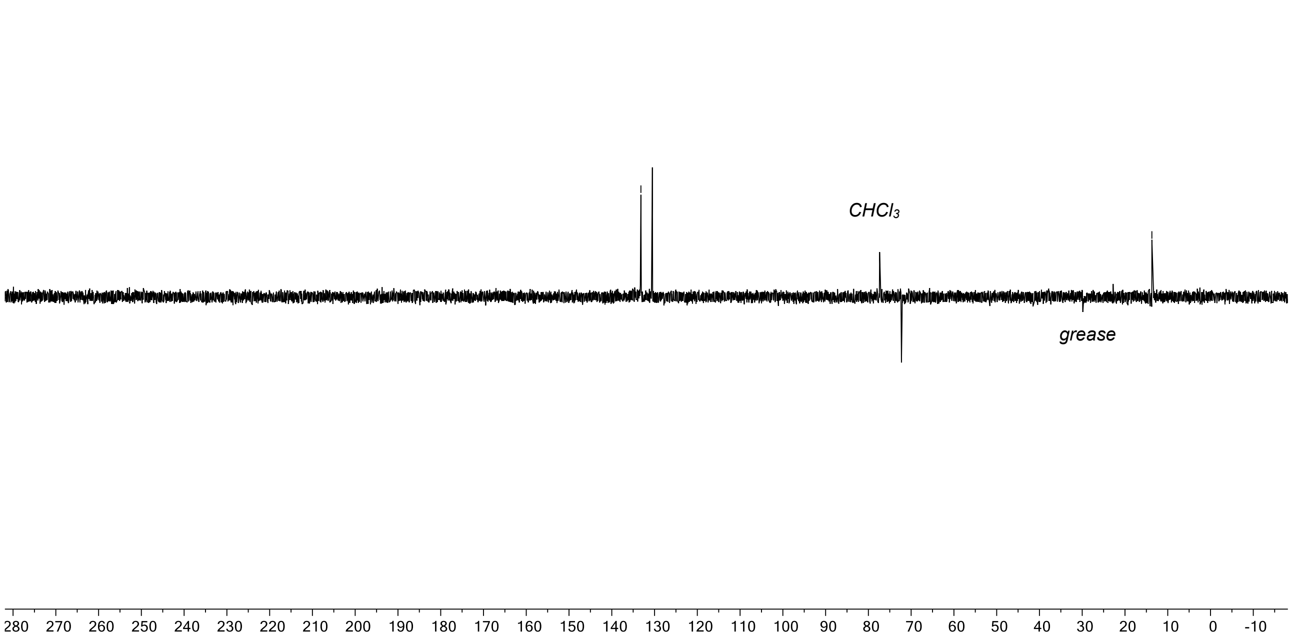

$^{11}B\{^1H\}$-NMR (128 MHz, $CDCl_3$)

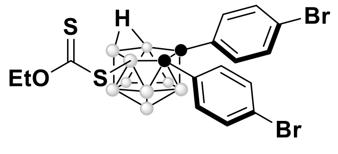

4h

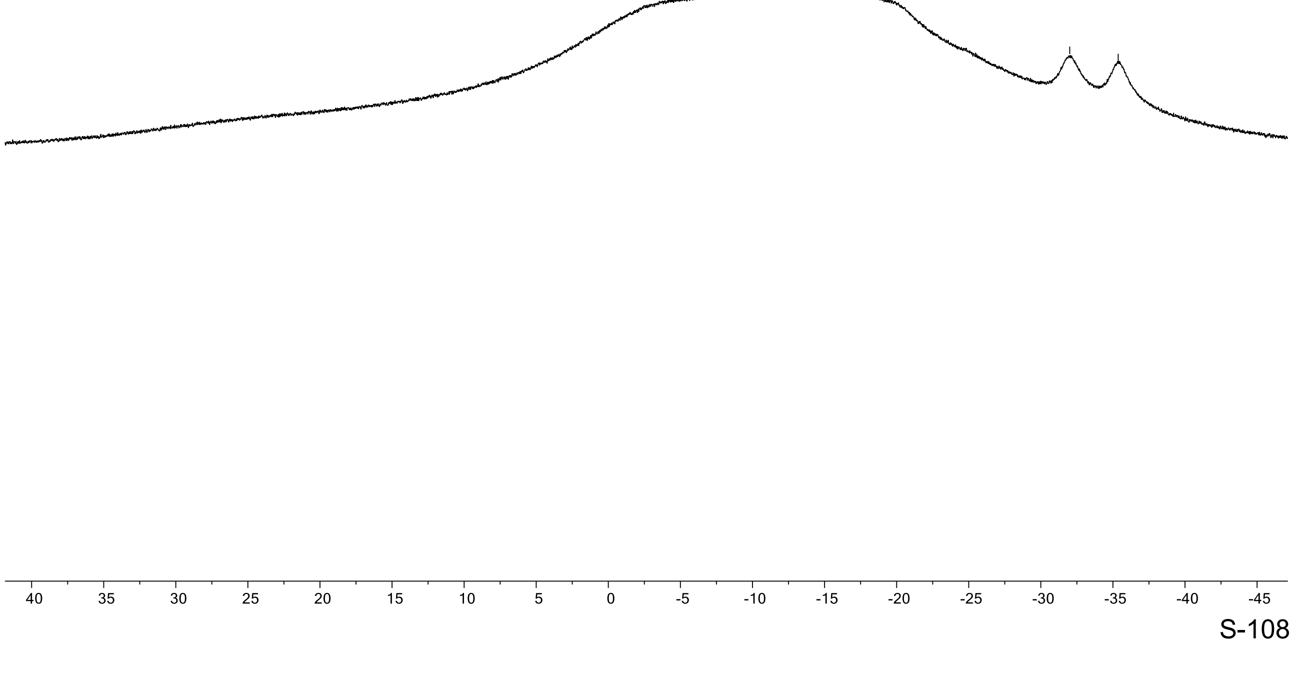

2D – COSY (400 MHz, $CDCl_3$)

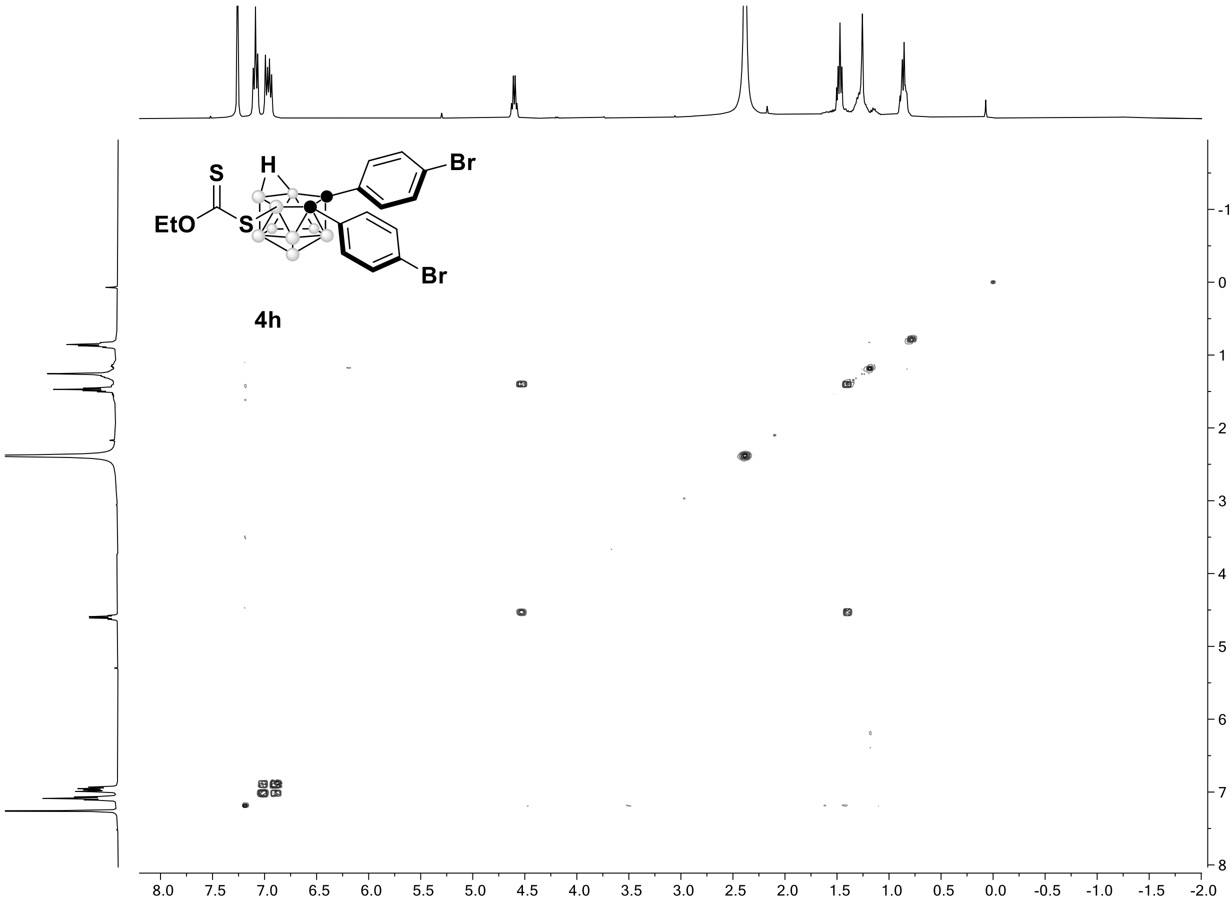

Supplement: Supplementary file 1 — Supporting File 1: anie72341‐sup‐0001‐SuppMat.pdf. [file ANIE-65-e7686382-s001.pdf]
